# Overcoming the Limitations of Transition-Metal Catalysis in the Chemoenzymatic Dynamic Kinetic Resolution (DKR) of Atropisomeric Bisnaphthols

**DOI:** 10.1021/acscentsci.4c01370

**Published:** 2024-11-05

**Authors:** Kun Wang, Wei Wang, Dingkai Lou, Jie Zhang, Changli Chi, Jan-E. Bäckvall, Xiang Sheng, Can Zhu

**Affiliations:** † Department of Chemistry, Fudan University, 2005 Songhu Road, Shanghai 200438, P.R. China; ‡ Tianjin Institute of Industrial Biotechnology, Chinese Academy of Sciences, Tianjin 300308, P.R. China; § National Center of Technology Innovation for Synthetic Biology and Key Laboratory of Engineering Biology for Low-Carbon Manufacturing, Tianjin 300308, P.R. China; ∥ Department of Organic Chemistry, Arrhenius Laboratory, Stockholm University, SE-10691 Stockholm, Sweden

## Abstract

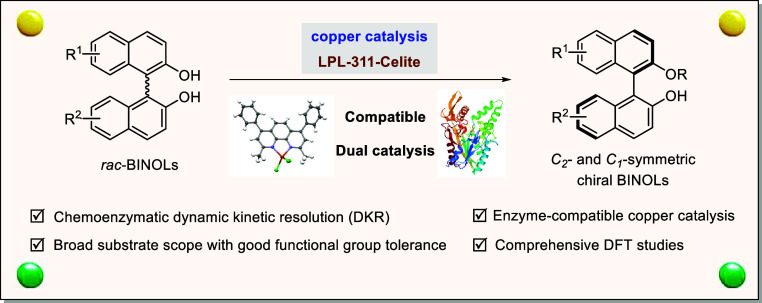

Chemoenzymatic dynamic kinetic resolution (DKR), combining a metal racemization catalyst with an enzyme, has emerged as an elegant solution to transform racemic substrates into enantiopure products, while compatibility of dual catalysis is the key issue. Conventional solutions have utilized presynthesized metal complexes with a fixed and bulky ligand to protect the metal from the enzyme system; however, this has been generally limited to anionic ligands. Herein, we report our strategy to solve the compatibility issue by employing a reliable ligand that firmly coordinates *in situ* to the metal. Such a reliable ligand offers π* orbitals, allowing additional metal-to-ligand d−π* back-donation, which can significantly enhance coordination effects between the ligand and metal. Therefore, we developed an efficient DKR method to access chiral BINOLs from racemic derivatives under dual copper and enzyme catalysis. In cooperation with lipase LPL-311-Celite, the DKR of BINOLs was successfully realized with a copper catalyst via *in situ* coordination of BCP (**L8**) to CuCl. A series of functionalized *C*_2_- and *C*_1_-symmetric chiral biaryls could be synthesized in high yields with good enantioselectivity. The racemization mechanism was proposed to involve a radical-anion intermediate, which allows the axial rotation with a dramatic decrease of the rotation barrier.

## Abstract 2

A bathocuproine ligand offers d−π* back-donation to enhance the coordination effect in copper catalysis to enable the chemoenzymatic dynamic kinetic resolution (DKR) of atropisomeric bisnaphthols.

## Introduction

**Figure 1:**
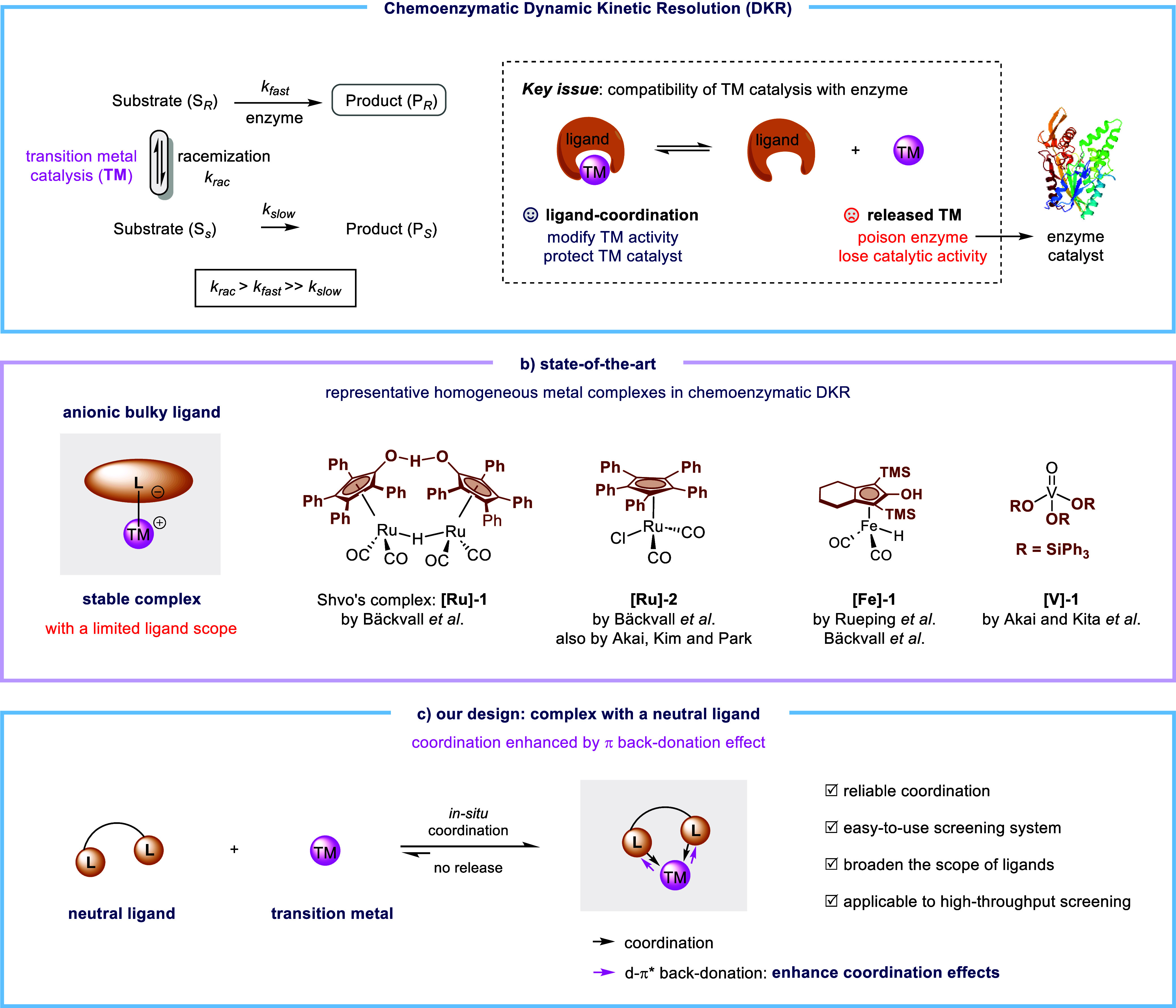
Chemoenzymatic dynamic kinetic resolution (DKR).

Chemoenzymatic dynamic kinetic resolution (DKR) has emerged as an efficient tool to transform racemic substrates into enantiopure products in 100% theoretical yield under cooperative enzyme and metal catalysis (Figure 1a).^1,2^ Compared to an enzymatic kinetic resolution (KR) process, DKR still relies on racemization catalysis of transition metals, in which each enantiomer of the substrate is continuously isomerized to the other during the resolution process.^3^ In this field, the compatibility of metal and enzyme catalysis has risen up as the key issue for the development of enzymatic DKR,^4,5^ given the fact that biocatalysts would get poisoned by released metal species, and the metal catalyst loses its activity at the same time.^6^ Classic strategies were to utilize complexes with a fixed and bulky ligand to protect the central metal, e.g. cyclopentadienyl ligand in Shvo’s and Bäckvall’s ruthenium catalyst,^7a−7c^ a Knölker iron catalyst,^7f,7g^ and triphenylsiloxyl ligand in a vanadium catalyst,^7d,7e^ while they are generally limited to anionic ligands (Figure 1b).^7^ Moreover, fast optimization and modification of ligands for metal catalysts would be time-consuming, since metal complexes have to be presynthesized. In contrast, *in situ* coordination of a free and neutral ligand to the metal would greatly broaden the scope of the metal catalysts and could provide an easy-to-use screening system for enzymatic DKR development (Figure 1c). The challenge will be the design of suitable ligands for the metals. Our strategy focuses on the development of unfixed but reliable ligands, which can firmly coordinate to the central metal, thus avoiding the release of free metal to poison the enzyme catalyst.^8^ Such reliable ligands with π* orbitals allow additional metal-to-ligand d−π* back-donation, which can significantly enhance coordination effects between the ligand and metal.

**Figure 2:**
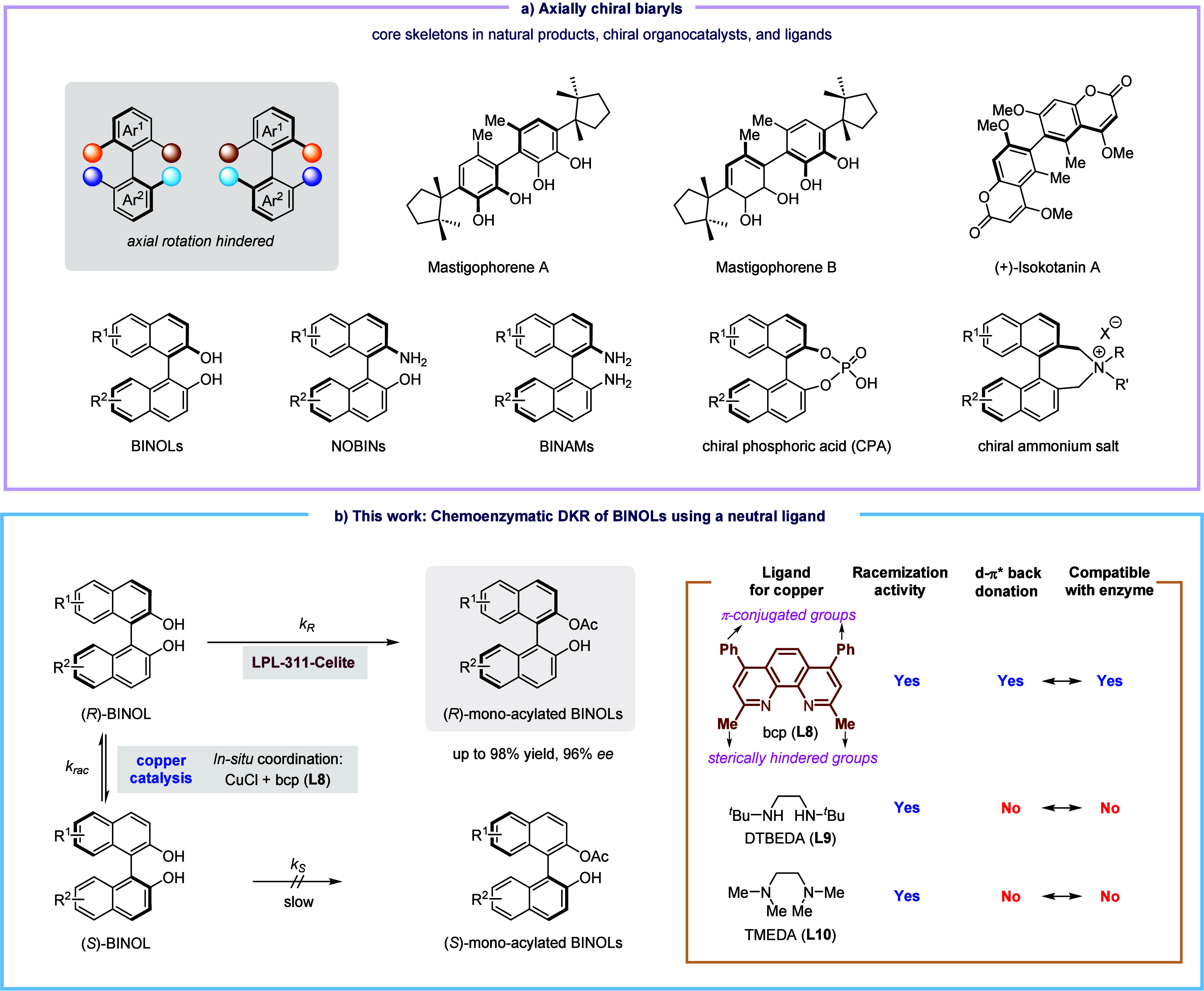
DKR of atropisomeric bisnaphthols.

On the other hand, atropisomeric biaryls serve as building blocks for various optically active natural products and have found crucial applications in versatile chiral catalysts and ligands for transition metals, e.g. 2,2′-dihydroxy-1,1′-biaryls (BINOLs) and their derivatives (Figure 2a).^9,10^ In addition to asymmetric oxidative coupling^11^ and chiral resolution approaches,^12^ the groups of Tsuji,^13a^ Zhao,^13b^ Sibi,^13c^ M. D. Smith,^13d^ A. D. Smith,^13e^ Wang,^13f^ and Tan^13g^ independently developed catalytic KR approaches to chiral BINOLs, which improved the synthetic efficiency impressively, but with reaction yields being below 50%. Targeting the DKR of BINOLs, pioneering work has realized the interconversion of each enantiomer, although stoichiometric copper and chiral diamine ligand are required to irreversibly produce the matched ternary metal complex with a BINOL substrate.^14^ In 2018, Akai and co-workers elegantly developed the chemoenzymatic DKR^15^ of BINOLs using a ruthenium complex (**[Ru]-2**, 10 mol %), which bears a fixed pentaphenylcyclopentadienyl ligand.^16^ However, the utilization of an *in situ* coordination strategy in the chemoenzymatic DKR of atropisomeric biaryls is still undeveloped, especially that based on a 3d transition metal with a neutral ligand. Herein, we disclose our recent development of ligand-accelerated copper catalysis for the efficient chemoenzymatic DKR of atropisomeric biaryls (Figure 2b). A bathocuproine (BCP, **L8**) ligand coordinates firmly *insitu* to CuCl, a simple and readily accessible 3d transition metal salt, so that this copper catalyst is compatible with the enzyme catalyst Lipase LPL-311. The BCP-ligand offers π* orbitals allowing additional metal-to-ligand d−π* back-donation to enhance the coordination effect, which can overcome the limited scope of transition-metal catalysts in the chemoenzymatic DKR.

## Results and Discussion

### Optimization of the Reaction Conditions

**Figure 3:**
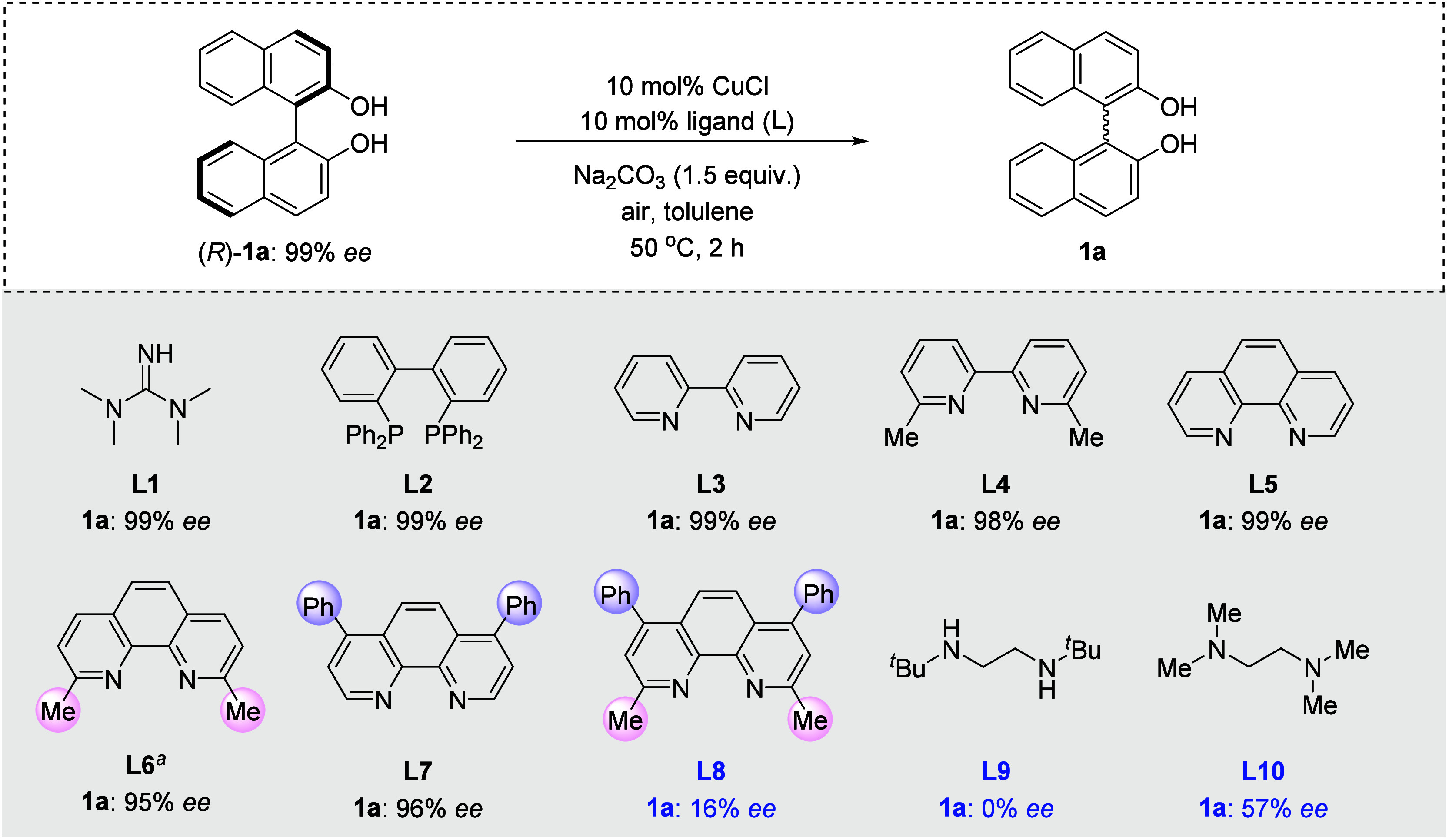
Ligand effect for the copper-catalyzed racemization of (*R*)-BINOL. The reaction was carried out using (*R*)-**1a** (0.1 M), CuCl (10 mol %), ligand (10 mol %), and Na_2_CO_3_ (1.5 equiv) at 50 °C in toluene for 2 h under an air atmosphere. Legend: (a) a mixed solvent (toluene:DCM = 7:3) was used.

Stoichiometric amounts of both copper and chiral amine are necessary to combine with BINOL, forming a BINOL-Cu-amine ternary complex, in which one enantiomer of BINOL can be transformed to the other via axial rotation.^14^ We anticipated that a suitable ligand may trigger the release of copper from the BINOL-Cu-amine ternary complex, thus finishing the catalytic cycle of racemization. Therefore, in this case, only a catalytic amount of copper and a ligand can be enough for BINOL racemization. A collection of nitrogen-containing bidentate ligands were studied together with the catalyst CuCl (Figure 3). In the presence of guanidine (**L1**), bisphosphine (**L2**), bipyridine (**L3**), or 1,10-Phen (**L5**) ligand, the copper catalyst displayed no activity on BINOL racemization, as shown by the recovery of **1a** with 99% ee in all cases. Only a slight decrease in enantiomeric excess was detected by using dimethyl bipyridine (**L4**, 98% ee) or 2,9-dimethyl-1,10-Phen (**L6**, 95% ee). The reaction also gave a slight loss of enantiomeric purity with 1,10-Phen ligand **L7**, in which two phenyl groups are installed in the 4,7-positions. To our delight, on simultaneously introducing the methyl groups at the 2,9-positions together with two phenyl groups at the 4,7-positions, a 1,10-Phen derivative ligand (**L8**) enhanced the efficiency of racemization catalysis remarkably, thus affording almost racemic BINOL (16% ee). A faster racemization process was observed in the reaction with the ligand **L9**, bearing two bulky ^*t*^Bu groups in an aliphatic diamine skeleton. Moreover, **L10**, a tertiary diamine ligand, was also able to be considered as a candidate ligand for the DKR development, as shown by the recovery of BINOL with 57% ee, although its efficiency was not as high as those of **L8** and **L9**.

With the optimal ligands for BINOL racemization in hand, we turned to evaluating the performance of biocatalysts for the KR process.^15^ Biocatalyst screening showed that LPL-311, a lipoprotein lipase from *Pseudomonas* sp., could be immobilized on Celite and gave the highest *E* value in KR. Aiming to develop a chemoenzymatic DKR^17^ of BINOLs, we next combined the copper racemization catalyst with the lipase-catalyzed KR system. A further detailed survey of solvents, base, acyl donor, and temperature (see Tables S3–S6 for more details) revealed that monoacylated product (*R*)-**2a** could be obtained in 85% yield with 96% ee, with the catalyst loading of CuCl and its ligand **L8** as low as 1 mol % (entry 1, Table 1). Neither CuCl nor the ligand (**L8**) could be absent from the reaction (entries 2 and 3, Table 1). Na_2_CO_3_ was surprisingly found as an indispensable element to ensure the DKR process, and without Na_2_CO_3_ the enzymatic resolution process was slowed down (entry 4, Table 1). Moreover, Na_2_CO_3_ probably also acts as a deacidification reagent to protect the alkaline ligand. The enzyme catalyst becomes incompatible with the copper system by replacing **L8** with **L9** or **L10**, given the fact that lower conversion and enantioselectivity was observed in the DKR reactions (entries 5 and 6, Table 1). Moreover, high enantioselectivity (97% ee for **L9**, >99% ee for **L10**) was detected in the recovered BINOL with the ligand of **L9** or **L10**, implying that the racemization process was dramatically retarded in the DKR reaction conditions, compared to the isolated racemization evaluation in Figure 3. These outcomes point to the incompatibility of dual catalysis of copper (with **L9** or **L10**) and lipase with each other. Lipases other than LPL-311-Celite failed to give better performance (entries 7–10, Table 1). Finally, when isopropenyl acetate was employed as the acyl donor, the DKR reaction became slower (entry 11, Table 1).

**Table 1:**
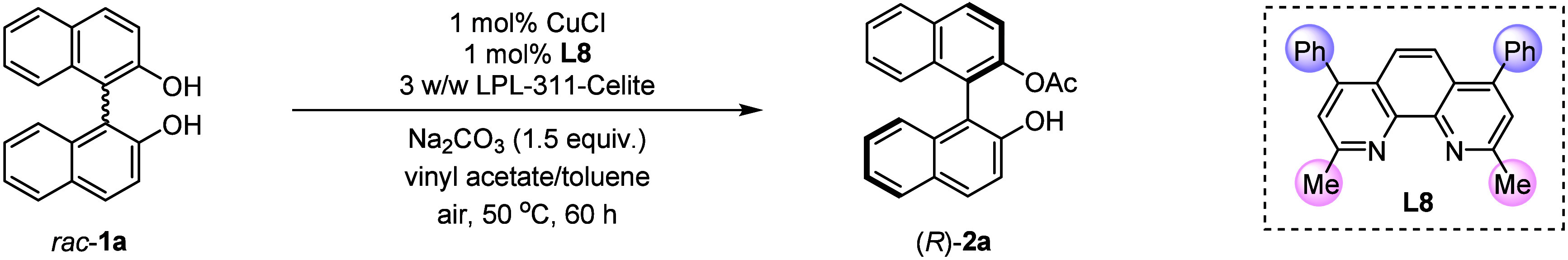
Deviation from Standard Conditions and Control Setting^a^

entry	deviations	yield of (*R*)-**2a** (%)^b^	ee of (*R*)-**2a** (%)^c^	recovery of (*S*)-**1a** (%)^b^	ee of recovery (*S*)-**1a** (%)^c^
1	none	85	96	<5	<5
2	no CuCl	45	96	55	73
3	no **L8**	59	60	41	>99
4	no Na_2_CO_3_	21	97	79	11
5	**L9** instead of **L8**	57	84	40^*d*^	97
6	**L10** instead of **L8**	65	50	30^*e*^	>99
7	CALB	12	20	88	<5
8	PS-IM	35	90	47	<5
9	Lipase CL	3	12	97	<5
10	Lipozyme RM	55	60	42	59
11	isopropenyl acetate	64	95	33	27

a The reaction was carried out using *rac*-**1a** (0.08 M), CuCl (1 mol %), ligand **L8** (1 mol %), LPL-311-Celite (3 w/w), and Na_2_CO_3_ (1.5 equiv) at 50 °C in vinyl acetate/toluene (0.2 mL:1 mL) for 60 h under an air atmosphere.

b Determined by ^1^H NMR analysis with dibromomethane as the internal standard.

c Determined by chiral HPLC.

### Scope of the Reaction

**Figure 4:**
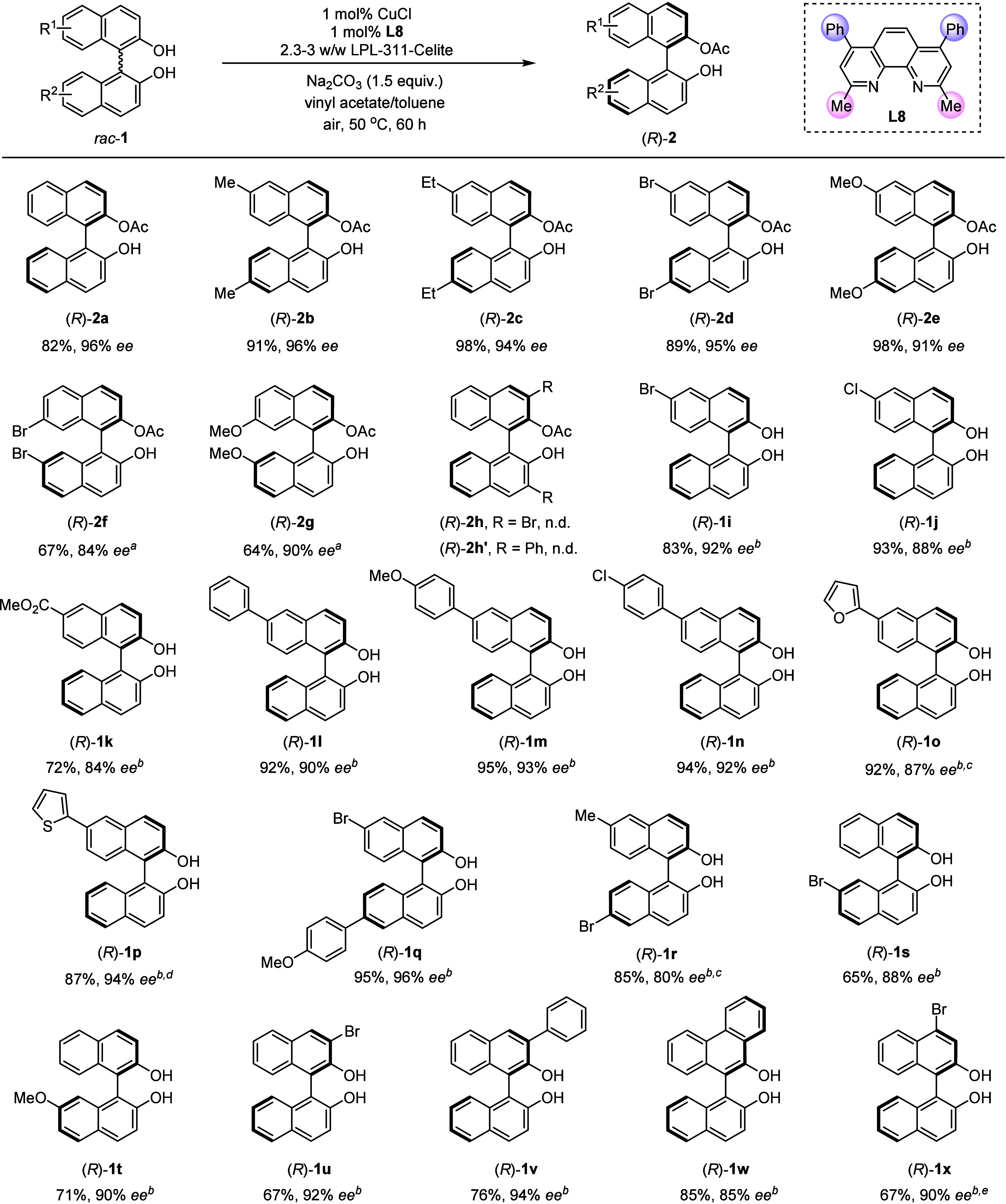
Enzymatic DKR of *C*_2_- and *C*_1_-symmetric BINOLs. The reaction was conducted with *rac*-**1** (0.08 M), CuCl (1 mol %), ligand **L8** (1 mol %), LPL-311-Celite (2.3–3 w/w), and Na_2_CO_3_ (1.5 equiv) at 50 °C in vinyl acetate/toluene (0.2 mL:1 mL) for 60 h under an air atmosphere. Legend: (a) reaction conducted with CuCl (5 mol %) and **L8** (5 mol %) under an oxygen atmosphere (1 atm); (b) hydrolysis of isolated monoacetylated product with K_2_CO_3_ (2 equiv) in MeOH at rt for 0.5 h; (c) reaction run for 48 h; (d) reaction run for 36 h; (e) reaction conducted under an oxygen atmosphere (1 atm).

Under the optimized DKR conditions, *C*_2_-symmetric 2,2′-binaphthols bearing dimethyl, diethyl, dibromo, or dimethoxyl substituents at the 6- and 6′-positions were converted into the corresponding optically active products (*R*)-**2b**, (*R*)-**2c**, (*R*)-**2d**, and (*R*)-**2e** in 89–98% isolated yields and excellent ees (91–96%), respectively (Figure 4). Racemization became much slower with functional groups, such as bromo or methoxyl groups at the 7- and 7′-positions, probably due to the increase of steric hindrance during axial rotation. However, this process could be significantly accelerated with a higher loading of copper catalyst under an oxygen atmosphere (balloon, 1 atm); thus, the DKRs of racemic BINOLs **1f** and **1g** were also successful in delivering the corresponding monoacylated BINOLs **2f** and **2g**, respectively, with slightly lower enantioselectivity (84% and 90% ee). However, the enzymatic resolution process became inert with two bromo (**1h**) or phenyl (**1h′**) substituents installed at 3- and 3′-positions. *C*_1_-symmetric 2,2′-binaphthol skeletons have attracted considerable attention because of their important applications in chiral auxiliaries and ligands.^18^ This DKR method turned out to be applicable to *C*_1_-symmetric 2,2′-binaphthols under dual copper/lipase catalysis (Figure 4). For each single *C*_1_-symmetric 2,2′-binaphthol substrate in DKR, a pair of ester products would be produced as a mixture, given the fact that esterification was able to occur at either hydroxyl group. Therefore, for compounds **1i**–**1x**, the isolated ester products were hydrolyzed by K_2_CO_3_ in MeOH after DKR (cf. note (b) in Figure 4), leading to chiral BINOLs **1i**–**1x**. Functional groups of Br, Cl, and CO_2_Me at the 6-position on one naphthene ring were found to be nicely tolerated (**1i**–**1k**). Substituents could be extended to aromatic and heteroaromatic rings, delivering chiral products **1l**–**1p** in 87–95% yields with high enantioselectivity (87–94%). *Rac*-BINOLs **1q** and **1r** with two different groups at 6- and 6′-positions also reacted smoothly. Reaction yields were decreased with a substrate bearing a Br or MeO group at the 7-position, but still with high enantioselectivity. The reaction works equally well when groups (bromo, phenyl, or fused benzene ring) occupied one 3-position. It is noteworthy that synthesis of such chiral BINOLs with high enantioselectivity is greatly challenging with current asymmetric oxidative approaches.^11^ Further studies showed that substitution at the 4-position was also compatible with this DKR approach, as shown by the formation of (*R*)-**1x** in 67% yield with 90% ee. The slightly lower yields in the cases of **1s**, **1u**, and **1x** are probably due to a limited racemization reaction rate (for details, see the Supporting Information). The low loading of a non-noble metal catalyst (1 mol % CuCl) endows the method with application potentials in the synthesis of axially chiral skeletons on a large scale.

**Figure 5:**
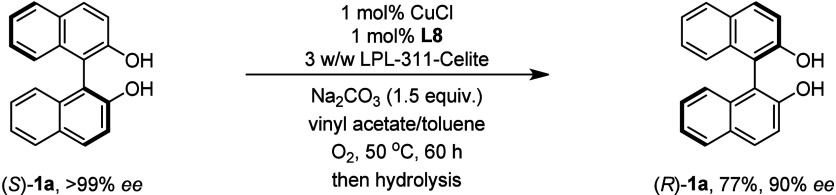
Axial chirality inversion of (*S*)-**1a**.

Finally, it is encouraging to observe that the axial chirality could be eventually inversed after DKR and hydrolysis protocols; thus, (*R*)-**1a** was obtained in 77% yield with 90% ee from (*S*)-**1a** of 99% ee (Figure 5). The success of axial chirality inversion endorses the high efficiency and compatibility of Cu/**L8** in cooperation with lipase catalysis.

### Mechanistic Studies

**Figure 6:**
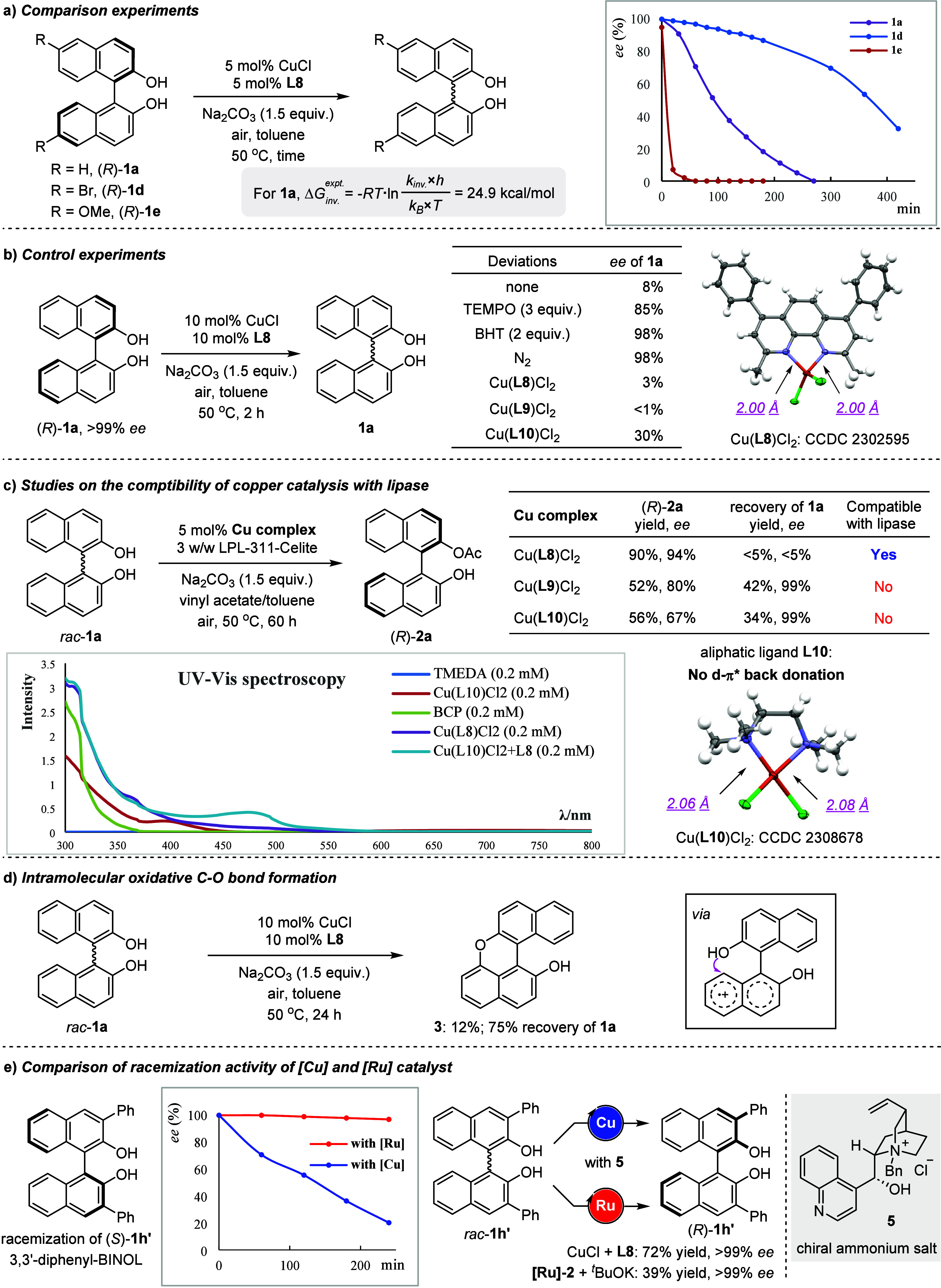
Mechanistic studies.

To gain deeper insight into the racemization mechanism, comparison and control experiments were carried out as shown in Figure 6. Three parallel experiments of (*R*)-**1a**, (*R*)-**1d**, and (*R*)-**1e** indicated that electron-rich BINOLs were racemized much faster and thus were more reactive under copper catalysis (Figure 6a). These results imply a pathway of electron transfer from the BINOL moiety during racemization. The energy barrier for the chirality inversion of (*R*)-**1a** under copper catalysis was measured to be 24.9 kcal/mol according to the Eyring equation (for details, see the Supporting Information). Control experiments with (*R*)-**1a** were performed respectively (Figure 6b). The dynamic process was almost suppressed when (2,2,6,6-tetramethylpiperidin-1-yl)oxyl (TEMPO) or butylated hydroxytoluene (BHT) was added, suggesting a possible radical pathway for BINOL racemization. The axial chirality of BINOL stopped flipping when the air atmosphere was replaced by a N_2_ atmosphere, indicating that oxygen plays an indispensable role in maintaining the activity of the copper catalyst. Next, we synthesized three copper(II) complexes, Cu(**L8**)Cl_2_, Cu(**L9**)Cl_2_, and Cu(**L10**)Cl_2_, and all of these coordination compounds exhibit equal activity on BINOL racemization, indicating the intermediacy of Cu(II) species.^19^ However, only Cu(**L8**)Cl_2_ gives excellent performance in cooperation with enzyme catalysis for the DKR of BINOL. In contrast, in the presence of Cu(**L9**)Cl_2_ or Cu(**L10**)Cl_2_, the enzyme lost partial activity and selectivity since much lower enantiomeric excesses of the produced **2a** (80% and 67% ee, respectively) were observed (Figure 6c). Moreover, **1a** was recovered with 99% ee in both cases, implying that Cu(**L9**)Cl_2_ or Cu(**L10**)Cl_2_ also became inactive for BINOL racemization in the enzymatic reaction. These outcomes point to the incompatibility of the enzyme catalyst with either Cu(**L9**)Cl_2_ or Cu(**L10**)Cl_2_, and consequently both sides lose catalytic activity simultaneously. Emphatically, atom distances of Cu–N in Cu(**L10**)Cl_2_ are 2.06 and 2.08 Å, which are significantly longer than those in Cu(**L8**)Cl_2_, i.e. 2.00 and 2.00 Å. These observations imply a stronger interaction of the ligand with the copper center in Cu(**L8**)Cl_2_, contributed by the d−π* back-donation from copper to π* orbital in **L8** to enhance the coordination effect. This conclusion was further supported by the experiment of UV–vis spectroscopy, and the absorption curve of the mixture of Cu(**L10**)Cl_2_ and **L8** is highly related to that of Cu(**L8**)Cl_2_, indicating the stronger interaction of copper with **L8** triggering ligand exchange of Cu(**L10**)Cl_2_ with **L8**. Therefore, the employment of unfixed but reliable ligand **L8** becomes the key element to ensure the compatibility of copper catalysis with enzyme catalysis. Furthermore, the oxidized product **3** was generated in 12% yield, forming an intramolecular C–O bond under the catalysis of CuCl with **L8** in the presence of Na_2_CO_3_, pointing to the intermediacy of radical-cation species (Figure 6d). Finally, comparison of copper with ruthenium catalysis for the racemization of 3,3′-diphenyl-BINOL (*S*)-**1h′** was conducted (Figure 6e). The catalytic system of CuCl/**L8** racemizes (*S*)-**1h′** efficiently, while ruthenium fails to show any activity, probably due to the steric effect of its bulky Cp^Ph^-anionic ligand with 3,3′-diphenyl substituents in **1h′**. The unsuccessful DKR of **1h′** shown in Figure 4 is attributed to the failed KR by the lipase. By using stoichiometric amounts of chiral ammonium salt **5**,^20^ the dynamic classical resolution of *rac*-**1h′** under copper catalysis led to (*R*)-**1h′** in 72% yield with >99% ee; in contrast, reaction with **[Ru]-2** provided a level of kinetic resolution, with (*R*)-**1h′** in only 39% yield being obtained.

**Figure 7:**
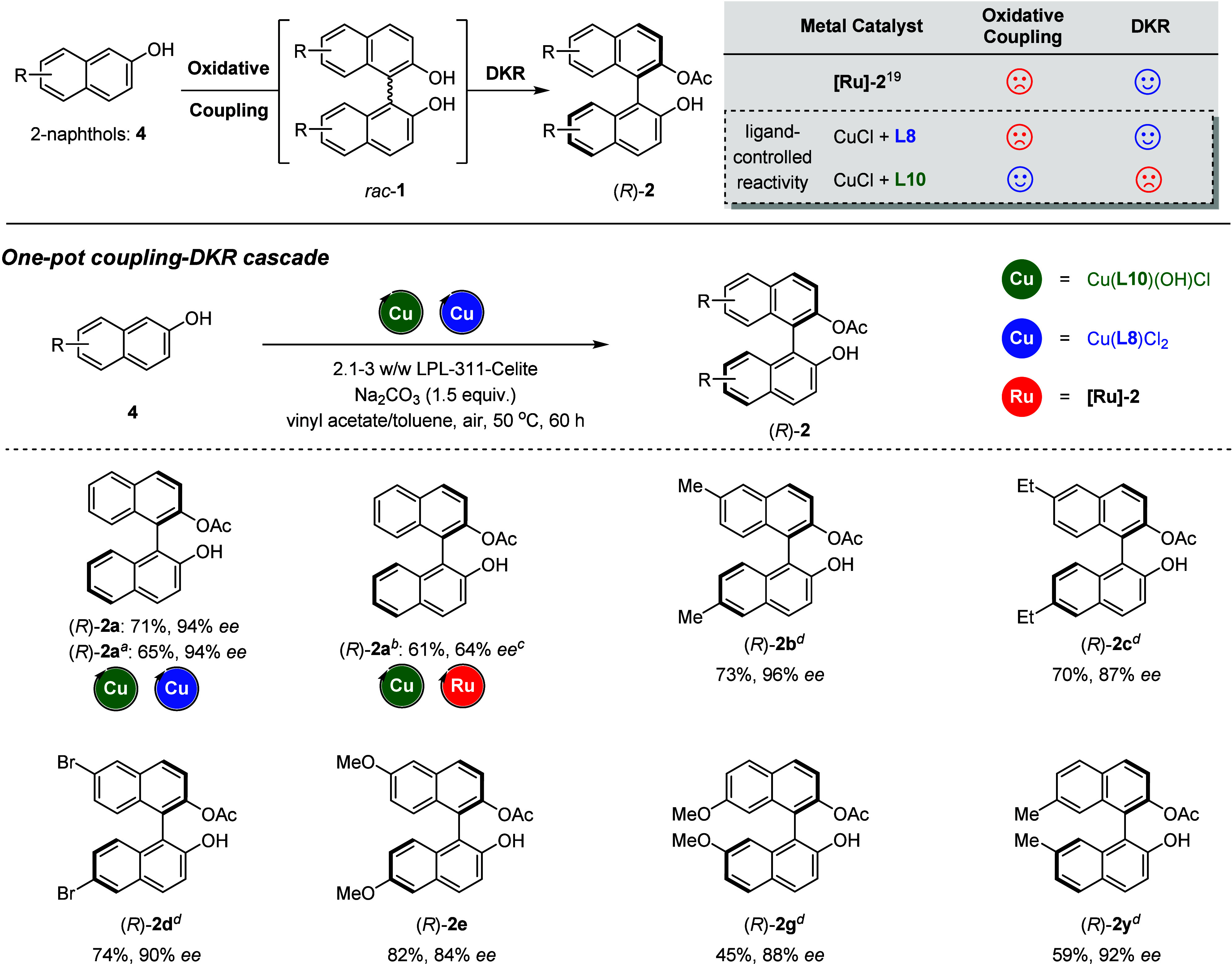
One-pot oxidative coupling-DKR cascade for the synthesis of *C*_2_-symmetric BINOLs. The reaction was carried out using **4** (0.2 mmol), Cu(**L10**)(OH)Cl (1 mol %), Cu(**L8**)Cl_2_(2.5 mol %), LPL-311-Celite (90.0 mg), and Na_2_CO_3_(0.75 equiv) at 40 °C in vinyl acetate/toluene (0.2 mL:1 mL) for 48 h under an air atmosphere. Legend: (a) reaction conducted by using CuCl (1 mol %) and **L10** (1 mol %) instead of Cu(**L10**)(OH)Cl, CuCl (2.5 mol %), and **L8** (2.5 mol %) instead of Cu(**L8**)Cl_2_; (b) reaction carried out using **4a** (0.2 mmol), Cu(**L10**)(OH)Cl (1 mol %), [Ru]-**2** (5 mol %), ^*t*^BuOK (5 mol %), LPL-311-Celite (90.0 mg), and Na_**2**_CO_3_ (0.75 equiv) at 40 °C in vinyl acetate/toluene (0.2 mL:1 mL) for 48 h under an air atmosphere; (c) (*S*)-**1a** concomitantly detected in 30% yield with 99% ee; (d) reaction conducted in vinyl acetate/toluene (0.3 mL:1 mL) under an oxygen atmosphere (1 atm).

To exhibit the advantage of utilizing a switchable ligand in the copper complex in the chemoenzymatic DKR, we performed the coupling-DKR cascade from 2-naphthols in one-pot transformations (Figure 7). Although copper catalyst Cu(**L8**)Cl_2_, as well as **[Ru]-2**,^21^ gave a satisfying performance in racemizing BINOL for the DKR development, both of them failed to promote an efficient oxidative coupling of 2-naphthol (**4**) to afford a racemic BINOL (**1**). However, it is convenient to modify the catalytic activity of the copper complex by replacing **L8** with **L10** as the ligand, providing excellent performance in the coupling step. Therefore, by combining the two copper catalysts with the lipase catalyst, (*R*)-**2a** was successfully delivered in 71% yield with 94% ee via one-pot oxidative coupling-DKR cascades directly starting from 2-naphthol. A slightly lower yield (65%) of (*R*)-**2a** but equally good ee (94%) was obtained by using CuCl together with **L8** and **L10** instead of their complexes. In contrast, the combination of catalysts based on hybrid transition metals (Cu+Ru) only provides (*R*)-**2a** in 61% yield with 64% ee and recovered BINOL (*S*)-**1a** with 99% ee, implying the incompatibility of different catalytic systems during the DKR process. The one-pot coupling-DKR cascade can be extended to the synthesis of *C*_2_-symmetric BINOL derivatives (*R*)-**2b**–**2e**, (*R*)-**2g**, and (*R*)-**2y** with good enantioselectivity under cooperative ternary catalytic systems.

**Figure 8:**
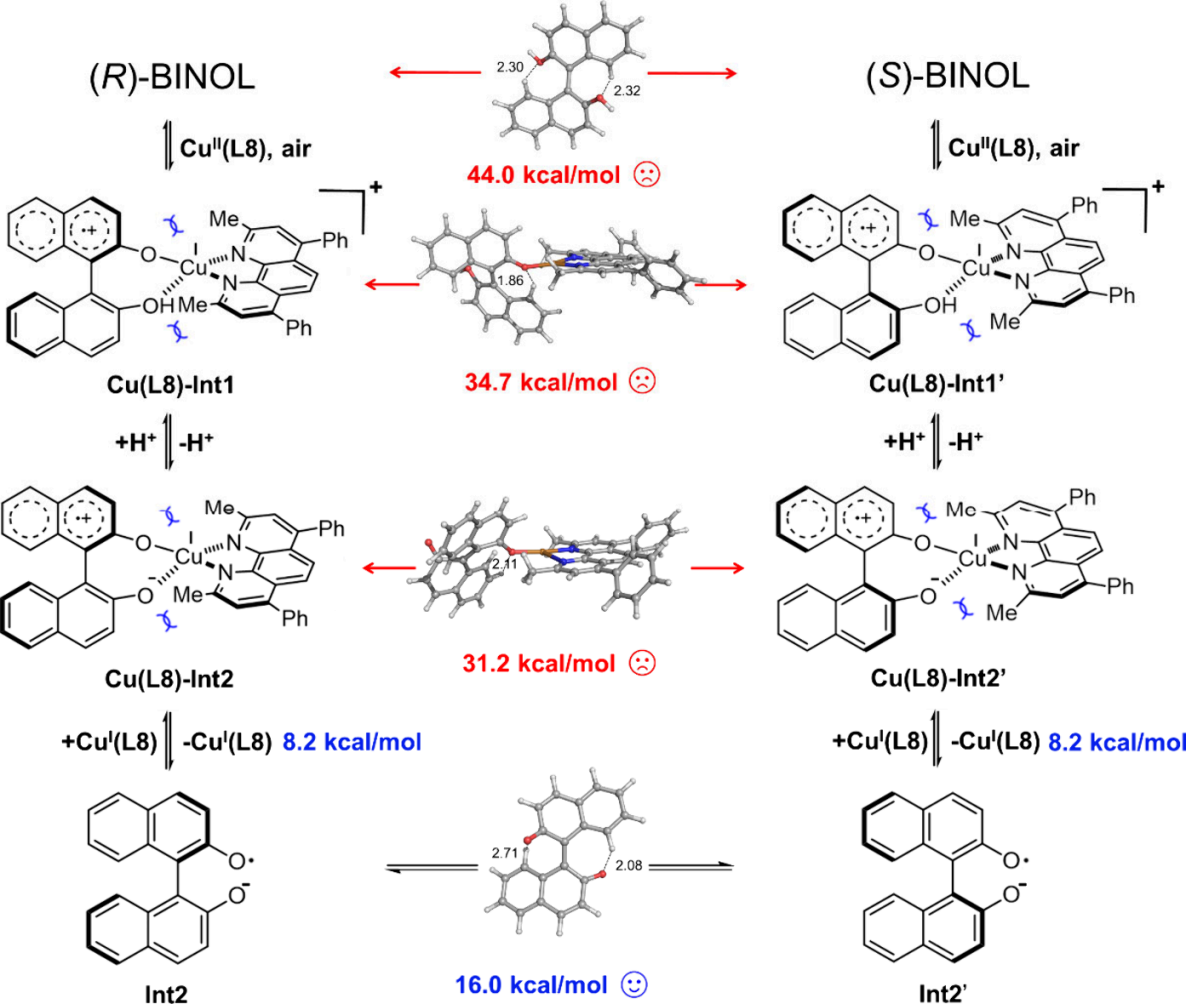
Proposed racemization mechanism.

Density functional theory (DFT) calculations provide support for the radical mechanism of the racemization of BINOL (Figure 8). The calculations showed that the direct rotation of atropisomeric BINOL has to overcome a high energy barrier of 44.0 kcal/mol. After the binuclear coordination of (*R*)- or (*S*)-BINOL to the Cu^II^ center in the presence of bidentate ligand **L8**, an intramolecular single-electron oxidation^22^ of the BINOL moiety by copper(II) leads to the formation of radical-cation copper(I) complex **Cu(L8)-Int1** and **Cu(L8)-Int1′**, respectively, which can be further deprotonated to the complexes **Cu(L8)-Int2** and **Cu(L8)-Int2′**. However, the barriers for the rotation of BINOL in these complexes are also very high, 34.7 and 31.2 kcal/mol for **Cu(L8)-Int1** and **Cu(L8)-Int2**, respectively. Analysis of the optimized structures of the transition states showed that this can be attributed to the steric hindrance between BINOL and ligand **L8**. We then considered the racemization independent of Cu(**L8**) with BINOL in different forms (for details, see Figure S12 in the Supporting Information). Interestingly, the rotation between **Int2** and **Int2′** independent of Cu(**L8**) becomes feasible with a calculated barrier of 16.0 kcal/mol (Figure 8), while all of the other states of BINOL are associated with high barriers. Considering that the dissociation of the doubly deprotonated radical-anion BINOL (**Int2**) from the coordination of catalyst has an energy penalty of 8.2 kcal/mol (for details, see Table S17 in the Supporting Information), the overall activation barrier for the racemization is thus 24.2 kcal/mol, in excellent agreement with the experimentally measured data (24.9 kcal/mol in Figure 6a).

To clarify the factors responsible for the lower barrier of racemization of **Int2**, we applied the activation strain model (ASM) to calculate the energy components (Δ*E*_strain_ and Δ*E*_int_) for **Int2** and also those of BINOL for comparison. Δ*E*_strain_ and Δ*E*_int_ for BINOL are calculated to be 27.8 and 14.8 kcal/mol, respectively, while Δ*E*_strain_ and Δ*E*_int_ for **Int2** are determined to be 6.4 and 9.6 kcal/mol, respectively. It is thus evident that the lower barrier for the racemization of **Int2** primarily arises from a significantly reduced strain energy. Moreover, an overlay of the structures of **Int2** and BINOL leads to the same conclusion, in which the naphthalene moiety in the transition state structure of BINOL is more distorted than that in **Int2** (for details, see Figure S13 in the Supporting Information). Furthermore, the analysis by using an interaction region indicator (IRI),^23^ which can highlight the weak interaction regions, reveals that the lower Δ*E*_strain_ of **Int2** can be attributed to the weaker repulsive interactions between the oxygen functional groups and the naphthalene moieties, as compared to BINOL without deprotonation and single-electron oxidation (for details, see Figure S14 in the Supporting Information).

Finally, to demonstrate the advantages of the current strategy employing a neutral ligand via *in situ* coordination, high-throughput screening (HTC) of neutral ligands (>50) for the racemization of methylated BINOL (*S*)-**6** was conveniently conducted to identify an efficient ligand (**L21**), and **6** was detected with 38% ee after the reaction (for details, see Table S14 in the Supporting Information). This ligand can also offer π* orbitals to accept d-electrons from the metal. Therefore, this strategy can accelerate the discovery of suitable racemization catalyst(s), thus facilitating DKR development.

## Conclusion

In conclusion, a novel strategy was proposed and applied to overcome the limitations of transition-metal catalysis in the chemoenzymatic DKR of atropisomeric bisnaphthols. Based on this strategy, we developed the chemoenzymatic DKR of BINOLs under compatible copper and enzyme catalysis. BCP-ligand **L8** was found as a crucial element not only to significantly accelerate the racemization process but also to offer π* orbitals allowing additional metal-to-ligand d−π* back-donation, enhancing the coordination effect between the ligand and metal. With this DKR approach, a variety of functionalized *C*_2_- and *C*_1_-symmetric BINOLs could be employed to access enantioenriched BINOL skeletons with as low as 1 mol % loading of CuCl and **L8**. Notably, the coupling-DKR cascade was successfully realized from 2-naphthols in one-pot transformations, exhibiting the advantage of utilizing unfixed ligands in copper catalysts. Mechanistic studies suggest that racemization of BINOL proceeds via a doubly deprotonated radical-anion intermediate, which allows axial rotation. Finally, because of the wide application of axially chiral skeletons in catalysts and ligands, this chemistry will be of great interest for synthetic chemists. Further studies of the mechanism and synthetic applications are currently ongoing in our laboratory.

## Methods

### Representative Procedure for Enzymatic DKR of Atropisomeric Biaryls by Copper Catalysis

Under an air atmosphere, to a tube charged with a stir bar were added CuCl (0.1 mg, 0.001 mmol), ligand **L8** (0.4 mg, 0.001 mmol), and 1 mL of toluene sequentially. The reaction tube was placed under sonication in a water bath for 2 min, and then the mixture was continued stirring at room temperature for an additional 10 min. *rac*-**1** (0.1 mmol), lipase LPL-311-Celite (2.1–3 w/w), Na_2_CO_3_ (15.9 mg, 0.15 mmol), and vinyl acetate (0.2 mL, 0.2 v/v) were sequentially added to the rection mixture. After that, the tube was sealed with a rubber septum and stirred at 50 °C for 36–60 h. After the reaction was complete, the mixture was filtered through filter paper by using a Buchner funnel. The residual Celite pad was washed with EtOAc. After removal of the solvent, the crude product was purified via flash column chromatography on silica gel (eluent: petroleum ether/ethyl ether) to afford (*R*)-monoacylated **2**.

## Data Availability

All data generated or analyzed during this study are included in this Article and the Supporting Information. Details about materials and methods, experimental procedures, mechanistic studies, characterization data, computational details, NMR and HPLC spectra are available in the Supporting Information. Calculated coordinates are available in the Supplementary Data file. All other data are available from the corresponding author upon request.

## Supporting Information Available

Experimental details and procedures, characterization, crystallographic and computation data, NMR and HPLC spectra (PDF)
CheckCIF file for Cu(**L8**)Cl_2_ (PDF)
X-ray crystallographic data for Cu(**L8**)Cl_2_ (CIF)
CheckCIF file for Cu(**L10**)Cl_2_ (PDF)
X-ray crystallographic data for Cu(**L10**)Cl_2_ (CIF)

## Supplementary Material

oc4c01370_si_002.pdf

oc4c01370_si_003.cif

oc4c01370_si_004.pdf

oc4c01370_si_005.cif
