# Supplementary material for: Overcoming the Limitations of Transition-Metal Catalysis in the Chemoenzymatic Dynamic Kinetic Resolution (DKR) of Atropisomeric Bisnaphthols
Source: ACS Cent Sci. 2024 Nov 5;10(11):2099–110. doi: 10.1021/acscentsci.4c01370 (PMC11613327; doi:10.1021/acscentsci.4c01370)
Supplement: Supplementary file 1 — oc4c01370_si_001.pdf [file oc4c01370_si_001.pdf]

Supporting Information

Overcoming the Limitations of Transition-Metal Catalysis in the Chemoenzymatic Dynamic Kinetic Resolution (DKR) of Atropisomeric Bisnaphthols

Kun Wang[1,†], Wei Wang[2,3,†], Dingkai Lou[1], Jie Zhang[1], Changli Chi[1], Jan-E. Bäckvall[4],* Xiang Sheng[2,3],* and Can Zhu[1]*

[1]Department of Chemistry, Fudan University, 2005 Songhu Road, Shanghai 200438, China.

[2]Tianjin Institute of Industrial Biotechnology, Chinese Academy of Sciences, Tianjin 300308, P.R. China.

[3]National Center of Technology Innovation for Synthetic Biology and Key Laboratory of Engineering Biology for Low-Carbon Manufacturing, Tianjin 300308, P.R. China.

[4]Department of Organic Chemistry, Arrhenius Laboratory, Stockholm University, SE-10691 Stockholm, Sweden

*Email: zhucan@fudan.edu.cn (Can Zhu); shengx@tib.cas.cn (Xiang Sheng); jan.e.backvall@su.se (Jan-E. Bäckvall)

[†]These authors contribute equally

Table of Contents

General information S1

General procedure for the preparation of starting materials S2

General procedure for Cu-complexes synthesis S3

Investigation of racemization catalysts S4

Investigation of biocatalysts for the KR process S5

Optimization of reaction conditions for the DKR process..S7

General procedure A for the DKR of C_2-symmetric BINOLs..................................S10

General procedure B for the DKR of C_1-symmetric BINOLs..................................S19

General procedure for the one-pot oxidative coupling-DKR cascades to access C_2-symmetric BINOLs from 2-naphthols S43

Mechanistic Studies S52

Comparison experiments S52

UV-Vis absorption spectroscopy S57

X-Ray Crystallography Data...S68

DFT Calculation...S72

References...S84

NMR spectra S86

General information

Unless otherwise noted, all reagents were used as received from the commercial suppliers. CuCl was obtained from aladdin and used without further treatment. Binaphthols (BINOLs) were prepared according to reported procedure. The immobilized *Burkholderia cepacia* lipase PS-IM was used as received from Amano Enzyme Inc. The lyophilized powder *Pseudomonas fluorescens* lipase AK, *Candida rugose* lipase AYS, *Candida antarctica* lipase CL was gifted from Amano Enzyme Inc. and immobilized on Celite® 545. The lyophilized powder *pocine pancreas* lipase PPL was supplied by Sigma-Aldrich and immobilized on Celite® 545. Novozyme 435 was used as received from novocata biotechnology co., Ltd. The immobilized Lipozyme RM-IM and Lipozyme TL-IM was used as received from Beijing Cliscent Technology Co., Ltd. The solvents of DCM and toluene were obtained from Solvent Purifier. Reactions were monitored using thin-layer chromatography (SiO_2). TLC plates were visualized with UV light (254 nm) or $KMnO_4$ stain. Reaction products were separated via flash chromatography using 300-400 mesh silica gel. NMR spectra were recorded at 400 MHz (^{1}H) and at 101 MHz (^{13}C) on a Bruker spectrometer, respectively. Chemical shifts (δ) are reported in ppm, using the residual solvent peak in $CDCl_3$ (H_{TMS} = 0.00 and C = 77.0 ppm) or in DMSO-d_6 (H = 2.50 and C = 39.52 ppm) as internal standard, and coupling constants (*J*) are given in Hz. HRMS were recorded using EI or ESI techniques. Enantiomeric excesses (*ee*) were determined by HPLC analysis on Agilent HPLC units.

General procedure for the preparation of starting materials

Starting material *rac*-**1a** is commercially available. *Rac*-**1b-x** were prepared according to reported procedures.[1-5]

Preparation of lipase LPL-311-Celite for DKR reaction

Lipase LPL-311 (*lipoprotein lipase from pseudomonas sp.*) from Toyobo Co., Ltd. (received as lyophilized powder, 300 mg) was dissolved in 0.05 M sodium hydrogen phosphate buffer (pH 7.0, 6 mL) at 0 °C. Celite® 545 (5.7 g) was added to the enzyme solution. The activity of LPL-311 lyophilized powder was 33.9 U/mg, therefore the theoretical activity of loaded lipase was calculated as 1.70 U/mg. The suspension was stirred at room temperature for 24 h, and then filtrated using a Buchner funnel. The obtained residue was continuously dried under vacuum conditions at room temperature for 36 h. According to the above producer, the lyophilized powder *Pseudomonas fluorescens* lipase AK, *Candida rugose* lipase AYS, *Candida antarctica* lipase CL, and *pocine pancreas* lipase PPL were immobilized on Celite® 545 respectively.

Preparation of lipase LPL-311-Celite for one-pot oxidative coupling-DKR cascade reaction

Lipase LPL-311 (*lipoprotein lipase from pseudomonas sp.*) from Toyobo Co., Ltd. (received as lyophilized powder, 1200 mg) was dissolved in 0.05 M sodium hydrogen phosphate buffer (pH 7.0, 10ml) at 0 °C. Celite® 545 (4.8 g) was added to the enzyme solution. The activity of LPL-311 lyophilized powder was 33.9 U/mg, therefore the theoretical activity of loaded lipase was calculated as was 6.9 U/mg. The suspension was stirred at room temperature for 24 h, and then filtrated using a Buchner funnel. The obtained residue was continuously dried under vacuum conditions at room temperature for 36 h.

General procedure for Cu-complexes synthesis

*Synthesis of Cu(**L8**)Cl2*

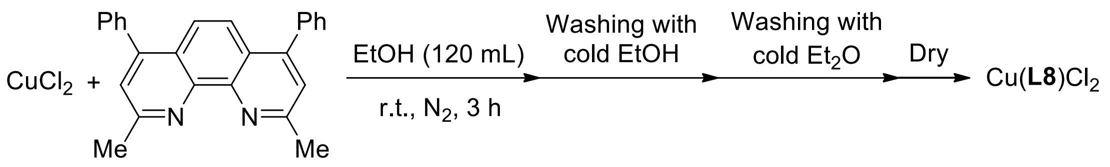

L8

Under N_2 atmosphere, to an overdried 2-neck round bottom flask charged with a stir bar were added $CuCl_2$ (268.9 mg, 2.0 mmol), ligand **L8** (720.9 mg, 2.0 mmol) and 120 mL of EtOH sequentially. The reaction flask was placed under sonication in a water bath for 10 min, and then let the mixture continue to stir at room temperature for additional 3 h. After that, the misture was filtrated and washed with cold EtOH (100 mL) and cold Et_2O (100 mL) in order. The residual was vacuum dried for 24 h afforded Cu(**L8**)Cl_2 (reddish solid, 774.0 mg, 78% yield). The structure of Cu(**L8**)Cl_2 can be seen in **X-Ray Crystallography Data** part.

*Synthesis of Cu(**L10**)Cl2*

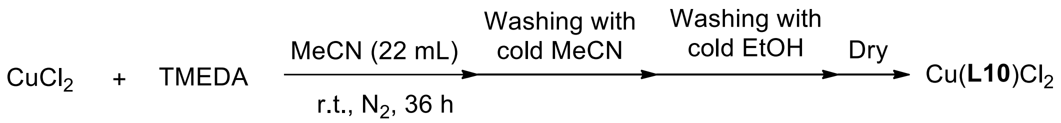

L10

Under N_2 atmosphere, to an overdried 2-neck round bottom flask charged with a stir bar were added $CuCl_2$ (672.3 mg, 5.0 mmol), ligand **L10** (750.0 μL, 5.0 mmol) and 22 mL of MeCN sequentially. The reaction flask was placed under sonication in a water bath for 10 min, and then let the mixture continue to stir at room temperature for additional 36 h. After that, the misture was filtrated and washed with cold MeCN (30 mL) and cold EtOH (30 mL) in order. The residual was vacuum dried for 24 h afforded Cu(**L10**)Cl_2 (blue solid, 688.7 mg, 55% yield). The structure of Cu(**L10**)Cl_2 can be seen in **X-Ray Crystallography Data** part. Cu(**L9**)Cl_2 can be synthesized using same method (green solid, 1.1 g, 72% yield).

Investigation of racemization catalysts

Table S1. Ligand effect in the racemization of (*R*)-BINOL[a]

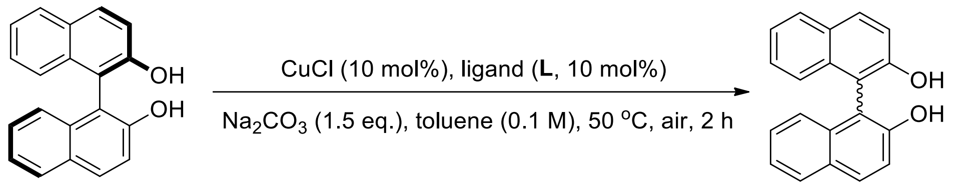

(*R*)-**1a**: >99% ee

1a

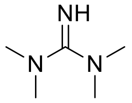

L1

1a: 99% *ee*

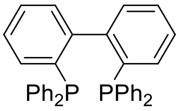

L2

1a: 99% *ee*

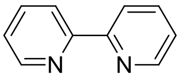

L3

1a: 99% *ee*

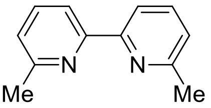

L4

1a: 98% *ee*

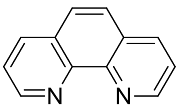

L5

1a: 99% *ee*

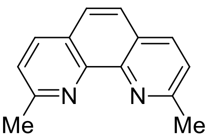

L6[b]

1a: 95% *ee*

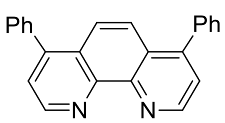

L7

1a: 96% *ee*

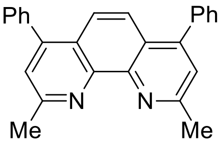

L8[c]

1a: 16% *ee*

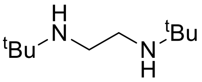

L9[d]

1a: 0% *ee*

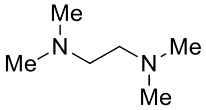

L10[e]

1a: 57% *ee*

[a]The reaction was carried out using (*R*)-**1a** (*c*=0.1 M), CuCl (10 mol%), ligand (10 mol%) and Na_2CO_3 (1.5 eq.) at 50 °C in toluene for 2 h under an air atmosphere. [b]The reaction was performed in mixed solvents (toluene:DCM = 7:3). [c]BINOL was recovered of 92% yield in 2 h by ^{1}H NMR analysis with CH_2Br_2 as the internal standard. [d]BINOL was recovered of 65% yield in 2 h by ^{1}H NMR analysis with CH_2Br_2 as the internal standard. [e]BINOL was recovered of 95% yield in 2 h by ^{1}H NMR analysis with CH_2Br_2 as the internal standard.

Investigation of biocatalysts for the KR process

Table S2. Screening of lipase[a]

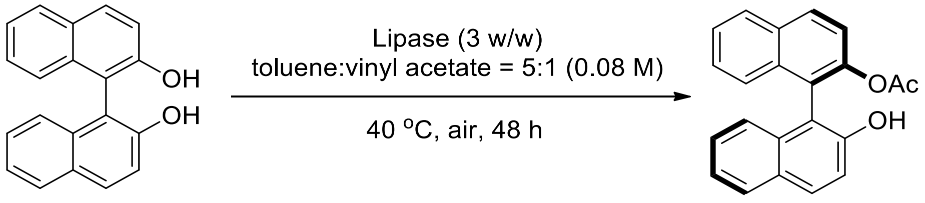

rac-**1a** (*R*)-**2a**

Entry	Lipase	Conversion (%)[b]	*ee* (%) of (*R*)-**2a**	E value[c]
1	PS-IM	42	89	26
2	AK-IM	<1	-	-
3	AYS-IM	<1	-	-
4	CL-IM	<1	-	-
5	PPL-IM	<1	-	-
6	Novozym 435	<1	-	-
**7**	**LPL-311-Celite**	**38**	**98**	**205**
8	Lipozyme TL-IM	14	86	16
9	Lipozyme RM	35	92	38

[a]The reaction was carried out using *rac*-**1a** (*c*=0.08 M), Lipase (3 w/w) at 40 °C in toluene/vinyl acetate (1.0 mL:0.2 mL) for 48 h under an air atmosphere. [b]Determined by ^{1}H NMR analysis with CH_2Br_2 as the internal standard. [c]For the kinetic resolution, E value is an index to show the enantioselectivity and can be calculated as follows:

$$E = \frac{\ln[(1-e.e.\ s)/(1+\frac{e.e.\ s}{e.e.\ p})]}{\ln[(1+e.e.\ s)/(1+\frac{e.e.\ s}{e.e.\ p})]}$$ (*e.e. s* is optical purity of recovered starting material; *e.e. p* is optical purity of product.)

Table S3. Optimization of temperature for the KR process[a]

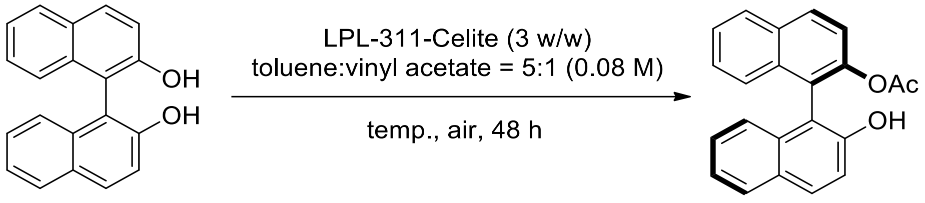

rac-**1a** (*R*)-**2a**

Entry	Temperature	Conversion (%)[b]	*ee* (%) of (*R*)-**2a**
1	40 ºC	38	98
**2**	**50 ºC**	**47**	**96**

[a]The reaction was carried out using *rac*-**1a** (*c*=0.08 M), Lipase LPL-311-Celite (3 w/w) at 40 ºC or 50 ºC in toluene/vinyl acetate (1.0 mL:0.2 mL) for 48 h under an air atmosphere. [b]Determined by ^1H NMR analysis with CH_2Br_2 as the internal standard.

Optimization of reaction conditions for the DKR process

Table S4. Preliminary attempts to combine copper racemization catalysis with KR system[a]

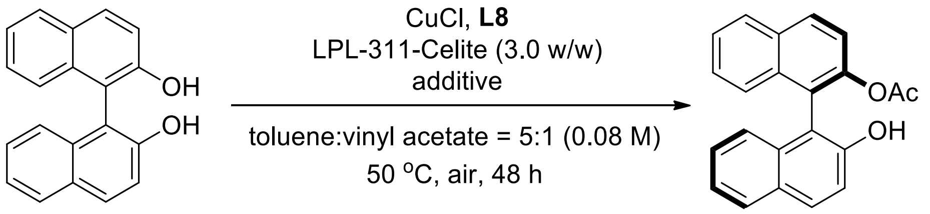

rac-**1a** (*R*)-**2a**

Entry	CuCl (mol%)	**L8** (mol%)	Additive	(*R*)-**2a** Yield (%)[b], *ee* (%)		Recovery Yield (%)[b], *ee* (%)	
1	5	5	Na_2CO_3 (1.5 eq.)	59	95	18	<5
2	2	2	Na_2CO_3 (1.5 eq.)	71	96	14	<5
**3**	**1**	**1**	**Na_2CO_3 (1.5 eq.)**	**81**	**96**	**18**	**<5**
4	1	1	$NaHCO_3$ (3.0 eq.)	52	94	48	70
5	1	1	CH_3COOH (1.5 eq.)	N.D.		86	0
6	1	1	none	21	97	79	11

[a]The reaction was carried out using *rac*-**1a** (*c*=0.08 M), CuCl, Ligand **L8**, LPL-311-Celite (3 w/w) and Na_2CO_3 (1.5 equiv) at 50 °C in toluene/vinyl acetate (1.0 mL:0.2 mL) for 48 h under an air atmosphere. [b]Determined by ^1H NMR analysis with CH_2Br_2 as the internal standard.

Equation S1. Acylation of BINOL *rac*-**1a** in the presence of Na_2CO_3

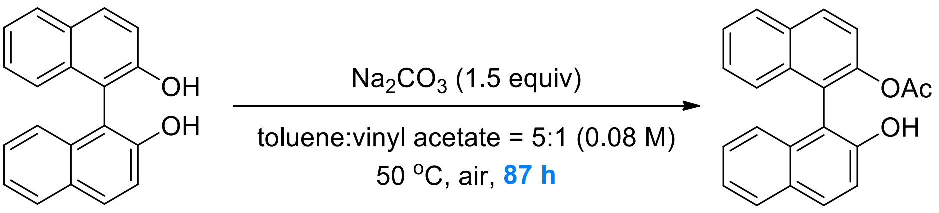

rac-**1a**

rac-**2a**
trace
No background reaction

Table S5. Screening of solvents[a]

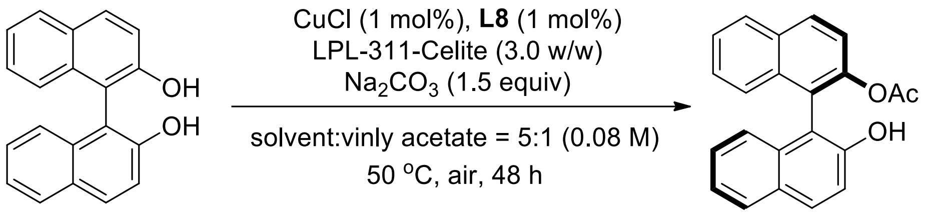

Entry	Solvents	(*R*)-**2a** Yield (%)[b]	*ee* (%)	Recovery Yield (%)[b]	*ee* (%)
1	Anisole	57	95	35	48
2	*tert*-Butyl methyl ether	49	96	51	67
3	*m*-Xylene	76	95	24	40
4	*p*-Xylene	87	92	10	10
5	*o*-Xylene	79	95	21	24
6	Benzotrifluoride	82	93	15	53
7	Hexane	57	70	43	88
8	2-Methyltetrahydrofuran	20	89	70	12
9	DME	21	40	71	4
10	Chlorobenzene	71	96	29	28
11	Toluene	81	96	18	<5
**12[c]**	**Toluene**	**85**	**96**	**<5**	**<5**
12	1,4-Dioxane	5	82	92	1
13	CPME	46	93	44	50
14	*i*-Pr_2O	72	84	28	90
15	DCM	17	40	70	1
16	THF	8	47	70	1

[a]The reaction was carried out using *rac*-**1a** (*c*=0.08 M), CuCl (1 mol%), Ligand **L8** (1 mol%), LPL-311-Celite (3 w/w) and Na_2CO_3 (1.5 equiv) at 50 °C in solvents/vinyl acetate (1.0 mL:0.2 mL) for 48 h under an air atmosphere. [b]etermined by ^1H NMR analysis with CH_2Br_2 as the internal standard. [c]The reaction time was 60 h.

Table S6. Screening of acyl donor[a]

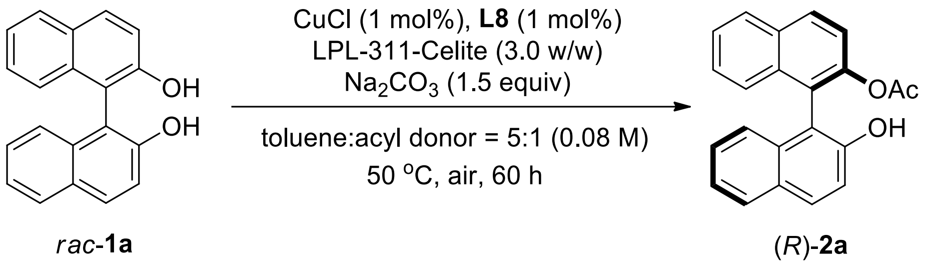

Entry	Acyl donor	(*R*)-**2a** Yield (%)[b]	(*R*)-**2a** *ee* (%)	Recovery Yield (%)[b]	Recovery *ee* (%)
1	isopropenyl acetate	64	95	33	27
**2**	**vinyl acetate**	**85**	**96**	**<5**	**<5**
3	vinyl butyrate	37	39	61	6
4	vinyl octanoate	20	57	73	10

[a]The reaction was carried out using *rac*-**1a** (c=0.08 M), CuCl (1 mol%), Ligand **L8** (1 mol%), LPL-311-Celite (3 w/w) and Na_2CO_3 (1.5 equiv) at 50 °C in toluene/acyl donor (1.0 mL:0.2 mL) for 60 h under an air atmosphere. [b]Determined by ^1H NMR analysis with CH_2Br_2 as the internal standard.

Finnally, CuCl (1 mol%), ligand **L8** (1 mol%), LPL-311-Celite (3.0 w/w), Na_2CO_3 (1.5 equiv) and BINOLs (1.0 equiv) in mixed solvents (toluene:vinyl acetate = 1.0 mL: 0.2 mL) at 50 °C for 60 h were defined as the optimal reaction conditions.

General procedure A for the DKR of C_2-symmetric BINOLs

*(R)-2'-Hydroxy-[1,1'-binaphthalen]-2-yl acetate (R)-**2a***

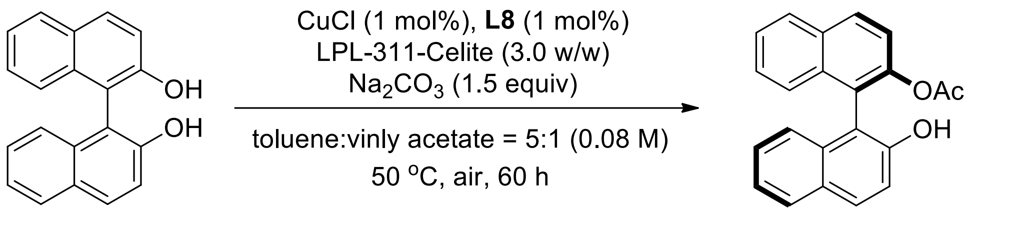

Under an air atmosphere, to a 10 mL tube charged with a stir bar were added CuCl (0.1 mg, 0.001 mmol), ligand **L8** (0.4 mg, 0.001 mmol) and 1 mL of toluene sequentially. The reaction tube was placed under sonication in a water bath for 2 min, and then let the mixture continue to stir at room temperature for additional 10 min. *Rac*-**1a** (28.6 mg, 0.1 mmol), lipase LPL-311-Celite (85.8 mg, 3 w/w), Na_2CO_3 (15.9 mg, 0.15 mmol) and vinyl acetate (0.2 mL, 0.2 v/v) were sequentially added to the rection mixture. After that, the tube was sealed with a rubber septum and stirred at 50 °C for 60 h. After the reaction was complete, the mixture was filtrated through filter paper by using a buchner funnel. The residual Celite pad was washed with EtOAc. After removal of the solvent, the crude product was purified via flash column chromatography on silica gel (eluent: petroleum ether/ethyl ether = 10/1) afforded (*R*)-**2a** (26.8 mg, 82% yield, 96% *ee*): colorless oil. $[\alpha]^{24}_{D}$ +109.4 (*c* = 0.94, $CHCl_3$); **^{1}H NMR** (400 MHz, Chloroform-*d*) δ 8.06 (d, *J* = 8.9 Hz, 1H), 7.96 (d, *J* = 8.2 Hz, 1H), 7.90 (d, *J* = 8.9 Hz, 1H), 7.84 (d, *J* = 8.1 Hz, 1H), 7.49 (t, *J* = 7.4 Hz, 1H), 7.39 (d, *J* = 8.9 Hz, 1H), 7.35-7.30 (m, *J* = 9.3, 4.8 Hz, 3H), 7.25-7.22 (m, *J* = 7.8 Hz, 2H), 7.03 (d, *J* = 8.4 Hz, 1H), 5.25 (s, 1H), 1.85 (s, 3H); **^{13}C NMR** (101 MHz, Chloroform-*d*) δ 170.40, 151.71, 147.99, 133.47, 133.42, 132.20, 130.77, 130.36, 128.94, 128.25, 127.97, 127.43, 126.68, 126.28, 125.72, 124.51, 123.51, 123.09, 121.73, 118.20, 113.94, 20.37. **HRMS (EI)** *m/z* calcd for $C_{22}H_{16}O_3$ [M^+]: 328.1094, found: 328.1093. The optical purity of (*R*)-**2a** was determined by HPLC analysis on a CHIRALPAK AD-H column, 25 °C, wavelength = 250 nm, *n*-Hexane/*i*-PrOH = 90:10, flow rate = 1.0 mL/min, t_R = 12.67

min for major isomer, t_S = 10.97 min for minor isomer.

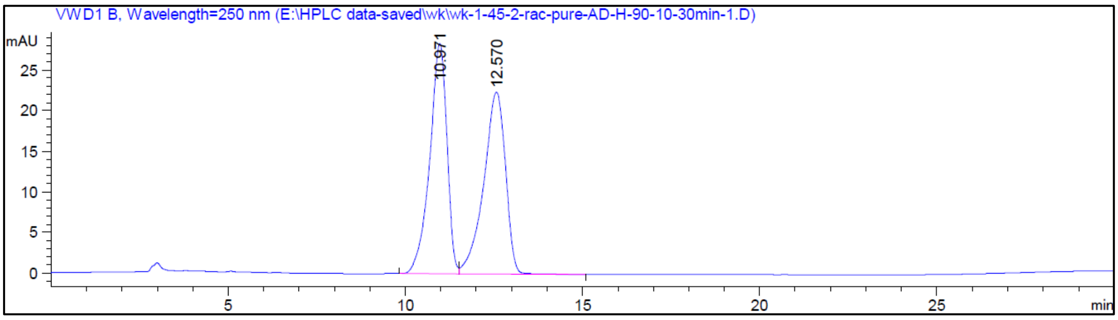

Peak #	RetTime [min]	Type	Width [min]	Area [mAU*s]	Height [mAU]	Area %
1	10.971	BV	0.5123	958.13873	28.34891	50.0142
2	12.570	VB	0.6486	957.59406	22.40618	49.9858

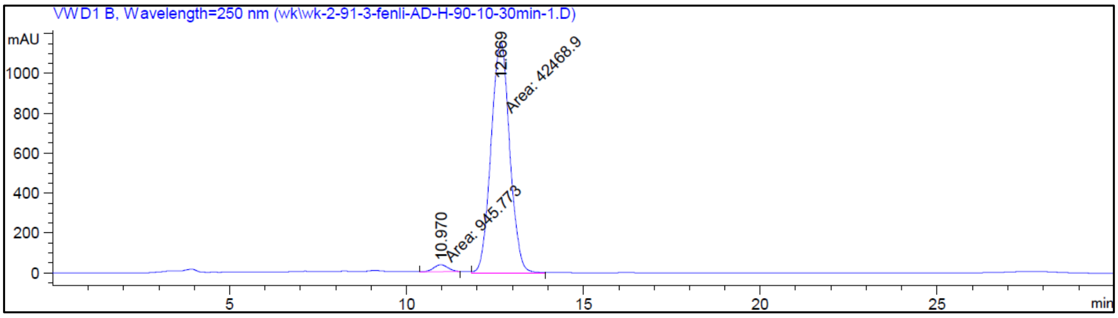

Peak #	RetTime [min]	Type	Width [min]	Area [mAU*s]	Height [mAU]	Area %
1	10.970	MM	0.4587	945.77313	34.36636	2.1785
2	12.669	MM	0.6125	4.24689e4	1155.62878	97.8215

*(R)-2'-Hydroxy-6,6'-dimethyl-[1,1'-binaphthalen]-2-yl acetate (R)-**2b***

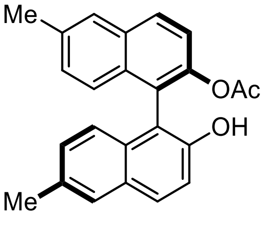

(*R*)-**2b**

According to *gerneral procedure A*, the reaction of *rac*-**1b** (0.1 mmol) under the catalysis of CuCl (0.001 mmol), ligand **L8** (0.001 mmol), and LPL-311-Celite (3.0 w/w) in the presence of Na_2CO_3 (0.15 mmol) at 50 ºC in toluene/vinyl acetate (1.0 mL:0.2 mL) for 60 h under an air atmosphere afforded (*R*)-**2b** (32.3 mg, 91% yield, 96% *ee*):

colorless oil. $[\alpha]^{24}_{D}$ +7.2 (c = 1.16, $CHCl_3$); **^{1}H NMR** (400 MHz, Chloroform-d) δ 7.95 (d, J = 8.9 Hz, 1H), 7.80 (d, J = 8.9 Hz, 1H), 7.72 (s, 1H), 7.61 (s, 1H), 7.34 (d, J = 8.9 Hz, 1H), 7.28 (d, J = 8.9 Hz, 1H), 7.15 (s, 2H), 7.07 (dd, J = 8.6, 1.3 Hz, 1H), 6.93 (d, J = 8.6 Hz, 1H), 5.15 (s, 1H), 2.48 (s, 3H), 2.44 (s, 3H), 1.85 (s, 3H); **^{13}C NMR** (101 MHz, Chloroform-d) δ 170.46, 151.04, 147.29, 136.03, 132.92, 132.44, 131.69, 131.61, 129.97, 129.66, 129.57, 129.15, 128.86, 127.25, 127.03, 125.60, 124.46, 123.02, 121.69, 118.17, 114.05, 21.46, 21.28, 20.38. **HRMS (EI)** m/z calcd for $C_{24}H_{20}O_3$ $[M^+]$: 356.1407, found: 356.1406. **HPLC conditions**: CHIRALPAK IC column, 25 °C, wavelength = 250 nm, n-Hexane/i-PrOH = 95:5, flow rate = 1.0 mL/min, t_R = 16.20 min for major isomer, t_S = 25.98 min for minor isomer.

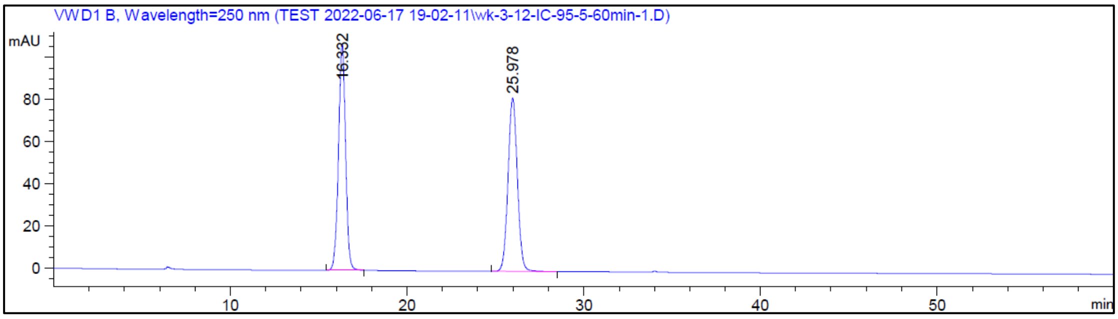

Peak #	RetTime [min]	Type	Width [min]	Area [mAU*s]	Height [mAU]	Area %
1	16.332	BB	0.4268	2990.43579	107.39996	49.7042
2	25.978	BB	0.5694	3026.03394	82.21667	50.2958

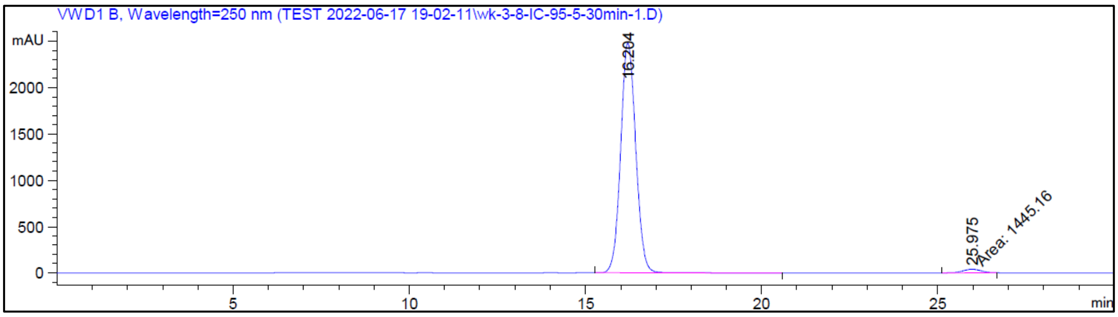

Peak #	RetTime [min]	Type	Width [min]	Area [mAU*s]	Height [mAU]	Area %
1	16.204	BV R	0.4609	7.39620e4	2481.26660	98.0835
2	25.975	MM	0.6109	1445.16016	39.42890	1.9165

*(R)-6,6'-Diethyl-2'-hydroxy-[1,1'-binaphthalen]-2-yl acetate (R)-**2c***

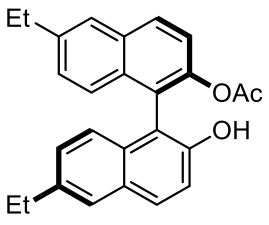

(*R*)-**2c**

According to *gerneral procedure A*, the reaction of *rac*-**1c** (0.1 mmol) under the catalysis of CuCl (0.001 mmol), ligand **L8** (0.001 mmol), and LPL-311-Celite (3.0 w/w) in the presenece of Na_2CO_3 (0.15 mmol) at 50 °C in toluene/vinyl acetate (1.0 mL:0.2 mL) for 60 h under an air atmosphere afforded (*R*)-**2c** (37.8 mg, 98% yield, 94% *ee*): colorless oil. $[\alpha]^{23}_{D}$ +3.5 (c = 1.44, $CHCl_3$); **^{1}H NMR** (400 MHz, Chloroform-*d*) δ 7.98 (d, 1H), 7.82 (d, J = 8.9 Hz, 1H), 7.73 (s, 1H), 7.63 (s, 1H), 7.34 (d, J = 8.8 Hz, 1H), 7.29 (d, J = 8.9 Hz, 1H), 7.21-7.15 (m, 2H), 7.11 (dd, J = 8.6, 1.6 Hz, 1H), 6.96 (d, J = 8.6 Hz, 1H), 5.19 (s, 1H), 2.76 (dq, J = 15.1, 7.6 Hz, 4H), 1.86 (s, 3H), 1.29 (td, J = 7.6, 1.9 Hz, 6H); **^{13}C NMR** (101 MHz, Chloroform-*d*) δ 170.53, 151.08, 147.31, 142.27, 139.19, 132.46, 131.90, 131.84, 130.14, 129.73, 129.14, 128.64, 127.82, 125.93, 125.72, 125.66, 124.51, 123.05, 121.62, 118.12, 114.05, 28.78, 28.61, 20.40, 15.34. **HRMS (EI)** *m/z* calcd for $C_{26}H_{24}O_3$ [M^+]: 384.1720, found: 384.1721. **HPLC conditions**: CHIRALPAK AD-H column, 25 °C, wavelength = 250 nm, *n*-Hexane/*i*-PrOH = 90:10, flow rate = 1.0 mL/min, t_R = 11.31 min for major isomer, t_S = 13.53 min for minor isomer.

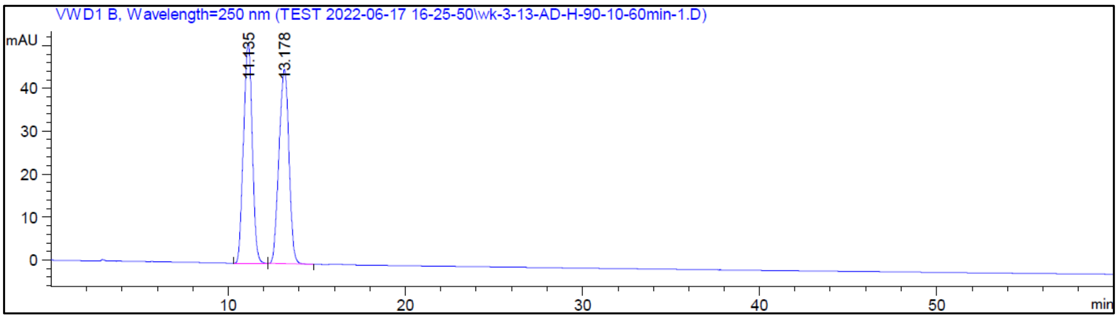

Peak #	RetTime [min]	Type	Width [min]	Area [mAU*s]	Height [mAU]	Area %
1	11.135	BB	0.5445	1761.48389	51.30949	49.7229
2	13.178	BB	0.6320	1781.11755	45.37415	50.2771

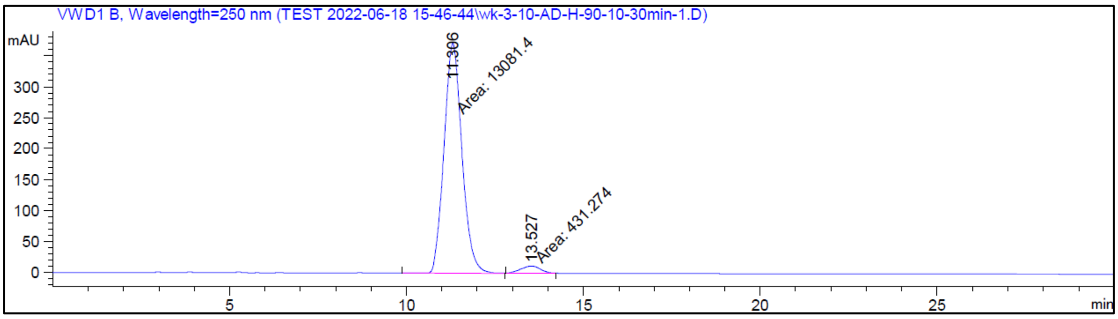

Peak #	RetTime [min]	Type	Width [min]	Area [mAU*s]	Height [mAU]	Area %
1	11.306	MM	0.5869	1.30814e4	371.47668	96.8084
2	13.527	MM	0.6276	431.27426	11.45302	3.1916

*(R)-6,6'-Dibromo-2'-hydroxy-[1,1'-binaphthalen]-2-yl acetate (R)-**2d***

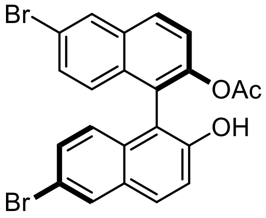

(*R*)-**2d**

According to *gerneral procedure A*, the reaction of *rac*-**1d** (0.1 mmol) under the catalysis of CuCl (0.001 mmol), ligand **L8** (0.001 mmol), and LPL-311-Celite (2.25 w/w) in the presenece of Na_2CO_3 (0.15 mmol) at 50 ºC in toluene/vinyl acetate (1.0 mL:0.2 mL) for 60 h under an air atmosphere afforded (*R*)-**2d** (43.4 mg, 89% yield, 95% *ee*): colorless oil. $[\alpha]^{23}_{D}$ −41.2 (*c* = 1.39, $CHCl_3$); ^{1}**H NMR** (400 MHz, Chloroform-*d*) δ 8.12 (d, *J* = 2.0 Hz, 1H), 8.00 (d, *J* = 2.1 Hz, 1H), 7.96 (d, *J* = 8.9 Hz, 1H), 7.80 (d, *J* = 9.0 Hz, 1H), 7.40 (dd, *J* = 8.9, 1.7 Hz, 2H), 7.34-7.28 (m, 2H), 7.06 (d, *J* = 9.0 Hz, 1H), 6.85 (d, *J* = 9.0 Hz, 1H), 5.28 (s, 1H), 1.87 (s, 3H); ^{13}**C NMR** (101 MHz, Chloroform-*d*) δ 170.13, 152.05, 148.18, 133.27, 131.89, 131.82, 130.91, 130.32, 130.09, 130.02, 129.98, 129.67, 127.32, 126.16, 122.99, 120.63, 119.45, 117.48, 113.71, 20.32. **HRMS (EI)** *m/z* calcd for $C_{22}H_{14}{}^{79}Br^{81}BrO_3$ [M^+]: 485.9285, found: 485.9288.

HPLC conditions: CHIRALPAK AD-H column, 25 ºC, wavelength = 250 nm, *n*-Hexane/*i*-PrOH = 90:10, flow rate = 1.0 mL/min, t_R = 15.78 min for major isomer, t_S = 13.02 min for minor isomer.

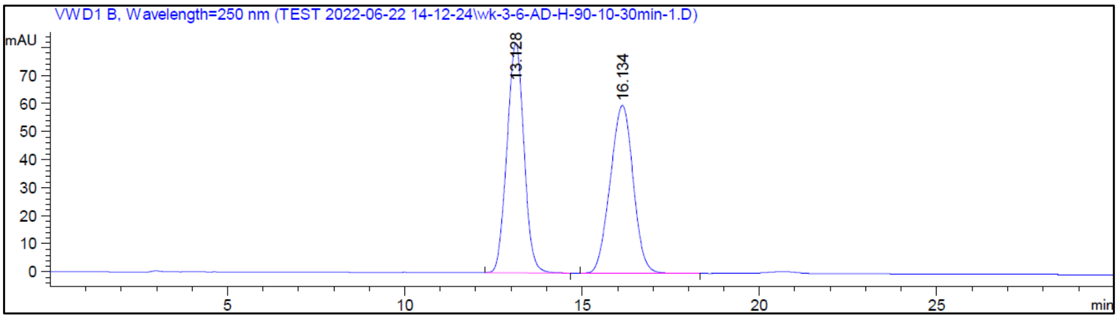

Peak #	RetTime [min]	Type	Width [min]	Area [mAU*s]	Height [mAU]	Area %
1	13.128	BB	0.5190	2726.20654	81.75751	50.3074
2	16.134	BB	0.6985	2692.88696	59.89481	49.6926

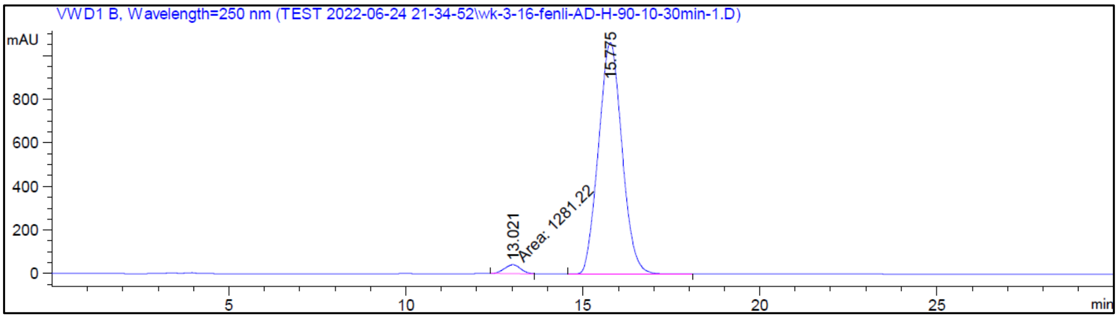

Peak #	RetTime [min]	Type	Width [min]	Area [mAU*s]	Height [mAU]	Area %
1	13.021	MM	0.5258	1281.21936	40.61238	2.5496
2	15.775	BB	0.7106	4.89703e4	1060.83948	97.4504

*(R)-2'-Hydroxy-6,6'-dimethoxy-[1,1'-binaphthalen]-2-yl acetate (R)-**2e***

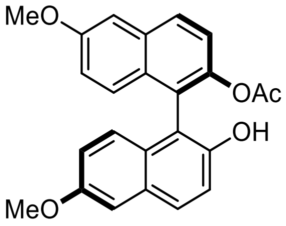

(*R*)-**2e**

According to *gerneral procedure A*, the reaction of *rac*-**1e** (0.1 mmol) under the catalysis of CuCl (0.001 mmol), ligand **L8** (0.001 mmol), and LPL-311-Celite (3.0 w/w) in the presenece of Na_2CO_3 (0.15 mmol) at 50 ºC in toluene/vinyl acetate (1.0 mL:0.2

mL) for 60 h under an air atmosphere afforded (*R*)-**2e** (38.1 mg, 98% yield, 91% *ee*): colorless oil. $[\alpha]^{23}_{D}$ +27.5 (*c* = 1.50, $CHCl_3$); **^{1}H NMR** (400 MHz, Chloroform-*d*) δ 7.91 (d, *J* = 8.9 Hz, 1H), 7.77 (d, *J* = 8.9 Hz, 1H), 7.33 (d, *J* = 8.9 Hz, 1H), 7.29 (d, *J* = 8.9 Hz, 1H), 7.23 (d, *J* = 3.4 Hz, 1H), 7.18-7.12 (m, 2H), 6.99 (dd, *J* = 9.2, 2.6 Hz, 1H), 6.97-6.90 (m, 2H), 5.13 (s, 1H), 3.89 (d, *J* = 7.6 Hz, 6H), 1.85 (s, 3H); **^{13}C NMR** (101 MHz, Chloroform-*d*) δ 170.54, 157.90, 155.95, 150.04, 146.12, 133.47, 129.81, 129.27, 128.92, 128.67, 128.63, 127.28, 126.08, 123.25, 122.12, 120.00, 119.05, 118.63, 114.47, 106.35, 55.34, 55.23, 20.36. **HRMS (EI)** *m/z* calcd for $C_{24}H_{20}O_5$ [M$^+$]: 388.1305, found: 388.1305. **HPLC conditions**: CHIRALPAK AD-H column, 25 °C, wavelength = 200 nm, *n*-Hexane/*i*-PrOH = 90:10, flow rate = 1.0 mL/min, t_R = 26.88 min for major isomer, t_S = 34.24 min for minor isomer.

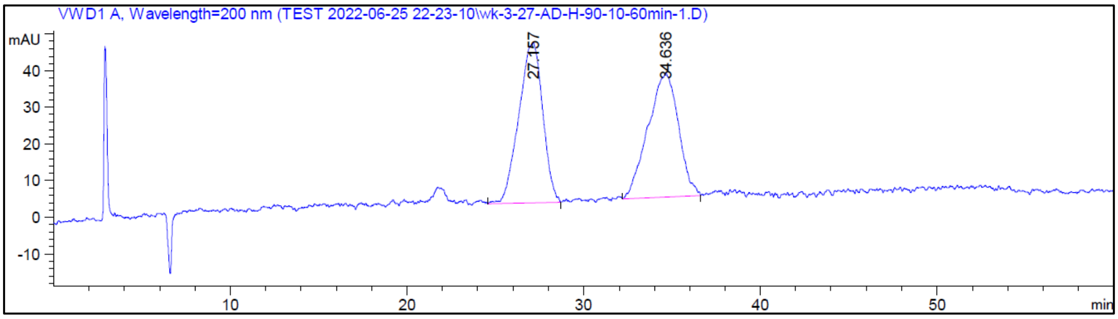

Peak #	RetTime [min]	Type	Width [min]	Area [mAU*s]	Height [mAU]	Area %
1	27.157	VV R	1.1286	4107.26855	43.61755	50.3349
2	34.636	VV R	1.4376	4052.60840	33.87751	49.6651

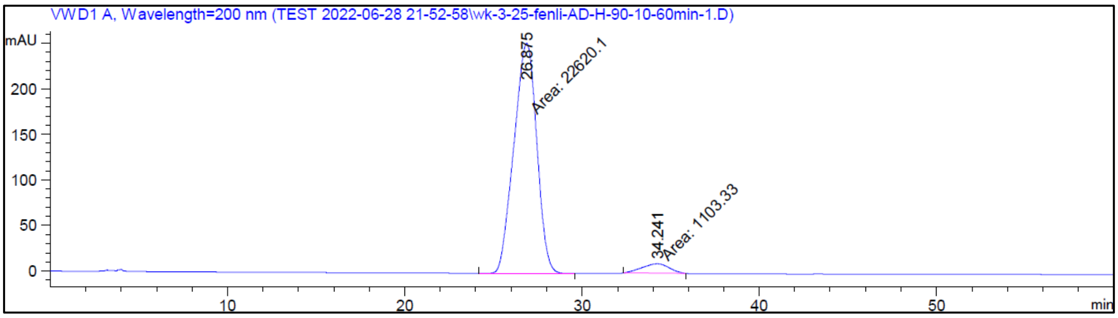

Peak #	RetTime [min]	Type	Width [min]	Area [mAU*s]	Height [mAU]	Area %
1	26.875	MM	1.4933	2.26201e4	252.46413	95.3492
2	34.241	MM	1.7809	1103.33191	10.32556	4.6508

*(R)-7,7'-Dibromo-2'-hydroxy-[1,1'-binaphthalen]-2-yl acetate (R)-**2f***

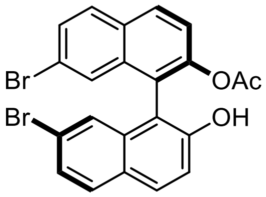

(*R*)-**2f**

According to *gerneral procedure A*, the reaction of *rac*-**1f** (0.1 mmol) under the catalysis of CuCl (0.005 mmol), ligand **L8** (0.005 mmol), and LPL-311-Celite (2.25 w/w) in the presenece of Na_2CO_3 (0.15 mmol) at 50 °C in toluene/vinyl acetate (1.0 mL:0.2 mL) for 60 h under an oxygen atmosphere (balloon, 1 atm) afforded (*R*)-**2f** (32.6 mg, 67% yield, 84% *ee*): colorless oil. $[\alpha]^{23}_{D}$ −85.9 (*c* = 1.13, $CHCl_3$); **^{1}H NMR** (400 MHz, Chloroform-*d*) δ 8.02 (d, *J* = 8.9 Hz, 1H), 7.86 (d, *J* = 8.9 Hz, 1H), 7.82 (d, *J* = 8.7 Hz, 1H), 7.71 (d, *J* = 8.7 Hz, 1H), 7.58 (dd, *J* = 8.7, 1.9 Hz, 1H), 7.43-7.33 (m, 3H), 7.31 (dd, *J* = 8.9, 2.2 Hz, 1H), 7.14 (s, 1H), 5.34 (s, 1H), 1.85 (s, 3H); **^{13}C NMR** (101 MHz, Chloroform-*d*) δ 170.23, 152.59, 148.83, 134.54, 134.51, 131.03, 130.70, 130.65, 129.98, 129.76, 127.43, 127.41, 127.14, 126.22, 122.34, 122.24, 121.92, 121.52, 118.84, 112.68, 20.30. **HRMS (EI)** *m/z* calcd for $C_{22}H_{14}{}^{79}Br^{81}BrO_3$ [M^+]: 485.9285, found: 485.9281. **HPLC conditions**: CHIRALPAK OD-H column, 25 °C, wavelength = 250 nm, *n*-Hexane/*i*-PrOH = 90:10, flow rate = 1.0 mL/min, t_R = 9.06 min for major isomer, t_S = 11.32 min for minor isomer.

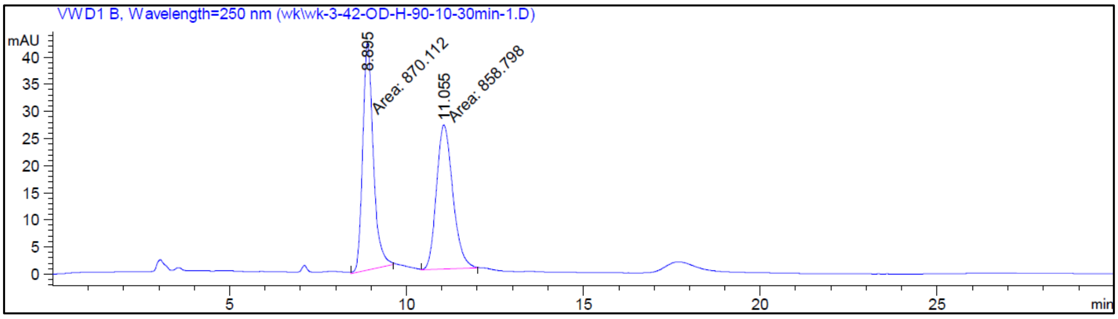

Peak #	RetTime [min]	Type	Width [min]	Area [mAU*s]	Height [mAU]	Area %
1	8.895	MM	0.3466	870.11224	41.84433	50.3272
2	11.055	MM	0.5382	858.79755	26.59267	49.6728

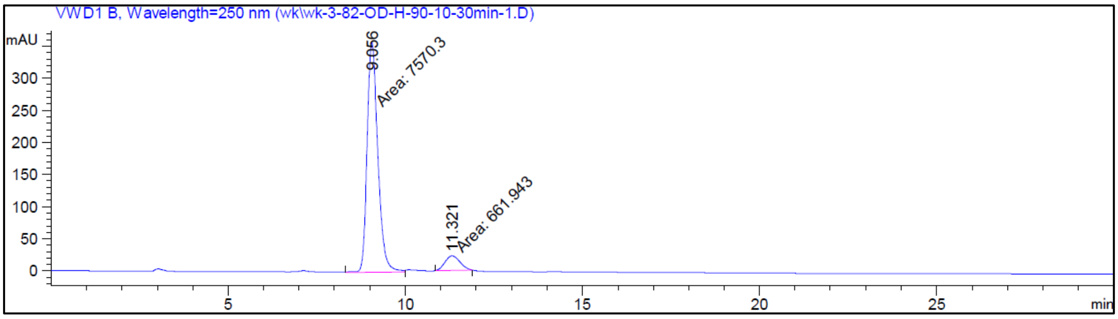

Peak #	RetTime [min]	Type	Width [min]	Area [mAU*s]	Height [mAU]	Area %
1	9.056	MM	0.3520	7570.29736	358.39511	91.9591
2	11.321	MM	0.4848	661.94257	22.75489	8.0409

*(R)-2'-Hydroxy-7,7'-dimethoxy-[1,1'-binaphthalen]-2-yl acetate (R)-**2g***

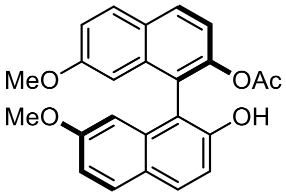

(*R*)-**2g**

According to *gerneral procedure A*, the reaction of *rac*-**1g** (0.1 mmol) under the catalysis of CuCl (0.005 mmol), ligand **L8** (0.005 mmol), and LPL-311-Celite (2.6 w/w) in the presenece of Na_2CO_3 (0.15 mmol) at 50 °C in toluene/vinyl acetate (1.0 mL:0.2 mL) for 60 h under an oxygen atmosphere (balloon, 1 atm) afforded (*R*)-**2g** (22.2 mg, 64% yield, 90% *ee*): colorless oil. $[\alpha]^{23}_{D}$ −42.8 (*c* = 1.33, $CHCl_3$); **^{1}H NMR** (400 MHz, Chloroform-*d*) δ 7.93 (d, *J* = 8.8 Hz, 1H), 7.82 (d, *J* = 8.9 Hz, 1H), 7.78 (d, *J* = 8.8 Hz, 1H), 7.72 (d, *J* = 8.9 Hz, 1H), 7.22 (d, *J* = 5.2 Hz, 1H), 7.18-7.12 (m, 2H), 6.97 (dd, *J* = 8.9, 2.5 Hz, 1H), 6.57 (d, *J* = 2.6 Hz, 1H), 6.38 (d, *J* = 2.5 Hz, 1H), 5.18 (s, 1H), 3.53 (s, 3H), 3.52 (s, 3H), 1.81 (s, 3H); **^{13}C NMR** (101 MHz, Chloroform-*d*) δ 170.18, 158.87, 158.33, 152.15, 148.64, 134.81, 134.69, 130.30, 130.01, 129.80, 129.46, 127.61, 124.34, 121.75, 119.28, 118.70, 115.51, 115.45, 113.26, 104.06, 103.60, 55.04, 54.96, 20.38. **HRMS (EI)** *m/z* calcd for $C_{24}H_{20}O_5$ [M$^+$]: 388.1305, found: 388.1305. **HPLC conditions**: CHIRALPAK IC column, 25 °C, wavelength = 250 nm, *n*-Hexane/*i*-PrOH = 95:5, flow rate = 0.5 mL/min, t_R = 17.80 min for major isomer, t_S =

26.25 min for minor isomer.

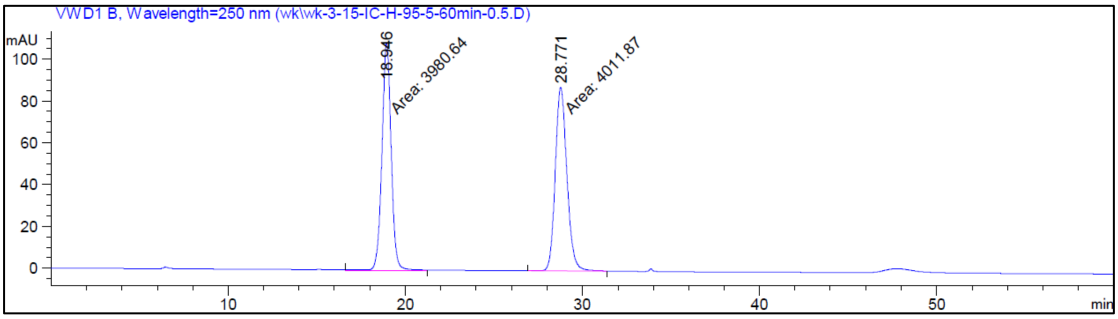

Peak #	RetTime [min]	Type	Width [min]	Area [mAU*s]	Height [mAU]	Area %
1	18.946	MM	0.6095	3980.63550	108.84559	49.8046
2	28.771	MM	0.7621	4011.87061	87.73353	50.1954

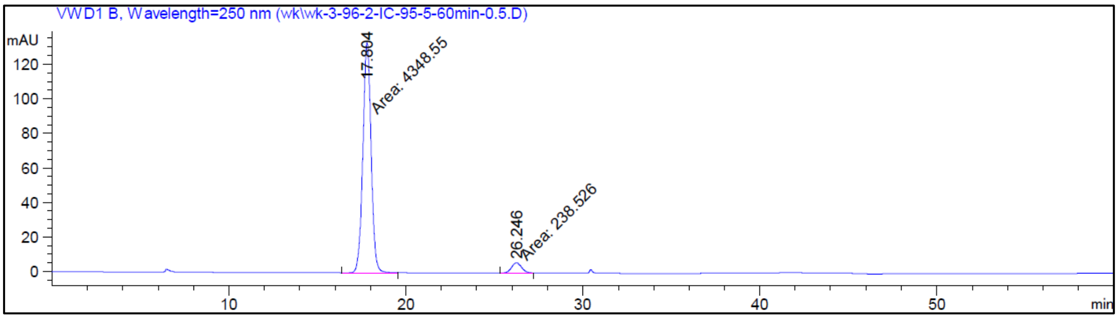

Peak #	RetTime [min]	Type	Width [min]	Area [mAU*s]	Height [mAU]	Area %
1	17.804	MM	0.5441	4348.54932	133.19612	94.8001
2	26.246	MM	0.6657	238.52559	5.97170	5.1999

General procedure B for the DKR of C_1-symmetric BINOLs

*(R)-6-Bromo-[1,1'-binaphthalene]-2,2'-diol (R)-**1i***

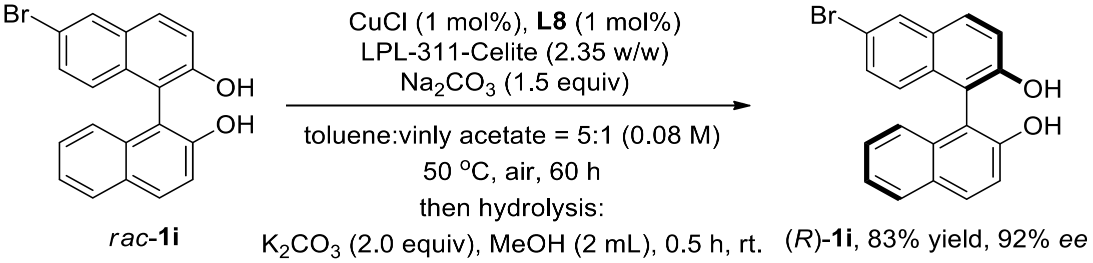

Under an air atmosphere, to a 10 mL tube charged with a stir bar were added CuCl

(0.1 mg, 0.001 mmol), ligand **L8** (0.4 mg, 0.001 mmol) and 1 mL of toluene sequentially. The reaction tube was placed under sonication in a water bath for 2 min, and then let the mixture continue to stir at room temperature for additional 10 min. *Rac*-**1i** (36.5 mg, 0.1 mmol), lipase LPL-311-Celite (85.8 mg, 2.35w/w), Na_2CO_3 (15.9 mg, 0.15 mmol) and vinyl acetate (0.2 mL, 0.2 v/v) were sequentially added to the rection mixture. After that, the tube was sealed with a rubber septum and stirred at 50 °C for 60 h. After the reaction was complete, the mixture was filtrated through filter paper by using a buchner funnel. The residual Celite pad was washed with EtOAc. After removal of the solvent, the crude product was purified via flash column chromatography on silica gel (eluent: petroleum ether/ethyl ether = 10/1) afforded (*R*)-mono-acylated **2i** as white soild. To a stirred solution of (*R*)-mono-acylated **2i** in MeOH (2.0 mL) was added K_2CO_3 (27.6 mg, 0.2 mmol). After being stirred for 0.5 h at room temperature, the reaction mixture was acidified by 0.5 mL 1 M HCl and transferred to a 25 mL separatory funnel. The mixture was extracted with 3×10 mL CH_2Cl_2. The combined organic layer was washed with brine and dried over anhydrous Na_2SO_4. After then the mixture was filtrated and concentrated in vacuo to afford (*R*)-**1i** (30.3 mg, 83% yield, 92% *ee*): white solid. MP: 182−184 °C; $[\alpha]^{24}_{D}$ −58.7 (*c* = 0.54, $CHCl_3$); **^{1}H NMR** (400 MHz, Chloroform-*d*) δ 8.00 (d, *J* = 2.1 Hz, 1H), 7.93 (d, *J* = 9.0 Hz, 1H), 7.85 (d, *J* = 8.0 Hz, 1H), 7.82 (d, *J* = 9.0 Hz, 1H), 7.38-7.26 (m, 5H), 7.06 (d, *J* = 8.4 Hz, 1H), 6.98 (d, *J* = 9.0 Hz, 1H), 5.08 (d, *J* = 33.6 Hz, 2H); **^{13}C NMR** (101 MHz, Chloroform-*d*) δ 152.95, 152.67, 133.28, 132.02, 131.60, 130.65, 130.52, 130.32, 129.41, 128.43, 127.61, 126.09, 124.15, 123.98, 118.90, 117.83, 117.76, 111.35, 110.28. **HRMS (EI)** *m/z* calcd for $C_{20}H_{13}{}^{81}BrO_2$ [M^+]: 366.0075, found: 366.0070. The optical purity of (*R*)-**1i** was determined by HPLC analysis on a CHIRALPAK OD-H column, 25 °C, wavelength = 250 nm, *n*-Hexane/*i*-PrOH = 90:10, flow rate = 1.0 mL/min, t_R = 26.43 min for major isomer, t_S = 16.92 min for minor isomer.

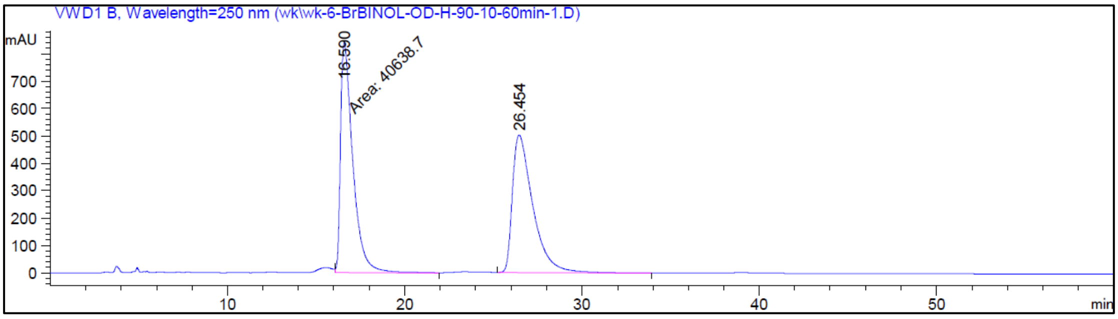

Peak #	RetTime [min]	Type	Width [min]	Area [mAU*s]	Height [mAU]	Area %
1	16.590	FM	0.8053	4.06387e4	841.10388	50.2680
2	26.454	BB	1.1937	4.02053e4	502.35938	49.7320

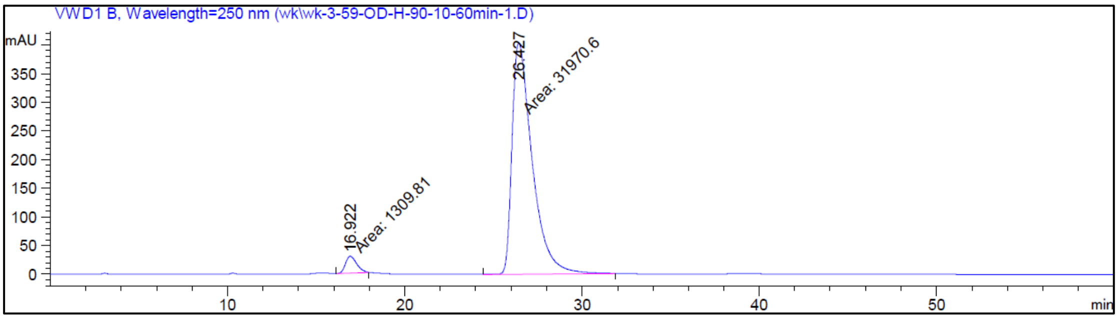

Peak #	RetTime [min]	Type	Width [min]	Area [mAU*s]	Height [mAU]	Area %
1	16.922	MM	0.7369	1309.80725	29.62495	3.9357
2	26.427	MM	1.3202	3.19706e4	403.61017	96.0643

*(R)-6-Chloro-[1,1'-binaphthalene]-2,2'-diol (R)-**1j***

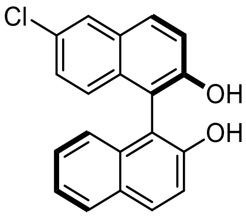

(*R*)-**1j**

According to *gerneral procedure B*, the reaction of *rac*-**1j** (0.1 mmol) under the catalysis of CuCl (0.001 mmol), ligand **L8** (0.001 mmol), and LPL-311-Celite (3.0 w/w) in the presenece of Na_2CO_3 (0.15 mmol) at 50 °C in toluene/vinyl acetate (1.0 mL:0.2 mL) for 60 h under an air atmosphere and hydrolysising afforded (*R*)-**1j** (29.8 mg, 93% yield, 88% *ee*): white solid. MP: 140−142 °C; $[\alpha]^{22}_{D}$ −36.4 (*c* = 0.64, $CHCl_3$); **^{1}H NMR**

(400 MHz, Chloroform-*d*) δ 7.91 (d, *J* = 8.9 Hz, 1H), 7.84 (d, *J* = 8.1 Hz, 1H), 7.83-7.77 (m, 2H), 7.39-7.24 (m, 4H), 7.23-7.18 (m, 1H), 7.05 (t, *J* = 8.1 Hz, 2H), 5.06 (d, *J* = 25.7 Hz, 2H); **^{13}C NMR** (101 MHz, Chloroform-*d*) δ 152.82, 152.63, 133.26, 131.75, 131.55, 130.32, 129.98, 129.81, 129.37, 128.41, 128.16, 127.59, 127.01, 125.93, 124.13, 123.96, 118.90, 117.73, 111.27, 110.32. **HRMS (EI)** *m/z* calcd for $C_{20}H_{13}{}^{35}ClO_2$ [M$^+$]: 320.0599, found: 320.0600. **HPLC conditions**: CHIRALPAK OD-H column, 25 °C, wavelength = 250 nm, *n*-Hexane/*i*-PrOH = 90:10, flow rate = 1.0 mL/min, t_R = 23.10 min for major isomer, t_S = 15.58 min for minor isomer.

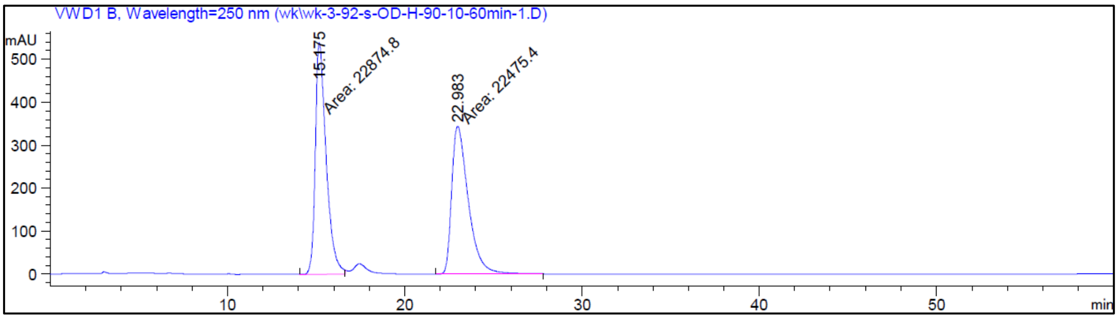

Peak #	RetTime [min]	Type	Width [min]	Area [mAU*s]	Height [mAU]	Area %
1	15.175	MM	0.7084	2.28748e4	538.19000	50.4404
2	22.983	MF	1.0891	2.24754e4	343.93604	49.5596

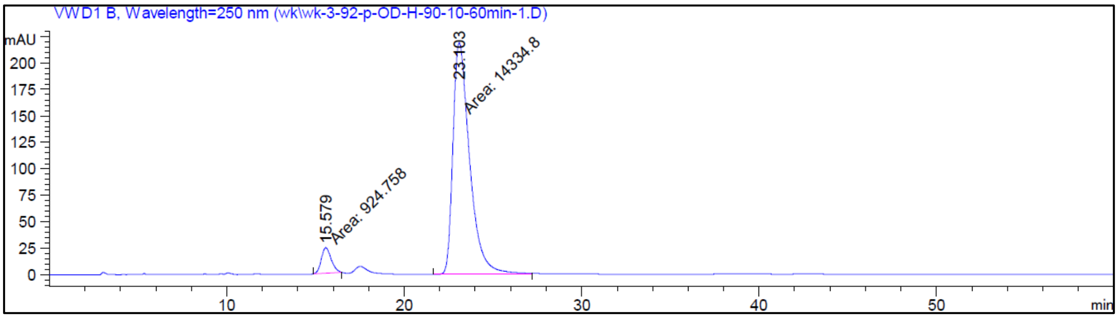

Peak #	RetTime [min]	Type	Width [min]	Area [mAU*s]	Height [mAU]	Area %
1	15.579	MM	0.6419	924.75793	24.01087	6.0602
2	23.103	MF	1.0914	1.43348e4	218.90149	93.9398

*(R)-Methyl 2,2'-dihydroxy-[1,1'-binaphthalene]-6-carboxylate (R)-**1k***

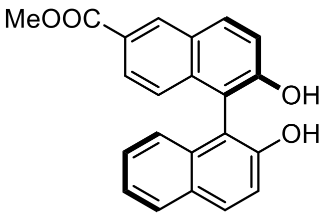

(*R*)-**1k**

According to *gerneral procedure B*, the reaction of *rac*-**1k** (0.1 mmol) under the catalysis of CuCl (0.001 mmol), ligand **L8** (0.001 mmol), and LPL-311-Celite (2.5 w/w) in the presenece of Na_2CO_3 (0.15 mmol) at 50 °C in toluene/vinyl acetate (1.0 mL:0.2 mL) for 60 h under an air atmosphere and hydrolysising afforded (*R*)-**1k** (24.8 mg, 72% yield, 84% *ee*): yellow solid. MP: 199−200 °C; $[\alpha]^{23}_{D}$ −50.8 (*c* = 1.00, $CHCl_3$); **^{1}H NMR** (400 MHz, Chloroform-*d*) δ 8.31 (s, 1H), 7.89 (t, *J* = 9.0 Hz, 2H), 7.82 (d, *J* = 8.0 Hz, 1H), 7.74-7.66 (m, 1H), 7.34 (d, *J* = 8.8 Hz, 2H), 7.31 (d, *J* = 7.7 Hz, 1H), 7.23 (t, *J* = 3.4 Hz, 1H), 7.12 (d, *J* = 8.8 Hz, 1H), 7.04 (d, *J* = 8.3 Hz, 1H), 5.84 (s, 1H), 5.52 (s, 1H), 3.82 (s, 3H); **^{13}C NMR** (101 MHz, Chloroform-*d*) δ 167.16, 154.71, 153.01, 136.10, 133.36, 132.51, 131.47, 131.34, 129.30, 128.35, 128.22, 127.43, 126.53, 125.00, 124.45, 123.95, 123.92, 118.51, 118.09, 111.91, 110.36, 52.17. **HRMS (EI)** *m/z* calcd for $C_{22}H_{16}O_4$ [M^+]: 344.1043, found: 344.1043. **HPLC conditions**: CHIRALPAK OD-H column, 25 °C, wavelength = 250 nm, *n*-Hexane/*i*-PrOH = 80:20, flow rate = 1.0 mL/min, t_R = 14.89 min for major isomer, t_S = 10.95 min for minor isomer.

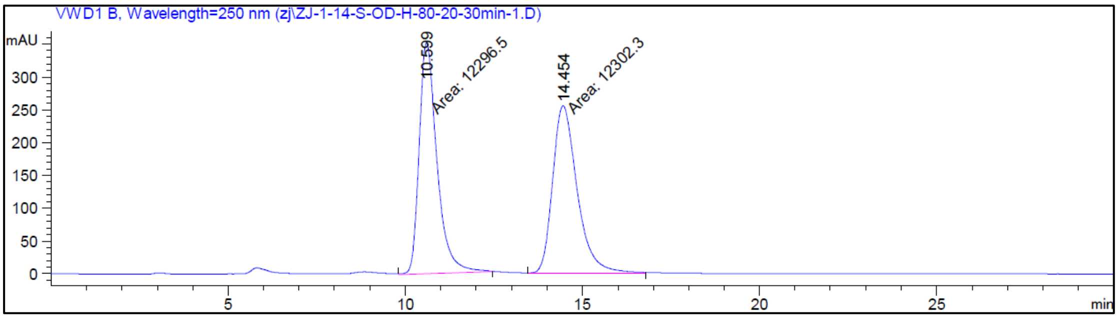

Peak #	RetTime [min]	Type	Width [min]	Area [mAU*s]	Height [mAU]	Area %
1	10.599	MM	0.5824	1.22965e4	351.90485	49.9884
2	14.454	MF	0.8043	1.23023e4	254.93585	50.0116

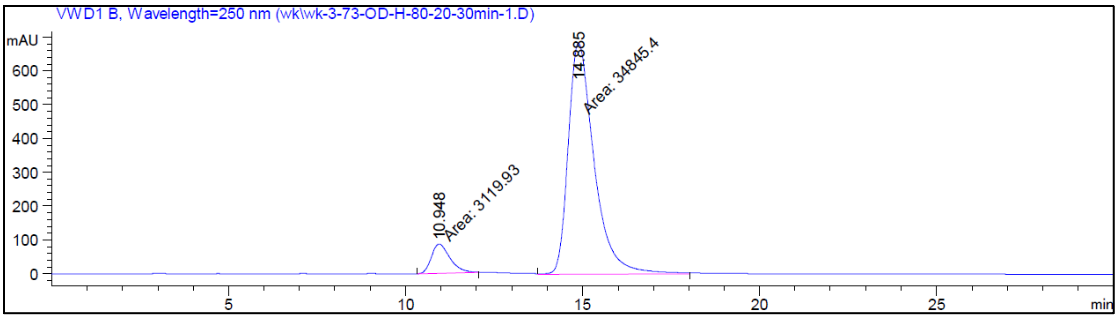

Peak #	RetTime [min]	Type	Width [min]	Area [mAU*s]	Height [mAU]	Area %
1	10.948	MM	0.6036	3119.93115	86.15148	8.2178
2	14.885	MM	0.8498	3.48454e4	683.39801	91.7822

*(R)-6-Phenyl-[1,1'-binaphthalene]-2,2'-diol (R)-**1l***

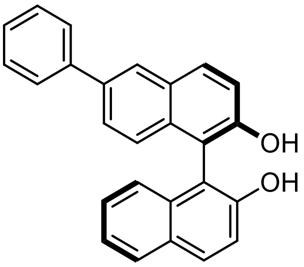

(*R*)-**1l**

According to *gerneral procedure B*, the reaction of *rac*-**1l** (0.1 mmol) under the catalysis of CuCl (0.001 mmol), ligand **L8** (0.001 mmol), and LPL-311-Celite (2.4 w/w) in the presenece of Na_2CO_3 (0.15 mmol) at 50 °C in toluene/vinyl acetate (1.0 mL:0.2 mL) for 60 h under an air atmosphere and hydrolysising afforded (*R*)-**1l** (33.3 mg, 92% yield, 90% *ee*): white solid. MP: 199−200 °C; $[\alpha]^{22}_{D}$ −87.7 (*c* = 0.20, $CHCl_3$); **^{1}H NMR** (400 MHz, Chloroform-*d*) δ 8.04 (d, *J* = 1.5 Hz, 1H), 7.95 (d, *J* = 8.9 Hz, 1H), 7.91 (d, *J* = 9.0 Hz, 1H), 7.85 (d, *J* = 7.8 Hz, 1H), 7.64 (d, *J* = 7.3 Hz, 2H), 7.53 (dd, *J* = 8.7, 1.8 Hz, 1H), 7.43 (t, *J* = 7.6 Hz, 2H), 7.40-7.27 (m, 5H), 7.17 (dd, *J* = 12.2, 8.6 Hz, 2H), 5.11 (s, 2H); **^{13}C NMR** (101 MHz, Chloroform-*d*) δ 152.77, 152.70, 140.76, 136.83, 133.38, 132.57, 131.58, 131.38, 129.66, 129.39, 128.83, 128.37, 127.49, 127.23,

127.19, 127.07, 126.27, 124.77, 124.16, 124.03, 118.18, 117.75, 110.88, 110.81. **HRMS (EI)** *m/z* calcd for $C_{26}H_{18}O_2$ [M^+]: 362.1301, found: 362.1302. **HPLC conditions**: CHIRALPAK OD-H column, 25 ºC, wavelength = 250 nm, *n*-Hexane/*i*-PrOH = 90:10, flow rate = 1.0 mL/min, t_R = 29.46 min for major isomer, t_S = 19.56 min for minor isomer.

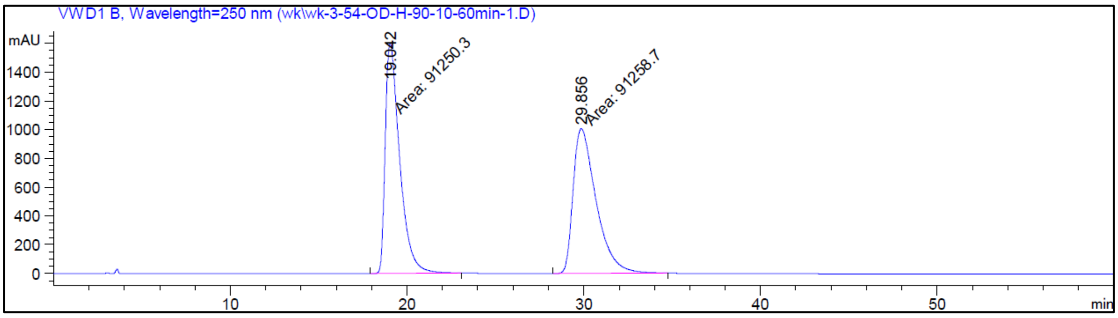

Peak #	RetTime [min]	Type	Width [min]	Area [mAU*s]	Height [mAU]	Area %
1	19.042	MM	0.9476	9.12503e4	1604.92590	49.9977
2	29.856	MM	1.5083	9.12587e4	1008.42877	50.0023

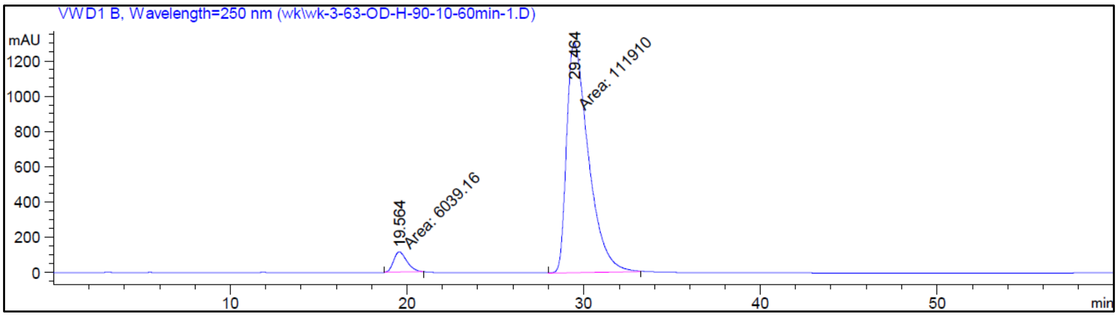

Peak #	RetTime [min]	Type	Width [min]	Area [mAU*s]	Height [mAU]	Area %
1	19.564	MM	0.8746	6039.15576	115.07768	5.1202
2	29.464	MM	1.4312	1.11910e5	1303.17993	94.8798

*(R)-6-(4-Methoxyphenyl)-[1,1'-binaphthalene]-2,2'-diol (R)-**1m***

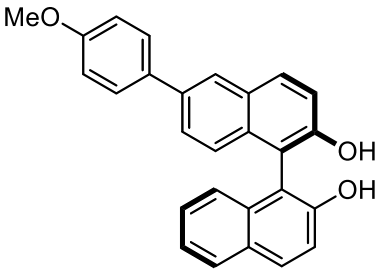

(*R*)-**1m**

According to *gerneral procedure B*, the reaction of *rac*-**1m** (0.1 mmol) under the catalysis of CuCl (0.001 mmol), ligand **L8** (0.001 mmol), and LPL-311-Celite (2.55 w/w) in the presenece of Na_2CO_3 (0.15 mmol) at 50 °C in toluene/vinyl acetate (1.0 mL:0.2 mL) for 60 h under an air atmosphere and hydrolysising afforded (*R*)-**1m** (37.2 mg, 95% yield, 93% *ee*): light yellow solid. MP: 186−187 °C; $[\alpha]^{23}_{D}$ −95.8 (*c* = 1.05, $CHCl_3$); **^{1}H NMR** (400 MHz, Chloroform-*d*) δ 8.00 (d, 1H), 7.97 (d, *J* = 5.9 Hz, 1H), 7.94 (d, *J* = 5.9 Hz, 1H), 7.87 (d, *J* = 7.9 Hz, 1H), 7.58 (d, *J* = 8.7 Hz, 2H), 7.51 (dd, *J* = 8.7, 1.7 Hz, 1H), 7.40-7.33 (m, 3H), 7.33-7.28 (m, 1H), 7.18 (d, *J* = 8.5 Hz, 2H), 6.97 (d, *J* = 8.7 Hz, 2H), 5.12 (s, 1H), 5.07 (s, 1H), 3.83 (s, 3H); **^{13}C NMR** (101 MHz, Chloroform-*d*) δ 159.14, 152.74, 152.59, 136.50, 133.41, 133.31, 132.23, 131.49, 131.39, 129.78, 129.44, 128.40, 128.20, 127.50, 126.94, 125.57, 124.71, 124.20, 124.03, 118.13, 117.77, 114.32, 110.85, 55.33. **HRMS (EI)** *m/z* calcd for $C_{27}H_{20}O_3$ [M^+]: 392.1407, found: 392.1411. **HPLC conditions**: CHIRALPAK OD-H column, 25 °C, wavelength = 250 nm, *n*-Hexane/*i*-PrOH = 80:20, flow rate = 1.0 mL/min, t_R = 20.32 min for major isomer, t_S = 13.99 min for minor isomer.

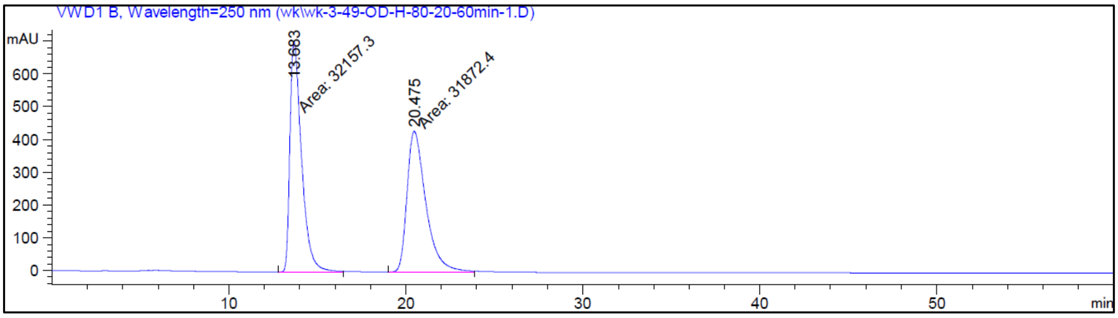

Peak #	RetTime [min]	Type	Width [min]	Area [mAU*s]	Height [mAU]	Area %
1	13.683	MF	0.7623	3.21573e4	703.07568	50.2225
2	20.475	MF	1.2366	3.18724e4	429.56824	49.7775

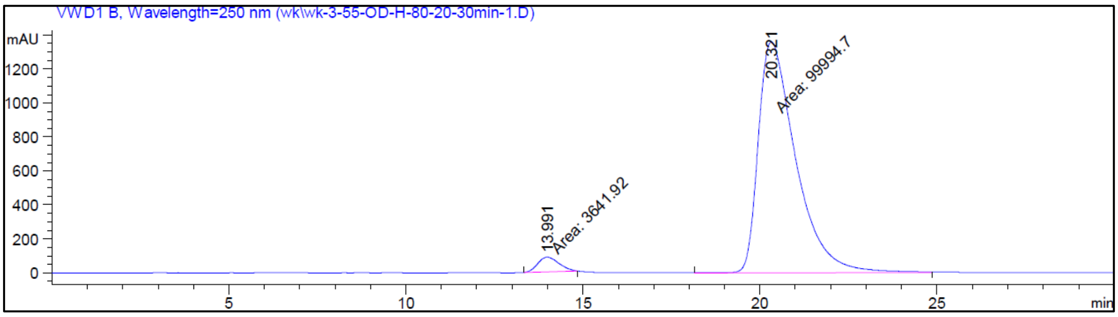

Peak #	RetTime [min]	Type	Width [min]	Area [mAU*s]	Height [mAU]	Area %
1	13.991	MM	0.6961	3641.91650	87.19928	3.5141
2	20.321	MM	1.2272	9.99947e4	1358.07861	96.4859

*(R)-6-(4-Chlorophenyl)-[1,1'-binaphthalene]-2,2'-diol (R)-**1n***

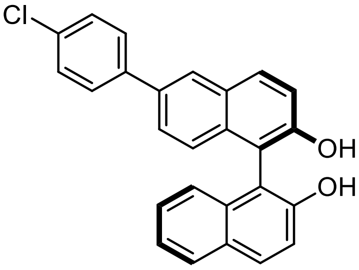

(*R*)-**1n**

According to *gerneral procedure B*, the reaction of *rac*-**1n** (0.1 mmol), under the catalysis of CuCl (0.001 mmol), ligand **L8** (0.001 mmol), and LPL-311-Celite (2.2 w/w) in the presenece of Na_2CO_3 (0.15 mmol) at 50 °C in toluene/vinyl acetate (1.0 mL:0.2 mL) for 60 h under an air atmosphere and hydrolysising afforded (*R*)-**1n** (37.3 mg, 94% yield, 92% *ee*): white solid. MP: 104−109 °C; $[\alpha]^{23}_{D}$ −85.4 (*c* = 0.32, $CHCl_3$); **^{1}H NMR** (400 MHz, Chloroform-*d*) δ 7.98 (s, 1H), 7.92 (dd, *J* = 8.8, 5.2 Hz, 2H), 7.84 (d, *J* = 8.0 Hz, 1H), 7.54 (d, *J* = 8.4 Hz, 2H), 7.45 (d, *J* = 8.7 Hz, 1H), 7.41-7.31 (m, 5H), 7.27 (t, *J* = 7.4 Hz, 1H), 7.15 (dd, *J* = 15.7, 8.5 Hz, 2H), 5.11 (d, *J* = 4.3 Hz, 2H); **^{13}C NMR** (101 MHz, Chloroform-*d*) δ 152.93, 152.70, 139.19, 135.54, 133.37, 133.34, 132.73, 131.54, 131.43, 129.59, 129.40, 128.98, 128.40, 128.37, 127.51, 126.67, 126.18,

124.96, 124.11, 124.06, 118.37, 117.76, 111.00, 110.71. **HRMS (EI)** *m/z* calcd for $C_{26}H_{17}{}^{35}ClO_2$ [M^+]: 396.0912, found: 396.0915. **HPLC conditions**: CHIRALPAK OD-H column, 25 °C, wavelength = 250 nm, *n*-Hexane/*i*-PrOH = 80:20, flow rate = 1.0 mL/min, t_R = 15.21 min for major isomer, t_S = 10.39 min for minor isomer.

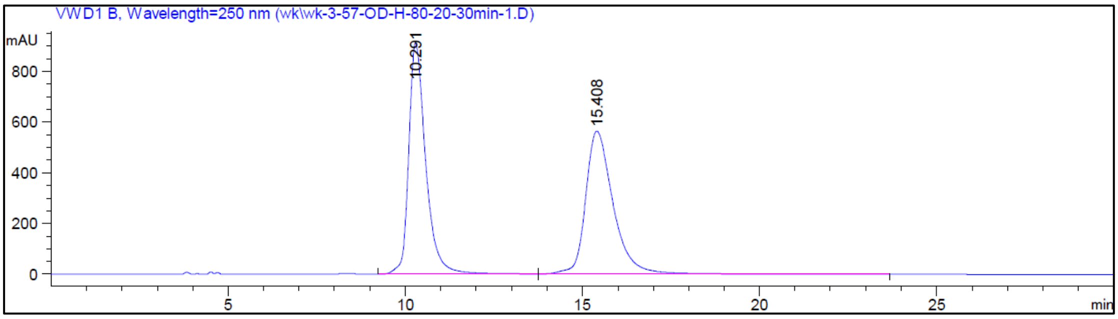

Peak #	RetTime [min]	Type	Width [min]	Area [mAU*s]	Height [mAU]	Area %
1	10.291	BB	0.5027	3.03766e4	911.66711	49.9152
2	15.408	BB	0.8166	3.04799e4	563.91400	50.0848

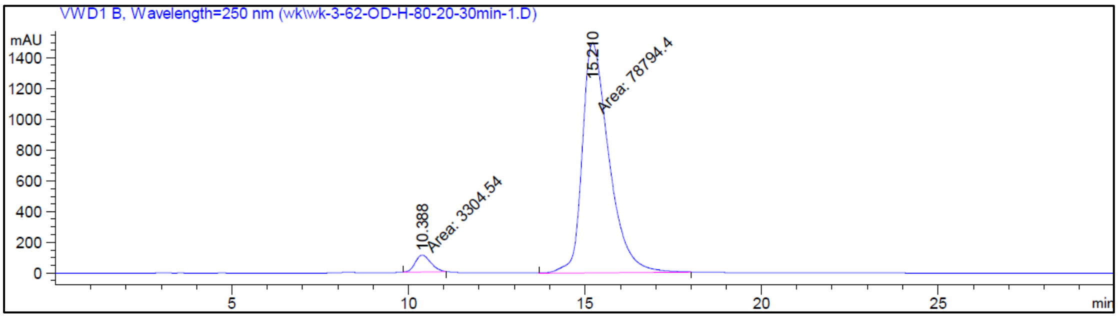

Peak #	RetTime [min]	Type	Width [min]	Area [mAU*s]	Height [mAU]	Area %
1	10.388	MM	0.5051	3304.53613	109.04458	4.0251
2	15.210	MM	0.8776	7.87944e4	1496.41968	95.9749

*(R)-6-(Furan-2-yl)-[1,1'-binaphthalene]-2,2'-diol (R)-***1o**

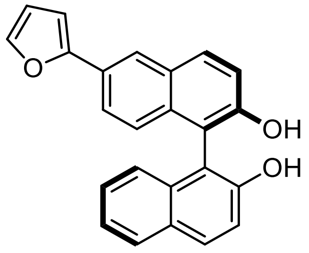

(*R*)-**1o**

According to *gerneral procedure B*, the reaction of *rac*-**1o** (0.1 mmol) under the

catalysis of CuCl (0.001 mmol), ligand **L8** (0.001 mmol), and LPL-311-Celite (2.4 w/w) in the presenece of Na_2CO_3 (0.15 mmol) at 50 °C in toluene/vinyl acetate (1.0 mL:0.2 mL) for 48 h under an air atmosphere and hydrolysising afforded (*R*)-**1o** (32.5 mg, 92% yield, 87% *ee*): light yellow solid. MP: 180−182 °C; $[\alpha]^{23}_{D}$ −102.3 (*c* = 0.47, $CHCl_3$); **^{1}H NMR** (400 MHz, Chloroform-*d*) δ 8.15 (s, 1H), 7.94 (d, *J* = 8.9 Hz, 2H), 7.86 (d, *J* = 8.0 Hz, 1H), 7.56 (dd, *J* = 8.8, 1.5 Hz, 1H), 7.48 (d, 1H), 7.34 (d, *J* = 8.6 Hz, 3H), 7.29 (t, *J* = 7.1 Hz, 1H), 7.13 (t, *J* = 8.3 Hz, 2H), 6.66 (d, *J* = 3.2 Hz, 1H), 6.47 (dd, *J* = 3.1, 1.7 Hz, 1H), 5.08 (d, *J* = 6.5 Hz, 2H); **^{13}C NMR** (101 MHz, Chloroform-*d*) δ 153.84, 152.78, 152.68, 142.15, 133.38, 132.63, 131.48, 131.37, 129.46, 129.38, 128.36, 127.48, 126.66, 124.68, 124.15, 124.02, 123.89, 122.68, 118.31, 117.75, 111.71, 111.23, 110.76, 105.18. **HRMS (EI)** *m/z* calcd for $C_{24}H_{16}O_3$ [M^+]: 352.1094, found: 352.1097. **HPLC conditions**: CHIRALPAK OD-H column, 25 °C, wavelength = 250 nm, *n*-Hexane/*i*-PrOH = 80:20, flow rate = 1.0 mL/min, t_R = 15.16 min for major isomer, t_S = 10.39 min for minor isomer.

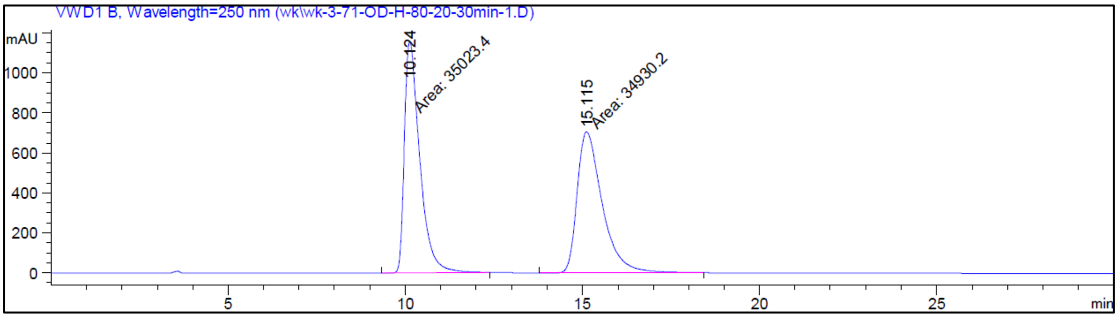

Peak #	RetTime [min]	Type	Width [min]	Area [mAU*s]	Height [mAU]	Area %
1	10.124	MM	0.5045	3.50234e4	1156.96606	50.0667
2	15.115	MM	0.8247	3.49302e4	705.95807	49.9333

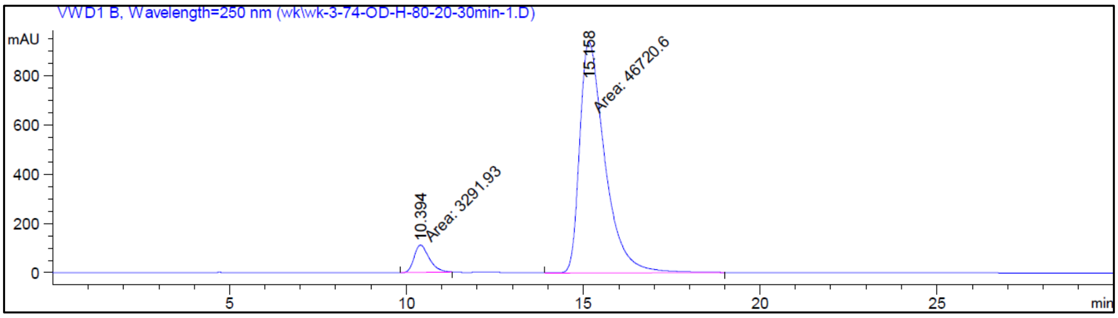

Peak #	RetTime [min]	Type	Width [min]	Area [mAU*s]	Height [mAU]	Area %
1	10.394	MM	0.4947	3291.93359	110.90903	6.5822
2	15.158	MM	0.8301	4.67206e4	938.02557	93.4178

*(R)-6-(Thiophen-2-yl)-[1,1'-binaphthalene]-2,2'-diol (R)-**1p***

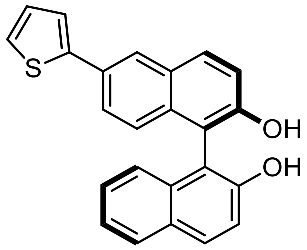

(*R*)-**1p**

According to *gerneral procedure B*, the reaction of *rac*-**1p** (0.1 mmol) under the catalysis of CuCl (0.001 mmol), ligand **L8** (0.001 mmol), and LPL-311-Celite (2.3 w/w) in the presenece of Na_2CO_3 (0.15 mmol) at 50 °C in toluene/vinyl acetate (1.0 mL:0.2 mL) for 36 h under an air atmosphere and hydrolysising afforded (*R*)-**1p** (32.1 mg, 87% yield, 94% *ee*): light yellow solid. MP: 127−130 °C; $[\alpha]^{23}_{D}$ −102.1 (*c* = 0.49, $CHCl_3$); **^{1}H NMR** (400 MHz, Chloroform-*d*) δ 8.05 (d, *J* = 1.6 Hz, 1H), 7.93 (d, *J* = 3.6 Hz, 1H), 7.91 (d, *J* = 3.6 Hz, 1H), 7.85 (d, *J* = 7.8 Hz, 1H), 7.53 (dd, *J* = 8.8, 1.8 Hz, 1H), 7.37-7.24 (m, 6H), 7.16-7.10 (m, 2H), 7.06 (dd, *J* = 5.0, 3.6 Hz, 1H), 5.09 (s, 2H); **^{13}C NMR** (101 MHz, Chloroform-*d*) δ 152.81, 152.69, 144.16, 133.36, 132.70, 131.43, 131.38, 130.22, 129.59, 129.40, 128.39, 128.09, 127.52, 125.98, 124.88, 124.80, 124.13, 124.06, 123.20, 118.42, 117.75, 111.15, 110.67. **HRMS (EI)** *m/z* calcd for $C_{24}H_{16}O_2S$ [M^+]: 368.0866, found: 368.0866. **HPLC conditions**: CHIRALPAK OD-H column, 25 °C, wavelength = 250 nm, *n*-Hexane/*i*-PrOH = 90:10, flow rate = 1.0 mL/min, t_R = 35.91 min for major isomer, t_S = 23.18 min for minor isomer.

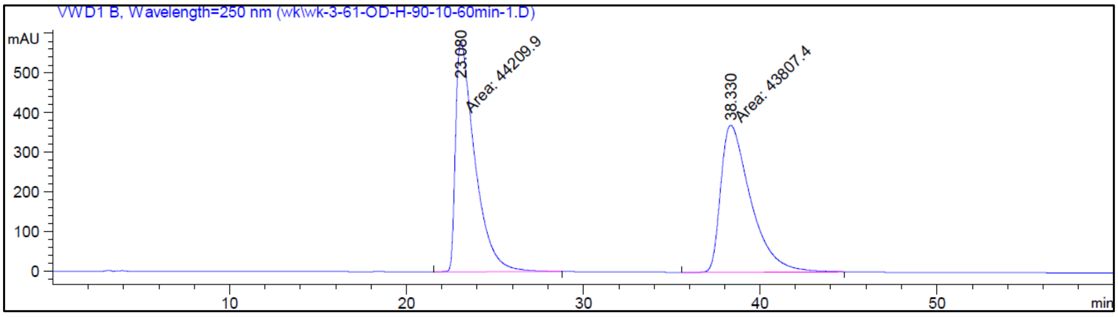

Peak #	RetTime [min]	Type	Width [min]	Area [mAU*s]	Height [mAU]	Area %
1	23.080	MM	1.2680	4.42099e4	581.08643	50.2287
2	38.330	MM	1.9691	4.38074e4	370.78165	49.7713

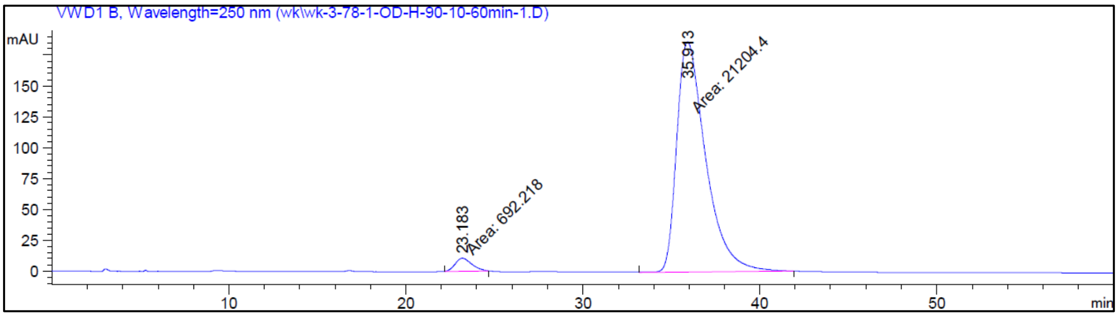

Peak #	RetTime [min]	Type	Width [min]	Area [mAU*s]	Height [mAU]	Area %
1	23.183	MM	1.0844	692.21796	10.63924	3.1613
2	35.913	MM	1.8964	2.12044e4	186.35306	96.8387

*(R)-6-Bromo-6'-(4-methoxyphenyl)-[1,1'-binaphthalene]-2,2'-diol (R)-**1q***

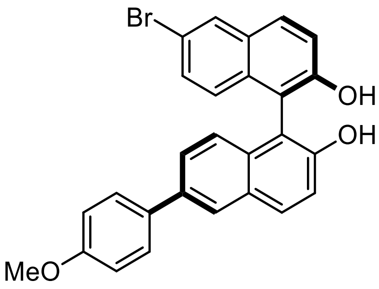

(*R*)-**1q**

According to *gerneral procedure B*, the reaction of *rac*-**1q** (0.1 mmol) under the catalysis of CuCl (0.001 mmol), ligand **L8** (0.001 mmol), and LPL-311-Celite (2.1 w/w) in the presenece of Na_2CO_3 (0.15 mmol) at 50 °C in toluene/vinyl acetate (1.0 mL:0.2 mL) for 60 h under an air atmosphere and hydrolysising afforded (*R*)-**1q** (44.7 mg, 95%

yield, 96% *ee*): white solid. MP: 129−131 °C; $[\alpha]^{23}_{D}$ −204.6 (*c* = 1.54, $CHCl_3$); **^{1}H NMR** (400 MHz, Chloroform-*d*) δ 8.04 (dd, *J* = 8.3, 1.7 Hz, 2H), 8.00 (d, *J* = 9.0 Hz, 1H), 7.88 (d, *J* = 9.0 Hz, 1H), 7.59 (d, *J* = 8.7 Hz, 2H), 7.54 (dd, *J* = 8.7, 1.8 Hz, 1H), 7.43-7.34 (m, 3H), 7.13 (d, *J* = 8.7 Hz, 1H), 7.05 (d, *J* = 9.0 Hz, 1H), 6.99 (d, *J* = 8.8 Hz, 2H), 5.12 (s, 2H), 3.85 (s, 3H); **^{13}C NMR** (101 MHz, Chloroform-*d*) δ 159.23, 153.02, 152.58, 136.68, 133.21, 132.10, 132.04, 131.77, 130.71, 130.57, 130.42, 130.37, 129.81, 128.22, 127.11, 126.13, 125.65, 124.53, 118.97, 118.19, 117.87, 114.36, 111.35, 110.25, 55.36. **HRMS (ESI)** *m/z* calcd for $C_{27}H_{19}{}^{81}BrO_3$ [M^+]: 472.0501, found: 472.0490. **HPLC conditions**: CHIRALPAK OD-H column, 25 °C, wavelength = 250 nm, *n*-Hexane/*i*-PrOH = 80:20, flow rate = 1.0 mL/min, t_R = 27.06 min for major isomer, t_S = 12.76 min for minor isomer.

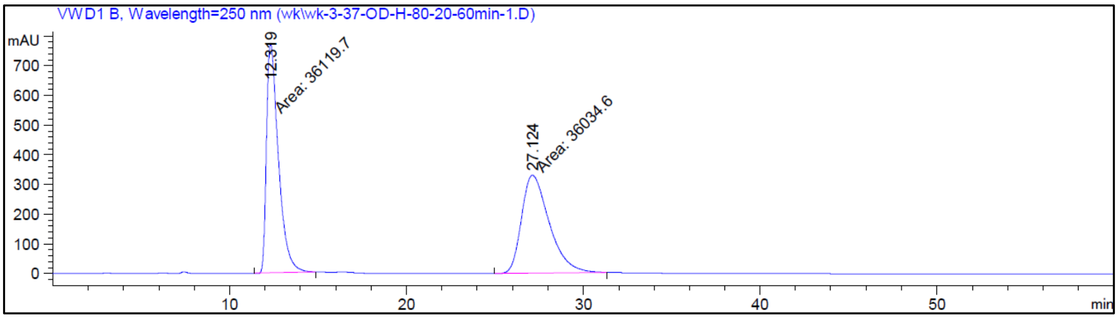

Peak #	RetTime [min]	Type	Width [min]	Area [mAU*s]	Height [mAU]	Area %
1	12.319	MM	0.7776	3.61197e4	774.19464	50.0590
2	27.124	MM	1.8168	3.60346e4	330.57236	49.9410

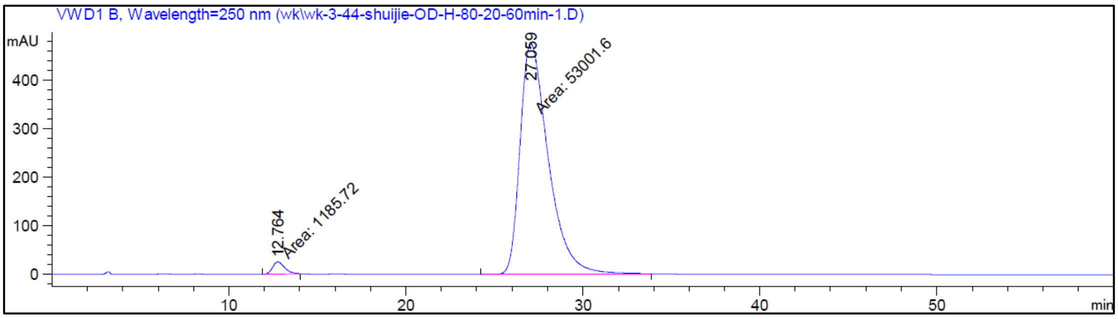

Peak #	RetTime [min]	Type	Width [min]	Area [mAU*s]	Height [mAU]	Area %
1	12.764	MM	0.7866	1185.72290	25.12433	2.1882
2	27.059	MM	1.8563	5.30016e4	475.87466	97.8118

*(R)-6-Bromo-6'-methyl-[1,1'-binaphthalene]-2,2'-diol (R)-**1r***

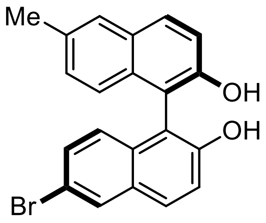

(*R*)-**1r**

According to *gerneral procedure B*, the reaction of *rac*-**1r** (0.1 mmol), under the catalysis of CuCl (0.001 mmol), ligand **L8** (0.001 mmol), and LPL-311-Celite (2.3 w/w) in the presenece of Na_2CO_3 (0.15 mmol) at 50 °C in toluene/vinyl acetate (1.0 mL:0.2 mL) for 48 h under an air atmosphere and hydrolysising afforded (*R*)-**1r** (32.1 mg, 85% yield, 80% *ee*): white solid. MP: 175−177 °C; $[\alpha]^{23}_{D}$ −78.5 (*c* = 0.67, $CHCl_3$); **^{1}H NMR** (400 MHz, Chloroform-*d*) δ 8.00 (d, *J* = 1.9 Hz, 1H), 7.82 (dd, *J* = 8.8, 6.6 Hz, 2H), 7.62 (s, 1H), 7.38-7.26 (m, 3H), 7.13 (dd, *J* = 8.6, 1.4 Hz, 1H), 6.97 (t, *J* = 9.1 Hz, 2H), 5.10 (s, 1H), 4.91 (s, 1H), 2.45 (s, 3H); **^{13}C NMR** (101 MHz, Chloroform-*d*) δ 152.90, 151.96, 133.75, 132.01, 131.34, 130.90, 130.60, 130.48, 130.27, 130.24, 129.80, 129.60, 127.51, 126.12, 123.84, 118.86, 117.78, 117.71, 111.48, 110.08, 21.29. **HRMS (EI)** *m/z* calcd for $C_{21}H_{15}{}^{81}BrO_2$ [M^+]: 380.0232, found: 380.0226. **HPLC conditions**: CHIRALPAK OD-H column, 25 °C, wavelength = 250 nm, *n*-Hexane/*i*-PrOH = 90:10, flow rate = 1.0 mL/min, t_R = 26.02 min for major isomer, t_S = 12.34 min for minor isomer.

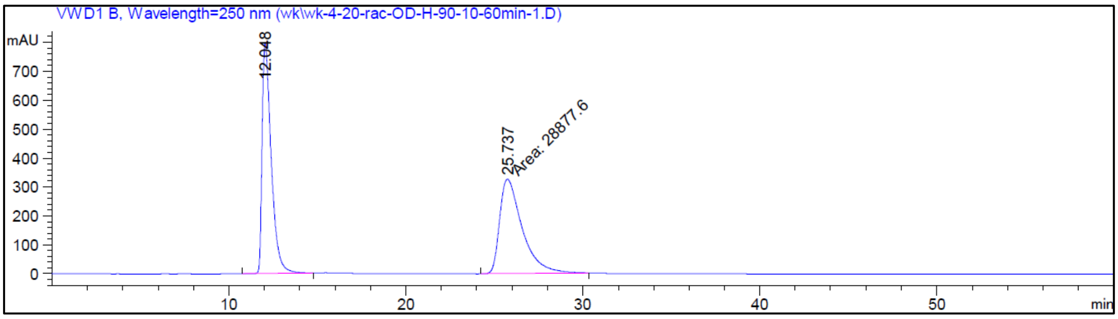

Peak #	RetTime [min]	Type	Width [min]	Area [mAU*s]	Height [mAU]	Area %
1	12.048	BB	0.5396	2.85655e4	798.05060	49.7283
2	25.737	MM	1.4737	2.88776e4	326.58154	50.2717

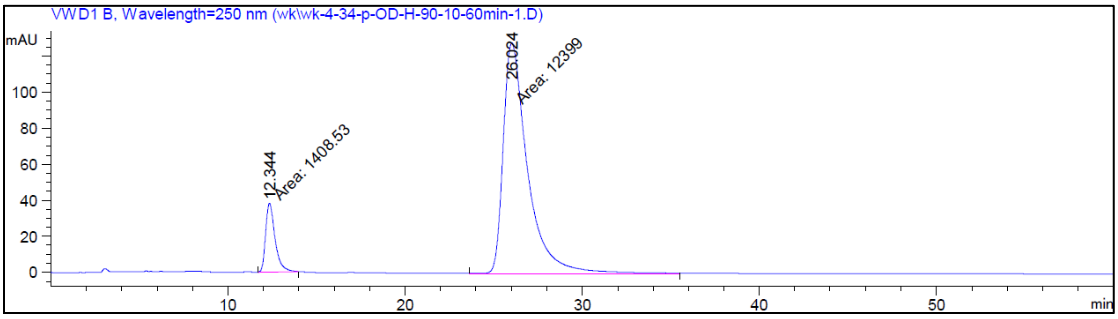

Peak #	RetTime [min]	Type	Width [min]	Area [mAU*s]	Height [mAU]	Area %
1	12.344	MM	0.6155	1408.53186	38.13915	10.2012
2	26.024	MM	1.6132	1.23990e4	128.10106	89.7988

*(R)-7-Bromo-[1,1'-binaphthalene]-2,2'-diol (R)-**1s***

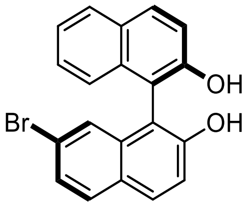

(*R*)-**1s**

According to *gerneral procedure B*, the reaction of *rac*-**1s** (0.1 mmol) under the catalysis of CuCl (0.001 mmol), ligand **L8** (0.001 mmol), and LPL-311-Celite (2.35 w/w) in the presenece of Na_2CO_3 (0.15 mmol) at 50 °C in toluene/vinyl acetate (1.0 mL:0.2 mL) for 60 h under an air atmosphere and hydrolysising afforded (*R*)-**1s** (23.7 mg, 65% yield, 88% *ee*): brown solid. MP: 181−182 °C; $[\alpha]^{23}_{D}$ −62.7 (*c* = 1.12, $CHCl_3$); **^{1}H NMR** (400 MHz, Chloroform-*d*) δ 7.92 (d, *J* = 8.9 Hz, 1H), 7.85 (dd, *J* = 8.3, 4.6 Hz, 2H), 7.70 (d, *J* = 8.7 Hz, 1H), 7.42 (dd, *J* = 8.7, 1.9 Hz, 1H), 7.39-7.24 (m, 5H), 7.08 (d, *J* = 8.3 Hz, 1H), 5.12 (s, 1H), 5.01 (s, 1H); **^{13}C NMR** (101 MHz, Chloroform-*d*) δ 153.49, 152.69, 134.78, 133.20, 131.68, 131.22, 129.97, 129.43, 128.46, 127.80, 127.65, 127.50, 126.20, 124.16, 123.88, 122.16, 118.14, 117.79, 110.42, 110.01. **HRMS (EI)** *m/z* calcd for $C_{20}H_{13}{}^{81}BrO_2$ [M^{+}]: 366.0075, found: 366.0078. **HPLC conditions**: CHIRALPAK OD-H column, 25 °C, wavelength = 250 nm, *n*-Hexane/*i*-PrOH = 80:20, flow rate = 1.0 mL/min, t_R = 11.24 min for major isomer, t_S = 8.90 min

for minor isomer.

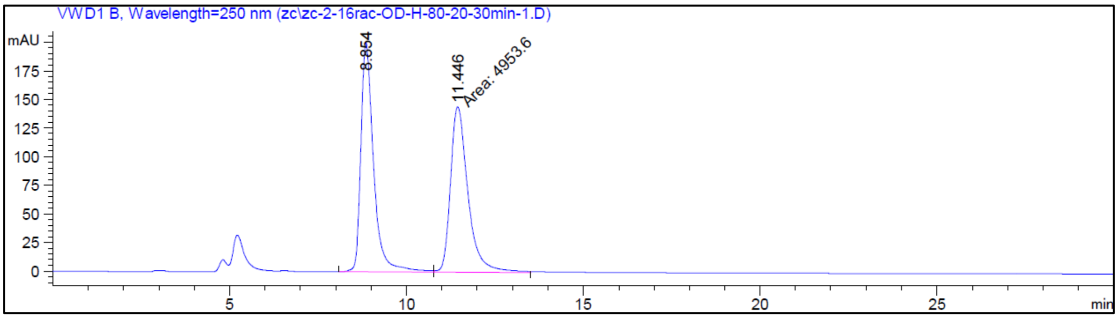

Peak #	RetTime [min]	Type	Width [min]	Area [mAU*s]	Height [mAU]	Area %
1	8.854	BV	0.3767	5012.11328	199.79805	50.2936
2	11.446	MF	0.5724	4953.59521	144.23811	49.7064

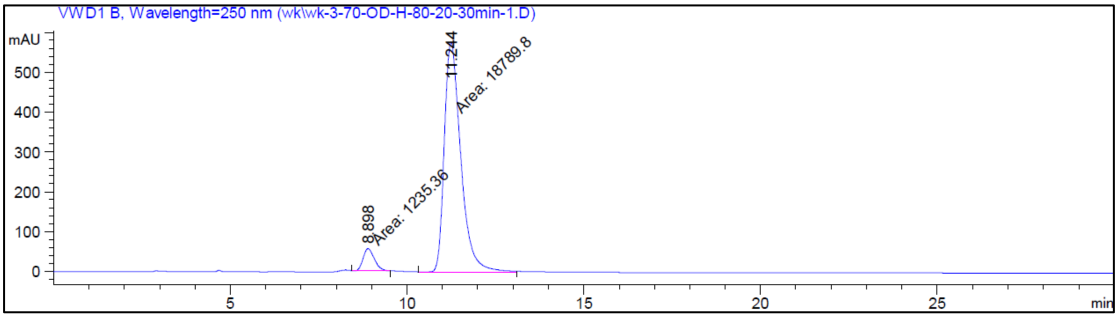

Peak #	RetTime [min]	Type	Width [min]	Area [mAU*s]	Height [mAU]	Area %
1	8.898	MM	0.3674	1235.36292	56.03481	6.1691
2	11.244	MM	0.5427	1.87898e4	577.00458	93.8309

*(R)-7-Methoxy-[1,1'-binaphthalene]-2,2'-diol (R)-**1t***

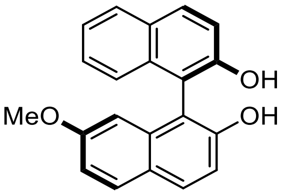

(*R*)-**1t**

According to *gerneral procedure B*, the reaction of *rac*-**1t** (0.1 mmol) under the catalysis of CuCl (0.001 mmol), ligand **L8** (0.001 mmol), and LPL-311-Celite (2.7 w/w) in the presenece of Na_2CO_3 (0.15 mmol) at 50 °C in toluene/vinyl acetate (1.0 mL:0.2 mL) for 60 h under an air atmosphere and hydrolysis to afford (*R*)-**1t** (22.5 mg, 71%

yield, 90% *ee*): white solid. MP: 89−90 °C; $[\alpha]^{23}_{D}$ −56.0 (*c* = 0.85, $CHCl_3$); **^{1}H NMR** (400 MHz, Chloroform-*d*) δ 7.92 (d, *J* = 8.9 Hz, 1H), 7.84 (t, *J* = 8.4 Hz, 2H), 7.74 (d, *J* = 8.9 Hz, 1H), 7.39-7.27 (m, 3H), 7.18 (d, *J* = 8.7 Hz, 2H), 7.00 (dd, *J* = 8.9, 2.2 Hz, 1H), 6.41 (d, 1H), 5.15 (s, 1H), 5.04 (s, 1H), 3.51 (s, 3H); **^{13}C NMR** (101 MHz, Chloroform-*d*) δ 159.06, 153.34, 152.66, 134.89, 133.23, 131.33, 131.03, 129.94, 129.45, 128.35, 127.42, 124.71, 124.20, 123.98, 117.72, 115.98, 115.11, 111.01, 110.02, 103.24, 55.07. **HRMS (EI)** *m/z* calcd for $C_{21}H_{16}O_3$ $[M^+]$: 316.1094, found: 316.1098. **HPLC conditions**: CHIRALPAK OD-H column, 25 °C, wavelength = 250 nm, *n*-Hexane/*i*-PrOH = 90:10, flow rate = 1.0 mL/min, t_R = 22.17 min for major isomer, t_S = 15.45 min for minor isomer.

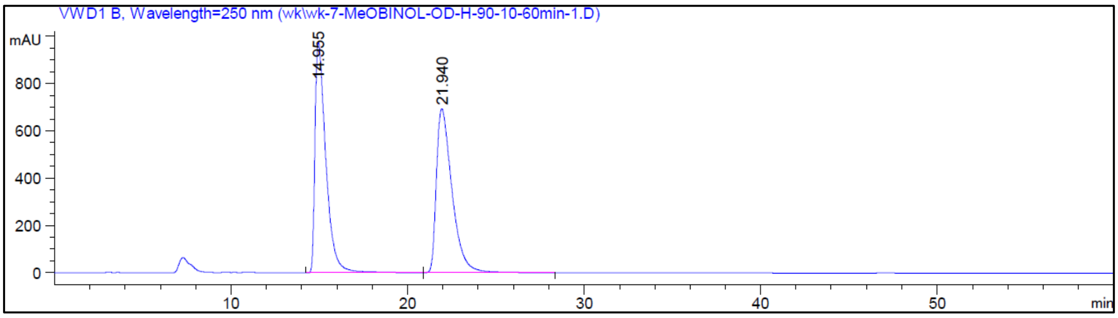

Peak #	RetTime [min]	Type	Width [min]	Area [mAU*s]	Height [mAU]	Area %
1	14.955	BB	0.6441	4.16032e4	970.49396	49.4659
2	21.940	BB	0.9388	4.25016e4	691.80487	50.5341

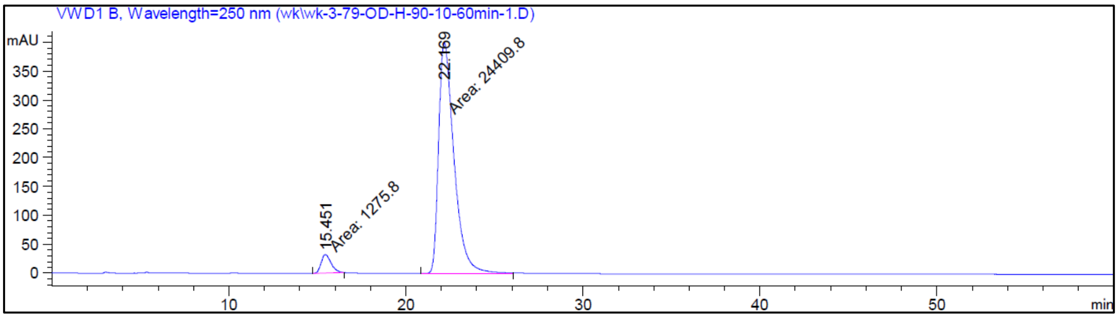

Peak #	RetTime [min]	Type	Width [min]	Area [mAU*s]	Height [mAU]	Area %
1	15.451	MM	0.6564	1275.80286	32.39483	4.9670
2	22.169	MM	1.0170	2.44098e4	400.02695	95.0330

*(R)-3-Bromo-[1,1'-binaphthalene]-2,2'-diol (R)-**1u***

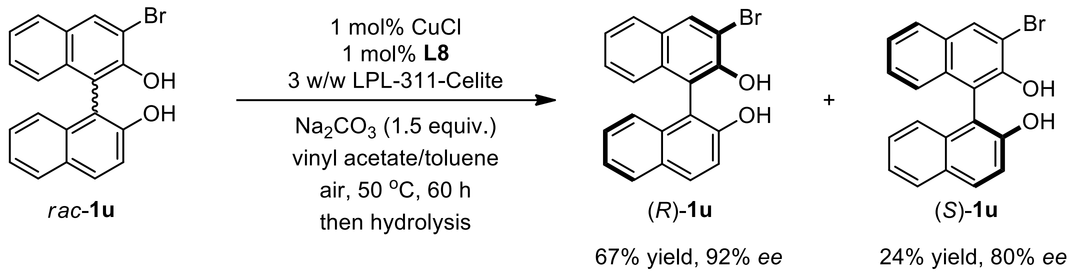

According to *gerneral procedure B*, the reaction of *rac*-**1u** (0.1 mmol) under the catalysis of CuCl (0.001 mmol), ligand **L8** (0.001 mmol), and LPL-311-Celite (2.35 w/w) in the presenece of Na_2CO_3 (0.15 mmol) at 50 °C in toluene/vinyl acetate (1.0 mL:0.2 mL) for 60 h under an air atmosphere afforded (*R*)-**1u**-OAc (then hydrolysis to afford (*R*)-**1u,** 24.5 mg, 67% yield, 92% *ee*) and (*S*)-**1u** (8.8 mg, 24% yield, 80% *ee*).

(*R*)-**1u**: white solid. MP: 139−140 °C; $[\alpha]^{23}_{D}$ +61.3 (*c* = 0.78, $CHCl_3$); **^{1}H NMR** (400 MHz, Chloroform-*d*) δ 8.26 (s, 1H), 7.95 (d, *J* = 8.9 Hz, 1H), 7.87 (d, *J* = 8.0 Hz, 1H), 7.80 (d, *J* = 8.1 Hz, 1H), 7.42-7.27 (m, 5H), 7.13 (d, *J* = 8.4 Hz, 1H), 7.09 (d, *J* = 8.3 Hz, 1H), 5.56 (s, 1H), 4.91 (s, 1H); **^{13}C NMR** (101 MHz, Chloroform-*d*) δ 152.15, 148.87, 133.30, 133.11, 132.86, 131.33, 129.88, 129.34, 128.39, 127.79, 127.43, 127.38, 125.03, 124.60, 124.12, 123.97, 117.73, 113.20, 112.08, 111.73. **HRMS (EI)** *m/z* calcd for $C_{20}H_{13}{}^{81}BrO_2$ $[M^+]$: 366.0075, found: 366.0079. **HPLC conditions**: CHIRALPAK OD-H column, 25 °C, wavelength = 250 nm, *n*-Hexane/*i*-PrOH = 90:10, flow rate = 1.0 mL/min, t_R = 14.93 min for major isomer, t_S = 27.31 min for minor isomer.

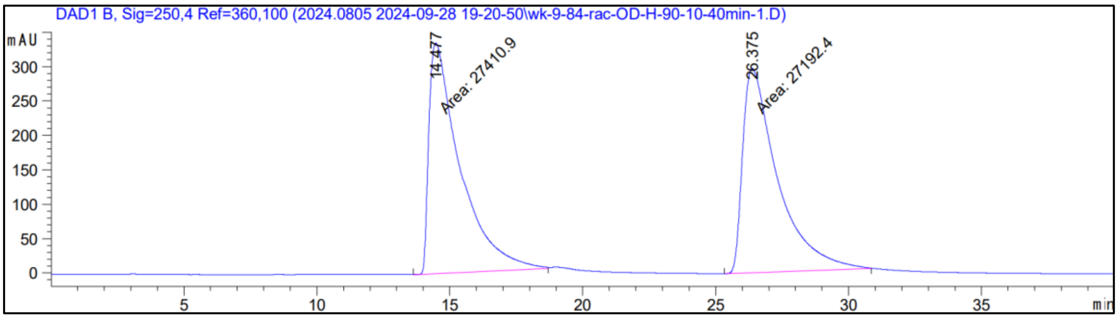

Peak #	RetTime [min]	Type	Width [min]	Area [mAU*s]	Height [mAU]	Area %
1	14.477	MM	1.3628	2.74109e4	335.22284	50.2002
2	26.375	MM	1.5246	2.71924e4	297.25653	49.7998

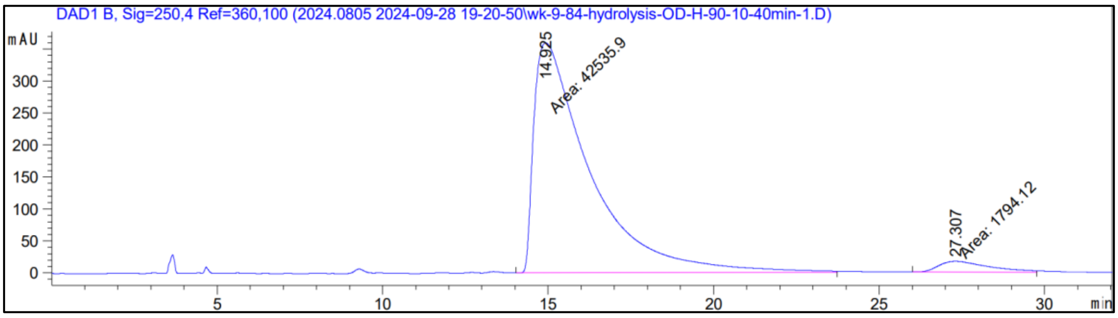

Peak #	RetTime [min]	Type	Width [min]	Area [mAU*s]	Height [mAU]	Area %
1	14.925	MF	1.9700	4.25359e4	359.85437	95.9528
2	27.307	MF	1.7723	1794.11743	16.87142	4.0472

*(R)-3-Phenyl-[1,1'-binaphthalene]-2,2'-diol (R)-**1v***

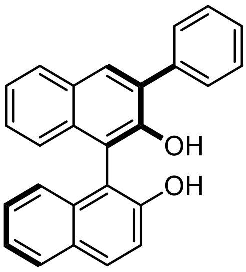

(*R*)-**1v**

According to *gerneral procedure B*, the reaction of *rac*-**1v** (0.1 mmol) under the catalysis of CuCl (0.001 mmol), ligand **L8** (0.001 mmol), and LPL-311-Celite (2.4 w/w) in the presenece of Na_2CO_3 (0.15 mmol) at 50 °C in toluene/vinyl acetate (1.0 mL:0.2 mL) for 60 h under an air atmosphere and hydrolysising afforded (*R*)-**1v** (27.5 mg, 76% yield, 94% *ee*): white solid. MP: 96−97 °C; $[\alpha]^{23}_{D}$ +121.7 (*c* = 0.75, $CHCl_3$); **^{1}H NMR** (400 MHz, Chloroform-*d*) δ 8.01 (s, 1H), 7.94 (d, *J* = 8.9 Hz, 1H), 7.88 (dd, *J* = 12.2, 8.2 Hz, 2H), 7.72 (d, 2H), 7.47 (t, *J* = 7.5 Hz, 2H), 7.42-7.26 (m, 6H), 7.21 (d, *J* = 4.2 Hz, 1H), 7.13 (d, *J* = 8.4 Hz, 1H), 5.29 (s, 1H), 5.10 (s, 1H); **^{13}C NMR** (101 MHz, Chloroform-*d*) δ 152.61, 150.23, 137.38, 133.38, 132.92, 131.45, 131.30, 130.66, 129.57, 129.42, 128.46, 128.41, 128.37, 127.76, 127.38, 124.36, 124.27, 124.15,

123.96, 117.72, 111.74, 111.43. **HRMS (EI)** *m/z* calcd for $C_{26}H_{18}O_2$ [M^+]: 362.1301, found: 362.1300. **HPLC conditions**: CHIRALPAK AD-H column, 25 ºC, wavelength = 250 nm, *n*-Hexane/*i*-PrOH = 90:10, flow rate = 1.0 mL/min, t_R = 24.91 min for major isomer, t_S = 45.33 min for minor isomer.

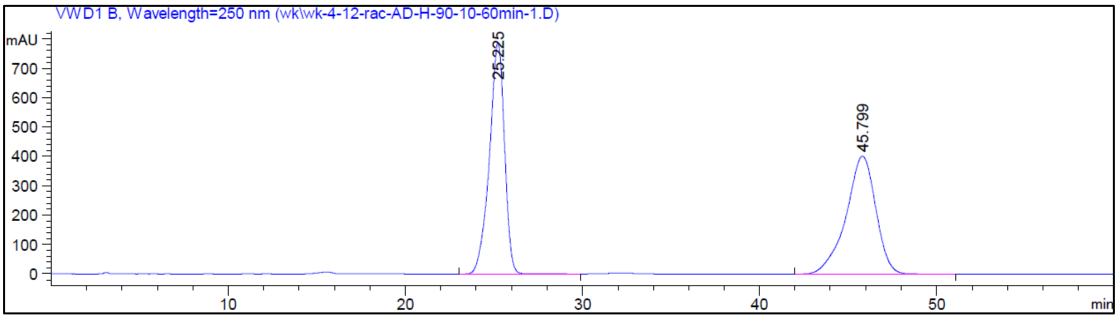

Peak #	RetTime [min]	Type	Width [min]	Area [mAU*s]	Height [mAU]	Area %
1	25.225	BB	0.8952	4.66090e4	786.69482	49.9470
2	45.799	BB	1.7628	4.67080e4	401.04730	50.0530

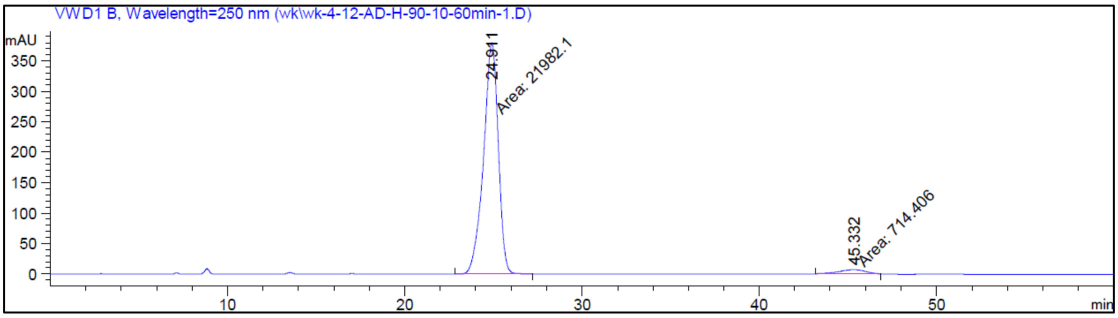

Peak #	RetTime [min]	Type	Width [min]	Area [mAU*s]	Height [mAU]	Area %
1	24.911	MF	0.9647	2.19821e4	379.75839	96.8524
2	45.332	MM	1.7128	714.40613	6.95149	3.1476

*(R)-10-(2-Hydroxynaphthalen-1-yl)phenanthren-9-ol (R)-**1w***

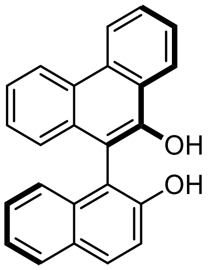

(*R*)-**1w**

According to *gerneral procedure B*, the reaction of *rac*-**1w** (0.1 mmol) under the catalysis of CuCl (0.001 mmol), ligand **L8** (0.001 mmol), and LPL-311-Celite (2.55 w/w) in the presenece of Na_2CO_3 (0.15 mmol) at 50 °C in toluene/vinyl acetate (1.0 mL:0.2 mL) for 60 h under an air atmosphere and hydrolysising afforded (*R*)-**1w** (28.6 mg, 85% yield, 85% *ee*): brown solid. MP: 179−180 °C; $[\alpha]^{23}_{D}$ +36.1 (*c* = 0.33, $CHCl_3$); **^{1}H NMR** (400 MHz, Chloroform-*d*) δ 8.76 (d, *J* = 8.3 Hz, 1H), 8.70 (d, *J* = 8.2 Hz, 1H), 8.43 (d, 1H), 7.93 (d, *J* = 9.0 Hz, 1H), 7.84 (d, *J* = 8.1 Hz, 1H), 7.80-7.75 (m, 1H), 7.69 (t, *J* = 7.2 Hz, 1H), 7.52-7.46 (m, 1H), 7.39-7.30 (m, 3H), 7.25-7.19 (m, 2H), 7.14 (d, *J* = 7.7 Hz, 1H), 5.47 (s, 1H), 5.12 (s, 1H); **^{13}C NMR** (101 MHz, Chloroform-*d*) δ 152.93, 149.11, 133.50, 131.90, 131.63, 131.46, 129.46, 128.37, 128.09, 127.61, 127.50, 126.99, 126.83, 124.91, 124.85, 124.72, 124.30, 124.05, 123.47, 122.87, 122.65, 117.72, 110.98, 106.94. **HRMS (EI)** *m/z* calcd for $C_{24}H_{16}O_2$ [M^+]: 336.1145, found: 336.1145. **HPLC conditions**: CHIRALPAK OD-H column, 25 °C, wavelength = 250 nm, *n*-Hexane/*i*-PrOH = 90:10, flow rate = 1.0 mL/min, t_R = 11.24 min for major isomer, t_S = 16.20 min for minor isomer.

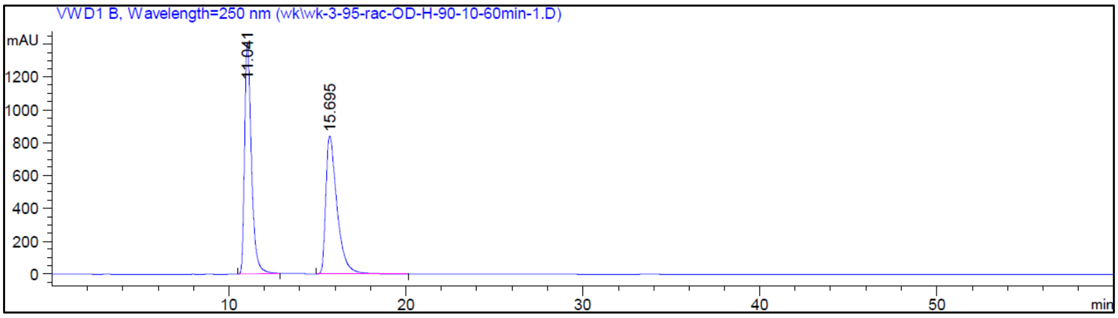

Peak #	RetTime [min]	Type	Width [min]	Area [mAU*s]	Height [mAU]	Area %
1	11.041	BB	0.4097	3.78333e4	1406.90308	50.4602
2	15.695	BB	0.6623	3.71432e4	839.26501	49.5398

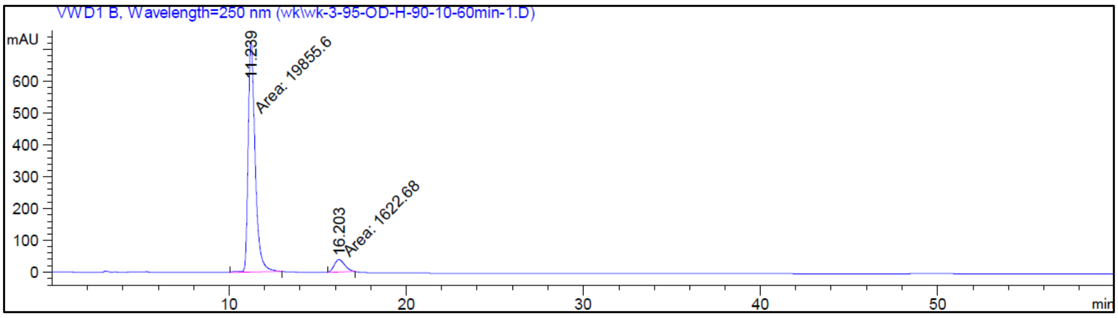

Peak #	RetTime [min]	Type	Width [min]	Area [mAU*s]	Height [mAU]	Area %
1	11.239	MM	0.4581	1.98556e4	722.39197	92.4450
2	16.203	MM	0.6947	1622.68018	38.92802	7.5550

*(R)-4-Bromo-[1,1'-binaphthalene]-2,2'-diol (R)-**1w***

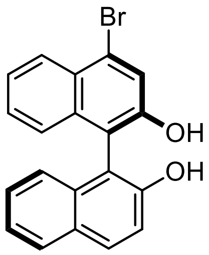

(*R*)-**1x**

According to *gerneral procedure B*, the reaction of *rac*-**1x** (0.1 mmol) under the catalysis of CuCl (0.001 mmol), ligand **L8** (0.001 mmol), and LPL-311-Celite (2.7 w/w) in the presenece of Na_2CO_3 (0.15 mmol) at 50 °C in toluene/vinyl acetate (1.0 mL:0.2 mL) for 60 h under an oxygen atmosphere (balloon, 1 atm) and hydrolysising afforded (*R*)-**1x** (24.5 mg, 67% yield, 90% *ee*): white solid. MP: 149−150 °C; $[\alpha]^{23}_{D}$ +20.2 (*c* = 0.36, $CHCl_3$); **^{1}H NMR** (400 MHz, Chloroform-*d*) δ 8.27 (d, *J* = 8.4 Hz, 1H), 7.90 (d, *J* = 8.9 Hz, 1H), 7.83 (d, *J* = 7.9 Hz, 1H), 7.71 (s, 1H), 7.47-7.41 (m, 1H), 7.36-7.25 (m, 4H), 7.11 (dd, *J* = 13.3, 8.4 Hz, 2H), 5.10 (s, 1H), 4.95 (s, 1H); **^{13}C NMR** (101 MHz, Chloroform-*d*) δ 152.59, 152.34, 134.05, 133.19, 131.62, 129.38, 128.40, 128.23, 128.11, 127.69, 127.63, 125.53, 125.32, 124.64, 124.15, 123.98, 121.94, 117.72, 111.17, 110.23. **HRMS (EI)** *m/z* calcd for $C_{20}H_{13}{}^{81}BrO_2$ [M^+]: 366.0075, found: 366.0068. **HPLC conditions**: CHIRALPAK OD-H column, 25 °C, wavelength = 250 nm, *n*-Hexane/*i*-PrOH = 90:10, flow rate = 1.0 mL/min, t_R = 9.55 min for major isomer, t_S = 16.52 min for minor isomer.

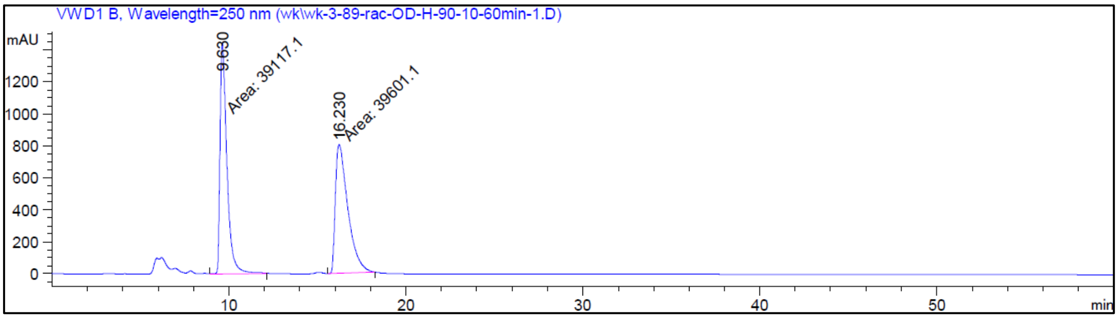

Peak #	RetTime [min]	Type	Width [min]	Area [mAU*s]	Height [mAU]	Area %
1	9.630	MM	0.4514	3.91171e4	1444.39270	49.6926
2	16.230	MM	0.8186	3.96011e4	806.30499	50.3074

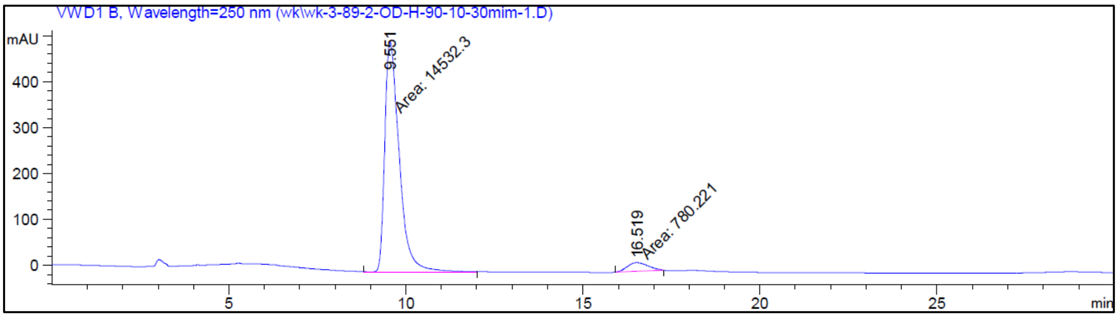

Peak #	RetTime [min]	Type	Width [min]	Area [mAU*s]	Height [mAU]	Area %
1	9.551	MM	0.4820	1.45323e4	502.53296	94.9047
2	16.519	MM	0.6782	780.22083	19.17422	5.0953

General procedure C for the one-pot oxidative coupling-DKR cascades to access C_2-symmetric BINOLs from 2-naphthols

*(R)-2'-Hydroxy-[1,1'-binaphthalen]-2-yl acetate (R)-**2a***

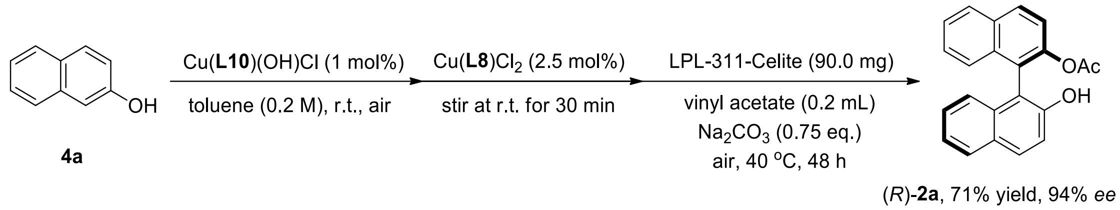

Under an air atmosphere, to a 10 mL tube charged with a stir bar were added **4a** (28.8 mg, 0.2 mmol), Cu(**L10**)(OH)Cl (0.5 mg, 0.002 mmol), and 1 mL of toluene sequentially. The reaction was placed under sonication in a water bath for 2 min, and continue to stir at room temperature until the complete consumption of **4a** as monitored by TLC. Cu(**L8**)Cl_2 (2.5 mg, 0.005 mmol) was then added. After the reaction mixture was placed under sonication in a water bath for 2 min and stir at room temperature for additional 30 min, lipase LPL-311-Celite (90.0 mg), Na_2CO_3 (15.9 mg, 0.15 mmol) and vinyl acetate (0.2 mL, 0.2 v/v) were sequentially added to the rection mixture. After that, the tube was sealed with a rubber septum and stirred at 40 °C for 48 h. After the reaction was complete, the mixture was filtrated through filter paper by using a buchner funnel. The residual Celite pad was washed with EtOAc. After removal of the solvent, the crude product was purified via flash column chromatography on silica gel (eluent: petroleum ether/ethyl ether = 10/1) afforded (*R*)-**2a** (23.4 mg, 71% yield, 94% *ee*): colorless oil. The optical purity of (*R*)-**2a** was determined by HPLC analysis on a CHIRALPAK OD-H column, 25 °C, wavelength = 250 nm, *n*-Hexane/*i*-PrOH = 90:10, flow rate = 1.0 mL/min, t_R = 7.65 min for major isomer, t_S = 8.95 min for minor isomer.

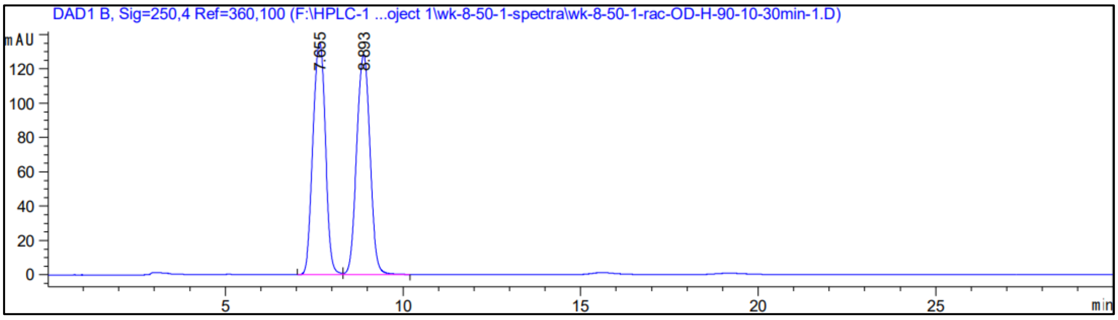

Peak #	RetTime [min]	Type	Width [min]	Area [mAU*s]	Height [mAU]	Area %
1	7.655	BV	0.3988	3318.99585	135.00343	49.7007
2	8.893	VB	0.4200	3358.97046	128.06451	50.2993

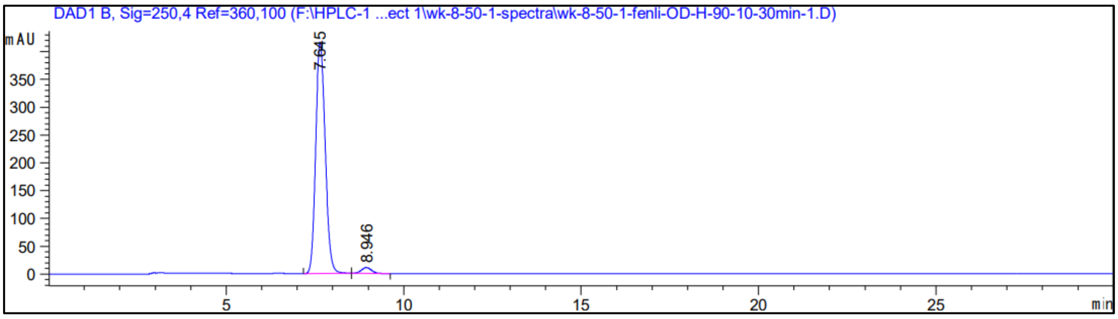

Peak #	RetTime [min]	Type	Width [min]	Area [mAU*s]	Height [mAU]	Area %
1	7.645	BB	0.2936	7753.88916	415.80313	97.3659
2	8.946	BB	0.3097	209.77344	10.56083	2.6341

General procedure D for one-pot oxidative coupling-DKR cascade for the synthesis of C_2-symmetric BINOLs

*(R)-2'-Hydroxy-6,6'-dimethyl-[1,1'-binaphthalen]-2-yl acetate (R)-**2b***

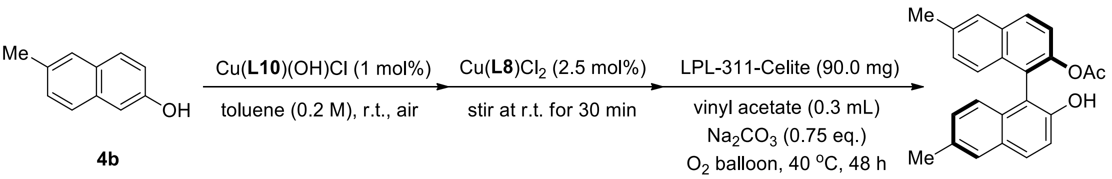

(*R*)-**2b**, 73% yield, 96% *ee*

Under an air atmosphere, to a 10 mL tube charged with a stir bar were added **4b** (31.6 mg, 0.2 mmol), Cu(**L10**)(OH)Cl (0.5 mg, 0.002 mmol), and 1 mL of toluene sequentially. The reaction was placed under sonication in a water bath for 2 min, and

continue to stir at room temperature until the complete consumption of the **4b** as monitored by TLC. Cu(**L8**)Cl_2 (2.5 mg, 0.005 mmol) was then added. After the reaction mixture was placed under sonication in a water bath for 2 min and stir at room temperature for additional 30 min, lipase LPL-311-Celite (90.0 mg), Na_2CO_3 (15.9 mg, 0.15 mmol) were sequentially added to the rection mixture. The tube was sealed with a rubber septum and replaced the inner atmosphere with oxygen, then injected vinly acetate (0.3 mL, 0.3 v/v) and stirred at 40 °C for 48 h. After the reaction was complete, the mixture was filtrated through filter paper by using a buchner funnel. The residual Celite pad was washed with EtOAc. After removal of the solvent, the crude product was purified via flash column chromatography on silica gel (eluent: petroleum ether/ethyl ether = 10/1) afforded (*R*)-**2b** (26.0 mg, 73% yield, 96% *ee*): colorless oil. The optical purity of (*R*)-**2b** was determined by HPLC analysis on a CHIRALPAK IC column, 25 °C, wavelength = 250 nm, *n*-Hexane/*i*-PrOH = 95:5, flow rate = 1.0 mL/min, t_R = 8.37 min for major isomer, t_S = 13.66 min for minor isomer.

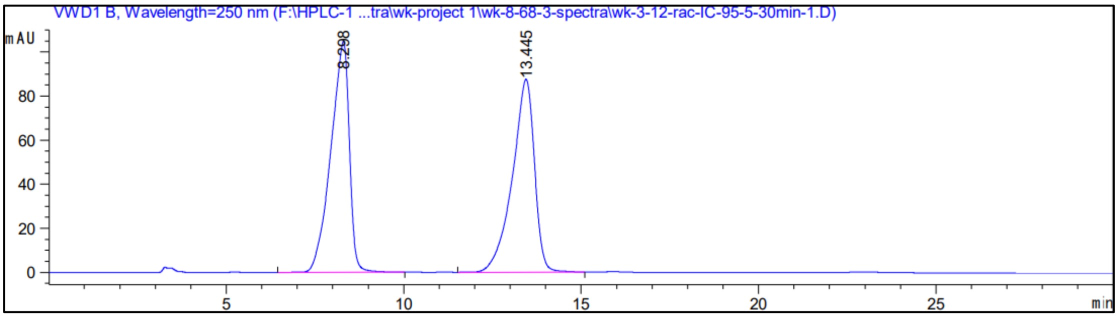

Peak #	RetTime [min]	Type	Width [min]	Area [mAU*s]	Height [mAU]	Area %
1	8.298	BB	0.5170	3798.43628	104.76004	49.9727
2	13.445	BB	0.6325	3802.58374	87.59206	50.0273

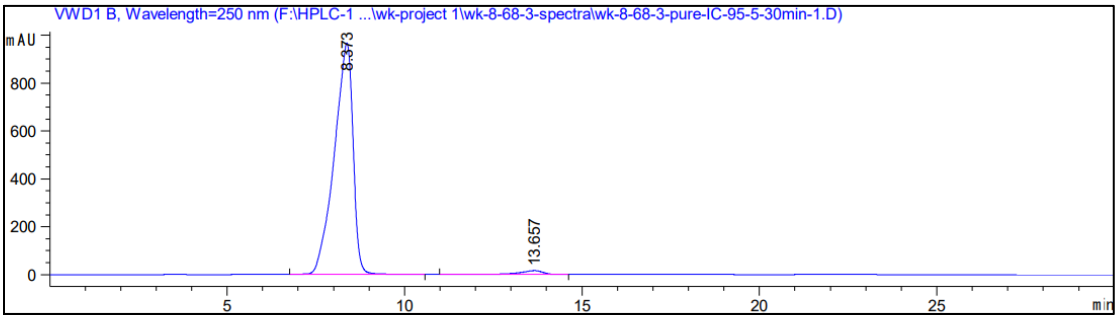

Peak #	RetTime [min]	Type	Width [min]	Area [mAU*s]	Height [mAU]	Area %
1	8.373	BB	0.5280	3.54422e4	966.18768	97.9220
2	13.657	BB	0.6663	752.10461	16.23987	2.0780

*(R)-6,6'-Diethyl-2'-hydroxy-[1,1'-binaphthalen]-2-yl acetate (R)-**2c***

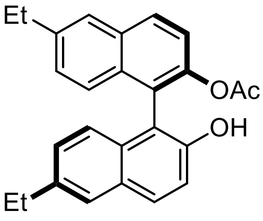

(*R*)-**2c**

According to *gerneral procedure D*, the reaction of **4c** (34.4 mg, 0.2 mmol) under the catalysis of Cu(**L10**)(OH)Cl (0.5 mg, 0.002 mmol), Cu(**L8**)Cl_2 (2.5 mg, 0.005 mmol), and LPL-311-Celite (90.0 mg) in the presenece of Na_2CO_3 (15.9 mg, 0.15 mmol) at 40 °C in toluene/vinyl acetate (1.0 mL:0.3 mL) for 48 h under an O_2 atmosphere afforded (*R*)-**2c** (26.8 mg, 70% yield, 87% *ee*): colorless oil. **HPLC conditions**: CHIRALPAK AD-H column, 25 °C, wavelength = 250 nm, *n*-Hexane/*i*-PrOH = 90:10, flow rate = 1.0 mL/min, t_R = 13.29 min for major isomer, t_S = 15.88 min for minor isomer.

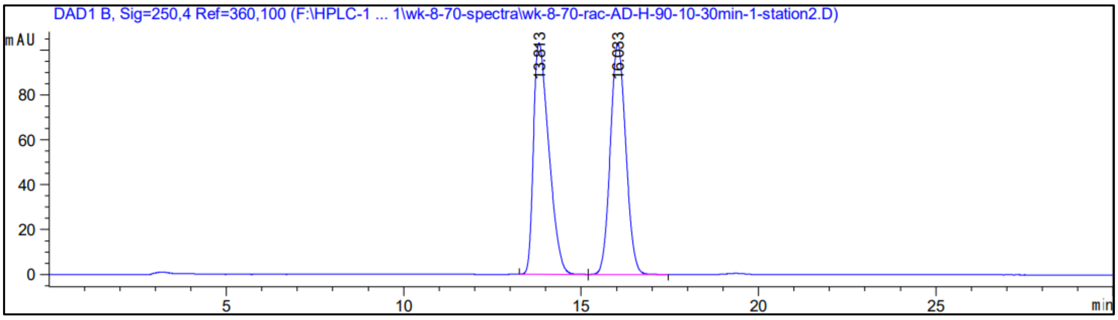

Peak #	RetTime [min]	Type	Width [min]	Area [mAU*s]	Height [mAU]	Area %
1	13.813	BB	0.4636	3109.71948	103.17303	49.9521
2	16.033	BB	0.4730	3115.68750	102.98697	50.0479

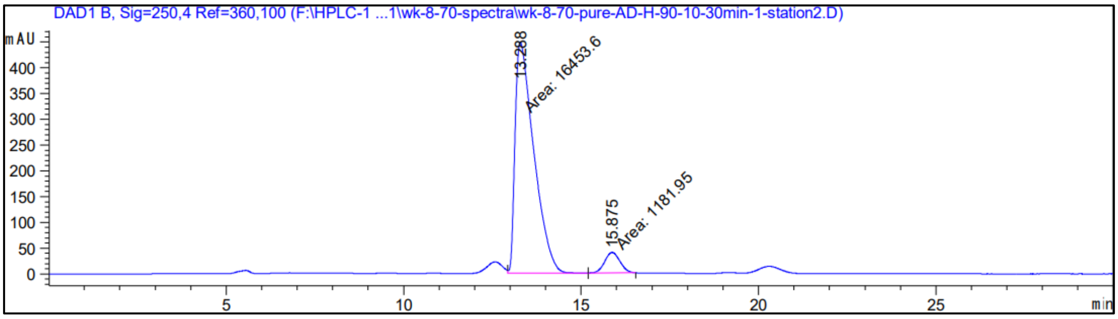

Peak #	RetTime [min]	Type	Width [min]	Area [mAU*s]	Height [mAU]	Area %
1	13.288	FM	0.6110	1.64536e4	448.83377	93.2979
2	15.875	MM	0.4969	1181.94751	39.64056	6.7021

*(R)-6,6'-Dibromo-2'-hydroxy-[1,1'-binaphthalen]-2-yl acetate (R)-**2d***

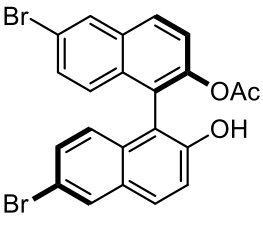

(*R*)-**2d**

According to *gerneral procedure D*, the reaction of **4d** (44.6 mg, 0.2 mmol) under the catalysis of Cu(**L10**)(OH)Cl (0.5 mg, 0.002 mmol), Cu(**L8**)Cl_2 (2.5 mg, 0.005 mmol), and LPL-311-Celite (90.0 mg) in the presenece of Na_2CO_3 (15.9 mg, 0.15 mmol) at 40 °C in toluene/vinyl acetate (1.0 mL:0.3 mL) for 48 h under an O_2 atmosphere afforded (*R*)-**2d** (35.9 mg, 74% yield, 90% *ee*): colorless oil. **HPLC conditions**: CHIRALPAK AD-H column, 25 °C, wavelength = 250 nm, *n*-Hexane/*i*-PrOH = 90:10, flow rate = 1.0 mL/min, t_R = 17.40 min for major isomer, t_S = 13.83 min for minor isomer.

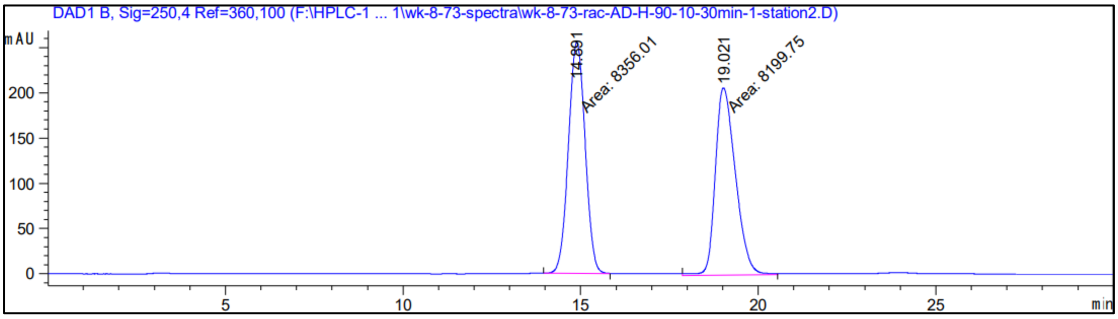

Peak #	RetTime [min]	Type	Width [min]	Area [mAU*s]	Height [mAU]	Area %
1	14.891	MM	0.5449	8356.01172	255.59442	50.4719
2	19.021	MM	0.6603	8199.75195	206.97089	49.5281

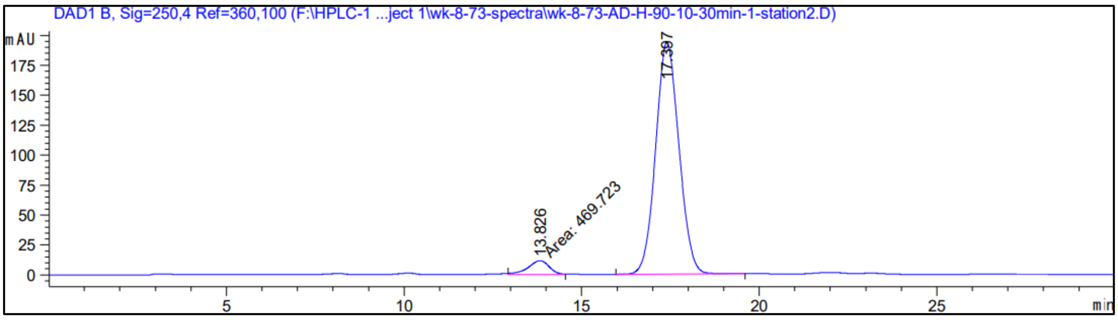

Peak #	RetTime [min]	Type	Width [min]	Area [mAU*s]	Height [mAU]	Area %
1	13.826	MF	0.6836	469.72324	11.45214	5.0543
2	17.397	BB	0.7065	8823.79395	193.32536	94.9457

*(R)-2'-Hydroxy-6,6'-dimethoxy-[1,1'-binaphthalen]-2-yl acetate (R)-**2e***

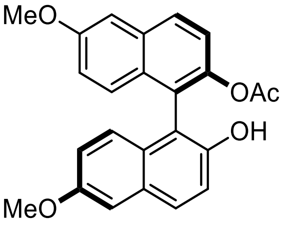

(*R*)-**2e**

According to *gerneral procedure C*, the reaction of **4e** (34.8 mg, 0.2 mmol) under the catalysis of Cu(**L10**)(OH)Cl (0.5 mg, 0.002 mmol), Cu(**L8**)Cl_2 (2.5 mg, 0.005 mmol), and LPL-311-Celite (90.0 mg) in the presenece of Na_2CO_3 (10.6 mg, 0.1 mmol) at 40 °C in toluene/vinyl acetate (1.0 mL:0.2 mL) for 48 h under an air atmosphere afforded (*R*)-**2e** (32.0 mg, 82% yield, 84% *ee*): colorless oil. **HPLC conditions**: CHIRALPAK AD-H column, 25 °C, wavelength = 250 nm, *n*-Hexane/*i*-PrOH = 90:10, flow rate = 1.0 mL/min, t_R = 32.65 min for major isomer, t_S = 44.03 min for minor isomer.

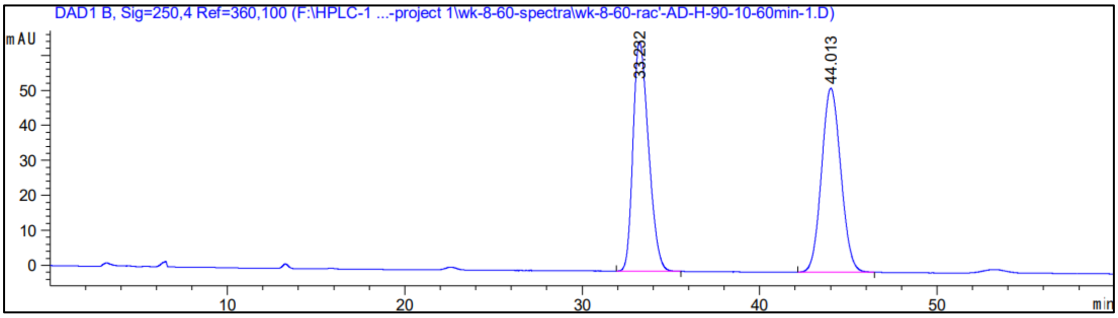

Peak #	RetTime [min]	Type	Width [min]	Area [mAU*s]	Height [mAU]	Area %
1	33.232	BB	0.9328	3989.27759	65.47949	50.1007
2	44.013	BB	1.1818	3973.23853	52.56976	49.8993

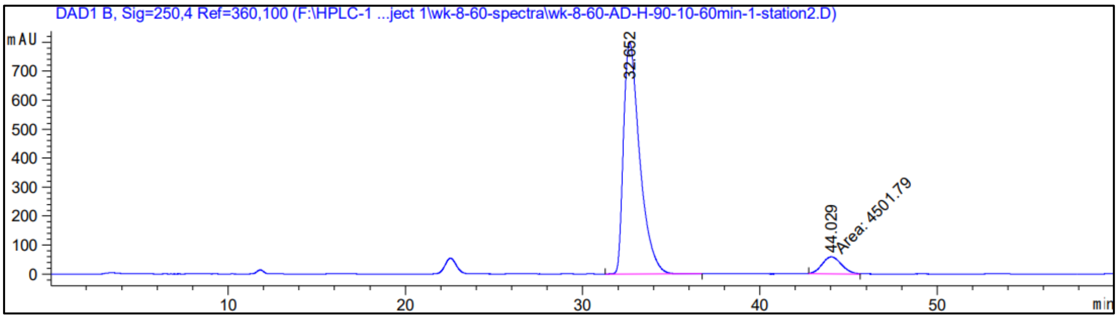

Peak #	RetTime [min]	Type	Width [min]	Area [mAU*s]	Height [mAU]	Area %
1	32.652	BB	0.9711	5.08760e4	798.70551	91.8708
2	44.029	FM	1.2718	4501.79150	58.99727	8.1292

*(R)-2'-Hydroxy-7,7'-dimethoxy-[1,1'-binaphthalen]-2-yl acetate (R)-**2g***

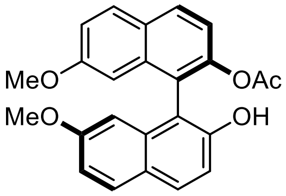

(*R*)-**2g**

According to *gerneral procedure D*, the reaction of **4g** (34.8 mg, 0.2 mmol) under the catalysis of Cu(**L10**)(OH)Cl (0.5 mg, 0.002 mmol), Cu(**L8**)Cl_2 (2.5 mg, 0.005 mmol), and LPL-311-Celite (90.0 mg) in the presenece of Na_2CO_3 (15.9 mg, 0.15 mmol) at 40 °C in toluene/vinyl acetate (1.0 mL:0.3 mL) for 48 h under an O_2 atmosphere afforded (*R*)-**2g** (17.3 mg, 45% yield, 88% *ee*): colorless oil. **HPLC conditions**: CHIRALPAK IC column, 25 °C, wavelength = 250 nm, *n*-Hexane/*i*-PrOH = 95:5, flow

rate = 1.0 mL/min, t_R = 9.93 min for major isomer, t_S = 15.43 min for minor isomer.

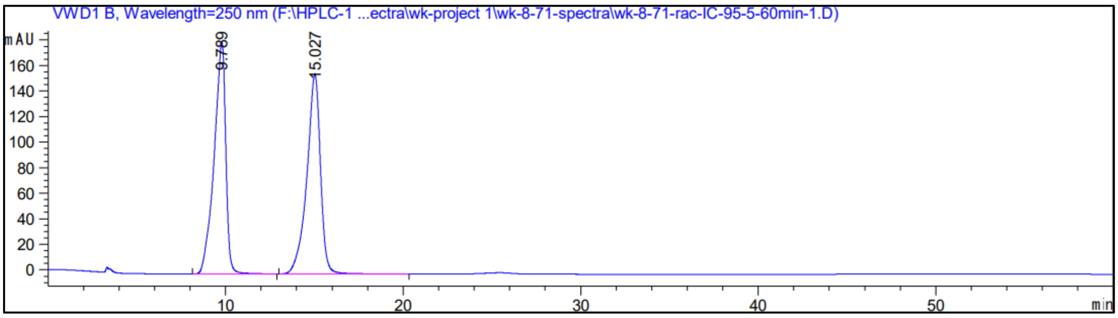

Peak #	RetTime [min]	Type	Width [min]	Area [mAU*s]	Height [mAU]	Area %
1	9.789	BB	0.6530	8254.18750	181.34529	49.7224
2	15.027	BB	0.7817	8346.35938	156.61748	50.2776

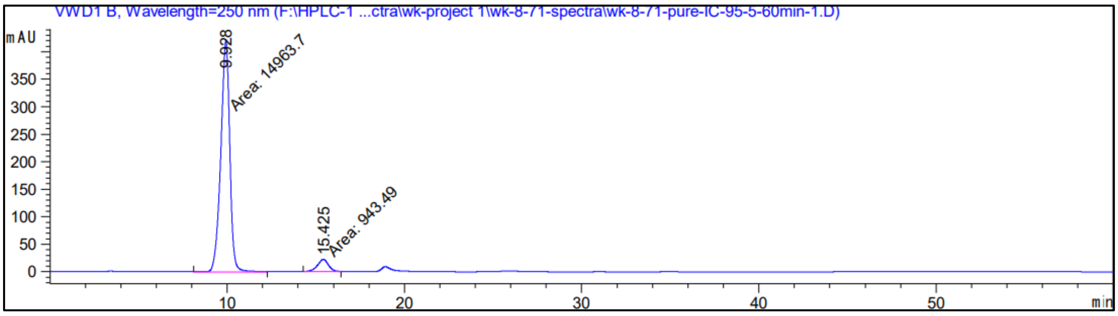

Peak #	RetTime [min]	Type	Width [min]	Area [mAU*s]	Height [mAU]	Area %
1	9.928	MM	0.5920	1.49637e4	421.26425	94.0688
2	15.425	MM	0.7195	943.48962	21.85383	5.9312

*(R)-2'-Hydroxy-7,7'-dimethyl-[1,1'-binaphthalen]-2-yl acetate (R)-***2y**

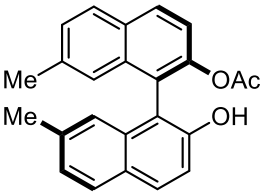

(*R*)-**2y**

According to *gerneral procedure D*, the reaction of **4y** (31.6 mg, 0.2 mmol) under the catalysis of Cu(**L10**)(OH)Cl (0.5 mg, 0.002 mmol), Cu(**L8**)Cl_2 (2.5 mg, 0.005 mmol), and LPL-311-Celite (90.0 mg) in the presenece of Na_2CO_3 (15.9 mg, 0.15 mmol) at 40 °C in toluene/vinyl acetate (1.0 mL:0.3 mL) for 48 h under an O_2 atmosphere afforded (*R*)-**2y** (21.0 mg, 59% yield, 92% *ee*): colorless oil. **^{1}H NMR** (400 MHz,

Chloroform-*d*) δ 8.01 (d, *J* = 8.8 Hz, 1H), 7.85 (dd, *J* = 8.6, 4.9 Hz, 2H), 7.74 (d, *J* = 8.3 Hz, 1H), 7.36 – 7.28 (m, 2H), 7.25 (d, *J* = 8.9 Hz, 1H), 7.16 (d, *J* = 8.3 Hz, 1H), 7.03 (s, 1H), 6.80 (s, 1H), 5.13 (s, 1H), 2.28 (s, 3H), 2.26 (s, 3H), 1.81 (s, 3H). **^{13}C NMR** (101 MHz, Chloroform-*d*) δ 170.39, 151.72, 148.17, 137.43, 136.39, 133.70, 133.68, 130.48, 130.40, 129.96, 128.60, 128.09, 127.78, 127.22, 125.77, 124.55, 123.57, 122.50, 120.76, 117.21, 113.68, 21.87, 21.85, 20.36. **HRMS (EI)** *m/z* calcd for $C_{24}H_{20}O_3$ [M^+]: 356.1407, found: 356.1407. **HPLC conditions**: CHIRALPAK IC column, 25 °C, wavelength = 250 nm, *n*-Hexane/*i*-PrOH = 90:10, flow rate = 1.0 mL/min, t_R = 5.00 min for major isomer, t_S = 7.26 min for minor isomer.

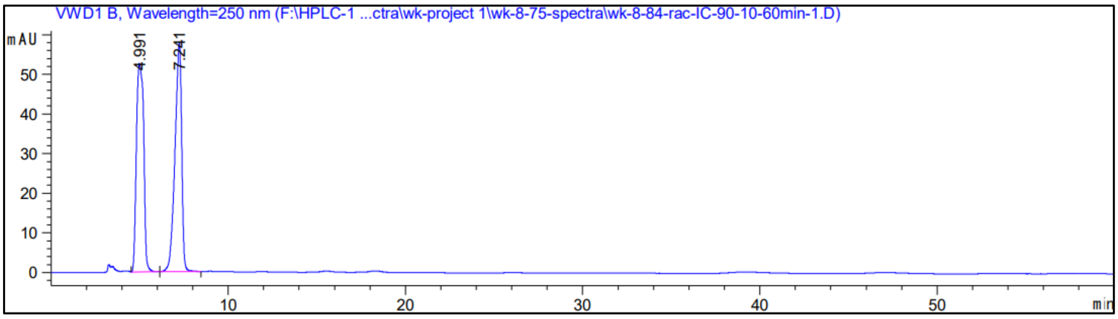

Peak #	RetTime [min]	Type	Width [min]	Area [mAU*s]	Height [mAU]	Area %
1	4.991	BB	0.4089	1500.17590	52.57383	49.5498
2	7.241	BB	0.3978	1527.43811	57.89241	50.4502

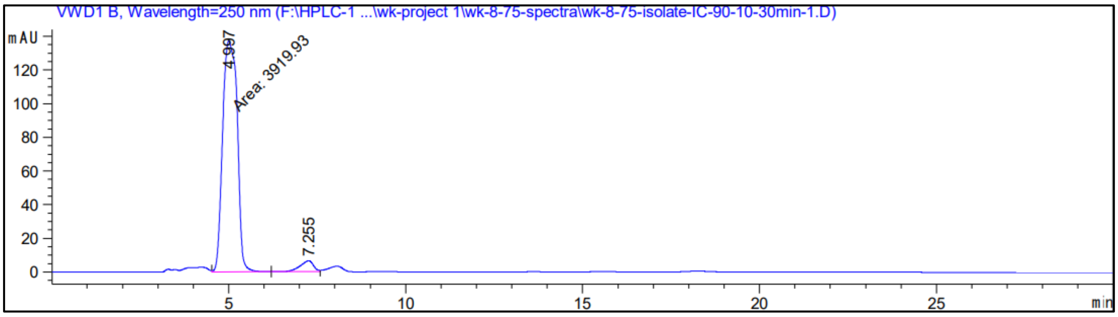

Peak #	RetTime [min]	Type	Width [min]	Area [mAU*s]	Height [mAU]	Area %
1	4.997	FM	0.4763	3919.93140	137.15897	95.7548
2	7.255	BV	0.3899	173.78487	6.54261	4.2452

Mechanistic Studies

Comparison experiments

Table S7. Racemization of (*R*)-**1a** by copper catalysis

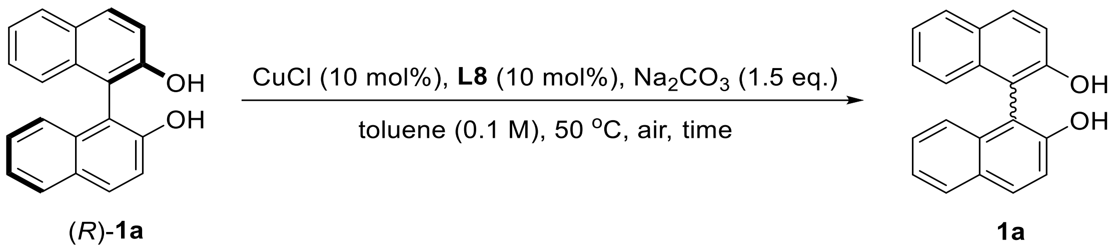

Entry	Time	*ee* (%) of **1a**
1	0 min	> 99
2	30 min	90
3	60 min	70
4	90 min	51
5	120 min	37
6	150 min	27
7	180 min	18
8	210 min	11
9	240 min	5
10	270 min	0

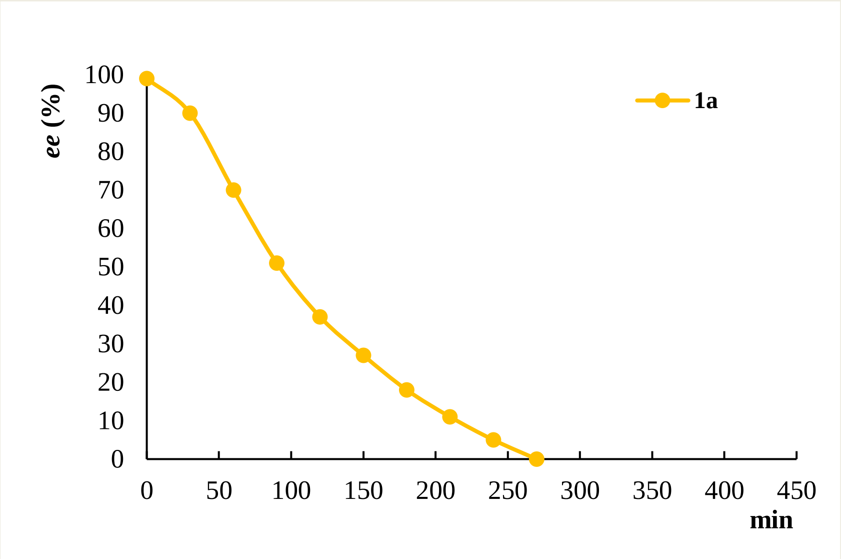

Figure S1. Racemization rate curve of (*R*)-**1a**

Because the racemization reaction almost stopped after 270 min, data after 270 min was not counted for the calculation of k_{rac}.

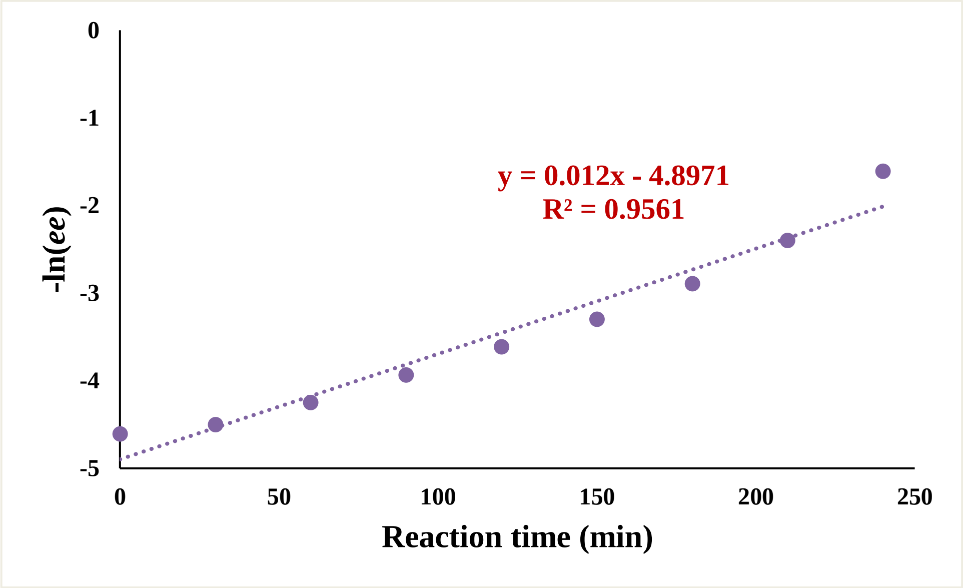

Figure S2. Studies on the racemization kinetics of (*R*)-**1a**

$k = 0.012\ \text{min}^{-1}$, $k_{rac} = k/2 = 0.006\ \text{min}^{-1} = 0.0001\ \text{s}^{-1}$

$\Delta G_{rac}^{323\ \text{K}} = -RT \bullet \ln(k_{rac} \cdot h/k_B \cdot T) = 24.9$ kcal/mol.

Table S8. Racemization of (*R*)-**1d** by copper catalysis

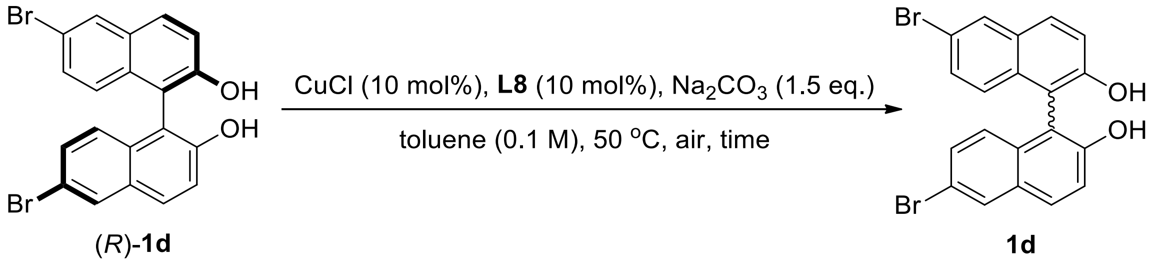

Entry	Time	*ee* (%) of **1d**
1	0 min	99
2	20 min	98
3	40 min	97
4	60 min	96
5	80 min	94
6	100 min	93
7	120 min	91
8	140 min	90
9	160 min	88
10	180 min	86
11	300 min	69
12	360 min	53
13	420 min	32

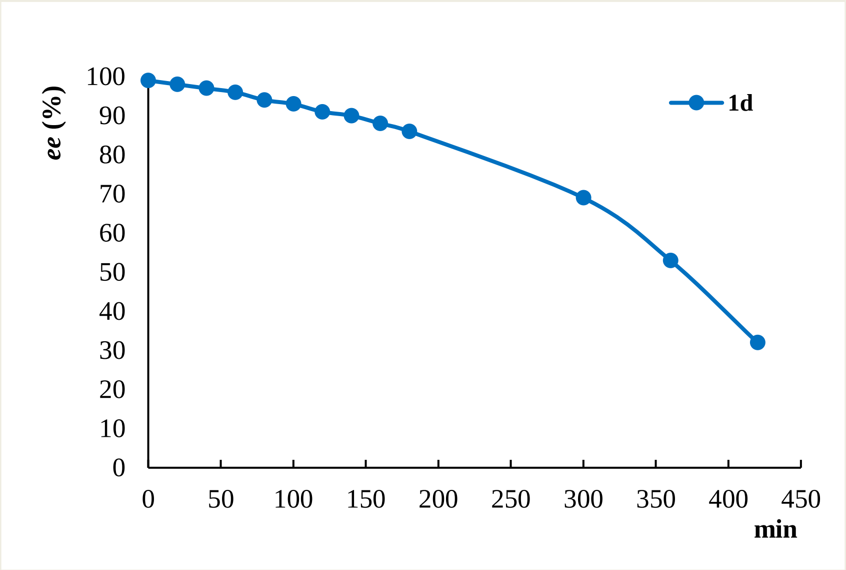

Figure S3. Racemization rate curve of (*R*)-**1d**

Table S9. Racemization of (*R*)-**1e** by copper catalysis

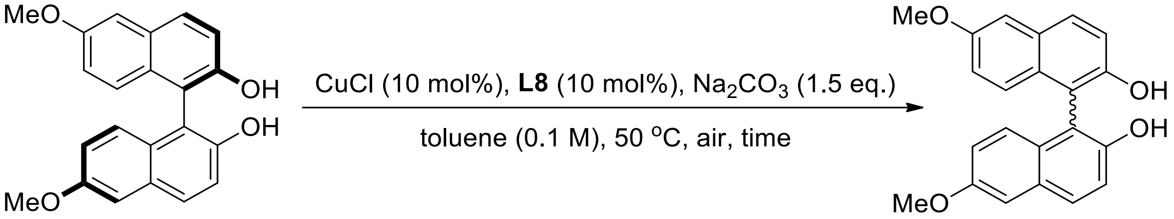

Entry	Time	*ee* (%) of **1e**
1	0 min	94
2	20 min	7
3	40 min	2
4	60 min	0
5	80 min	0
6	100 min	0
7	120 min	0
8	140 min	0
9	160 min	0

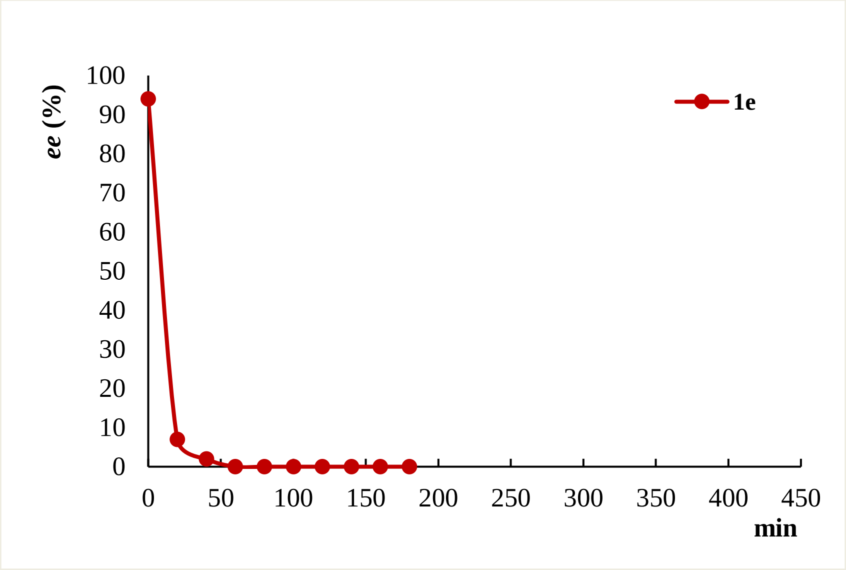

Figure S4. Racemization rate curve of (*R*)-**1e**

Table S10. Control experiments

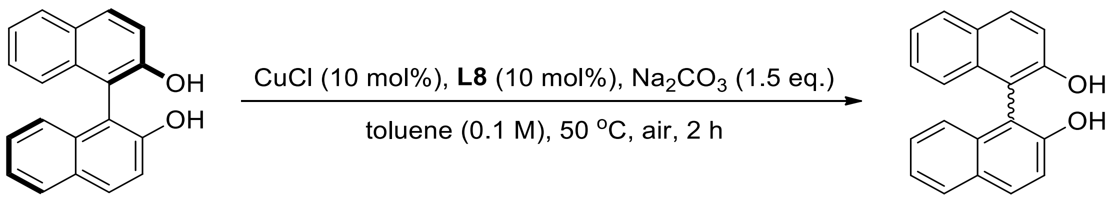

(*R*)-**1a**, >99% *ee* **1a**

Entry	Deviations	*ee* (%) of **1a**
1	none	8
2	TEMPO (3 equiv)	85
3	BHT (2 equiv)	98
4	N_2	98
5	Cu(**L8**)Cl_2	3
6	Cu(**L8**)Cl_2, inert conditions	58
7	Cu(**L9**)Cl_2	<1
8	Cu(**L10**)Cl_2	30

Table S11. Studies on the compatibility of individual copper catalyst with lipase[a]

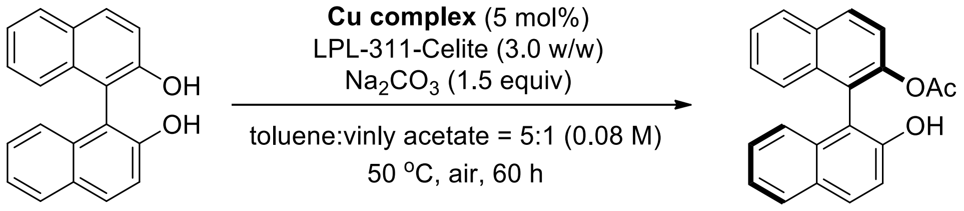

rac-**1a** (*R*)-**2a**

**Cu complex**	Yield[b] and *ee* of (*R*)-**2a**	Yield[b] and *ee* of recovery **1a**	Compatible with lipase
Cu(**L8**)Cl_2	90% yield, 94% *ee*	<5% yield, <5% *ee*	√
Cu(**L9**)Cl_2	52% yield, 80% *ee*	42% yield, 99% *ee*	×
Cu(**L10**)Cl_2	56% yield, 67% *ee*	34% yield, 99% *ee*	×

[a]The reaction was carried out using *rac*-**1a** (c=0.08 M), Cu complex (5 mol%), LPL-311-Celite (3 w/w) and Na_2CO_3 (1.5 equiv) at 50 °C in toluene/acyl donor (1.0 mL:0.2 mL) for 60 h under an air atmosphere. [b]Determined by ^1H NMR analysis with CH_2Br_2 as the internal standard.

UV-Vis absorption spectroscopy

Experimental procedure

Solutions of TMEDA (0.2 mM in $CHCl_3$), Cu(**L10**)Cl_2 (0.2 mM in $CHCl_3$), **L8** (BCP) (0.2 mM in $CHCl_3$), Cu(**L8**)Cl_2 (0.2 mM in $CHCl_3$), and mixture of Cu(**L10**)Cl_2 with **L8** (0.2 mM in $CHCl_3$) were prepared respectively. 3 mL of each solution was used for the measurement. All measurements were performed at room temperature.

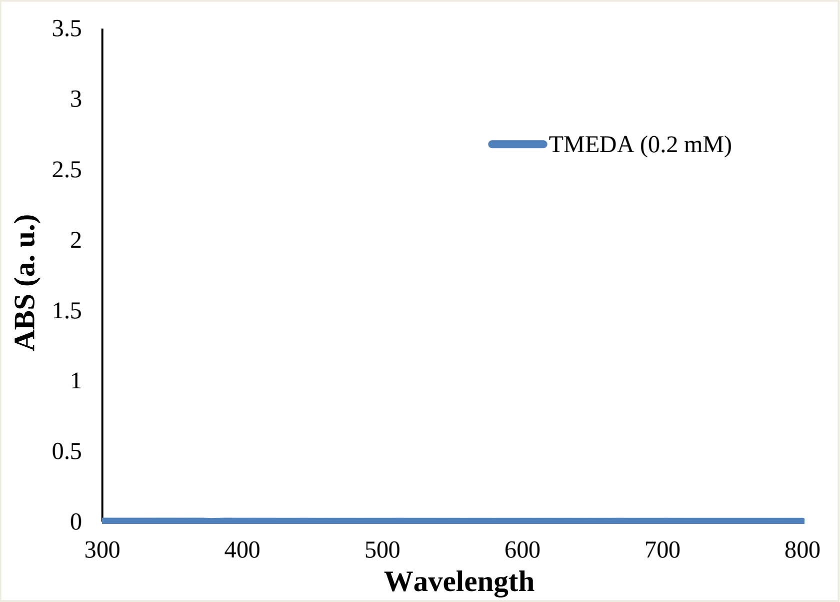

Figure S5. UV-Vis absorption spectrum of TMEDA

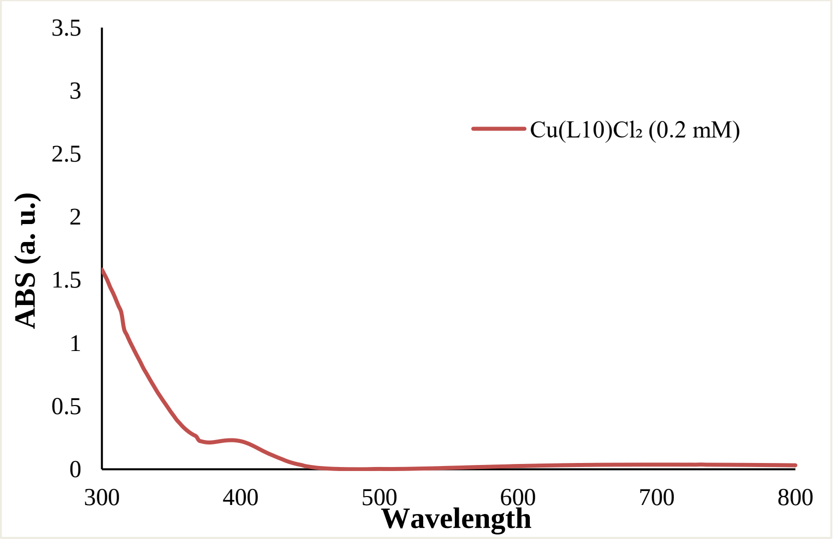

Figure S6. UV-Vis absorption spectrum of Cu(**L10**)Cl_2

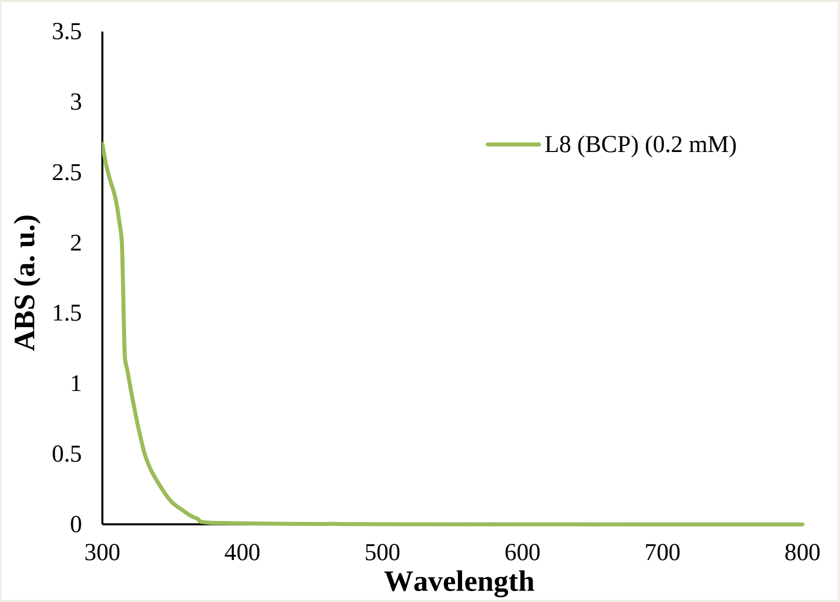

Figure S7. UV-Vis absorption spectrum of **L8** (BCP)

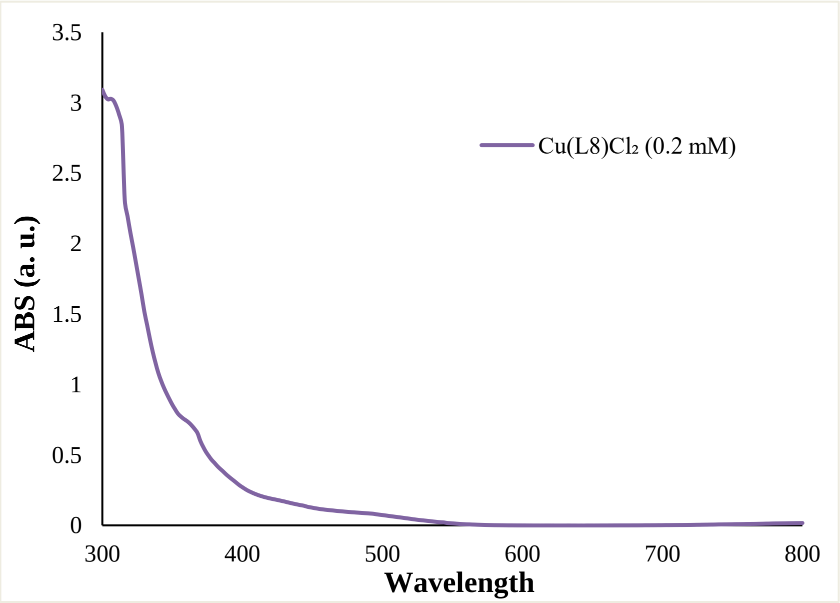

Figure S8. UV-Vis absorption spectrum of Cu(**L8**)Cl_2

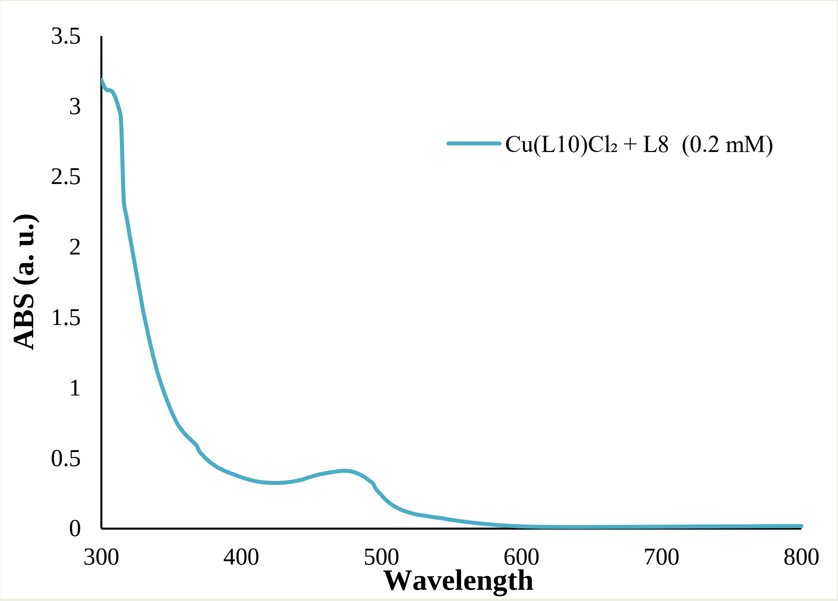

Figure S9. UV-Vis absorption spectrum of Cu(**L10**)Cl_2 with **L8**

Equation S2. Intramolecular oxidative C-O bond formation

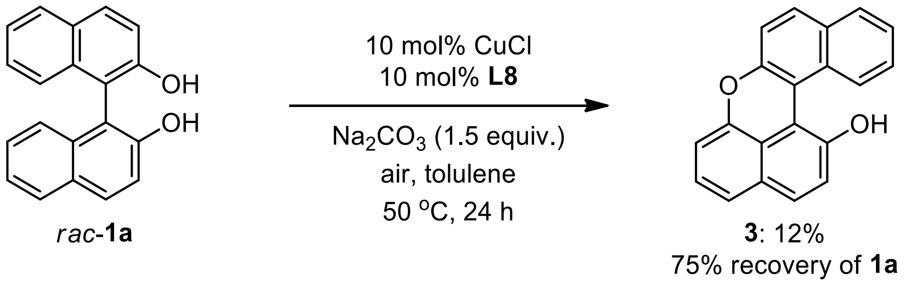

Under an air atmosphere, to a 10 mL tube charged with a stir bar were added CuCl (0.02 mmol), ligand **L8** (0.02 mmol) and 2 mL of toluene sequentially. The reaction tube was placed under sonication in a water bath for 5 min and then let the mixture continue to stir at room temperature for additional 10 min. *Rac*-**1a** (0.2 mmol) and Na_2CO_3 (0.3 mmol) were sequentially added to the reaction mixture. Immediately, the tube was stirred at 50 °C for 24 h. After the reaction was complete, the mixture was quenched by adding 4 mL 1.5 M HCl and poured into a 25 mL separatory funnel. The mixture was extracted with 3×10 mL EtOAc and dried over anhydrous Na_2SO_4. After removal of the solvent, the crude product was purified via flash column

chromatography on silica gel (eluent: petroleum ether/ethyl ether = 10/1) afforded **3** (12% yield determined by ^{1}H NMR) and recovery 72% yield (determined by ^{1}H NMR) of *rac*-**1a**. Spectra data for isolated **3**: ^{1}**H NMR** (400 MHz, DMSO-d_6) δ 10.18 (s, 1H), 7.89 (t, J = 8.7 Hz, 3H), 7.66 (d, J = 8.9 Hz, 1H), 7.45 (dt, J = 14.7, 6.5 Hz, 2H), 7.41-7.34 (m, 2H), 7.29 (d, J = 8.9 Hz, 1H), 7.21 (t, J = 7.8 Hz, 1H), 6.93 (d, J = 7.5 Hz, 1H); ^{13}**C NMR** (101 MHz, DMSO-d_6) δ 152.52, 149.63, 148.62, 130.91, 130.05, 129.10, 128.78, 127.62, 127.46, 126.99, 126.08, 124.75, 124.20, 121.76, 120.00, 116.79, 114.36, 108.28, 107.66.

Table S12. Racemization of (*S*)-**1h'** by copper catalysis

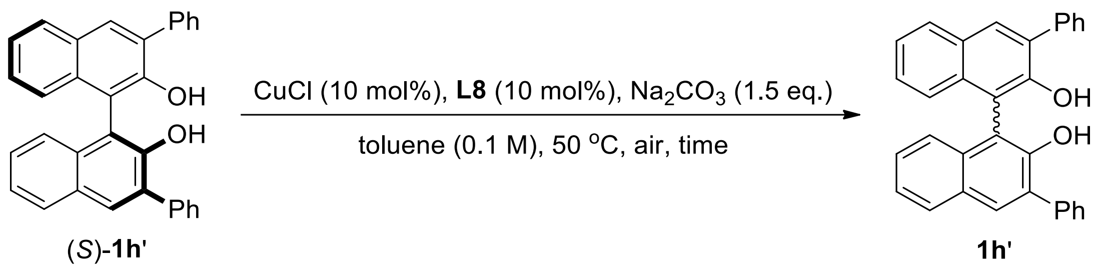

Entry	Time	*ee* (%) of **1h'**
1	0 min	99
2	60 min	70
3	120 min	55
4	180 min	36
5	240 min	20

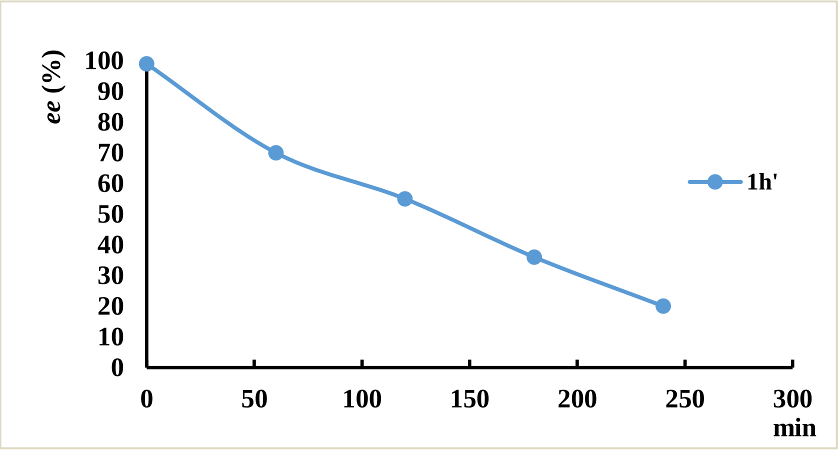

Figure S10. Racemization rate curve of (*S*)-**1h'**

Table S13. Racemization of (*S*)-**1h'** by **[Ru]-2**

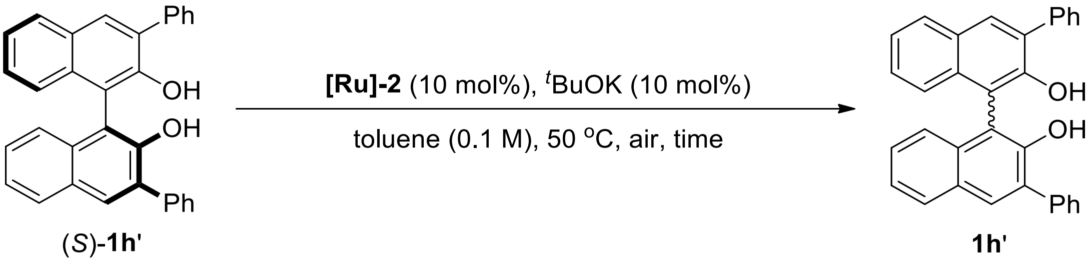

Entry	Time	*ee* (%) of **1h'**
1	0 min	99
2	240 min	96

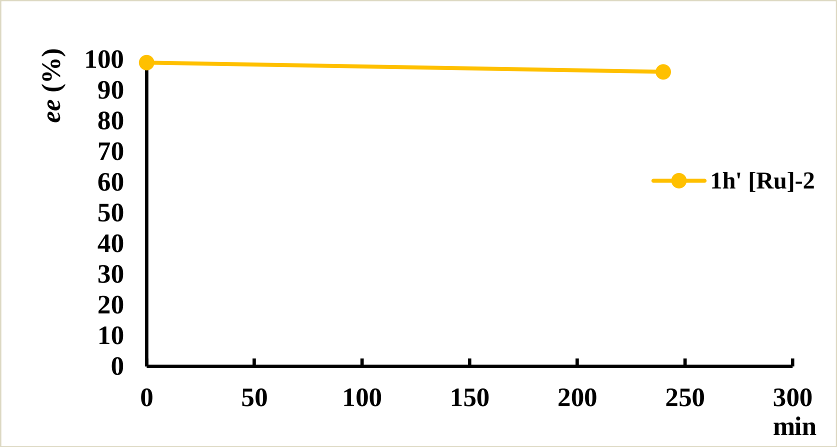

Figure S11. Racemization rate curve of (*S*)-**1h'** catalyzed by **[Ru]-2**

Gerneral procedure E for dynamic classical resolution of 1a, 1d and 1h’ with CuCl/L8 and chiral ammonium salt

*(R)-[1,1'-binaphthalene]-2,2'-diol (R)-**1a***

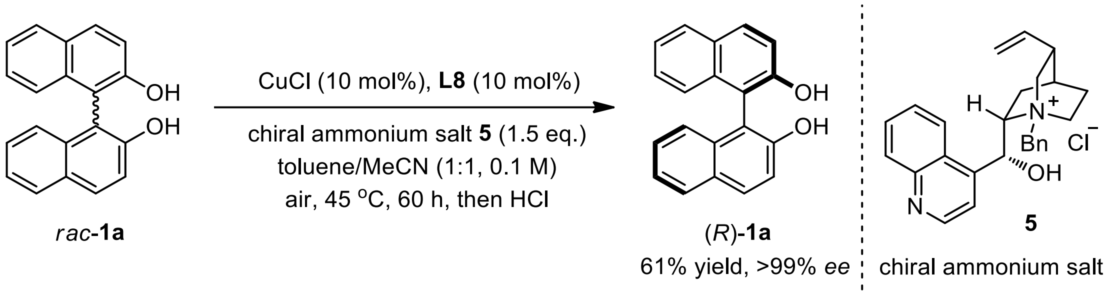

Under an air atmosphere, to a 10 mL tube charged with a stir bar were added CuCl (4.9 mg, 0.05 mmol), ligand **L8** (18.0 mg, 0.05 mmol) and 2.5 mL of toluene sequentially. The reaction tube was placed under sonication in a water bath for 2 min, and then let the mixture continue to stir at room temperature for additional 10 min. *Rac*-**1a** (143.2 mg, 0.50 mmol), chiral ammonium salt **5** (315.7 mg, 0.75 mmol) and 2.5 mL of MeCN were sequentially added to the rection mixture. After that, the tube was sealed with a rubber septum and stirred at 45 °C for 60 h. The resulting precipitate was isolated through filter paper by using a Buchner funnel and washed with cold MeCN (2 mL, 0 °C). The precipitated complex was fully dissolved in a mixture of HCl (aq., 1 M, 20 mL) and EtOAc (20 mL). The organic phase was separated, and the aqueous phase was further extracted with EtOAc (20 mL × 2). The combined organic phases were dried over anhydrous Na_2SO_4 and concentrated under reduced pressure to afford (*R*)-**1a** (87.2 mg, 61% yield, >99% *ee*): white solid. **HPLC conditions**: CHIRALPAK OD-H column, 25 °C, wavelength = 250 nm, *n*-Hexane/*i*-PrOH = 90:10, flow rate = 1.0 mL/min, t_R = 17.66 min for major isomer, t_S = 15.35 min for minor isomer.

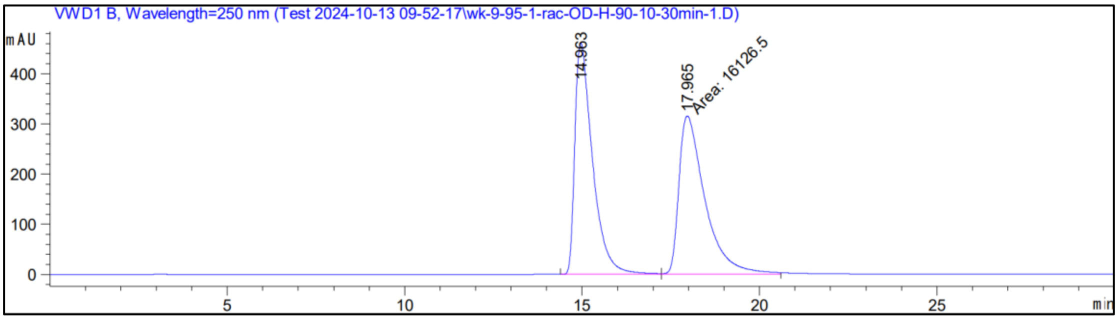

Peak #	RetTime [min]	Type	Width [min]	Area [mAU*s]	Height [mAU]	Area %
1	14.963	BV	0.5223	1.60635e4	461.26132	49.9021
2	17.965	MF	0.8517	1.61265e4	315.57840	50.0979

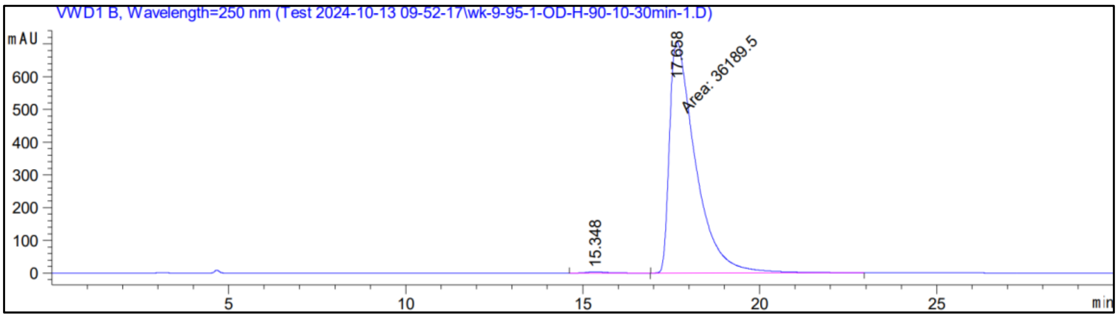

Peak #	RetTime [min]	Type	Width [min]	Area [mAU*s]	Height [mAU]	Area %
1	15.348	BB	0.5555	158.79651	4.41584	0.4369
2	17.658	MF	0.8548	3.61895e4	705.59619	99.5631

*(R)-6,6'-dibromo-[1,1'-binaphthalene]-2,2'-diol (R)-**1d***

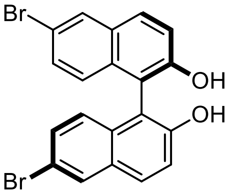

(*R*)-**1d**

According to *gerneral procedure E*, the reaction of *rac*-**1d** (0.5 mmol) and chiral ammonium salt **5** (0.75 mmol), under the catalysis of CuCl (0.05 mmol) and ligand **L8** (0.05 mmol) at 45 ºC in toluene/MeCN (2.5 mL:2.5 mL) for 60 h under an air atmosphere and hydrolysising afforded (*R*)-**1d** (141.7 mg, 64% yield, >99% *ee*): white solid. **HPLC conditions**: CHIRALPAK OD-H column, 25 ºC, wavelength = 250 nm, *n*-Hexane/*i*-PrOH = 90:10, flow rate = 1.0 mL/min, t_R = 39.14 min for major isomer, t_S = 15.59 min for minor isomer.

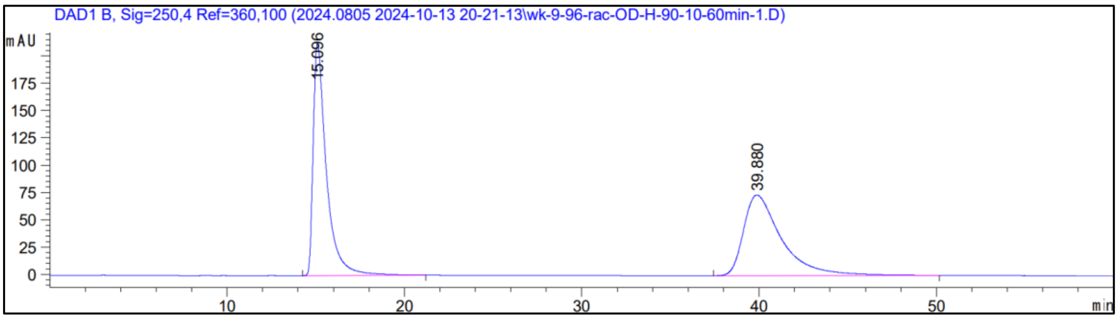

Peak #	RetTime [min]	Type	Width [min]	Area [mAU*s]	Height [mAU]	Area %
1	15.096	BB	0.7656	1.10164e4	212.22470	50.0557
2	39.880	BB	2.1397	1.09919e4	73.96838	49.9443

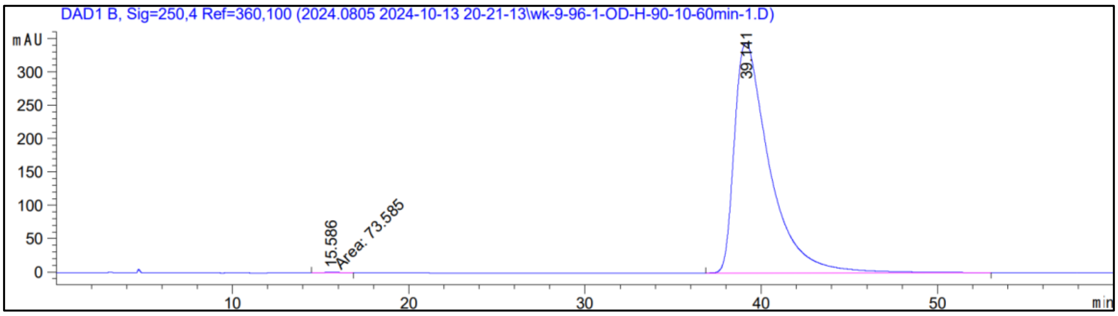

Peak #	RetTime [min]	Type	Width [min]	Area [mAU*s]	Height [mAU]	Area %
1	15.586	MM	0.9748	73.58503	1.25806	0.1538
2	39.141	BB	2.0043	4.77647e4	344.76328	99.8462

*(R)-3,3'-diphenyl-[1,1'-binaphthalene]-2,2'-diol (R)-**1h**'*

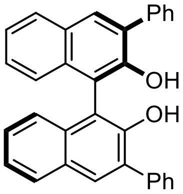

(*R*)-**1h'**

According to *gerneral procedure E*, the reaction of *rac*-**1h**' (0.5 mmol) and chiral ammonium salt **5** (0.75 mmol), under the catalysis of CuCl (0.05 mmol) and ligand **L8** (0.05 mmol) at 45 ºC in toluene/MeCN (2.5 mL:2.5 mL) for 60 h under an air atmosphere and hydrolysising afforded (*R*)-**1h**' (157.9 mg, 72% yield, >99% *ee*): light yellow solid. **^{1}H NMR** (400 MHz, Chloroform-*d*) δ 8.00 (s, 2H), 7.89 (d, *J* = 8.0 Hz, 2H), 7.72 (d, *J* = 7.3 Hz, 4H), 7.46 (t, *J* = 7.5 Hz, 4H), 7.37 (q, *J* = 7.7 Hz, 4H), 7.29 (t, *J* = 7.5 Hz, 2H), 7.22 (d, *J* = 8.3 Hz, 2H), 5.34 (s, 2H). **^{13}C NMR** (101 MHz, Chloroform-*d*) δ 150.11, 137.45, 132.94, 131.35, 130.66, 129.57, 129.42, 128.44, 128.42, 127.73, 127.30, 124.29, 124.25, 112.41. **HPLC conditions**: CHIRALPAK AD-H column, 25 ºC, wavelength = 250 nm, *n*-Hexane/*i*-PrOH = 90:10, flow rate = 1.0 mL/min, t_R = 33.94 min for major isomer, t_S = 30.88 min for minor isomer.

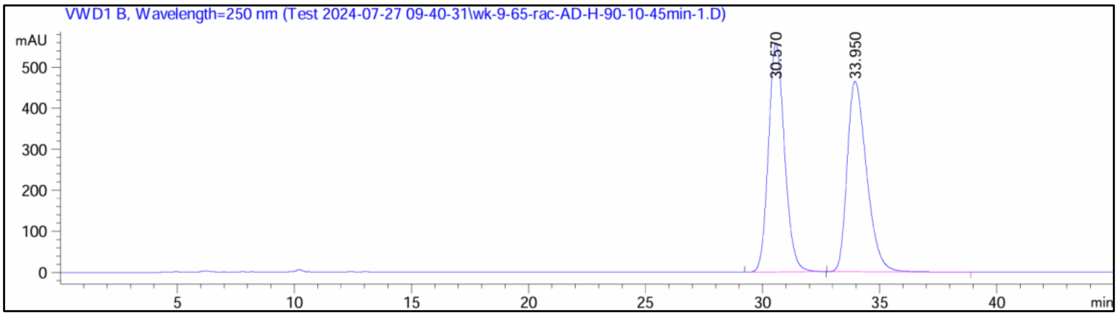

Peak #	RetTime [min]	Type	Width [min]	Area [mAU*s]	Height [mAU]	Area %
1	30.570	BB	0.7378	2.67135e4	558.70129	49.9312
2	33.950	BB	0.8885	2.67871e4	464.71814	50.0688

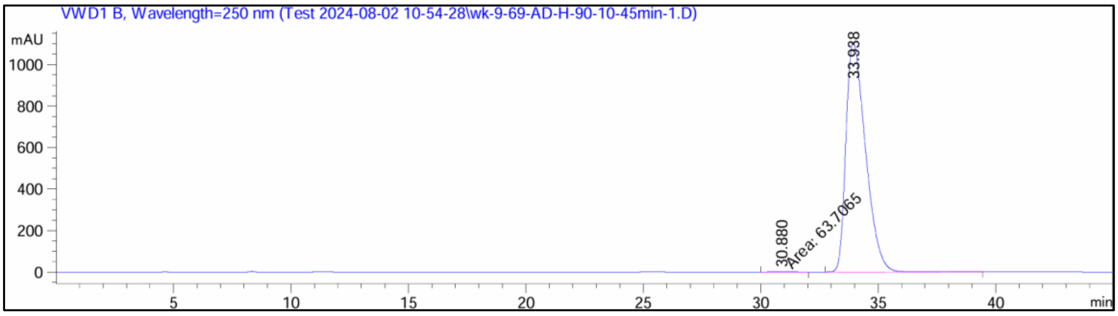

Peak #	RetTime [min]	Type	Width [min]	Area [mAU*s]	Height [mAU]	Area %
1	30.880	MM	0.8258	63.70646	1.28572	0.0993
2	33.938	BB	0.8965	6.41030e4	1105.49146	99.9007

Dynamic classical resolution of 1h' with [Ru]-2 and chiral ammonium salt

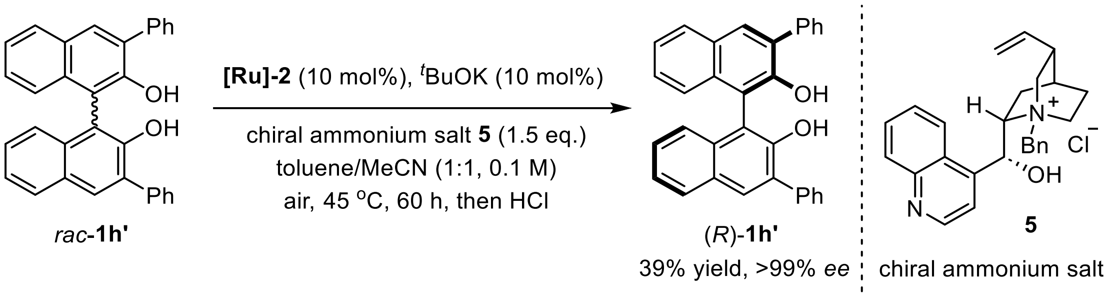

Under a N_2 atmosphere, to a 10 mL tube charged with a stir bar were added **[Ru]-2** (31.9 mg, 0.05 mmol), ^{t}BuOK (5.6 mg, 0.05 mmol) and 2.5 mL of toluene sequentially. The reaction tube was placed under sonication in a water bath for 2 min, and then let the mixture continue to stir at room temperature for additional 10 min. Then open the

tube, *rac*-**1h'** (219.3 mg, 0.50 mmol), chiral ammonium salt **5** (315.7 mg, 0.75 mmol) and 2.5 mL of MeCN were sequentially added to the rection mixture. After that, the tube was sealed with a rubber septum and stirred at 45 °C for 60 h. The resulting precipitate was isolated through filter paper by using a Buchner funnel and washed with cold MeCN (2 mL, 0 °C). The precipitated complex was fully dissolved in a mixture of HCl (aq., 1 M, 20 mL) and EtOAc (20 mL). The organic phase was separated, and the aqueous phase was further extracted with EtOAc (20 mL × 2). The combined organic phases were dried over anhydrous Na_2SO_4 and concentrated under reduced pressure to afford (*R*)-**1h'** (85.3 mg, 39% yield, >99% ee): light yellow solid. **HPLC conditions**: CHIRALPAK AD-H column, 25 °C, wavelength = 250 nm, *n*-Hexane/*i*-PrOH = 90:10, flow rate = 1.0 mL/min, t_R = 33.46 min for major isomer, t_S = 30.70 min for minor isomer.

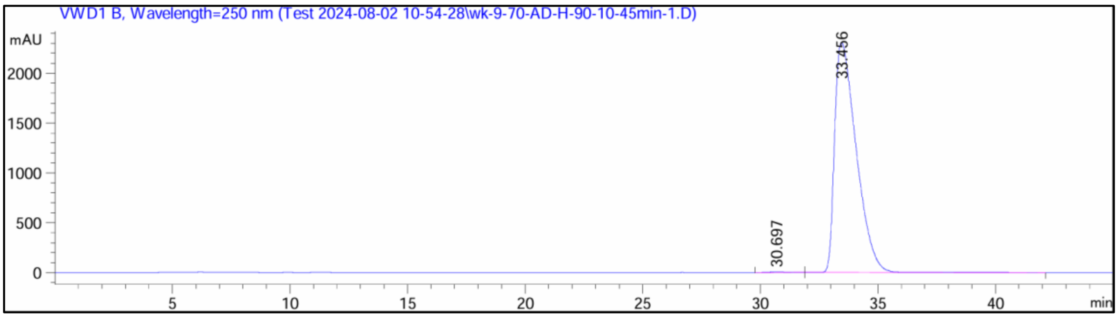

Peak #	RetTime [min]	Type	Width [min]	Area [mAU*s]	Height [mAU]	Area %
1	30.697	BB	0.6930	357.96872	8.07730	0.2425
2	33.456	BB	0.9792	1.47267e5	2305.66528	99.7575

Table S14. High-throughput screening of ligands for the racemization of (*S*)-**6**[a]

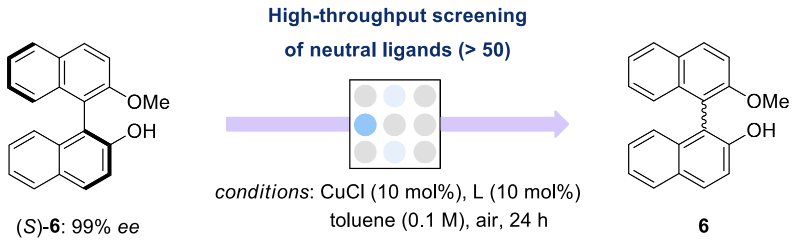

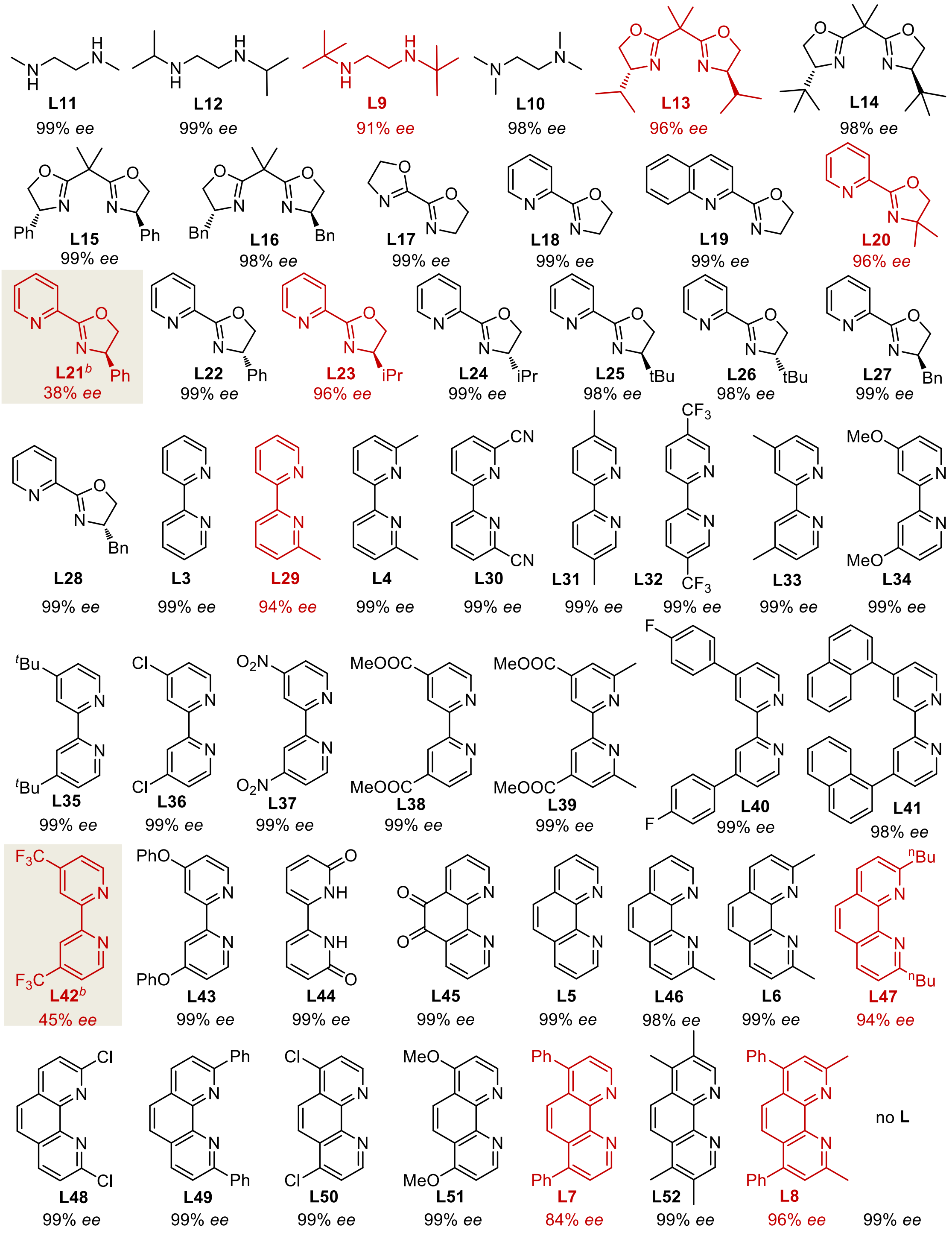

[a]Reaction conditions: 0.05 mmol or 0.025 mmol of (*S*)-**6**, CuCl (10 mol%), **L** (10 mol%), toluene (0.1 M), 40 °C, air, 24 h. Ee value was determined by chiral HPLC. [b]Temp. = 100 °C.

X-Ray Crystallography Data

X-Ray Structural Data for the Cu(L8)Cl₂

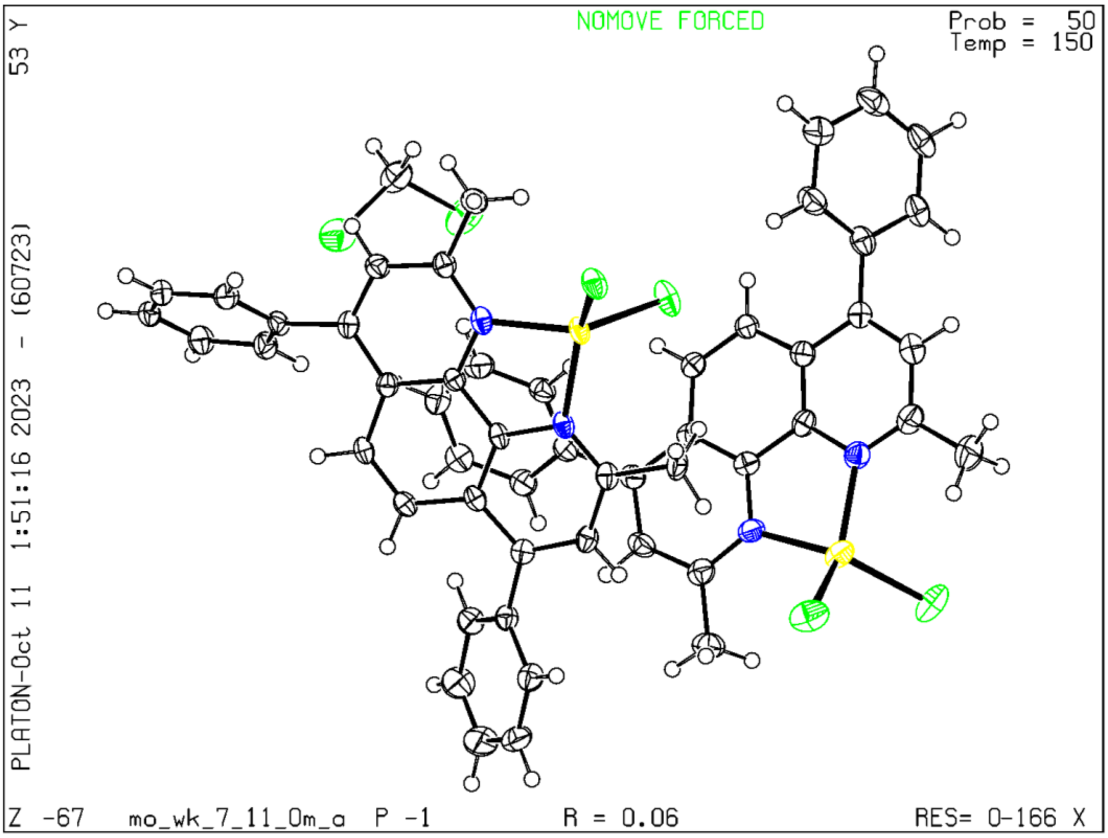

Crystals suitable for X-ray analysis were obtained via solution diffusion (solvent: DCM; antisolvent: hexane). **CCDC Number**: 2302595. Additional structural data can be found free of charge at ccdc.cam.ac.uk.

Table S15. Crystal data and structural refinement data for Cu(**L8**)Cl_2

**Name**	Cu(**L8**)Cl_2
**Identification code**	mo_wk_7_11_0m_a
**CCDC number**	2302595
**Chemical formula**	$C_{53}H_{42}Cl_6Cu_2N_4$
**Formula weight**	1074.68 g/mol
**Temperature**	150 K
**Wavelength**	0.71073 Å

**Crystal size**	0.2 × 0.2 × 0.1 mm^3
**Crystal system**	Triclinic
**Space group**	P -1
**Unit cell dimensions**	a = 11.9915(5) Å; α = 108.313(2)° b = 14.8367(7) Å; β = 99.180(2)° c = 15.2677(7) Å; γ = 103.501(2)°
**Volume**	2426.39(19) $Å^3$
**Z**	2
**Density (calculated)**	1.471 g/cm^3
**Absorption coefficient**	1.248 mm^{-1}
**F(000)**	1096.0
**Radiation source**	MoKα (λ = 0.71073)
**2Θ range for data collection**	4.784 to 56.564°
**Index ranges**	-12 ≤ h ≤ 15, -19 ≤ k ≤ 19, -20 ≤ l ≤ 20
**Reflections collected**	40514
**Independent reflections**	11982 [R_{int} = 0.0939, R_{sigma} = 0.0926]
**Data / restraints / paramet**	11982/0/590
**Goodness-of-fit on F^2**	1.071
**Final R indices [I>=2sigma(I)]**	R_1 = 0.0593, wR_2 = 0.1469
**Final R indices (all data)**	R_1 = 0.0995, wR_2 = 0.1680
**Largest diff. peak and hole**	0.88/-1.11 $eÅ^{-3}$

X-Ray Structural Data for the Cu(L10)Cl₂

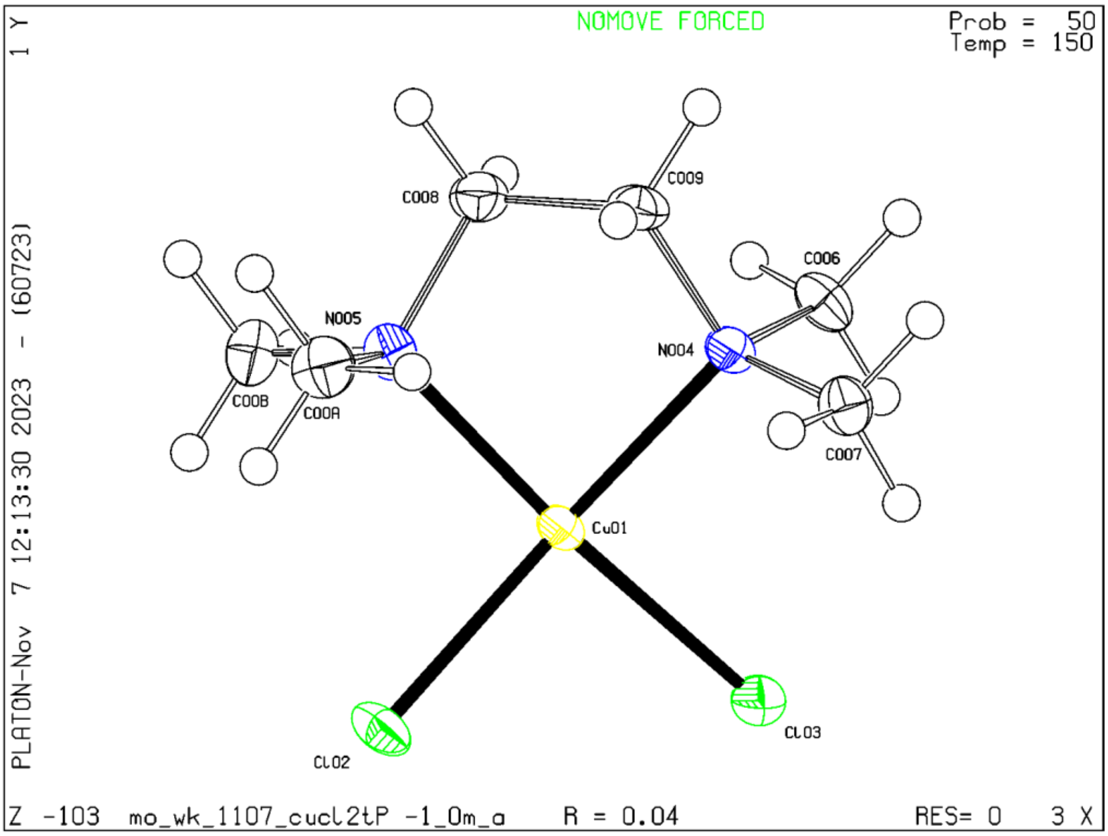

Crystals suitable for X-ray analysis were obtained via solution diffusion (solvent: DCM; antisolvent: hexane). **CCDC Number**: 2308678. Additional structural data can be found free of charge at ccdc.cam.ac.uk.

Table S16. Crystal data and structural refinement data for Cu(**L10**)Cl_2

**Name**	Cu(**L10**)Cl_2
**Identification code**	mo_wk_1107_cucl2tmeda_0m_a
**CCDC number**	2308678
**Chemical formula**	$C_6H_{16}Cl_2CuN_2$
**Formula weight**	250.65 g/mol
**Temperature**	150 K
**Wavelength**	0.71073 Å
**Crystal size**	0.2 × 0.2 × 0.2 mm^3

**Crystal system**	Triclinic
**Space group**	P -1
**Unit cell dimensions**	a = 7.6650(7) Å; α = 76.513(3)^o<br>b = 8.2843(7) Å; β = 85.305(4)^o<br>c = 9.1285(8) Å; γ = 64.891(3)^o
**Volume**	510.31(8) $Å^3$
**Z**	2
**Density (calculated)**	1.631 g/cm^3
**Absorption coefficient**	2.608 mm^{-1}
**F(000)**	258.0
**Radiation source**	MoKα (λ = 0.71073)
**2Θ range for data collection**	5.87 to 51.348^o
**Index ranges**	-9 ≤ h ≤ 8, -10 ≤ k ≤ 10, -10 ≤ l ≤ 11
**Reflections collected**	5323
**Independent reflections**	1899 [R_{int} = 0.0514, R_{sigma} = 0.0565]
**Data / restraints / paramet**	1899/0/104
**Goodness-of-fit on F^2**	1.078
**Final R indices [I>=2sigma(I)]**	R_1 = 0.0350, wR_2 = 0.0966
**Final R indices (all data)**	R_1 = 0.0395, wR_2 = 0.1048
**Largest diff. peak and hole**	0.57/-0.88 e$Å^{-3}$

DFT Calculation

Technical Details for the Computational Mechanistic Investigation

Density functional theory calculations (DFT) were performed by utilizing the hybrid B3LYP-D3(BJ) functional implemented in the Gaussian16 software package.[6-8] All the geometry optimizations were carried out with the pseudopotential basis set SDD for Cu and 6-31+g(d,p) basis set for other elements with the solute electron density (SMD) solvation model. Frequency calculations were preformed to obtain the Gibbs free energy corrections (at 298 K) at the same level as geometry optimization. The local minima representing intermediates were identified by the absence of any imaginary frequencies and the transition state was characterized by the presence of one imaginary frequency. To get more accurate energies, single point calculations were carried out with SDD for Cu and 6-311++g(d,p) for other elements based on the optimized structures. The final reported values are the large basis set energies (including dispersion correction and solvation effect), corrected for the Gibbs free energies. The IRI (Interaction Region Indicator) analysis was performed to reveal the weak interaction using the Multiwfn 3.8 program.[9]

The binding energies were calculated to evaluate the feasibility of the substrate separating from the catalyst. The binding energy was determined as the energy difference between the bound compound and its respective components, which is expressed by the equation:

$$\Delta G = G(\mathrm{AB}) + G(\mathrm{A}) - G(\mathrm{B}) \qquad (1)$$

where G(AB) is the free energy of compound AB, and G(A) and G(B) are the free energies of respective components A and B.

The activation strain model (ASM) is a fragment based approach that can be used to gain physical insights into the energy barriers of chemical reactions.[10] This model decomposes the relative energy of a molecular complex along the reaction energy profile (ΔE), into two terms, the strain (or distortion) energy ($\Delta E_{\mathrm{strain}}$) of the fragments and their mutual interaction energy (ΔE_{int}):

$$\Delta E = \Delta E_{\mathrm{strain}} + \Delta E_{\mathrm{int}} \qquad (2)$$

The strain energy, ΔE_{strain}, which arises from the distortion of the individual fragments, is the energy required to deform the fragments into the geometry they adopt in the interacting complex. The interaction energy, ΔE_{int}, represents the actual interaction between the deformed fragments.

Hypothetical Racemization Mechanism of Substrate

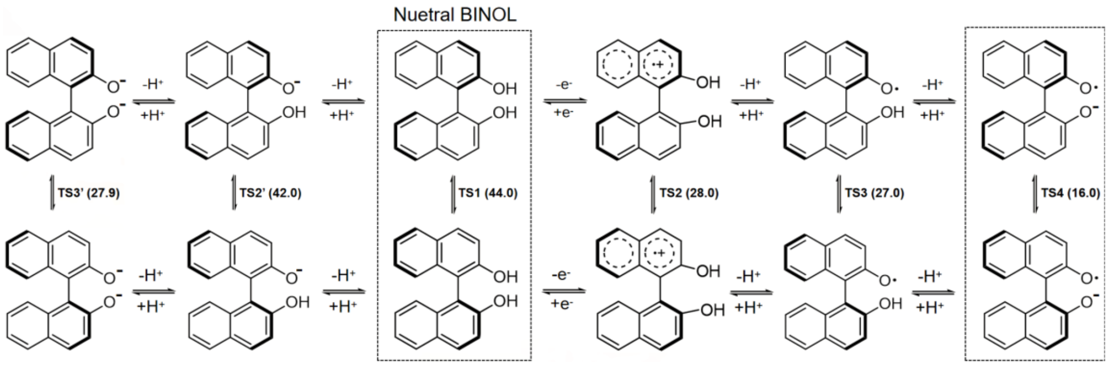

Figure S12. Hypothetical racemization mechanism of the substrate. The energies of transition states relative to corresponding substrates are given in kcal/mol.

Optimized Structures and the Corresponding Energies

The optimized structures by using DFT calculations and their corresponding energies (in a.u) are given in this section. The provided energies are the large basis set energies including solvation effect ($E_{bb\text{-}solv}$), the Gibbs free energy corrections ($E_{Gibbs\text{-}correct}$), and the total Gibbs free energies (G_{Total}). The Cartesian Coordinates of optimized structures are also given.

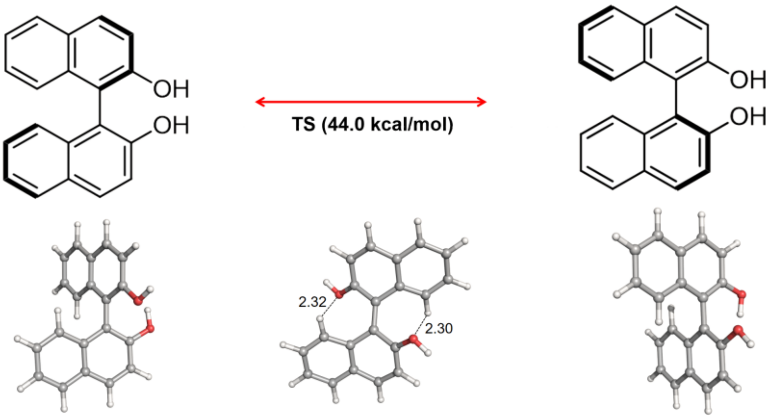

$E_{\text{bb-solv}}$	-921.32803	-921.26028	-921.32803
$E_{\text{Gibbs-correct}}$	0.23825	0.24067	0.23824
G_{Total}	-921.08978	-921.01961	-921.08979
**Cartesian Coordinates**	C 1.05103900 1.52929100 -1.23868500	C -1.05179700 1.52968300 -1.23818500	C 0.51400500 1.83448900 -0.32809600
	C 0.69415400 0.62276800 -0.24828600	C -0.69428700 0.62291300 -0.24821600	C 0.59022300 0.44887400 -0.06217100
	C 1.68248800 -0.29588900 0.23815500	C -1.68224900 -0.29602400 0.23840400	C 1.97769400 -0.03472200 -0.08214400
	C 3.01273600 -0.26475000 -0.29919200	C -3.01272100 -0.26503900 -0.29839400	C 3.05765400 0.84671700 0.26102400
	C 3.32115700 0.68756200 -1.30777900	C -3.32178000 0.68751300 -1.30656700	C 2.79846100 2.23755200 0.39410100
	C 2.36799400 1.56386600 -1.76857100	C -2.36898900 1.56415300 -1.76749000	C 1.57585300 2.71733000 0.00339200
	H 0.38228500 -1.29310200 1.65733300	H -0.38125700 -1.29323500 1.65684800	H 1.59919900 -1.93050700 -1.06098800
	C 1.38545900 -1.25652000 1.24590600	C -1.38460600 -1.25680900 1.24584500	C 2.35112300 -1.30491300 -0.60028800
	C 3.98508600 -1.18045700 0.18758600	C -3.98465600 -1.18109500 0.18855600	C 4.39019900 0.35637400 0.32347700
	H 4.32753000 0.71693400 -1.71688600	H -4.32833200 0.71679300 -1.71523800	H 3.59455000 2.91314300 0.69316500
	H 2.61145100 2.28812700 -2.54265900	H -2.61289700 2.28860600 -2.54125800	H 1.39291200 3.78839600 -0.05833500
	C 3.66490600 -2.09787300 1.16544400	C -3.66388700 -2.09866400 1.16607100	C 4.69450900 -0.92596800 -0.08052000
	C 2.35129300 -2.13269800 1.69569500	C -2.35006000 -2.13330800 1.69581800	C 3.66362000 -1.73722400 -0.60480600
	H 4.98928300 -1.14416000 -0.22759600	H -4.98902600 -1.14491800 -0.22621900	H 5.17614000 1.03792500 0.63962700
	H 4.41436700 -2.79405100 1.53037000	H -4.41305100 -2.79508900 1.53113000	H 5.71884900 -1.28615300 -0.05526000
	H 2.10212400 -2.85823100 2.46492200	H -2.10043500 -2.85894700 2.46479600	H 3.90428900 -2.70495400 -1.03672700
	C -0.69986500 0.60471400 0.28842600	C 0.69999900 0.60513800 0.28785200	C -0.59112300 -0.43367200 0.07940200
	C -1.69651500 -0.26509700 -0.25592200	C 1.69622600 -0.26548600 -0.25593900	C -1.98292900 0.03764700 0.07920600
	C -1.03610300 1.44254000 1.34124100	C 1.03690000 1.44418600 1.33949600	C -0.50531200 -1.81595800 0.35993400
	C -1.41954400 -1.15376000 -1.33235700	C 1.41859000 -1.15540300 -1.33118100	C -2.37671400 1.30557700 0.58733100
	C -3.02078600 -0.25707100 0.29636500	C 3.02079400 -0.25700000 0.29565800	C -3.04932900 -0.85740300 -0.27217900
	C -2.34337600 1.45454700 1.88967900	C 2.34448000 1.45667000 1.88721300	C -1.55449800 -2.71222700 0.02432400
	C -2.39840700 -1.98580500 -1.83591300	C 2.39706100 -1.98824500 -1.83418300	C -3.69363200 1.72447100 0.57095000
	H -0.42073300 -1.17055000 -1.75662700	H 0.41956700 -1.17256100 -1.75492600	H -1.63865300 1.94009300 1.05726800
	C -4.00590400 -1.12572500 -0.24609400	C 4.00548700 -1.12650200 -0.24621000	C -4.38568100 -0.38102800 -0.35628900
	C -3.30912500 0.62408500 1.37570600	C 3.30983600 0.62544900 1.37375200	C -2.77549900 -2.24705400 -0.38871100
	H -2.55323500 2.12942800 2.71342100	H 2.55481400 2.13254100 2.71002700	H -1.36364800 -3.78098900 0.10012500
	C -3.70608200 -1.97486800 -1.29097000	C 3.70500200 -1.97688700 -1.28988200	C -4.70858700 0.90051600 0.03582800
	H -2.16449100 -2.65625500 -2.65835700	H 2.16262800 -2.65965600 -2.65569400	H -3.95024900 2.69145500 0.99528800
	H -5.00628500 -1.10887500 0.17983300	H 5.00607700 -1.10930000 0.17921400	H -5.16019900 -1.07260800 -0.67878300
	H -4.31198200 0.63239100 1.79468800	H 4.31292200 0.63410600 1.79217800	H -3.56143600 -2.93258400 -0.69198000
	H -4.46756800 -2.63455500 -1.69690200	H 4.46616500 -2.63721800 -1.69536800	H -5.73603700 1.25016400 -0.00590700
	O -0.13793700 2.29572600 1.91703100	O 0.13921000 2.29812900 1.91479200	O -0.58307800 2.36028100 -0.94652400
	H 0.71646900 2.20941400 1.46370400	H -0.71552900 2.21125400 1.46220700	H -0.49899900 3.32282700 -1.00679400
	O 0.09219500 2.38904000 -1.69633700	O -0.09333800 2.38974800 -1.69599300	O 0.59083500 -2.32346600 0.99517400
	H 0.46307900 2.97219400 -2.37340000	H -0.46467900 2.97314300 -2.37259900	H 0.52099700 -3.28700100 1.05752100

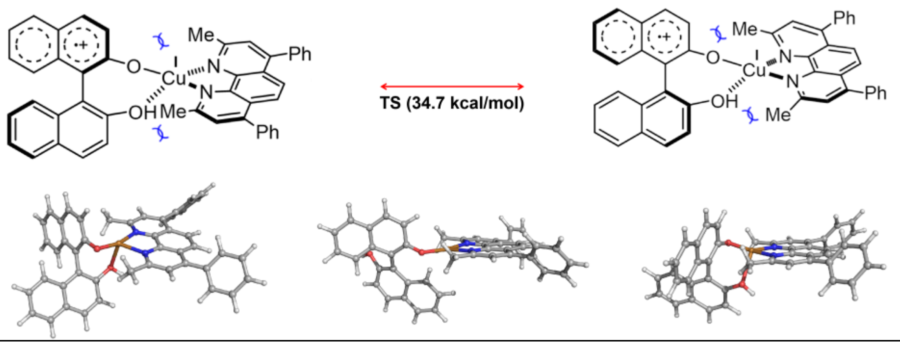

$E_{\text{bb-solv}}$	-2230.69770	-2230.64309	-2230.69205
$E_{\text{Gibbs-correct}}$	0.58791	0.58866	0.58776
G_{Total}	-2230.10980	-2230.05443	-2230.10429

Cartesian Coordinates

C	-2.34735100	-3.48031100	-0.58638900	C	3.16006800	-1.07404800	-0.76686900	C	-1.47870100	3.24771900	-0.38762100
C	-3.49667300	-2.77999700	-0.24326200	C	4.40944400	-0.58091900	-0.17902800	C	-2.80184300	2.83196100	-0.50715400
C	-3.39224500	-1.36084600	-0.08263400	C	5.58797900	-1.28654700	-0.69999700	C	-3.08069300	1.43545800	-0.33650000
C	-2.13420800	-0.77144300	-0.34735200	C	5.57380100	-1.86831700	-2.00486000	C	-1.99734500	0.59614800	0.00671200
C	-1.11708200	-2.82623400	-0.80728300	C	4.35005000	-1.85171900	-2.76384000	C	-0.44986400	2.34225000	-0.06252900
C	-4.45583900	-0.51503700	0.37945200	C	3.19148600	-1.49775600	-2.15374400	C	-4.36722100	0.83333400	-0.54642600
C	-1.96996900	0.66097000	-0.23896400	H	6.76267600	-1.12512400	1.11006400	C	-2.20552400	-0.81799200	0.21453100
C	-3.05879100	1.47750400	0.14438700	C	6.74664800	-1.48795400	0.08789600	C	-3.49907200	-1.37121200	0.06683300
C	-4.29585700	0.83781600	0.49135400	C	6.72572200	-2.50182900	-2.50943800	C	-4.56657800	-0.50758600	-0.35651200
C	-2.82749500	2.88923000	0.21886700	H	4.35804900	-2.20250400	-3.79257500	C	-3.63865600	-2.77832900	0.29852800
C	-1.53929200	3.35353300	-0.02835600	H	2.22274100	-1.56894500	-2.64194800	C	-2.49291000	-3.50715900	0.59158300
C	-0.49406800	2.47776500	-0.38136600	C	7.86603400	-2.63919800	-1.72728400	C	-1.22922800	-2.88869700	0.70898000
H	-5.39652700	-0.96743300	0.66929000	C	7.85907700	-2.15197000	-0.40946500	H	-5.18700900	1.45325800	-0.88810700
H	-2.39366700	-4.55467300	-0.72499900	H	6.69944200	-2.90921600	-3.51687400	H	-1.22974300	4.29643200	-0.50744500
H	-5.10916100	1.44199900	0.87419800	H	8.74418900	-3.14025000	-2.12305300	H	-5.54214500	-0.93613500	-0.55148900
H	-1.33178000	4.41670000	0.02523900	H	8.72574200	-2.29854200	0.22855700	H	-2.56672400	-4.57441300	0.77211900
N	-0.72960700	1.16315600	-0.49830800	C	4.59632700	0.50731700	0.78174000	N	-1.10906500	-1.56670900	0.53710800
N	-1.03562200	-1.49612400	-0.69873700	C	3.66966100	1.03272000	1.80318600	N	-0.72437800	1.05596900	0.14425300
C	-3.89596800	3.86013900	0.56350000	C	5.83363000	1.22904500	0.70835100	C	-4.95393500	-3.46491800	0.23514800
C	-5.10281100	3.90433200	-0.15715600	C	2.26668500	0.85035700	1.78983600	C	-6.03460000	-3.02229900	1.01903400
C	-3.68044900	4.79690300	1.58958600	C	4.17386500	1.85472500	2.87197600	C	-5.12200300	-4.59362900	-0.58412800
C	-6.07212700	4.86172900	0.14704100	C	6.32971500	1.98311700	1.79858400	C	-7.25835000	-3.69329600	0.97752300
H	-5.26839000	3.20890500	-0.97475400	C	1.42906900	1.37221500	2.76199000	H	-5.90830300	-2.16600500	1.67502700
C	-4.65823400	5.74322800	1.90163600	H	1.83150200	0.28178400	0.99254600	C	-6.35129700	-5.25400800	-0.63409900
H	-2.75355000	4.76874800	2.15558100	C	3.30743000	2.34399500	3.88258500	H	-4.29438800	-4.93890200	-1.19764100
C	-5.85576700	5.77877100	1.18062300	C	5.53958500	2.25258800	2.88254200	C	-7.42157300	-4.80652200	0.14570700
H	-6.99341000	4.89467900	-0.42727100	H	7.33984100	2.37867600	1.73871500	H	-8.08231200	-3.34845400	1.59559100
H	-4.48468500	6.45129400	2.70678600	C	1.94754000	2.10889800	3.84203400	H	-6.47254400	-6.11578400	-1.28391600
H	-6.61413700	6.51849700	1.41970000	H	0.35828300	1.20319900	2.67814200	H	-8.37627900	-5.32289500	0.10880700
C	-4.77517500	-3.51336200	-0.06754500	H	3.73655500	2.94328200	4.68115400	C	-3.85950200	3.83229100	-0.79880700
C	-4.82325000	-4.65228700	0.75515700	H	5.92226200	2.82543700	3.72256600	C	-3.70747900	4.72085400	-1.87765600
C	-5.93960100	-3.11398500	-0.74744900	H	1.29258100	2.50395900	4.61278200	C	-4.98764200	3.95486900	0.03150800
C	-6.01270300	-5.36773200	0.90517700	O	2.04662200	-1.16089200	-0.15917100	C	-4.67229700	5.69861800	-2.13081000
H	-3.93166700	-4.96318700	1.29231300	C	-1.28532900	2.90687400	-1.29106400	H	-2.84116200	4.63341600	-2.52707600
C	-7.12410400	-3.83887000	-0.60563300	C	-2.64703400	2.75571800	-1.05241500	C	-5.94241900	4.94194400	-0.21551300
H	-5.90995300	-2.25019200	-1.40535400	C	-3.13740500	1.44429700	-0.75653800	H	-5.10064200	3.29571900	0.88728200
C	-7.16549700	-4.96369000	0.22466500	C	-2.17783300	0.41622500	-0.61233200	C	-5.79144200	5.81195300	-1.29881100
H	-6.03892900	-6.23799100	1.55458300	C	-0.39895900	1.81638900	-1.18136200	H	-4.54890000	6.37156200	-2.97424400
H	-8.01296600	-3.52720500	-1.14647000	C	-4.52566600	1.09621800	-0.64259000	H	-6.80111400	5.03493700	0.44305300
H	-8.09014500	-5.52178300	0.33910300	C	-2.58685500	-0.91737900	-0.22910800	H	-6.53798700	6.57678300	-1.49103200
C	0.91055600	2.96028400	-0.62936700	C	-3.95959400	-1.21109300	-0.05520100	C	0.00278400	-3.67279000	1.05024300
H	1.59929400	2.39677900	0.00121800	C	-4.91667100	-0.17318000	-0.32120800	H	0.81064200	-3.44471400	0.34976000
H	1.20282500	2.79058000	-1.67270500	C	-4.29949200	-2.54447200	0.33765400	H	0.37093800	-3.38666100	2.04079700
H	1.01418400	4.02360500	-0.40596200	C	-3.26484600	-3.46134000	0.47966900	H	-0.19608200	-4.74638400	1.04074500
C	0.12072600	-3.58221400	-1.18897800	C	-1.91912700	-3.09096700	0.28719000	C	0.97448400	2.77859200	0.11471600
H	0.45636100	-3.28274200	-2.18691700	H	-5.27454600	1.85264100	-0.84617900	H	1.23993000	2.79138600	1.18023500
H	0.94222000	-3.34031200	-0.50855100	H	-0.88669000	3.89027900	-1.51834600	H	1.65511800	2.07952200	-0.37885500
H	-0.05460900	-4.65971000	-1.17856700	H	-5.97303600	-0.41080300	-0.28062200	H	1.13785900	3.77943500	-0.28908800
C	0.58115500	-0.25849700	-0.96307200	H	-3.48532800	-4.47868600	0.78498100	Cu	0.54759900	-0.37933100	0.70394800
C	2.76922900	-0.11588500	-1.72167100	N	-1.59746000	-1.83830300	-0.04935900	C	2.67137600	-0.23215000	1.66205100
C	3.57057600	0.28358700	-0.61691500	N	-0.84933700	0.61358100	-0.81384300	C	3.56426600	0.24445500	0.65618800
C	4.25934000	1.52554900	-0.66920800	C	-5.69599600	-2.97609700	0.59906100	C	4.23035000	1.49198600	0.83977900
C	4.22071400	2.31671200	-1.86985200	C	-6.51498900	-2.28085900	1.50642300	C	4.01763800	2.24992600	2.04252400
C	3.46968300	1.84839800	-2.99137800	C	-6.19811700	-4.13280400	-0.02262600	C	3.14355800	1.73114600	3.04552100
C	2.75947300	0.68461900	-2.92256900	C	-7.80784700	-2.73116100	1.77978100	C	2.49326500	0.54488500	2.86536800
H	5.03597400	1.43257900	1.35484900	H	-6.13023200	-1.40147000	2.01457700	H	5.25362000	1.48182100	-1.07528500
C	5.00000100	2.02054800	0.44390700	C	-7.49607000	-4.57349900	0.24287900	C	5.09313900	2.03655400	-0.15698300
C	4.91711200	3.54782000	-1.91088200	H	-5.57335600	-4.67769600	-0.72503500	C	4.68097900	3.48908100	2.21010400
H	3.46590500	2.44218000	-3.90209200	C	-8.30399000	-3.87429700	1.14488000	H	3.00548700	2.30078200	3.96127700
H	2.18261900	0.31107800	-3.76307000	H	-8.42624000	-2.19080400	2.49080400	H	1.82635100	0.13867600	3.62082100
C	5.62677500	3.99965400	-0.81212500	H	-7.87498600	-5.46192600	-0.25389200	C	5.51949400	3.98334000	1.22765900
C	5.66402900	3.22844100	0.37353300	H	-9.31232700	-4.21937100	1.35411100	C	5.72048300	3.25046900	0.03379900
H	4.88536600	4.13670300	-2.82406700	C	-3.53039900	3.94763800	-1.06696000	H	4.52169900	4.04779400	3.12857600
H	6.15714200	4.94629700	-0.85771700	C	-3.49002400	4.85370400	-2.14021600	H	6.02344600	4.93486700	1.36926300
H	6.22259700	3.58848000	1.23257200	C	-4.37107300	4.22216400	0.02696500	H	6.37586700	3.64631000	-0.73665900
C	3.67615300	-0.61300500	0.57052400	C	-4.28170700	6.00404500	-2.12411300	C	3.83839100	-0.60292100	-0.54467400
C	4.85878800	-1.39543400	0.78157000	H	-2.84805500	4.64627100	-2.99170200	C	5.07684100	-1.31622000	-0.68824000
C	2.63638600	-0.73572800	1.47182900	C	-5.15158100	5.37892700	0.04591500	C	2.87797800	-0.75090500	-1.53351000
C	5.96500800	-1.35148400	-0.11312300	H	-4.38977600	3.54189900	0.87349500	C	6.09949300	-1.27670400	0.30138000
C	4.94315000	-2.25841200	1.92550200	C	-5.11203500	6.27079200	-1.03088700	C	5.29390100	-2.12517000	-1.85675400
C	2.70752500	-1.58423800	2.60118400	H	-4.25014300	6.68971300	-2.96582900	C	3.08744000	-1.54009600	-2.68752000
C	7.09156100	-2.11377100	0.11567100	H	-5.78676400	5.58670000	0.90214400	C	7.27374300	-1.98183200	0.13506200
H	5.91644800	-0.71010100	-0.98662200	H	-5.72297500	7.16871200	-1.01703800	H	5.94855200	-0.68910700	1.19991800
C	6.12034200	-3.02918100	2.13307700	C	-0.79267400	-4.06745300	0.46807000	C	6.51750000	-2.83520300	-1.99717300
C	3.84405300	-2.32653700	2.82271400	H	-0.11068800	-4.02073000	-0.38690700	C	4.27550100	-2.20817300	-2.84349300
H	1.86392500	-1.64062400	3.28555300	H	-0.21279000	-3.81300500	1.36321000	H	2.30636400	-1.60765900	-3.44192700
C	7.17556700	-2.96029300	1.24910300	H	-1.16110700	-5.09038900	0.57362700	C	7.49071900	-2.76556100	-1.02579200
H	7.92296300	-2.06637800	-0.58195800	C	1.07909400	1.93736000	-1.42054900	H	8.03882100	-1.94019500	0.90502300
H	6.17070200	-3.67884600	3.00317800	H	1.61807000	1.83629200	-0.47174000	H	6.66887400	-3.44109200	-2.88665100
H	3.90706000	-2.97835700	3.68963500	H	1.42155900	1.12773600	-2.07701600	H	4.44523900	-2.81506000	-3.72831000
H	8.07027500	-3.55261400	1.41654400	H	1.34049400	2.89600700	-1.87354400	H	8.42148100	-3.31255000	-1.14239100
O	1.48936200	-0.00384000	1.22272100	Cu	0.18691400	-1.02363700	-0.32092200	O	1.68727700	-0.05917000	-1.37785300
O	2.04274600	-1.22421600	-1.66349300	O	6.55288000	1.21062800	-0.43141100	O	1.96084700	-1.34007000	1.51116100
H	0.82940300	-0.16548200	1.91186300	H	7.29055200	1.83470200	-0.37047600	H	1.12193100	-0.17331500	-2.15642700

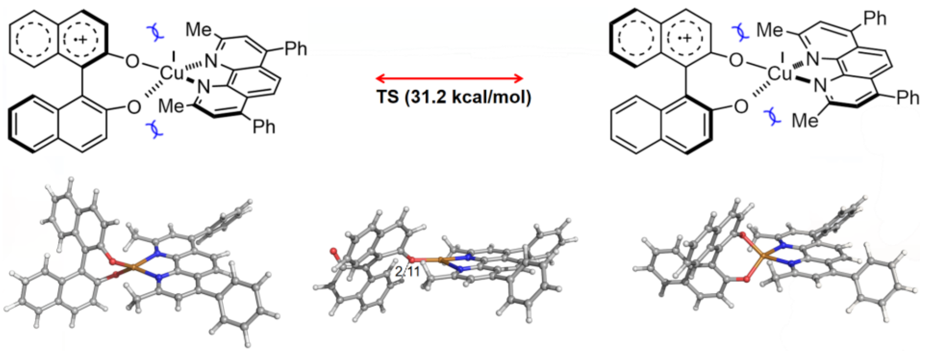

***E*bb-solv**	-2230.24973	-2230.19569	-2230.24972
***E*Gibbs-correct**	0.57384	0.56958	0.57352
***G*Total**	-2229.67589	-2229.62611	-2229.67620

Cartesian Coordinates

C	-2.39621700	-3.39923000	-0.35706200	C	2.82170000	0.16842000	-1.00409700	C	-1.72994100	3.34303700	-0.86634100
C	-3.59246600	-2.69921600	-0.28963500	C	4.22204000	-0.08602400	-0.70914300	C	-3.03924300	2.89438600	-0.77870300
C	-3.53346300	-1.29851200	-0.00877500	C	4.66675700	-1.43595300	-1.12183400	C	-3.25476500	1.50048800	-0.54291100
C	-2.24910900	-0.71599400	0.11746300	C	4.01173000	-2.13232900	-2.18472900	C	-2.10705700	0.68414300	-0.39742900
C	-1.15195800	-2.75315000	-0.19637700	C	2.81335700	-1.57684400	-2.73783700	C	-0.63362500	2.46481500	-0.71702800
C	-4.68486000	-0.46888000	0.20278900	C	2.22269500	-0.51103900	-2.12322500	C	-4.54760100	0.88026300	-0.49070200
C	-2.11914200	0.70046400	0.38399900	H	6.10328200	-1.72469400	0.47269300	C	-2.25664300	-0.72891300	-0.12374000
C	-3.27774800	1.50412300	0.51338700	C	5.66202600	-2.15776200	-0.41735700	C	-3.54887900	-1.29575800	-0.00683100
C	-4.56252800	0.86812800	0.45346200	C	4.47642500	-3.40187000	-2.60469400	C	-4.68828400	-0.45372800	-0.23363300
C	-3.08091500	2.90201100	0.74185500	H	2.34088800	-2.06462000	-3.58728200	C	-3.62688500	-2.69423600	0.28062600
C	-1.77780500	3.36678600	0.83809200	H	1.25113800	-0.13867400	-2.44141700	C	-2.43957900	-3.40779100	0.36305600
C	-0.66991600	2.50052500	0.70642400	C	5.51041200	-4.03990200	-1.94238300	C	-1.18641800	-2.77689700	0.21192900
H	-5.66874300	-0.92241200	0.18613300	C	6.07730900	-3.41977500	-0.81143600	H	-5.42734500	1.48332400	-0.68137500
H	-2.40617700	-4.46384400	-0.56472800	H	3.97721300	-3.88535800	-3.44163800	H	-1.53249500	4.39796600	-1.02472600
H	-5.45110200	1.46151700	0.63306300	H	5.85369900	-5.01880700	-2.26521500	H	-5.67761500	-0.89541700	-0.22334700
H	-1.59454300	4.42510400	0.99073400	H	6.84260500	-3.93331600	-0.23556000	H	-2.46387000	-4.47118200	0.57575600
N	-0.85713200	1.19383200	0.49791300	C	5.12179300	0.79954400	-0.03261000	N	-1.11623800	-1.45918300	0.00012800
N	-1.09900400	-1.43367600	0.00937400	C	4.84211700	1.99413400	0.79633300	N	-0.83806900	1.16186900	-0.50142000
C	-4.20964500	3.86094300	0.87002000	C	6.58663600	0.53159700	-0.17302400	C	-4.92016500	-3.39623100	0.48830100
C	-5.18880200	3.97070900	-0.13276500	C	3.81789000	2.94383500	0.57335400	C	-5.84663900	-2.93798400	1.44148600
C	-4.28248200	4.71062600	1.98696500	C	5.74137600	2.28099500	1.88047800	C	-5.21340600	-4.56280700	-0.23834600
C	-6.21784400	4.90739900	-0.01795600	C	7.41255500	0.74044200	1.01978400	C	-7.03898400	-3.63060900	1.65994000
H	-5.13069800	3.33543200	-1.01176600	C	3.61468400	4.02841800	1.42140600	H	-5.62052800	-2.05066900	2.02554900
C	-5.31874000	5.63959800	2.10497300	H	3.16895300	2.83068200	-0.27790800	C	-6.41173300	-5.24801700	-0.02602300
H	-3.53314000	4.62970900	2.76944300	C	5.48793400	3.35282700	2.75869100	H	-4.50656900	-4.92170100	-0.98114300
C	-6.28859200	5.74114600	1.10290500	C	6.97818000	1.54155000	2.01967700	C	-7.32728000	-4.78448100	0.92374600
H	-6.96121300	4.98868600	-0.80598400	H	8.39697600	0.28116400	1.02076200	H	-7.74032100	-3.27090200	2.40749300
H	-5.36734000	6.28253300	2.97922900	C	4.42445000	4.22207800	2.54957400	H	-6.62973700	-6.14225000	-0.60294900
H	-7.09215000	6.46652200	1.19308800	H	2.81963100	4.73483100	1.19565900	H	-8.25762400	-5.31961100	1.09120600
C	-4.87567300	-3.41699700	-0.50587400	H	6.17152000	3.51527200	3.58907500	C	-4.15580800	3.86538700	-0.92186800
C	-5.16303200	-4.58259400	0.22465900	H	7.59696700	1.73125100	2.89449100	C	-4.21192600	4.70744300	-2.04546300
C	-5.79794600	-2.97436000	-1.47044700	H	4.24887900	5.05413600	3.22583100	C	-5.14004900	3.99354100	0.07372400
C	-6.35166800	-5.28219600	0.00486000	O	7.10623200	0.15501900	-1.23780100	C	-5.23711200	5.64681500	-2.17721300
H	-4.45946400	-4.92946800	0.97620700	O	2.10773300	1.00544300	-0.32593500	H	-3.45834500	4.61240300	-2.82226900
C	-6.98046700	-3.68132400	-1.69624600	C	-1.30204200	-3.09938000	0.89616000	C	-6.15791300	4.94060300	-0.05477300
H	-5.57615300	-2.08770600	-2.05718200	C	-2.67442600	-2.91663100	0.80131600	H	-5.09457600	3.36426200	0.95779800
C	-7.26311700	-4.83413300	-0.95620500	C	-3.15821100	-1.60130200	0.51593300	C	-6.21226400	5.76652700	-1.18232800
H	-6.56540400	-6.17548600	0.58483300	C	-2.18962900	-0.59017300	0.29271500	H	-5.27299000	6.28356700	-3.05660400
H	-7.67855400	-3.33349100	-2.45239600	C	-0.40604600	-2.02654700	0.69078400	H	-6.90539700	5.03607000	0.72776300
H	8.18592600	-5.38035700	-1.12944100	C	-4.54618900	-1.23769000	0.50019500	H	-7.00724100	6.49990900	-1.28326600
C	0.73493900	3.02353600	0.74008400	C	-2.61292200	0.76184800	-0.02879400	C	0.09697800	-3.54830300	0.28443800
H	1.38239700	2.34933100	1.30686000	C	-3.99527900	1.07084900	-0.08940000	H	0.66013400	-3.42619400	-0.64785400
H	1.13620700	3.06116000	-0.28085000	C	-4.94396000	0.03709500	0.21551100	H	0.73644900	-3.14963900	1.07723800
H	0.77260800	4.03211200	1.15840200	C	-4.35839500	2.41620200	-0.40571400	H	-0.08826900	-4.61166700	0.45052600
C	0.14087000	-3.51014000	-0.25094500	C	-3.33715000	3.33882000	-0.58975300	C	0.77738600	2.97162100	-0.73860400
H	0.78696400	-3.10441300	-1.03470500	C	-1.98377700	2.95715300	-0.51172200	H	1.16762000	3.01169300	0.28652100
H	0.68953000	-3.38144500	0.68911200	H	-5.28997800	-1.98648900	0.74581400	H	1.42373500	2.28623200	-1.29305100
H	-0.02995700	-4.57562800	-0.41909500	H	-0.90312700	-4.08613300	1.10856100	H	0.83103000	3.97683300	-1.16319500
Cu	0.60068900	-0.29578900	0.29145500	H	-5.99840500	0.28477400	0.24139200	Cu	0.59966700	-0.34350300	-0.25960500
C	2.61817000	-0.09531500	-1.39088300	H	-3.57883900	4.36821400	-0.83157400	C	2.58569400	-0.15534900	1.46196400
C	3.70635300	0.32838700	-0.60826800	N	-1.64014800	1.69078700	-0.25040800	C	3.69392600	0.24871800	0.69722800
C	4.47313100	1.46233200	-1.02979200	N	-0.85616600	-0.81154400	0.38286300	C	4.46599900	1.37702300	1.12419500
C	4.19031200	2.09586700	-2.29044600	C	-5.77213800	2.85602200	-0.54314900	C	4.16679000	2.02432700	2.37398900
C	3.12111000	1.59541200	-3.08567400	C	-6.64466000	2.22151600	-1.44468700	C	3.07675200	1.54307300	3.15262000
C	2.35293800	0.54718900	-2.64320800	C	-6.24231500	3.95241500	0.19952400	C	2.30460000	0.50045600	2.70392100
H	5.74229100	1.56153300	0.72725800	C	-7.95713700	2.67303000	-1.59655800	H	5.76878100	1.44716300	-0.60945100
C	5.52371100	2.01171900	-0.23468900	H	-6.28642800	1.38728000	-2.04077700	C	5.53772400	1.90760900	0.34468500
C	4.97057200	3.20757700	-2.70786700	C	-7.55878900	4.39580900	0.05408100	C	4.95200900	3.13035900	2.79714400
H	2.91215100	2.06664000	-4.04386800	H	-5.57691200	4.44533200	0.90288100	H	2.85524100	2.02464800	4.10281900
H	1.51897300	0.16954200	-3.22900700	C	-8.41947200	3.75799900	-0.84449700	H	1.45505100	0.13766300	3.27657200
C	5.98760800	3.70355600	-1.91748000	H	-8.61686300	2.17979700	-2.30479600	C	5.98958900	3.60789700	2.02223700
C	6.25731300	3.09844600	-0.66437000	H	-7.91107600	5.23770000	0.64326800	C	6.27573100	2.98936100	0.77937100
H	4.74682800	3.66752400	-3.66821900	H	-9.44210900	4.10563700	-0.95994800	H	4.71548800	3.60091500	3.74925900
H	6.57602900	4.55500700	-2.24851600	C	-3.58303500	-4.07648500	1.00061300	H	6.58157600	4.45523700	2.35743600
H	7.05005900	3.49439600	-0.03484000	C	-3.45946800	-4.87758800	2.14853200	H	7.08469800	3.37098300	0.16173600
C	3.99326800	-0.37153100	0.67582100	C	-4.54325900	-4.42086900	0.03310600	C	3.99680700	-0.46458800	-0.57583200
C	5.20965300	-1.10760800	0.85584000	C	-4.28378900	-5.99041400	2.33000900	C	5.20720500	-1.21669000	-0.72727600
C	3.04670700	-0.33609400	1.71524700	H	-2.72459800	-4.61648000	2.90456600	C	3.07114100	-0.42444500	-1.63371200
C	6.16973700	-1.26493600	-0.18922800	C	-5.36007300	-5.53917600	0.21142900	C	6.14576500	-1.37707200	0.33672900
C	5.50131500	-1.72534200	2.12142700	H	-4.63342500	-3.82260300	-0.86895000	C	5.51482800	-1.84798300	-1.98237900
C	3.36082400	-0.95210800	2.96852100	C	-5.23585600	-6.32476600	1.36206100	C	3.40080200	-1.05448000	-2.87594000
C	7.34146400	-1.96676000	0.00830100	H	-4.18183000	-6.59430000	3.22721900	C	7.31220700	-2.09469800	0.16668400
H	5.96657900	-0.82420700	-1.15901200	H	-6.08942300	-5.79969500	-0.55032500	H	5.93030200	-0.92577600	1.29894900
C	6.71985900	-2.43540600	2.29487600	H	-5.87431000	-7.19242400	1.50214000	C	6.72746000	-2.57436400	-2.12732400
C	4.54722700	-1.61254900	3.16992900	C	-0.87233700	3.94111900	-0.74179200	C	4.58218000	-1.73161800	-3.04960000
H	2.61702600	-0.87642900	3.75722200	H	-0.19456200	3.95149600	0.11850300	H	2.67321500	-0.97548300	-3.67930300
C	7.62991200	-2.55653200	1.26443500	H	-0.27636400	3.64072800	-1.61105100	C	7.61656500	-2.69828500	-1.07909800
H	8.04974800	-2.06953200	-0.81000500	H	-1.25535900	4.95113700	-0.90651900	H	8.00397000	-2.19921400	0.99878400
H	6.92145200	-2.88991900	3.26282600	C	1.08335800	-2.20909200	0.76953400	H	6.94147400	-3.03913400	-3.08769500
H	4.76449100	-2.07058100	4.13275500	H	1.54519800	-1.35715000	1.27939100	H	4.81172400	-2.20008600	-4.00452600
H	8.55760900	-3.10336700	1.40986900	H	1.35055300	-3.13842800	1.27731400	H	8.53995400	-3.25760600	-1.20283100
O	1.86513300	0.26782000	1.60913900	H	1.51095700	-2.23811600	-0.23920900	O	1.89606000	0.19617000	-1.55474700
O	1.80470600	-1.07463100	-0.98870000	Cu	0.22369700	1.02023500	-0.22409900	O	1.76750700	-1.12740700	1.05179600

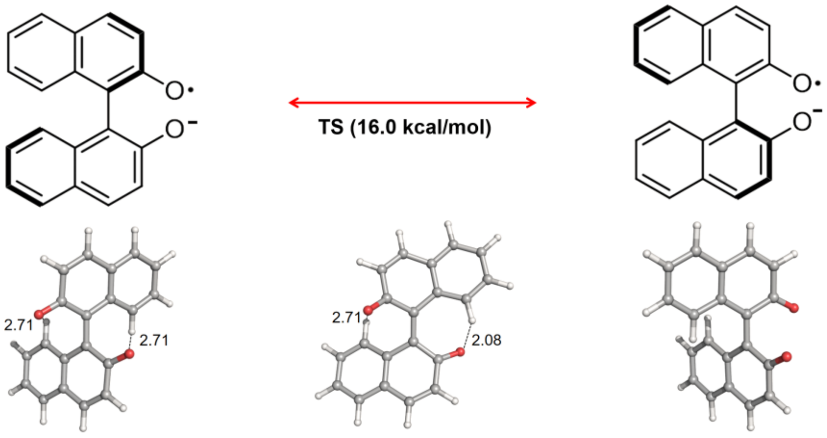

$E_{bb\text{-}solv}$	-920.18316	-920.15839	-920.18076
$E_{Gibbs\text{-}correct}$	0.21322	0.21400	0.21346
G_{Total}	-919.96994	-919.94439	-919.96730
**Cartesian Coordinates**	C -0.61994300 1.70670600 -1.01721400	C 0.57350900 2.03238100 -0.18192500	C 1.19349300 1.83077700 0.99634300
	C -0.61348800 0.39151500 -0.36802500	C 0.564234000.56287300 -0.00432600	C 0.70684700 0.71932900 0.18040200
	C -1.84174200 -0.13139800 0.17891700	C 1.90288300 -0.03941500 -0.15921000	C 1.61506500 -0.31529400 -0.23040800
	C -3.06679200 0.60587600 0.06750300	C 3.07890500 0.70370800 0.16949000	C 3.00061300 -0.25950700 0.14802500
	C -3.05410400 1.88115500 -0.59469500	C 2.94319200 2.09156200 0.53105800	C 3.46853800 0.86470100 0.91128600
	C -1.90294700 2.40677400 -1.08811900	C 1.75847200 2.72541200 0.33332800	C 2.62710100 1.86354000 1.28784000
	H -0.96824500 -1.90106100 1.06773600	H 1.2430010 -1.87231000 -1.09438100	H 0.19517100 -1.43416300 -1.42686400
	C -1.88489300 -1.34097400 0.92781300	C 2.10246600 -1.31550000 -0.73919600	C 1.22011300 -1.39375300 -1.07608400
	C -4.25566000 0.10015200 0.63838700	C 4.35443600 0.11091700 0.05205800	C 3.88791000 -1.27763200 -0.26402800
	H -3.99163300 2.42919800 -0.67772800	H 3.82138400 2.63549600 0.87436600	H4.52469600 0.90408300 1.17424200
	H -1.88667700 3.37653300 -1.57857800	H 1.64904800 3.79747700 0.47679300	H 2.98043200 2.71684900 1.86070600
	C -4.26707000 -1.10018000 1.33158500	C 4.50821700 -1.16922400 -0.45974800	C 3.45755800 -2.33803800 -1.04760700
	C -3.06041800 -1.81707000 1.47988800	C 3.36489100 -1.86902200 -0.88910300	C 2.10881400 -2.38079200 -1.46289600
	H -5.17061600 0.67978100 0.53165200	H 5.22653700 0.69449700 0.34049300	H 4.93068700 -1.21150200 0.04053600
	H -5.18877100 -1.47784300 1.76614300	H 5.49635300 -1.61112400 -0.55681300	H 4.15290800 -3.11436000 -1.35508000
	H -3.05038500 -2.75041000 2.03730600	H 3.46678600 -2.85032400 -1.34588200	H 1.76496000 -3.18940400 -2.10317000
	C 0.61342600 -0.39170600 -0.36813400	C -0.58593400 -0.25220700 0.22324700	C -0.70684500 0.71933700 -0.18038300
	C 1.84166800 0.13123600 0.17877200	C -2.01064400 0.10470000 0.07475200	C -1.61507100 -0.31528200 0.23042000
	C 0.62001100 -1.70667800 -1.01778200	C -0.37743300 -1.63424600 0.73865100	C -1.19348100 1.83080400 -0.99630500
	C 1.88475100 1.34077200 0.92772500	C -2.55064500 1.39892300 0.27603700	C -1.22013200 -1.39374200 1.07609900
	C 3.06679000 -0.60587900 0.06714700	C-2.95938900 -0.93547700 -0.21686700	C-3.00061500 -0.25948900 -0.14802600
	C 1.90309300 -2.40658300 -1.08892500	C -1.32230700 -2.65398900 0.28461600	C -2.62708000 1.86354900 -1.28785200
	C 3.06026400 1.81692700 1.47977800	C-3.90110900 1.67417000 0.10309700	C -2.10884000 -2.38077700 1.46290400
	H 0.96805800 1.90078200 1.06769700	H -1.89840300 2.20441000 0.57055300	H -0.19519200 -1.43415600 1.42688900
	C 4.25563600 -0.10011300 0.63803700	C-4.31894700 -0.62623200 -0.43550400	C -3.88792000 -1.27761100 0.26401700
	C 3.05420300 -1.88100900 -0.59533700	C-2.54183400 -2.31505200 -0.20448400	C -3.46852400 0.86471300 -0.91130600
	H 1.88692500 -3.37618000 -1.57970600	H -1.02166200 -3.68887700 0.42598900	H -2.98039800 2.71684800 -1.86074000
	C 4.26696700 1.10014100 1.33137700	C -4.7986950 0.66706500 -0.29057300	C -3.45758000 -2.33801800 1.04760200
	H 3.05018000 2.75022600 2.03726500	H -4.25943700 2.68605100 0.27615800	H -1.76499500 -3.18939000 2.10318100
	H 5.17065000 -0.67962500 0.53115900	H -4.99919800 -1.43753700 -0.68734400	H-4.93069300 -1.21147900 -0.04055900
	H 3.99178900 -2.42892400 -0.67856100	H -3.25701800 -3.07803200 -0.50692300	H -4.52467600 0.90409100 -1.17428500
	H 5.18865700 1.47785000 1.76591800	H -5.85059900 0.88955600 -0.44922400	H -4.15293500 -3.11433700 1.35506900
	O -0.39233800 -2.22448300 -1.55939700	O 0.54690500 -1.95268100 1.51973900	O -0.43024000 2.68098700 -1.52302600
	O 0.39253200 2.22469500 -1.55841400	O -0.33720500 2.71051000 -0.71426000	O 0.43027100 2.68101600 1.52300200

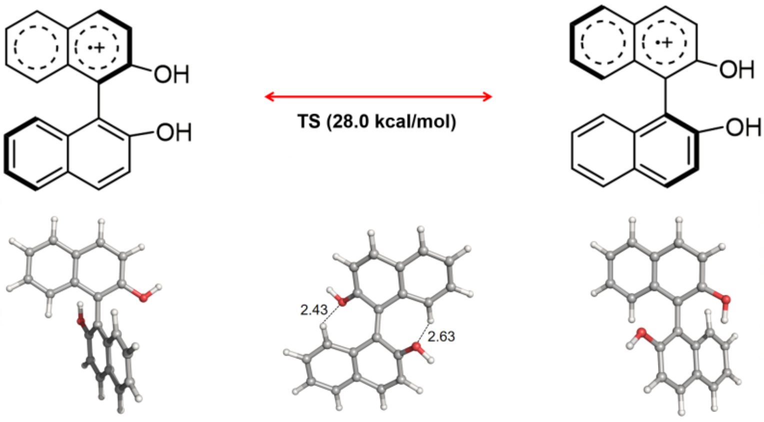

$\boldsymbol{E}_{\mathbf{bb\text{-}solv}}$	-921.10530	-921.06244	-921.10530
$\boldsymbol{E}_{\mathbf{Gibbs\text{-}correct}}$	0.23919	0.24103	0.23919
$\boldsymbol{G}_{\mathbf{Total}}$	-920.86611	-920.82141	-920.86611
**Cartesian Coordinates**	C 0.69293900 1.64187800 1.01462700<br>C 0.62418600 0.39733600 0.35156200<br>C 1.81931300 -0.13074300 -0.25361900<br>C 3.05875000 0.57365200 -0.10513600<br>C 3.07221700 1.80186100 0.61543800<br>C 1.92105800 2.32948700 1.15092100<br>H 0.86882000 -1.82466200 -1.23195900<br>C 1.80638100 -1.31069800 -1.04549800<br>C 4.23176800 0.05628200 -0.70290900<br>H 4.01352500 2.33097400 0.73245000<br>H 1.94449800 3.26737100 1.69973800<br>C 4.19533800 -1.11981400 -1.43504000<br>C 2.97391800 -1.80003300 -1.60848800<br>H 5.16440300 0.60092400 -0.58627000<br>H 5.10127300 -1.50785800 -1.88919500<br>H 2.94227500 -2.70811900 -2.20234200<br>C -0.63178200 -0.36571200 0.36470800<br>C -1.83010500 0.12446400 -0.24220800<br>C -0.68962500 -1.60575700 1.07760500<br>C -1.83432000 1.28977200 -1.05186900<br>C -3.05518900 -0.60018900 -0.08487000<br>C -1.90140200 -2.31354300 1.22299400<br>C -3.00887400 1.75011300 -1.63462300<br>H -0.90503400 1.81991900 -1.22939900<br>C -4.22875200 -0.12025700 -0.69403300<br>C -3.05375100 -1.81758300 0.66399800<br>H -1.89348700 -3.23287700 1.79792600<br>C -4.21086600 1.05011900 -1.45438700<br>H -2.99126500 2.64976400 -2.24123500<br>H -5.15424400 -0.67548300 -0.57137100<br>H -3.98853400 -2.35745000 0.78404900<br>H -5.12441300 1.40859300 -1.91766400<br>O 0.36303600 -2.13307500 1.71423400<br>H 1.17019900 -1.60764300 1.57873500<br>O -0.43682800 2.12085100 1.57733800<br>H -0.26489900 2.95053000 2.04892900	C 0.45882200 1.83499300 -0.39364200 C<br>0.56767900 0.41740700 -0.11512900<br>C 1.97104900 -0.04502600 -0.12957500<br>C 3.00032000 0.85937700 0.27792600<br>C 2.68682400 .24136600 0.44188100<br>C 1.46917000 2.72599700 0.01922600<br>H 1.64810700 -1.94741400 -1.10286600<br>C 2.37467400 -1.29259000 -0.63886800<br>C 4.34200200 0.42221300 0.35772500<br>H 3.45739400 2.92677900 0.78215800<br>H 1.28317700 3.79552000 -0.03416800<br>C 4.69516700 -0.84922700 -0.06669900<br>C 3.70894100 -1.68612200 -0.61213100<br>H 5.09796500 1.11856100 0.70932600<br>H 5.72926900 -1.17535000 -0.02547000<br>H 3.98752700 -2.64712600 -1.03352100<br>C -0.56765900 -0.41805100 0.11466500<br>C -1.97085300 0.04490900 0.12957800<br>C -0.45916700 -1.83573000 0.39280200<br>C -2.37373500 1.29257900 0.63923100<br>C -3.00063900 -0.85896400 -0.27768700<br>C -1.47022100 -2.72623600 -0.01943000<br>C -3.70782500 1.68673600 0.61297400<br>H -1.64666200 1.94690300 1.10319300<br>C -4.34219500 -0.42126600 -0.35682200<br>C -2.68778900 -2.24107700 -0.44168300<br>H -1.28476600 -3.79584200 0.03395300<br>C -4.69462400 0.85028700 0.06785800<br>H -3.98582000 2.64785700 1.03449200<br>H -5.09860600 -1.11732900 -0.70805200<br>H -3.45875800 -2.92616600 -0.78171400<br>H -5.72860900 1.17683500 0.02713600<br>O -0.60478400 2.28243700 -1.07080100<br>H -0.56949400 3.24717900 -1.18155400<br>O 0.60485000 -2.28384700 1.06884800<br>H 0.56911000 -3.24858300 1.17950600	C 1.17302700 1.72049800 -0.98316800<br>C 0.70549800 0.67445000 -0.16384400<br>C1.63326500 -0.34445300 0.26067600<br>C 2.99265100 -0.27916500 -0.18877900<br>C 3.39517700 0.78909200 -1.03798500<br>C 2.51317500 1.77068000 -1.42571800<br>H 0.25775400 -1.44402300 1.53129700<br>C1.27256900 -1.38546100 1.15427400<br>C 3.92191800 -1.25972800 0.24111400<br>H 4.42450400 0.82478200 -1.38281500<br>H 2.82703500 2.57426700 -2.08656000<br>C 3.53390300 -2.27342600 1.09671100<br>C2.20087400 -2.32855500 1.55718700<br>H 4.94897600 -1.19411000 -0.10675100<br>H4.25185000 -3.01831400 1.42421000<br>H1.90027100 -3.11673100 2.24053900<br>C -0.72311300 0.65273300 0.19311000<br>C-1.61952400 -0.37510400 -0.23754700<br>C-1.24730900 1.71340400 1.00540800<br>C-1.21140600 -1.41321900 -1.11677800<br>C-2.99305900 -0.33147400 0.17147500<br>C-2.60304500 1.75592800 1.38813400<br>C-2.10861300 -2.38647100 -1.53820400<br>H-0.18656700 -1.44370800 -1.46783300<br>C-3.88194300 -1.32628500 -0.27099600<br>C -3.44905700 0.75143600 0.98768800<br>H -2.93988100 2.57505500 2.01370400<br>C -3.44446500 -2.35167700 -1.11198600<br>H -1.77225200 -3.17292800 -2.20598500<br>H -4.92170200 -1.28350500 0.03964800<br>H -4.49175400 0.77131500 1.29102100<br>H-4.14296600 -3.11204400 -1.44584300<br>O -0.46771200 2.67241600 1.50415100<br>H 0.45913700 2.55667900 1.23004100<br>O 0.26113200 2.64155500 -1.38359200<br>H 0.67509000 3.33228000 -1.92376700

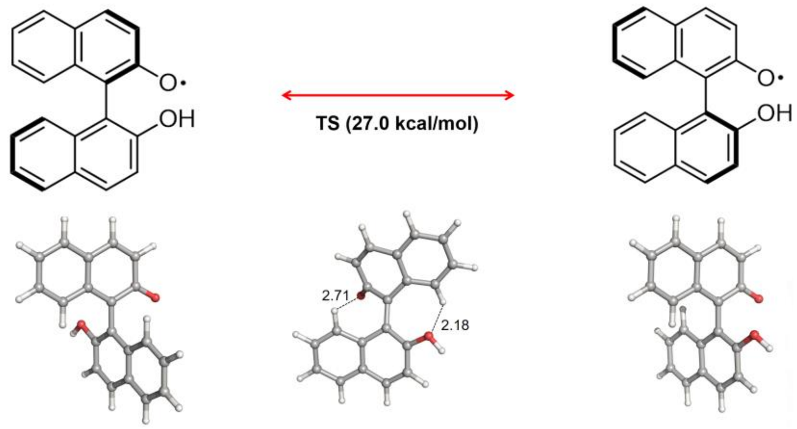

***E*bb-solv**	-920.68460	-920.64356	-920.68460
***E*Gibbs-correct**	0.22514	0.22713	0.22513
***G*Total**	-920.45946	-920.41643	-920.45947

Cartesian Coordinates

C	0.76137800	-1.56865700	-1.26173700	C	-0.37673800	-1.62030500	-0.76556700	C	1.11918700	1.64008500	1.23081900
C	0.65310400	-0.33929300	-0.47120500	C	-0.58189200	-0.22198200	-0.25871500	C	0.70839800	0.63960600	0.24386600
C	1.78551000	0.15523200	0.23465400	C	-2.01409300	0.09959200	-0.11084800	C	1.64765900	-0.30193500	-0.25451600
C	3.04040400	-0.54072800	0.14705200	C	-2.87810200	-0.94972100	0.34530300	C	3.00642100	-0.27112700	0.21681800
C	3.13494400	-1.74034800	-0.64596300	C	-2.38714100	-2.31487400	0.42889400	C	3.39997400	0.72686000	1.18144300
C	2.06241900	-2.23609100	-1.30671200	C	-1.21486100	-2.65817300	-0.14938600	C	2.51620100	1.63772700	1.66029900
H	0.77140900	1.83338300	1.14714600	H	-2.04045300	2.08075700	-0.96115800	H	0.27052600	-1.30763500	-1.59389900
C	1.71979100	1.31689900	1.05479900	C	-2.62850400	1.31269400	-0.48394700	C	1.28969500	-1.28322000	-1.22460300
C	4.15333700	-0.04643300	0.84746700	C	-4.23636300	-0.69010600	0.59027500	C	3.92852900	-1.20880600	-0.27912800
H	4.09776500	-2.24374500	-0.69385100	H	-3.04297500	-3.07050800	0.85525400	H	4.43427500	0.73145100	1.51746400
H	2.11599800	-3.14094700	-1.90325800	H	-0.88689800	3.68811000	-0.25244100	H	2.80360300	2.39206500	2.38611200
C	4.05859700	1.09733900	1.62860200	C	-4.79540800	0.55060700	0.29582900	C	3.54619900	-2.16097600	-1.21910400
C	2.83042600	1.77733800	1.73407100	C	-3.98943500	1.53541400	-0.28675700	C	2.21914700	-2.19339000	-1.69381000
H	5.09796900	-0.57848300	0.77214800	H	-4.86057900	1.49306600	0.97414000	H	4.95363400	-1.18021600	0.08079400
H	4.92968300	1.46296200	2.16394500	H	-5.85074100	0.73364400	0.47489600	H	4.27275800	-2.87741200	-1.59097800
H	2.75541000	2.66448100	2.35551900	H	-4.42355700	2.48121000	-0.59853300	H	1.92603000	-2.93397600	-2.43178100
C	-0.65634600	0.36203100	-0.45482000	C	0.57968100	0.58335500	-0.00723600	C	-0.70407400	0.65604800	-0.21183400
C	-1.79938800	-0.19446900	0.20830600	C	1.91282500	-0.04393000	0.13514800	C	-1.65467800	-0.30955400	0.24946300
C	-0.79140400	1.57769700	-1.11544600	C	0.60798000	2.00192100	0.15722100	C	-1.11974100	1.65242300	-1.08265200
C	-1.73785500	-1.41186400	0.94072400	C	2.09061800	-1.36813400	0.60242500	C	-1.30610400	-1.34662000	1.16121500
C	-3.05254700	0.50128600	0.16894400	C	3.10163100	0.71908100	-0.08956300	C	-3.01421500	-0.23691300	-0.20502400
C	-2.03203400	2.26022200	-1.15578200	C	1.78911500	2.74763100	-0.05028500	C	-2.46287000	1.72666300	-1.52945000
C	-2.84835000	-1.91529700	1.57927900	C	3.34412300	-1.94706000	0.70314100	C	-2.24417600	-2.26367500	1.58675200
H	-0.79762800	-1.94830100	0.99515300	H	1.23260800	-1.94527100	0.92319000	H	-0.28682500	-1.41074300	1.52685700
C	-4.18018800	-0.04667400	0.83627800	C	4.37507800	0.09622100	-0.03034400	C	-3.95610600	-1.19824400	0.25296500
C	-3.13380900	1.73175000	-0.53145300	C	2.99859600	2.12881700	-0.25458500	C	-3.38556800	0.80370500	-1.09777200
H	-2.10133500	3.20379800	-1.69327200	H	1.71976400	3.83343300	-0.05390500	H	-2.75148700	2.51753500	-2.21807200
C	-4.08609900	-1.23168200	1.52733300	C	4.50055900	-1.22676600	0.34239700	C	-3.58341300	-2.19497500	1.12896400
H	-2.77392300	-2.84897600	2.12915900	H	3.43213700	-2.96408700	1.07414100	H	-1.95397700	-3.04511700	2.28348900
H	-5.12219300	0.49401300	0.79166500	H	5.25590000	0.69609900	-0.24455000	H	-4.98223400	-1.12959000	-0.09987600
H	-4.08417900	2.25719100	-0.56662800	H	3.89153500	2.71457400	-0.45222400	H	-4.41479000	0.86278200	-1.44140700
H	-4.95391900	-1.64231900	2.03444000	H	5.47902800	-1.69411100	0.40012200	H	-4.31175300	-2.92312300	1.47367900
O	0.30536600	2.09353800	-1.74953000	O	0.42936300	-1.88736600	-1.66318200	O	-0.18043400	2.54047700	-1.52834400
O	-0.21555800	-2.02791300	-1.89075400	O	-0.52354400	2.67328600	0.49209700	O	0.29798200	2.45638300	1.71027800
H	0.05514300	2.91239400	-2.19624000	H	-0.33832600	3.62238700	0.54934200	H	-0.60293900	3.24700400	-2.03610900

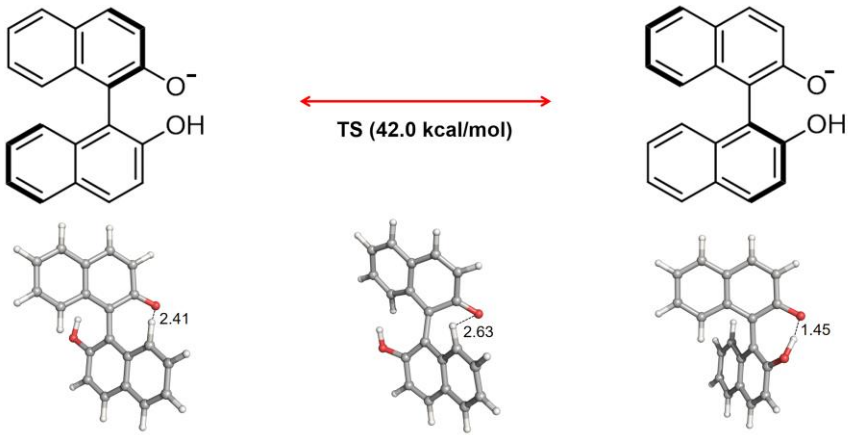

**$E_{bb\text{-}solv}$**	-920.83029	-920.76422	-920.82672
**$E_{Gibbs\text{-}correct}$**	0.22688	0.22782	0.22680
**G_{Total}**	-920.60341	-920.53640	-920.59992
**Cartesian Coordinates**	C 0.68260300 1.78742500 1.00586700	C -0.38035300 -1.57093900 0.89553400	C 1.34825300 1.89564300 0.78866200
	C 0.64973800 0.42104400 0.51072500	C -0.59973100 -0.23076200 0.33885500	C 0.74010300 0.77660800 0.14962300
	C 1.79495700 -0.13530200 -0.15418500	C -1.99901200 0.08054500 0.09567500	C 1.56467800 -0.32121600 -0.27666300
	C 3.05153100 0.57756900 -0.16780600	C -2.91771900 -0.97617900 -0.27901900	C 2.95115300 -0.38303000 0.11038000
	C 3.10107600 1.87478300 0.42924600	C -2.49021100 -2.33763400 -0.15674800	C 3.49226400 0.68796600 0.87976800
	C 1.99184400 2.44525300 0.97145600	C -1.31086600 -2.61645300 0.46104100	C 2.73061000 1.78273900 1.18722200
	H 0.84751500 -1.92883800 -0.93336300	H -2.12164800 2.08380000 0.93070400	H 0.06622300 -1.33866500 -1.47541000
	C 1.78154500 -1.38375300 -0.85738000	C -2.63172000 1.34501300 0.32543400	C 1.08975300 -1.37301600 -1.12031800
	C 4.18725300 0.01486800 -0.78763000	C -4.23870900 -0.67552700 -0.67056500	C 3.75432200 -1.48043800 -0.29267800
	H 4.05337300 2.40664700 0.42507100	H -3.15455700 -3.13233600 -0.49310700	H 4.53327800 0.63382800 1.19474500
	H 2.02078200 3.44345600 1.40295400	H -1.00120400 -3.63827900 0.66761800	H 3.14615700 2.62332500 1.73751400
	C 4.14056300 -1.21225500 -1.42403100	C -4.75622000 0.61134900 -0.60085800	C 3.24754400 -2.49554100 -1.08164700
	C 2.90901600 -1.89990300 -1.46388400	C -3.94852800 1.61112500 -0.02994700	C 1.89938600 -2.42253100 -1.50789900
	H 5.11582000 0.58468800 -0.76333100	H -4.87197200 -1.49859300 -0.99782800	H 4.79501300 -1.50201300 0.02643600
	H 5.02523100 -1.62998800 -1.89779500	H -5.77969700 0.81951900 -0.89950100	H 3.87662700 -3.32778200 -1.38623100
	H 2.83949100 -2.85126900 -1.98828900	H -4.36496500 2.59257800 0.18630100	H 1.49514900 -3.19726800 -2.15537700
	C -0.65202400 -0.30987900 0.52371200	C 0.59584700 0.62598200 0.13042300	C -0.72930900 0.78425900 -0.11885300
	C -1.85247200 0.16679900 -0.11153700	C 1.88747500 -0.02918200 -0.14077800	C -1.57535800 -0.30834100 0.29520500
	C -0.69331200 -1.59708700 1.06853300	C 0.68916900 2.03253800 0.04370500	C -1.34226200 1.87163900 -0.76412000
	C -1.91010200 1.42007700 -0.78625400	C 1.97442200 -1.35077400 -0.66125100	C -1.11199600 -1.35419800 1.14799700
	C -3.04289500 -0.64008300 -0.11734200	C 3.13374100 0.66861200 0.01141600	C-2.95072400 -0.36481100 -0.12270300
	C -1.86044700 -2.39564300 1.05888500	C 1.91639400 2.72431300 0.19917100	C-2.70285400 1.80296400 -1.17701400
	C -3.06200800 1.85270400 -1.40363600	C 3.18181600 -1.97901300 -0.89097900	C-1.92803700 -2.40202500 1.52228800
	H -1.02751600 2.04291700 -0.78987800	H 1.06316200 -1.87682800 -0.91311100	H -0.09369800 -1.31336600 1.51645300
	C -4.21974200 -0.15689600 -0.75212200	C 4.36702500 -0.01665700 -0.17166000	C -3.76203000 -1.46650500 0.26425500
	C -3.01325500 -1.92570700 0.48182200	C 3.11637700 2.06559000 0.2640530	C -3.47846800 0.70584700 -0.89519500
	H -1.81121200 -3.37551000 1.52497400	H 1.85891100 3.80748500 0.26412500	H -3.10204100 2.65480400 -1.72064600
	C -4.23778200 1.06422500 -1.38370000	C 4.40182600 -1.32578900 -0.60052000	C -3.26732700 -2.47429100 1.06559100
	H -3.06989000 2.81604900 -1.90797500	H 3.18928600 -2.98499700 -1.30321900	H -1.53944800 -3.17572300 2.17978300
	H -5.10928200 -0.78444000 -0.73462300	H 5.29016600 0.53413500 -0.00221200	H -4.79551300 -1.49181300 -0.07587800
	H -3.91453600 -2.53501200 0.47185900	H 4.04697400 2.60436200 0.42184800	H -4.51327100 0.66041000 -1.22771500
	H -5.14370800 1.42135500 -1.86804500	H 5.35002600 -1.83576700 -0.75057400	H -3.90085200 -3.30747100 1.35842400
	O 0.38599200 -2.14056300 1.70787300	O -0.35158300 2.90173400 -0.17766300	O -0.67784800 3.01125500 -1.03307000
	O -0.30899400 2.39549300 1.48369700	O 0.56400100 -1.88033900 1.67110700	O 0.71168200 3.00769600 1.02127600
	H 1.08142100 -1.45892000 1.68144400	H -1.14996600 2.41682500 -0.43649300	H -0.05649000 3.15979400 -0.20115500

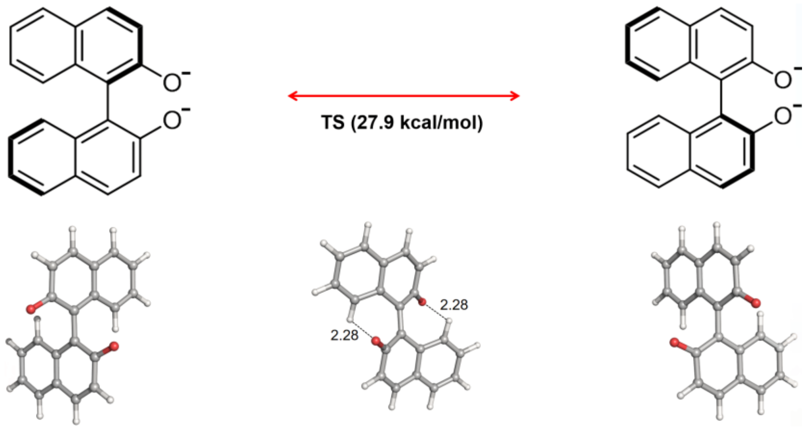

$E_{\text{bb-solv}}$	-920.24713	-920.20062	-920.24712
$E_{\text{Gibbs-correct}}$	0.21672	0.21471	0.21671
G_{Total}	-920.03041	-919.98591	-920.03041
**Cartesian Coordinates**	C -0.82389000 -1.57067800 1.31763600	C 0.47855500 -1.86556900 0.40269700	C 1.19349300 1.83077700 0.99634300
	C -0.66277200 -0.34675400 0.57192800	C 0.58514200 -0.44304400 0.06036400	C 0.70684700 0.71932900 0.18040200
	C -1.74666900 0.18860400 -0.17361800	C 1.96136100 0.03590100 0.08009600	C 1.61506500 -0.31529400 -0.23040800
	C -3.04009900 -0.46292800 -0.20190300	C 3.07007000 -0.84673300 -0.21929500	C 3.00061300 -0.25950700 0.14802500
	C -3.19362600 -1.66661700 0.54382900	C 2.80904400 -2.23861100 -0.38395900	C 3.46853800 0.86470100 0.91128600
	C -2.14625600 -2.18724700 1.25850900	C 1.57197200 -2.71920200 -0.04888900	C 2.62710100 1.86354000 1.28784000
	H -0.66781000 1.89745700 -0.95738300	H 1.57566800 2.00254500 0.91110100	H 0.19517100 -1.43416300 -1.42686400
	C -1.62456300 1.38630000 -0.95497800	C 2.33727100 1.34404100 0.52093700	C 1.22011300 -1.39375300 -1.07608400
	C -4.10117900 0.08966800 -0.96000400	C 4.39625500 -0.35008500 -0.27353700	C 3.88791000 -1.27763200 -0.26402800
	H -4.16282100 -2.16794100 0.53707400	H 3.61114300 -2.90436100 -0.70348100	H 4.52469600 0.90408300 1.17424200
	H -2.26503200 -3.11061200 1.82419800	H 1.36146500 -3.78782800 -0.04583000	H 2.98043200 2.71684900 1.86070600
	C -3.94335400 1.25311700 -1.69517000	C 4.69950400 0.95784600 0.06483900	C 3.45755800 -2.33803800 -1.04760700
	C -2.67801500 1.89882200 -1.68417400	C 3.64660300 1.79015000 0.51621200	C 2.10881400 -2.38079200 -1.46289600
	H -5.06191700 -0.42618000 -0.95463300	H 5.19024000 -1.04324200 -0.55244300	H 4.93068700 -1.21150200 0.04053600
	H -4.76957400 1.66497900 -2.27038400	H 5.72449300 1.32172700 0.03312800	H 4.15290800 -3.11436000 -1.35508000
	H -2.53687700 2.81442000 -2.25634600	H 3.86578900 2.79487000 0.87451600	H 1.76496000 -3.18940400 -2.10317000
	C 0.66280300 0.34687700 0.57183400	C -0.58524000 0.44235100 -0.06140800	C -0.70684500 0.71933700 -0.18038300
	C 1.74666400 -0.18864700 -0.17364300	C -1.96166100 0.03601700 -0.08029000	C -1.61507100 0.31528200 0.23042000
	C 0.82396800 1.57094800 1.31729400	C -0.47805100 1.86471300 -0.40428100	C -1.19348100 1.83080400 -0.99630500
	C 1.62452200 -1.38652700 -0.95471600	C -2.33846300 -1.34430700 -0.51986600	C -1.22013200 -1.39374200 1.07609900
	C 3.04008800 0.46288600 -0.20215000	C -3.06983500 0.84735300 0.21909600	C -3.00061500 -0.25948900 -0.14802600
	C 2.14631400 2.18753600 1.25791500	C -1.57099000 2.71909200 0.04698800	C -2.62708000 1.86354900 -1.28785200
	C 2.67793900 -1.89921800 -1.68384300	C -3.64794600 -1.78997800 -0.51356300	C -2.10884000 -2.38077700 1.46290400
	H 0.66777000 -1.89768500 -0.95695200	H -1.57746100 -2.00354000 -0.90989600	H -0.19519200 -1.43415600 1.42688900
	C 4.10112900 -0.08988000 -0.96018200	C -4.39617900 0.35130600 0.27462400	C -3.88792000 -1.27761100 0.26401700
	C 3.19364400 1.66675600 0.54328500	C -2.80815700 2.23922500 0.38271800	C -3.46852400 0.86471300 -0.91130600
	H 2.26510800 3.11103100 1.82338700	H -1.359984003.78761400 0.04325000	H -2.98039800 2.71684800 -1.86074000
	C 3.94327300 -1.25350500 -1.69506200	C -4.70018400 -0.95683400 -0.06223600	C -3.45758000 -2.33801800 1.04760200
	H 2.53677300 -2.81494700 -2.25579900	H -3.86775000 -2.79495700 -0.87076400	H -1.76499500 -3.18939000 2.10318100
	H 5.06186200 0.42598100 -0.95499500	H -5.189706001.04502800 0.55344500	H -4.93069300 -1.21147900 -0.04055900
	H 4.16283200 2.16809000 0.53634800	H -3.609879002.90550400 0.70207800	H -4.52467600 0.90409100 -1.17428500
	H 4.76946300 -1.66549400 -2.27022800	H -5.72529700 -1.32026600 -0.02942600	H -4.15293500 -3.11433700 1.35506900
	O -0.09982800 2.13528700 1.99343600	O 0.47962100 2.40265300 -1.04860800	O -0.43024000 2.68098700 -1.52302600
	O 0.09991500 -2.13480900 1.99393800	O -0.47876500 -2.40423800 1.04696200	O 0.43027100 2.68101600 1.52300200

Calculated Energy Penalty for Int2 Separating from Cu(L8)

The feasibility of **Int2** separating from Cu(**L8**) can be evaluated by the binding energies, which is expressed by the equation:

$$\Delta G(\mathbf{Int2}) = G(\mathbf{Cu(L8)\text{-}Int2}) + 2 \times G(\mathrm{Cl}^-) - G(\mathrm{Cu}(\mathbf{L8})\mathrm{Cl}_2) - G(\mathbf{Int2}) \quad (1)$$

The calculated free energies of **Cu(L8)-Int2**, Cl^-, Cu(**L8**)Cl_2, and **Int2** are shown in Table S17, and the value of Δ*G*(**Int2**) is thus 8.2 kcal/mol.

Table S17. Calculated free energies of **Cu(L8)-Int2**, Cl^-, Cu(**L8**)Cl_2, and **Int2** (in a.u.)

***G*(Cu(L8)-Int2)**	***G*(Cl^-)**	***G*(Cu(L8)Cl_2)**	***G*(Int2)**
-2229.67589	-460.38247	-2230.45789	-919.96994

Overlap of the Structures of BINOL and Int2

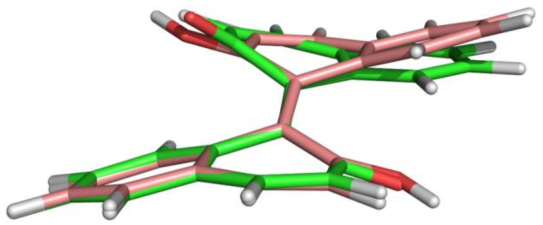

Figure S13. Superposition of the optimized structures of BINOL (C atoms in pink) and **Int2** (C atoms in green).

Interaction Region Indicator (IRI) Analysis

IRI analysis was performed to reveal the weak interactions between the oxygen functional groups and the naphthalene moieties for **Int2** and BINOL **(**Figure S14).

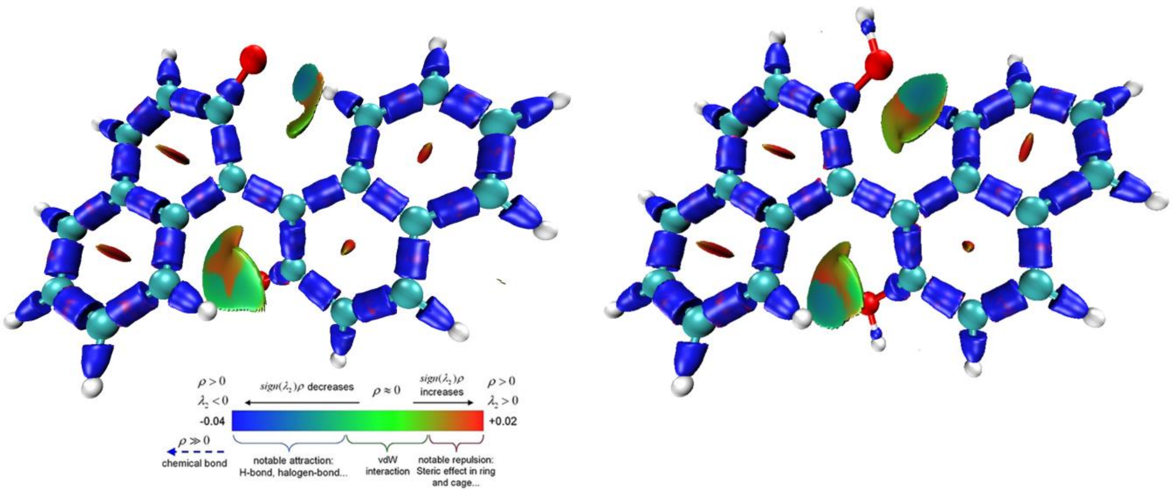

Figure S14. IRI representations mapped for **Int2** and BINOL

References:

1. Li, Y.; Li, Q., Photochemically Reversible and Thermally Stable Axially Chiral Diarylethene Switches. *Org. Lett.* **2012**, *14*, 4362–4365.

2. Narute, S.; Parnes, R.; Toste, F. D.; Pappo, D., Enantioselective Oxidative Homocoupling and Cross-Coupling of 2-Naphthols Catalyzed by Chiral Iron Phosphate Complexes. *J. Am. Chem. Soc.* **2016**, *138*, 16553–16560.

3. Wu, L. Y.; Usman, M.; Liu, W. B., Enantioselective Iron/Bisquinolyldiamine Ligand-Catalyzed Oxidative Coupling Reaction of 2-Naphthols. *Molecules* **2020**, *25*, 852–869.

4. Zhang, J.-W.; Jiang, F.; Chen, Y.-H.; Xiang, S.-H.; Tan, B., Synthesis of structurally diversified BINOLs and NOBINs via palladium-catalyzed C-H arylation with diazoquinones. *Sci. Chin. Chem.* **2021**, *64*, 1515–1521.

5. Fang, S.; Tan, J. P.; Pan, J.; Zhang, H.; Chen, Y.; Ren, X.; Wang, T., Enantiodivergent Kinetic Resolution of 1,1'-Biaryl-2,2'-Diols and Amino Alcohols by Dipeptide-Phosphonium Salt Catalysis Inspired by the Atherton-Todd Reaction. *Angew. Chem. Int. Ed.* **2021**, *60*, 14921–14930.

6. (a) Becke, A. D. Density-functional Thermochemistry. III. The Role of Exact Exchange. *J. Chem. Phys.* **1993**, *98*, 5648-5652. (b) Lee, C.; Yang, W.; Parr, R. G. Development of the Colle-Salvetti Correlation-energy Formula into a Functional of the Electron Density. Phys. Rev. B: Condens. *Matter Mater. Phys.* **1988**, *37*, 785-789.

7. (a) Grimme, S.; Antony, J.; Ehrlich, S.; Krieg, H. A Consistent and Accurate Ab Initio Parametrization of Density Functional Dispersion Correction (DFT-D) for the 94 Elements H-Pu. *J. Chem. Phys.* **2010**, *132*, 154104. (b) Grimme, S.; Ehrlich, S.; Goerigk, L. Effect of the Damping Function in Dispersion Corrected Density Functional Theory. *J. Comput. Chem.* **2011**, *32*, 1456-1465.

8. Frisch, M. J.; Trucks, G. W.; Schlegel, H. B.; Scuseria, G. E.; Robb, M. A.; Cheeseman, J. R.; Scalmani, G.; Barone, V.; Petersson, G. A.; Nakatsuji, H.; Li, X.; Caricato, M.; Marenich, A. V.; Bloino, J.; Janesko, B. G.; Gomperts, R.; Mennucci, B.; Hratchian, H. P.; Ortiz, J. V.; Izmaylov, A. F.; Sonnenberg, J. L.; Williams-Young, D.;

Ding, F.; Lipparini, F.; Egidi, F.; Goings, J.; Peng, B.; Petrone, A.; Henderson, T.; Ranasinghe, D.; Zakrzewski, V. G.; Gao, J.; Rega, N.; Zheng, G.; Liang, W.; Hada, M.; Ehara, M.; Toyota, K.; Fukuda, R.; Hasegawa, J.; Ishida, M.; Nakajima, T.; Honda, Y.; Kitao, O.; Nakai, H.; Vreven, T.; Throssell, K.; Montgomery, J. A., Jr.; Peralta, J. E.; Ogliaro, F.; Bearpark, M. J.; Heyd, J. J.; Brothers, E. N.; Kudin, K. N.; Staroverov, V. N.; Keith, T. A.; Kobayashi, R.; Normand, J.; Raghavachari, K.; Rendell, A. P.; Burant, J. C.; Iyengar, S. S.; Tomasi, J.; Cossi, M.; Millam, J. M.; Klene, M.; Adamo, C.; Cammi, R.; Ochterski, J. W.; Martin, R. L.; Morokuma, K.; Farkas, O.; Foresman, J. B.; Fox, D. J. Gaussian 16, revision C.01; Gaussian, Inc., Wallingford CT, 2016.

9. Lu, T.; Chen, Q. Interaction Region Indicator (IRI): A Simple Real Space Function Clearly Revealing Both Chemical Bonds and Weak Interactions, *Chemistry—Methods*, **2021**,*1*, 231-239.

10. (a) Fernández, I.; Bickelhaupt, F. M. The Activation Strain Model and Molecular Orbital Theory: Understanding and Designing Chemical Reactions. *Chem. Soc. Rev.* **2014**, *43*, 4953−4967. (b) Bickelhaupt, F. M.; Houk, K. N. Analyzing Reaction Rates with the Distortion/Interaction-Activation Strain Model. *Angew. Chem. Int. Ed.* **2017**, *56*, 10070−10086.

NMR spectra:

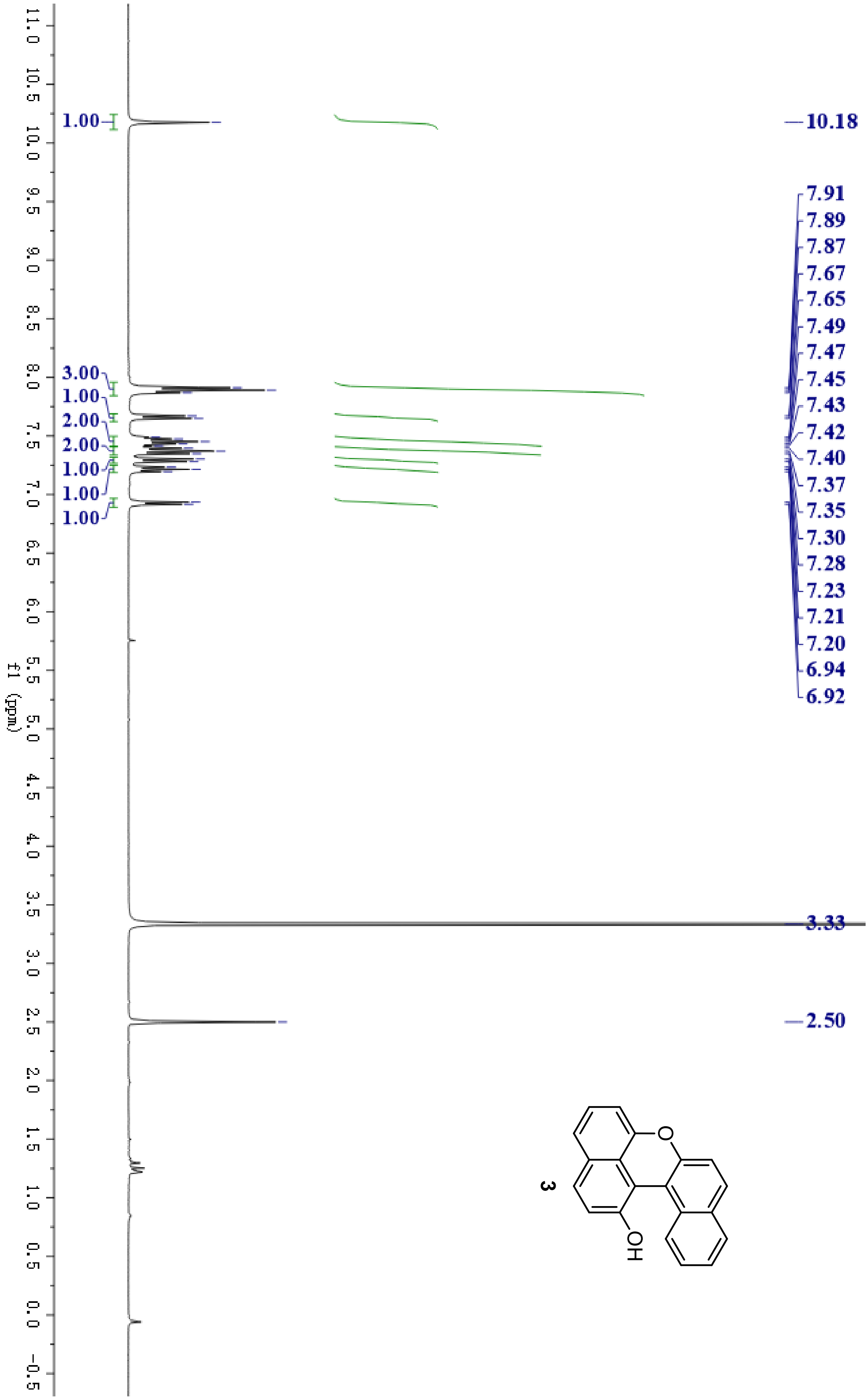
10.18
7.91
7.89
7.87
7.67
7.65
7.49
7.47
7.45
7.43
7.42
7.40
7.37
7.35
7.30
7.28
7.23
7.21
7.20
6.94
6.92
3.33
2.50
1.00
3.00
1.00
2.00
2.00
1.00
1.00
1.00
f1 (ppm)
OH
O
3

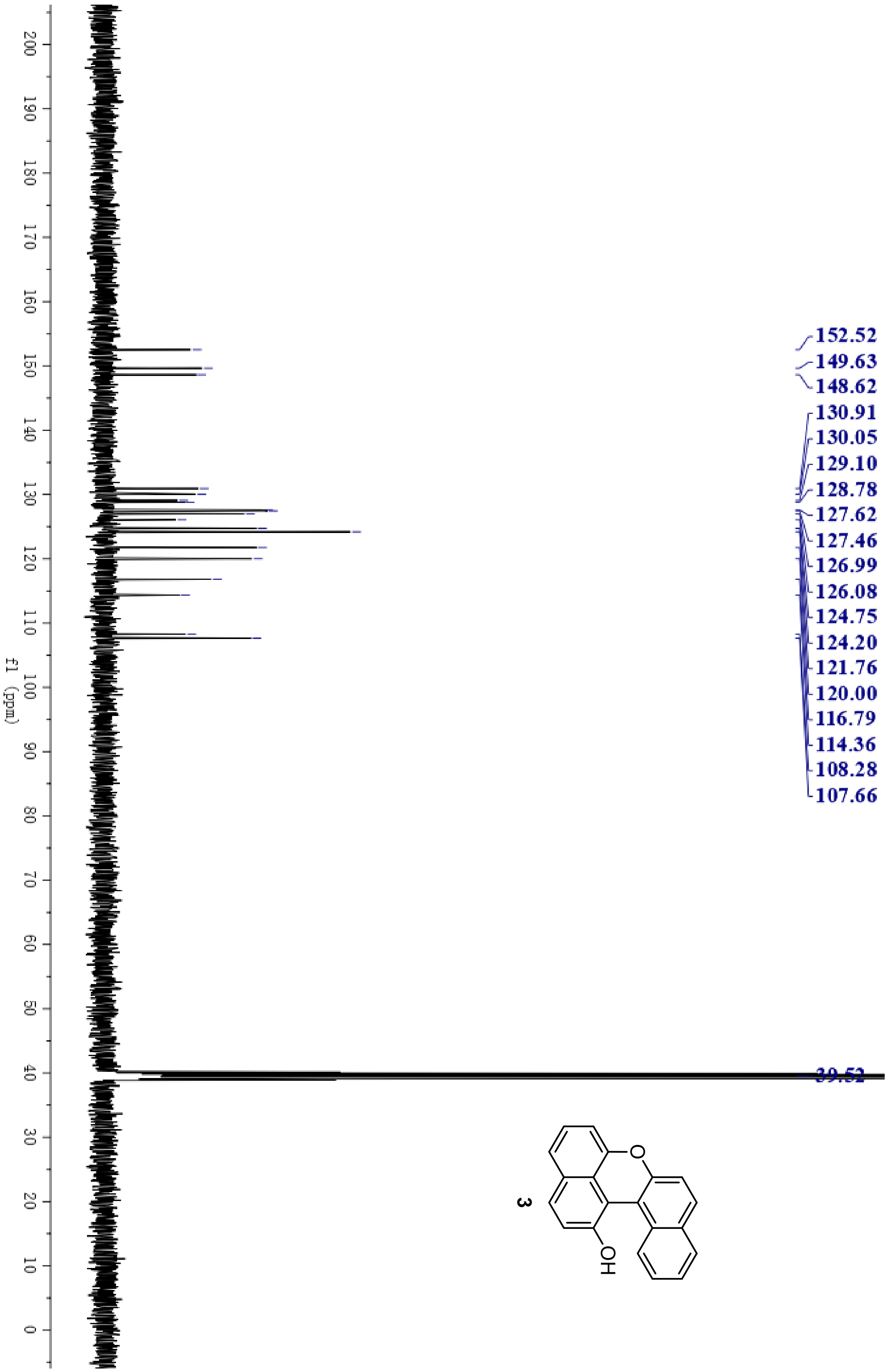

152.52
149.63
148.62
130.91
130.05
129.10
128.78
127.62
127.46
126.99
126.08
124.75
124.20
121.76
120.00
116.79
114.36
108.28
107.66
39.52
f1 (ppm)
200
190
180
170
160
150
140
130
120
110
100
90
80
70
60
50
40
30
20
10
0
3
O
OH

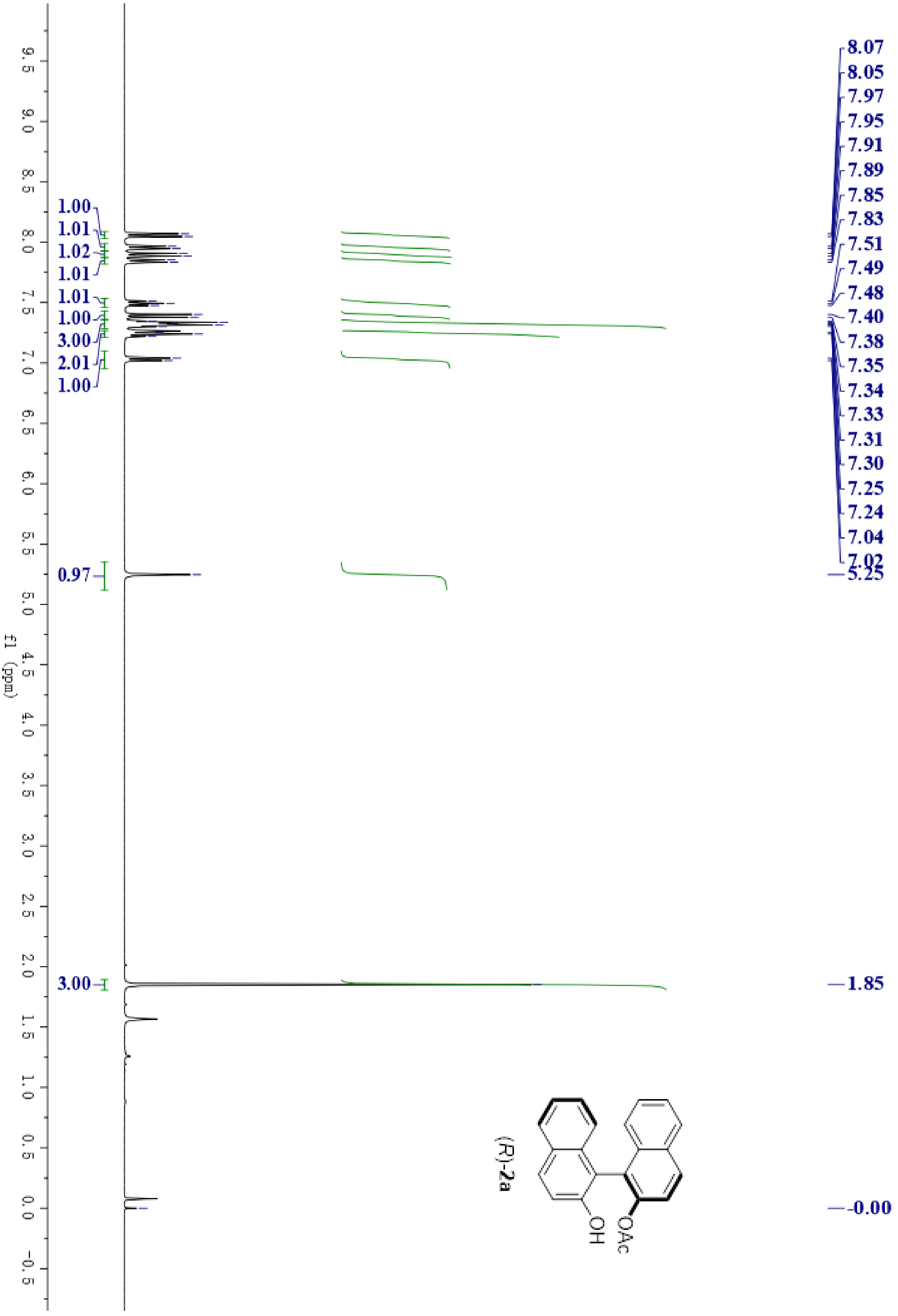
8.07
8.05
7.97
7.95
7.91
7.89
7.85
7.83
7.51
7.49
7.48
7.40
7.38
7.35
7.34
7.33
7.31
7.30
7.25
7.24
7.04
7.02
5.25
1.85
-0.00
1.00
1.01
1.02
1.01
1.01
1.00
3.00
2.01
1.00
0.97
3.00
f1 (ppm)
OAc
OH
(R)-2a

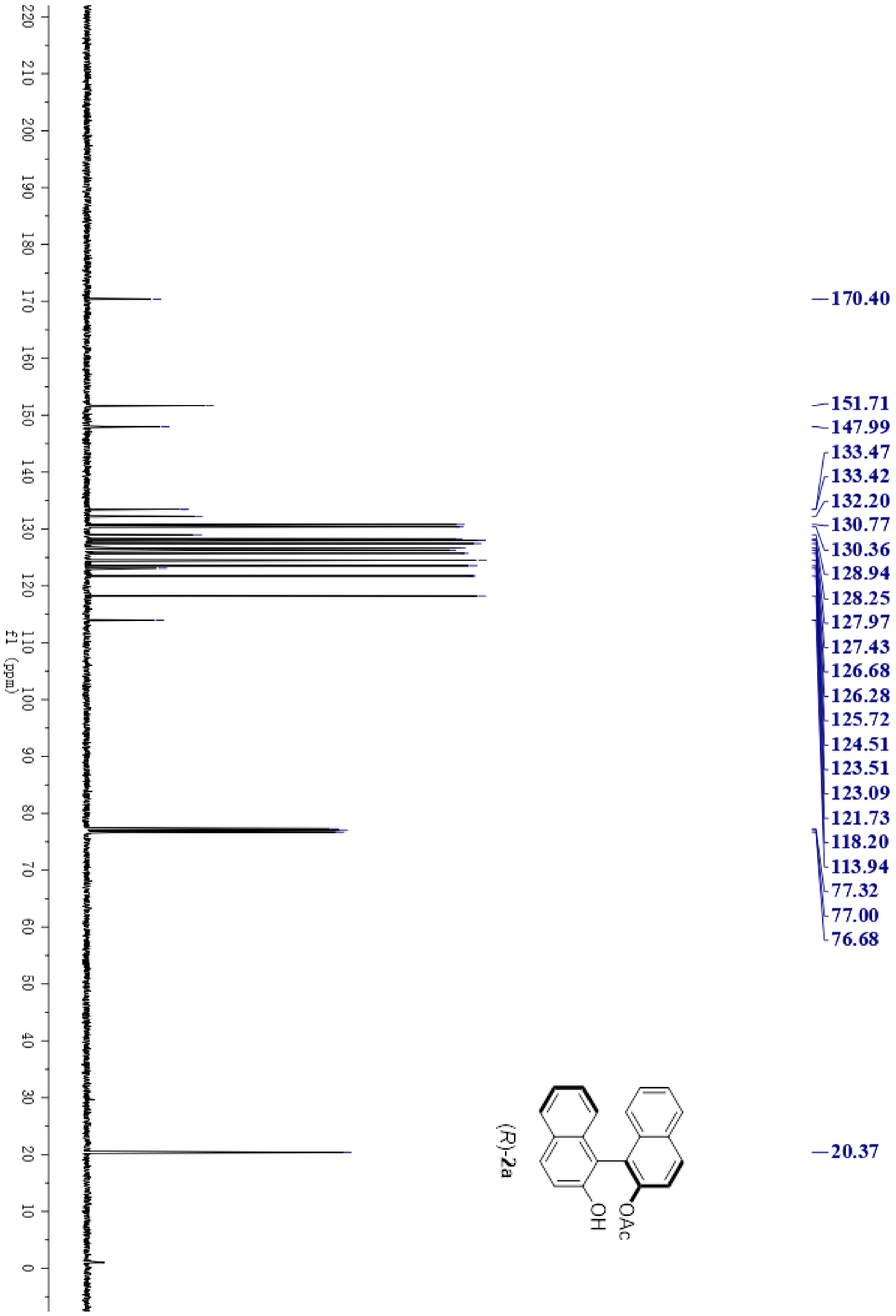
170.40
151.71
147.99
133.47
133.42
132.20
130.77
130.36
128.94
128.25
127.97
127.43
126.68
126.28
125.72
124.51
123.51
123.09
121.73
118.20
113.94
77.32
77.00
76.68
20.37
f1 (ppm)
OAc
OH
(R)-2a

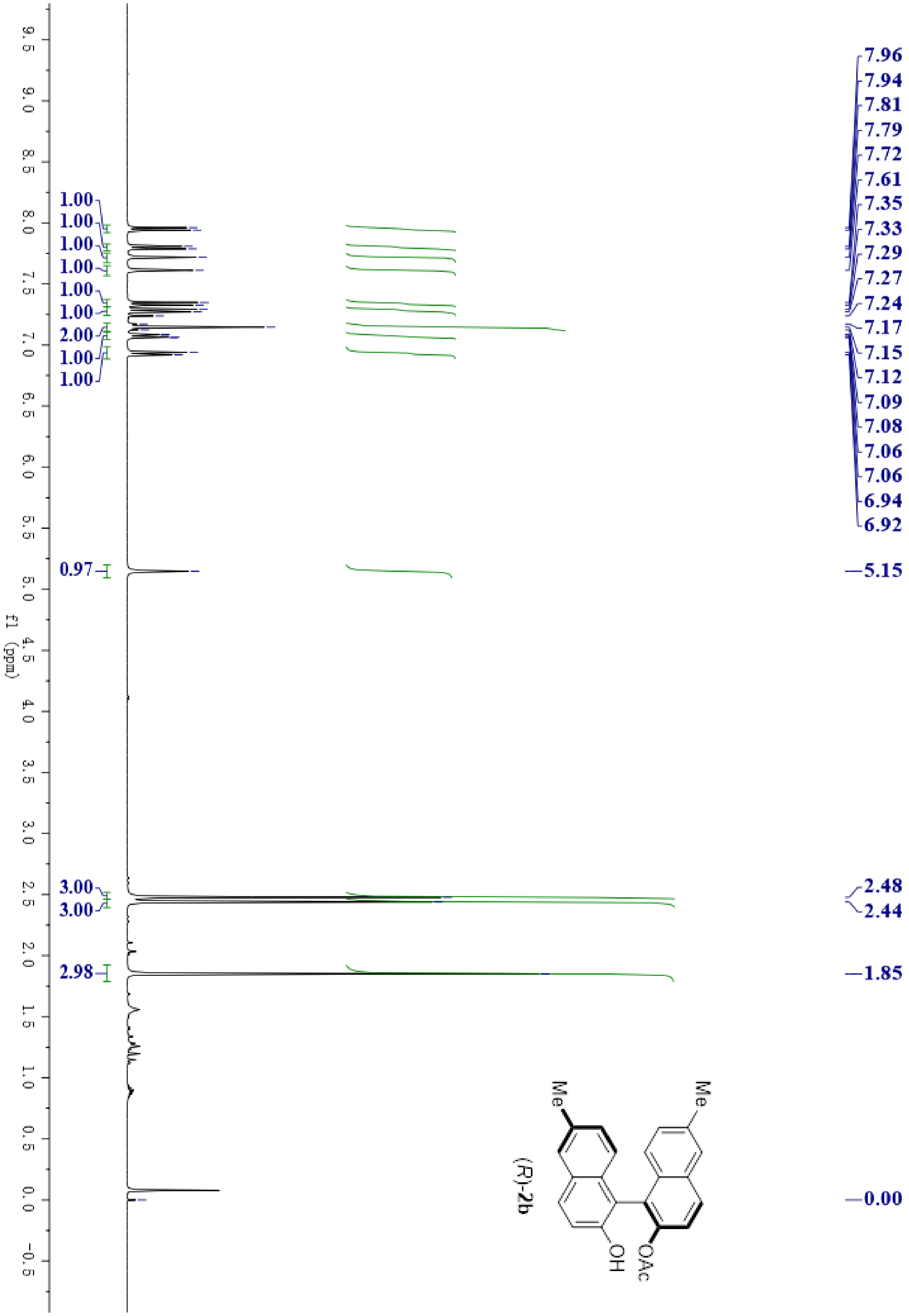
7.96
7.94
7.81
7.79
7.72
7.61
7.35
7.33
7.29
7.27
7.24
7.17
7.15
7.12
7.09
7.08
7.06
7.06
6.94
6.92
5.15
2.48
2.44
1.85
0.00
1.00
1.00
1.00
1.00
1.00
1.00
2.00
1.00
1.00
0.97
3.00
3.00
2.98
f1 (ppm)
Me
Me
OAc
OH
(R)-2b

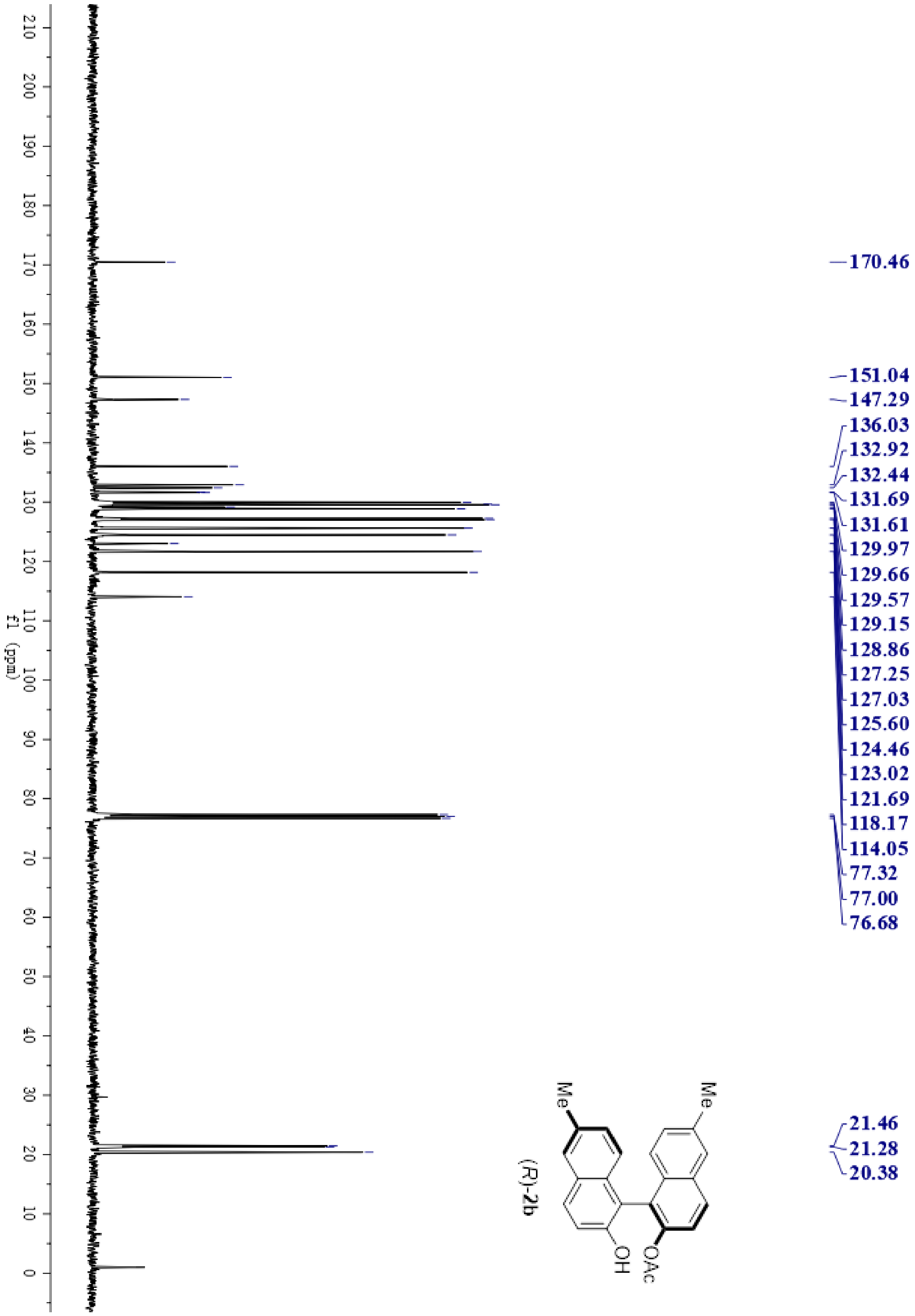
170.46
151.04
147.29
136.03
132.92
132.44
131.69
131.61
129.97
129.66
129.57
129.15
128.86
127.25
127.03
125.60
124.46
123.02
121.69
118.17
114.05
77.32
77.00
76.68
21.46
21.28
20.38
f1 (ppm)
Me
Me
OAc
OH
(R)-2b

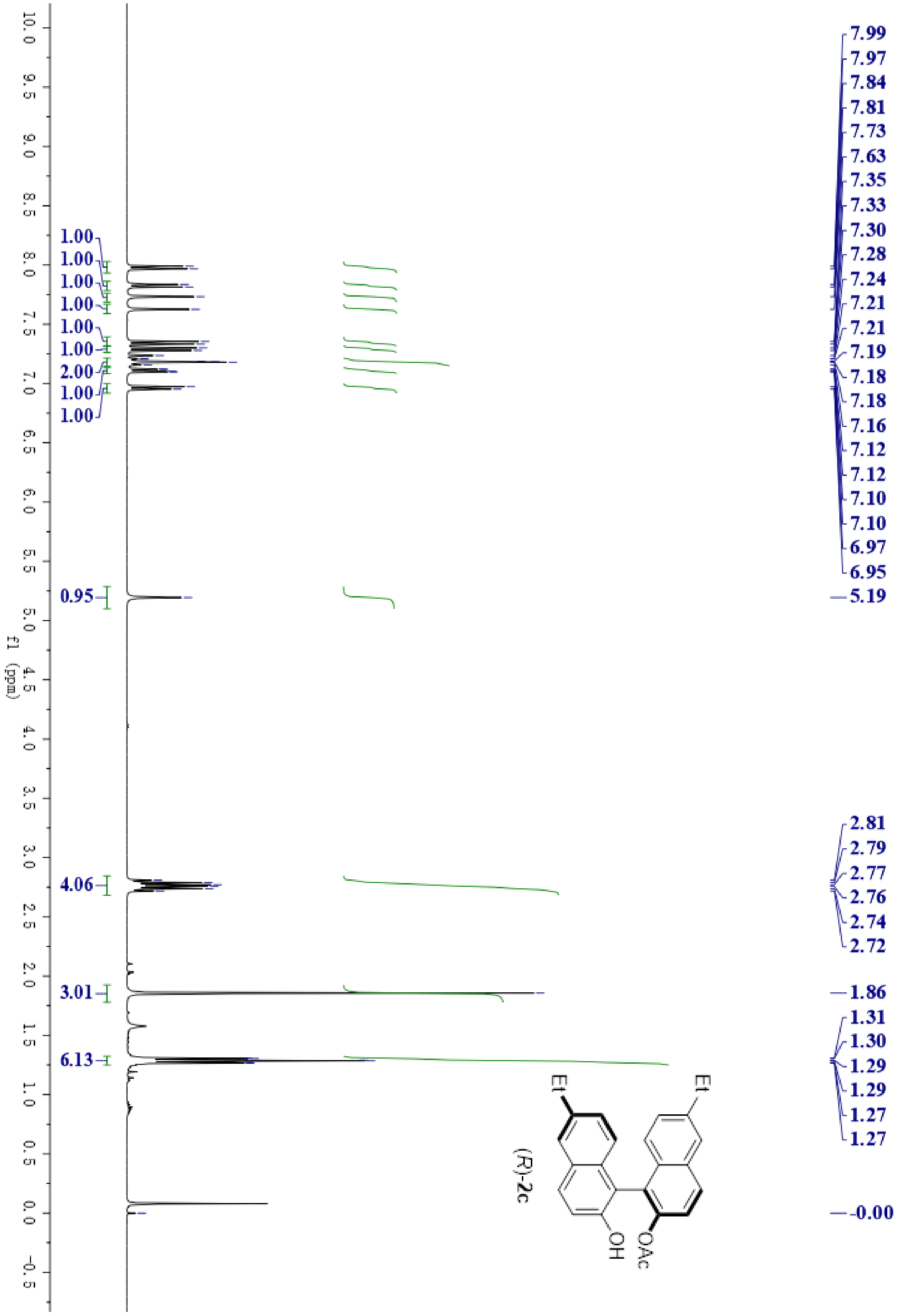
7.99
7.97
7.84
7.81
7.73
7.63
7.35
7.33
7.30
7.28
7.24
7.21
7.21
7.19
7.18
7.18
7.16
7.12
7.12
7.10
7.10
6.97
6.95
5.19
2.81
2.79
2.77
2.76
2.74
2.72
1.86
1.31
1.30
1.29
1.29
1.27
1.27
-0.00
1.00
1.00
1.00
1.00
1.00
1.00
2.00
1.00
1.00
0.95
4.06
3.01
6.13
f1 (ppm)
Et
Et
OAc
OH
(R)-2c

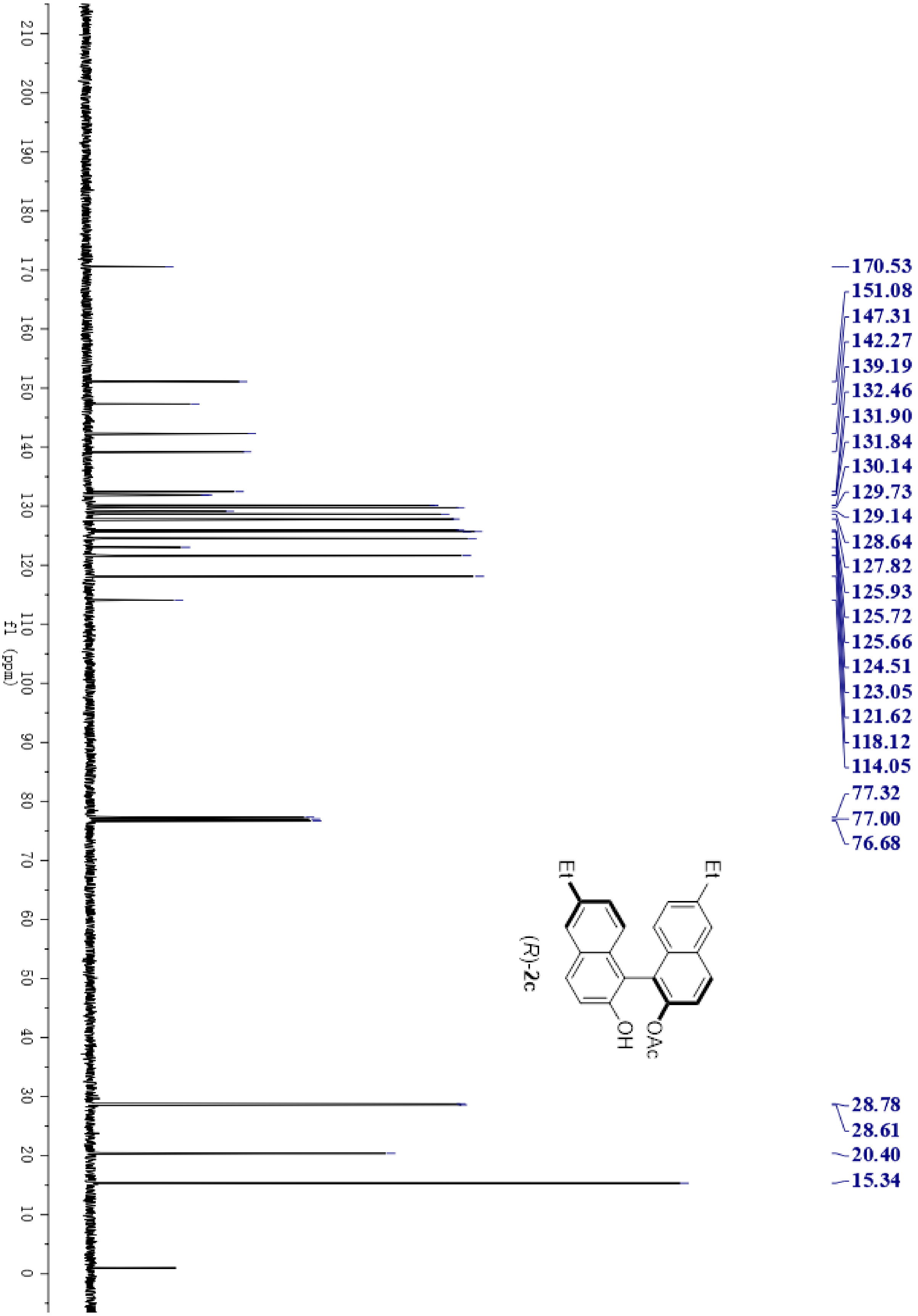
170.53
151.08
147.31
142.27
139.19
132.46
131.90
131.84
130.14
129.73
129.14
128.64
127.82
125.93
125.72
125.66
124.51
123.05
121.62
118.12
114.05
77.32
77.00
76.68
28.78
28.61
20.40
15.34
f1 (ppm)
Et
Et
OAc
OH
(R)-2c

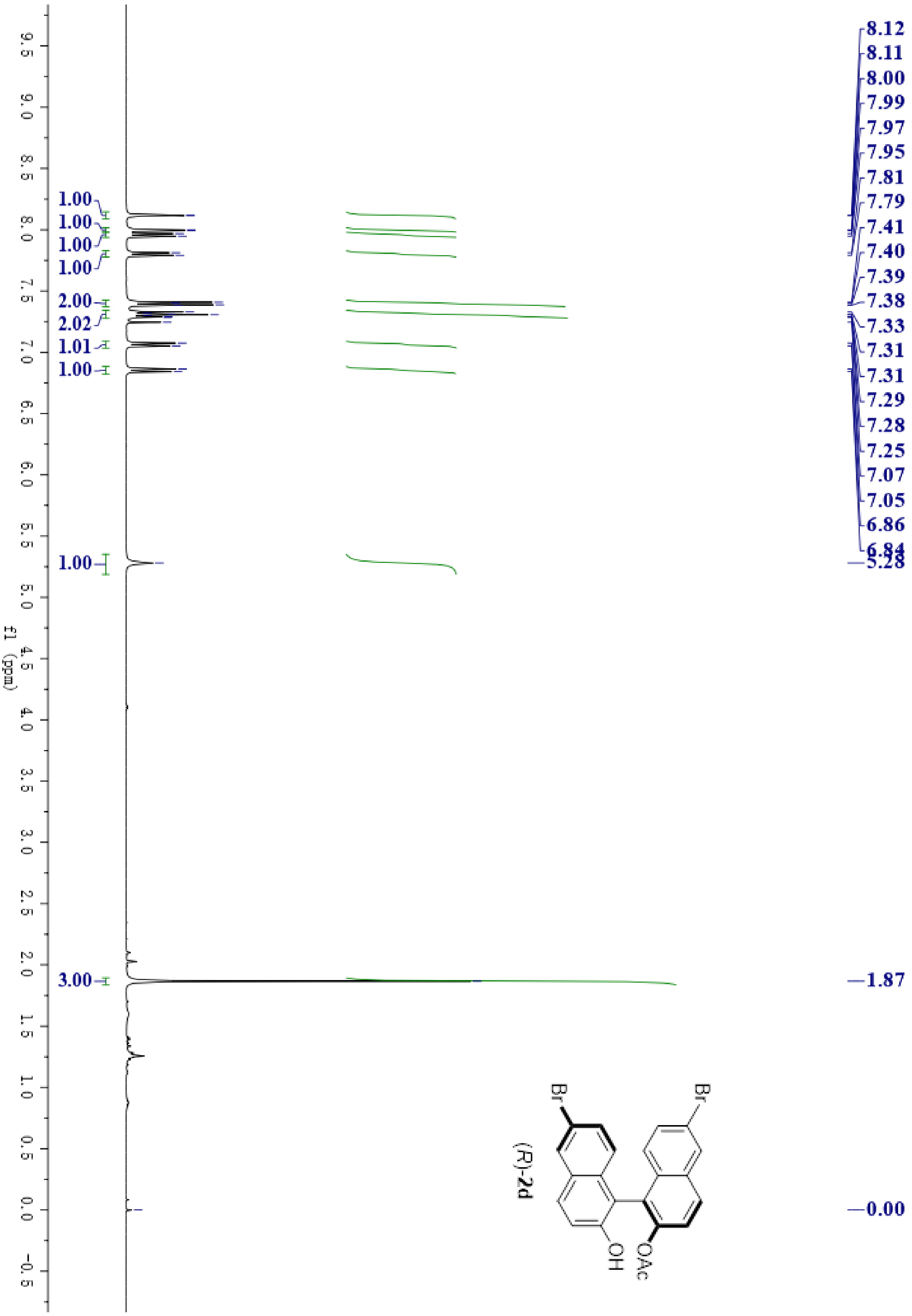

8.12
8.11
8.00
7.99
7.97
7.95
7.81
7.79
7.41
7.40
7.39
7.38
7.33
7.31
7.31
7.29
7.28
7.25
7.07
7.05
6.86
6.84
5.28
1.87
0.00
1.00
1.00
1.00
1.00
2.00
2.02
1.01
1.00
1.00
3.00
f1 (ppm)
Br
Br
OAc
OH
(R)-2d

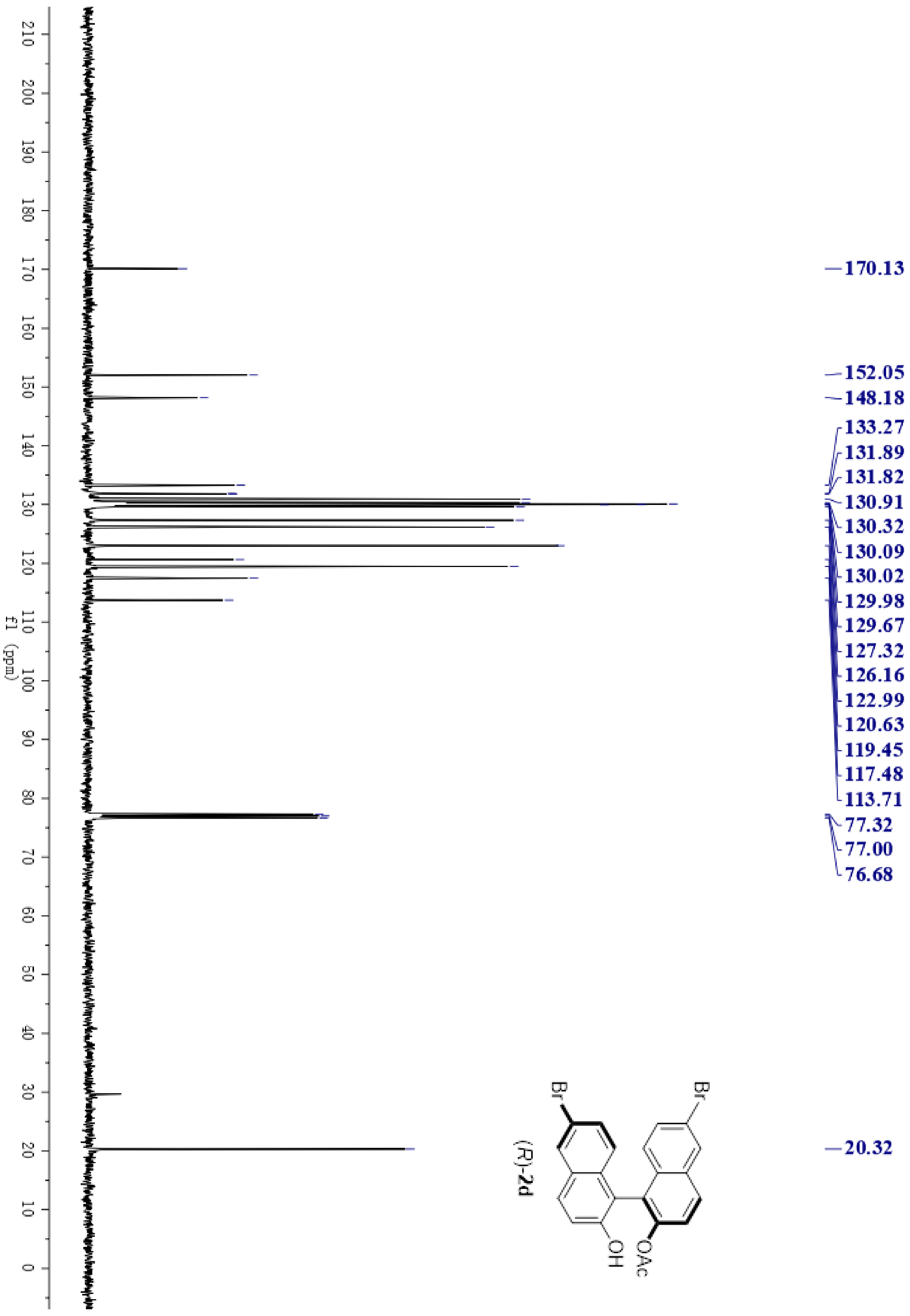
170.13
152.05
148.18
133.27
131.89
131.82
130.91
130.32
130.09
130.02
129.98
129.67
127.32
126.16
122.99
120.63
119.45
117.48
113.71
77.32
77.00
76.68
20.32
f1 (ppm)
Br
Br
OAc
OH
(R)-2d

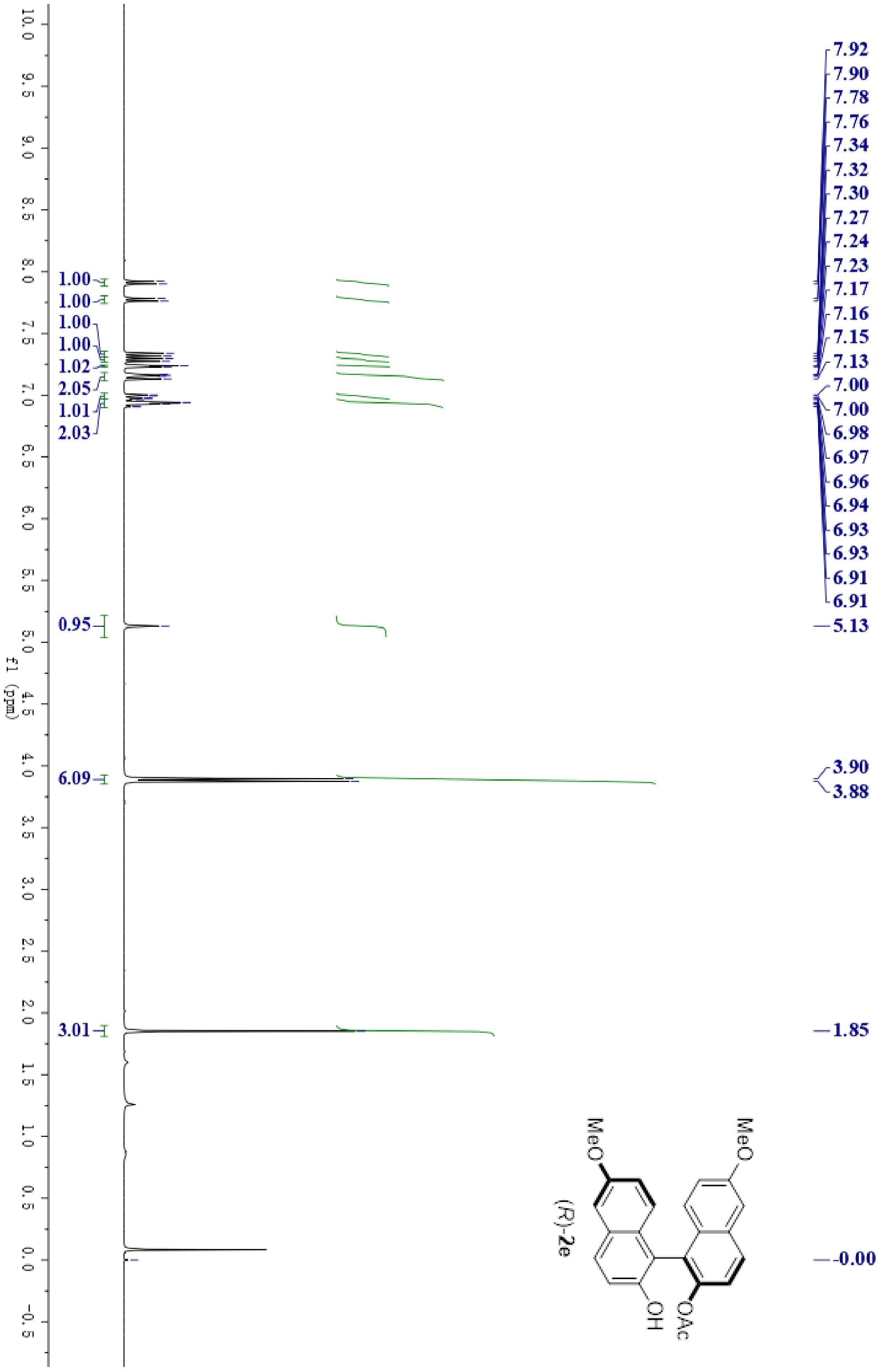
7.92
7.90
7.78
7.76
7.34
7.32
7.30
7.27
7.24
7.23
7.17
7.16
7.15
7.13
7.00
7.00
6.98
6.97
6.96
6.94
6.93
6.93
6.91
6.91
5.13
3.90
3.88
1.85
-0.00
1.00
1.00
1.00
1.00
1.02
2.05
1.01
2.03
0.95
6.09
3.01
f1 (ppm)
MeO
MeO
OAc
OH
(R)-2e

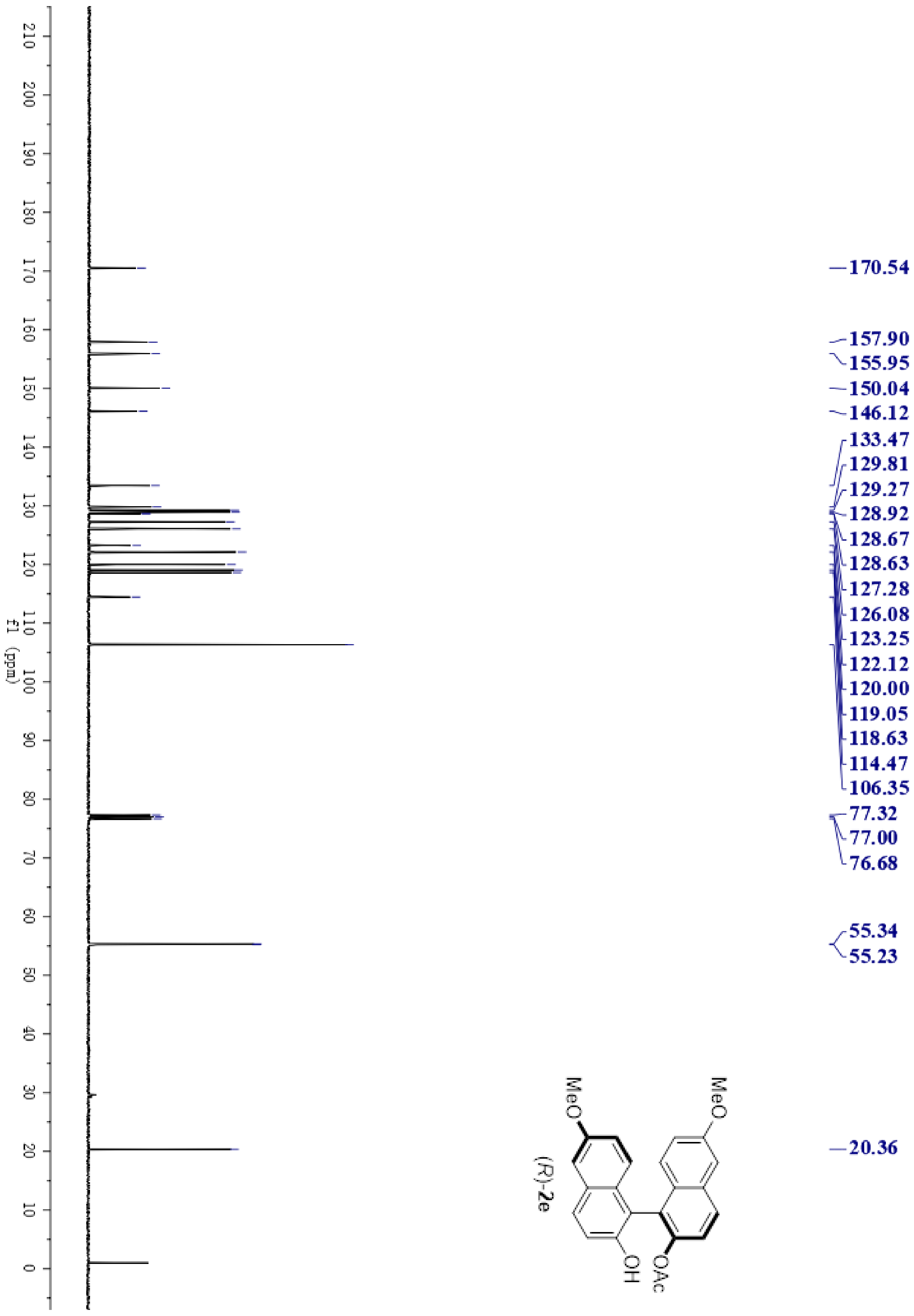
170.54
157.90
155.95
150.04
146.12
133.47
129.81
129.27
128.92
128.67
128.63
127.28
126.08
123.25
122.12
120.00
119.05
118.63
114.47
106.35
77.32
77.00
76.68
55.34
55.23
20.36
f1 (ppm)
MeO
MeO
OAc
OH
(R)-2e

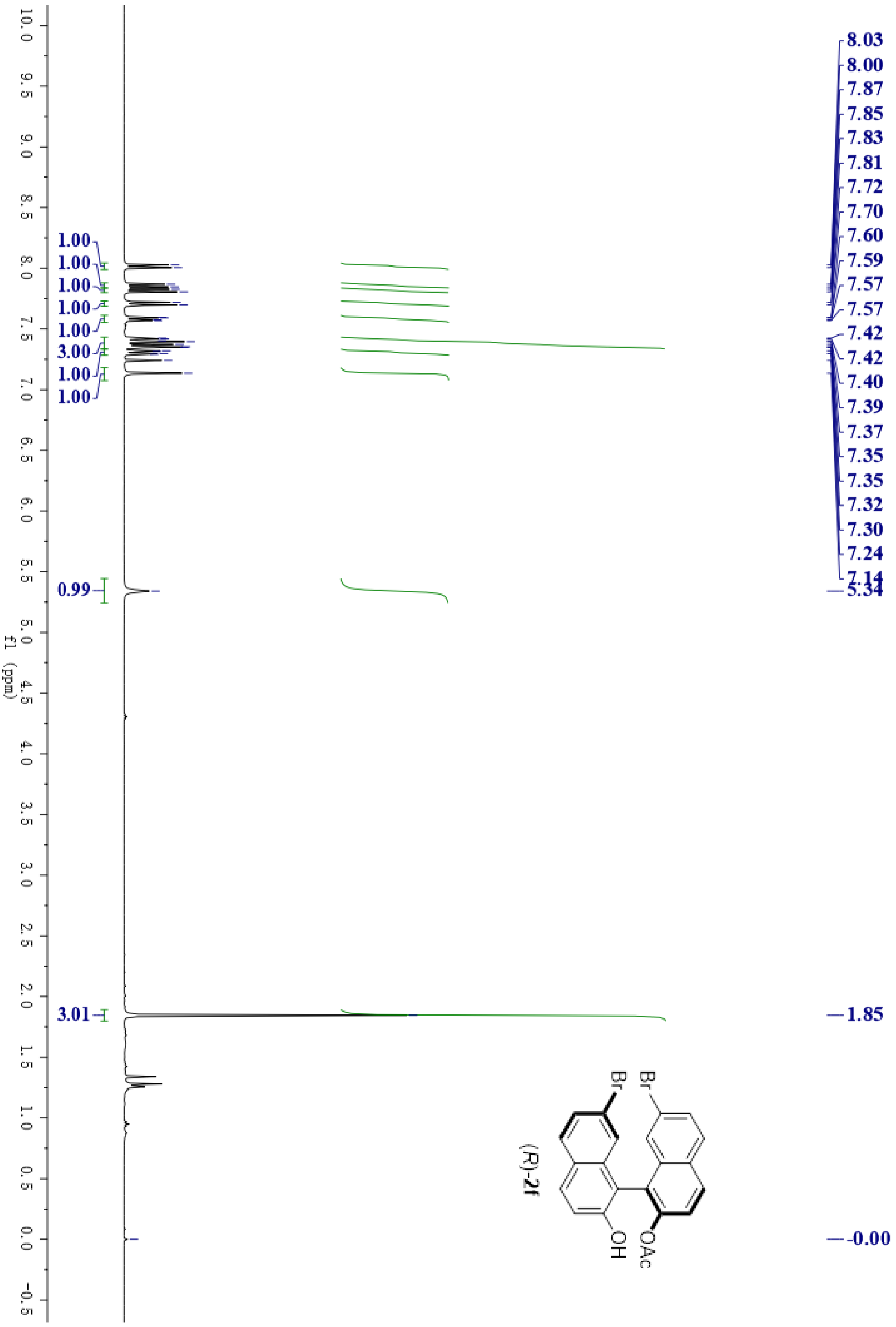

8.03
8.00
7.87
7.85
7.83
7.81
7.72
7.70
7.60
7.59
7.57
7.57
7.42
7.42
7.40
7.39
7.37
7.35
7.35
7.32
7.30
7.24
7.14
5.34
1.85
-0.00
1.00
1.00
1.00
1.00
1.00
3.00
1.00
1.00
0.99
3.01
f1 (ppm)
Br
Br
OAc
OH
(R)-2f

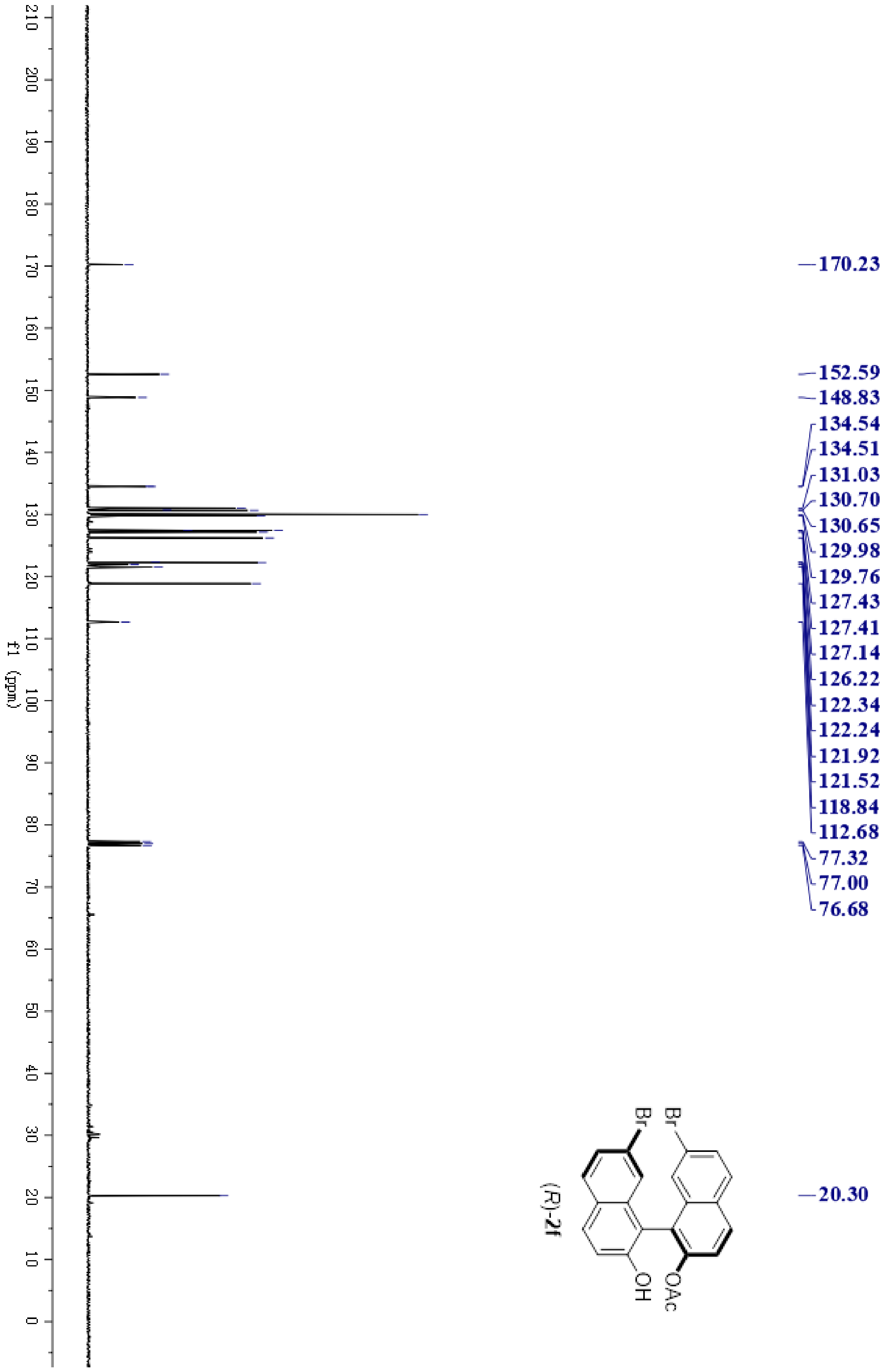

170.23
152.59
148.83
134.54
134.51
131.03
130.70
130.65
129.98
129.76
127.43
127.41
127.14
126.22
122.34
122.24
121.92
121.52
118.84
112.68
77.32
77.00
76.68
20.30
f1 (ppm)
Br
Br
OAc
OH
(R)-2f

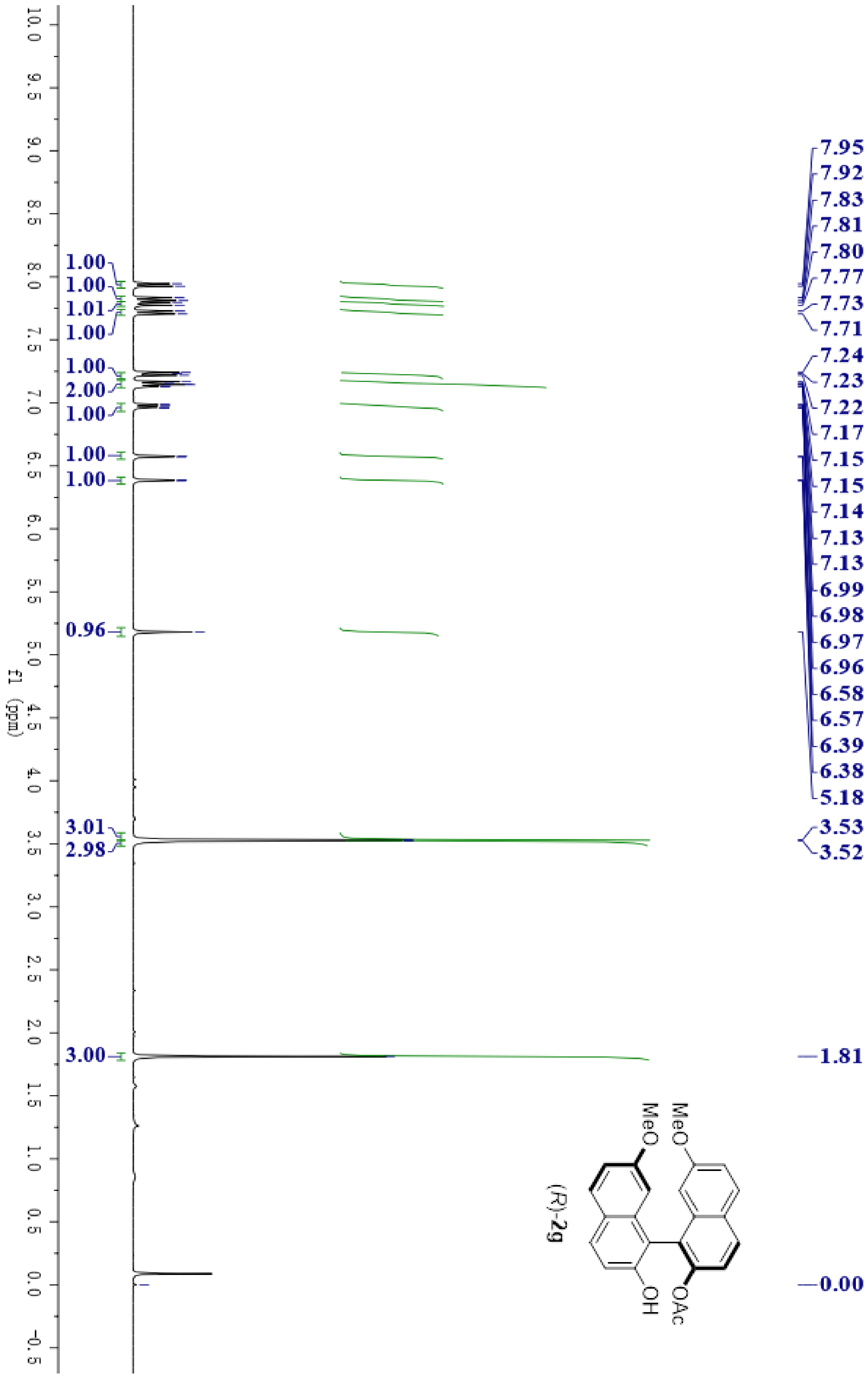
7.95
7.92
7.83
7.81
7.80
7.77
7.73
7.71
7.24
7.23
7.22
7.17
7.15
7.15
7.14
7.13
7.13
6.99
6.98
6.97
6.96
6.58
6.57
6.39
6.38
5.18
3.53
3.52
1.81
0.00
1.00
1.00
1.01
1.00
1.00
2.00
1.00
1.00
1.00
0.96
3.01
2.98
3.00
f1 (ppm)
MeO
MeO
OAc
OH
(R)-2g

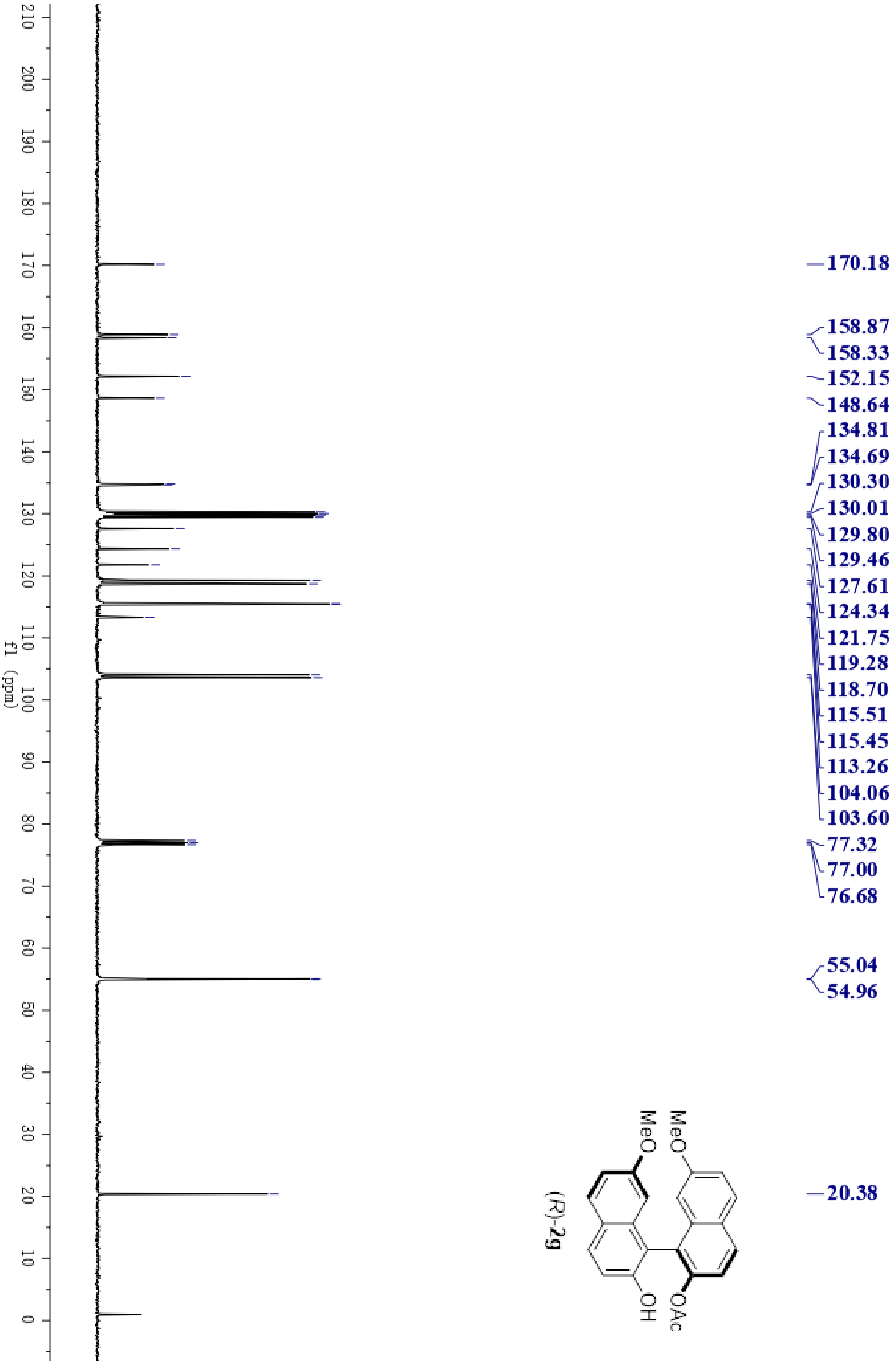
170.18
158.87
158.33
152.15
148.64
134.81
134.69
130.30
130.01
129.80
129.46
127.61
124.34
121.75
119.28
118.70
115.51
115.45
113.26
104.06
103.60
77.32
77.00
76.68
55.04
54.96
20.38
f1 (ppm)
MeO
MeO
OAc
OH
(R)-2g

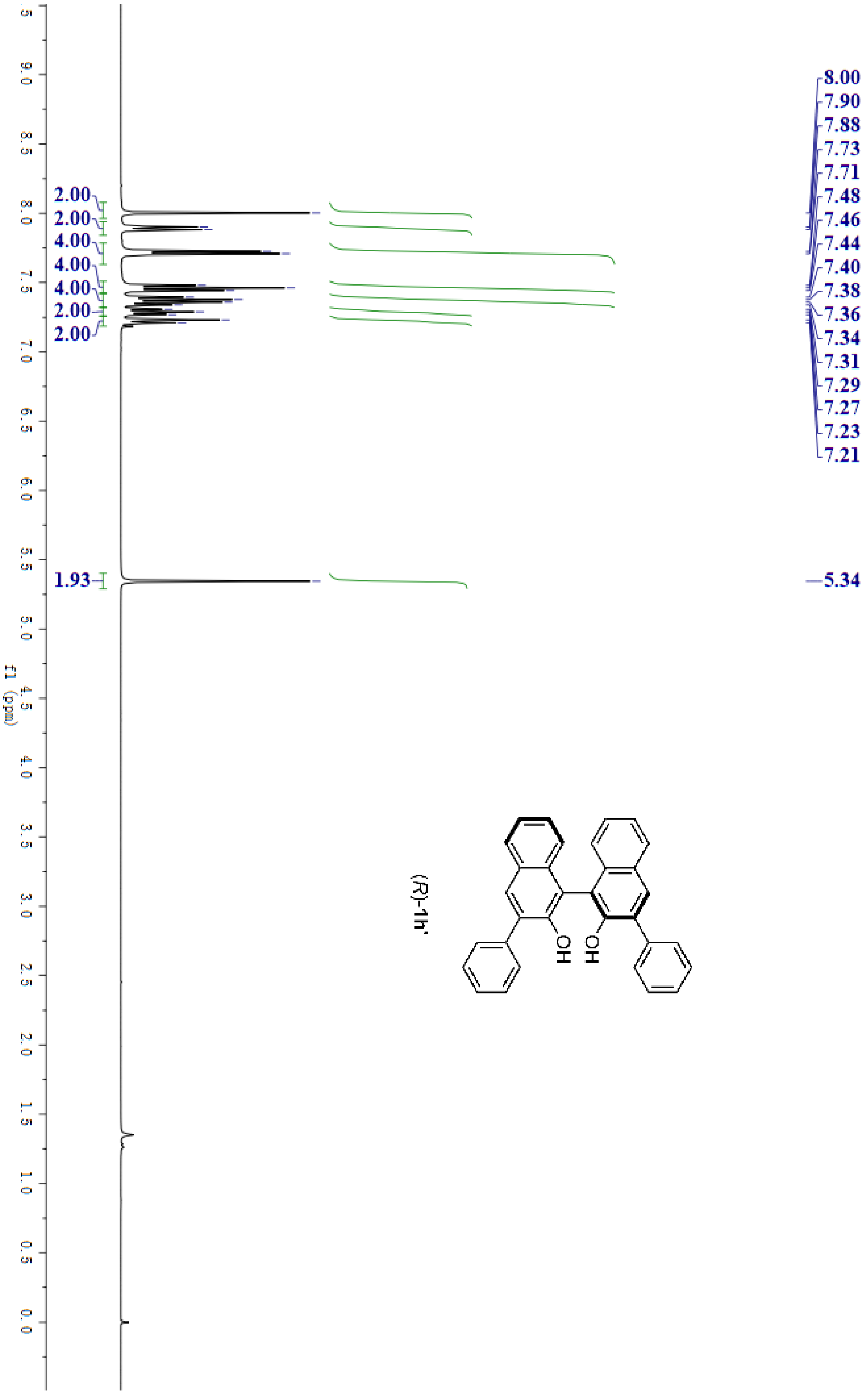
8.00
7.90
7.88
7.73
7.71
7.48
7.46
7.44
7.40
7.38
7.36
7.34
7.31
7.29
7.27
7.23
7.21
5.34
2.00
2.00
4.00
4.00
4.00
2.00
2.00
1.93
OH
OH
(R)-1h'
f1 (ppm)

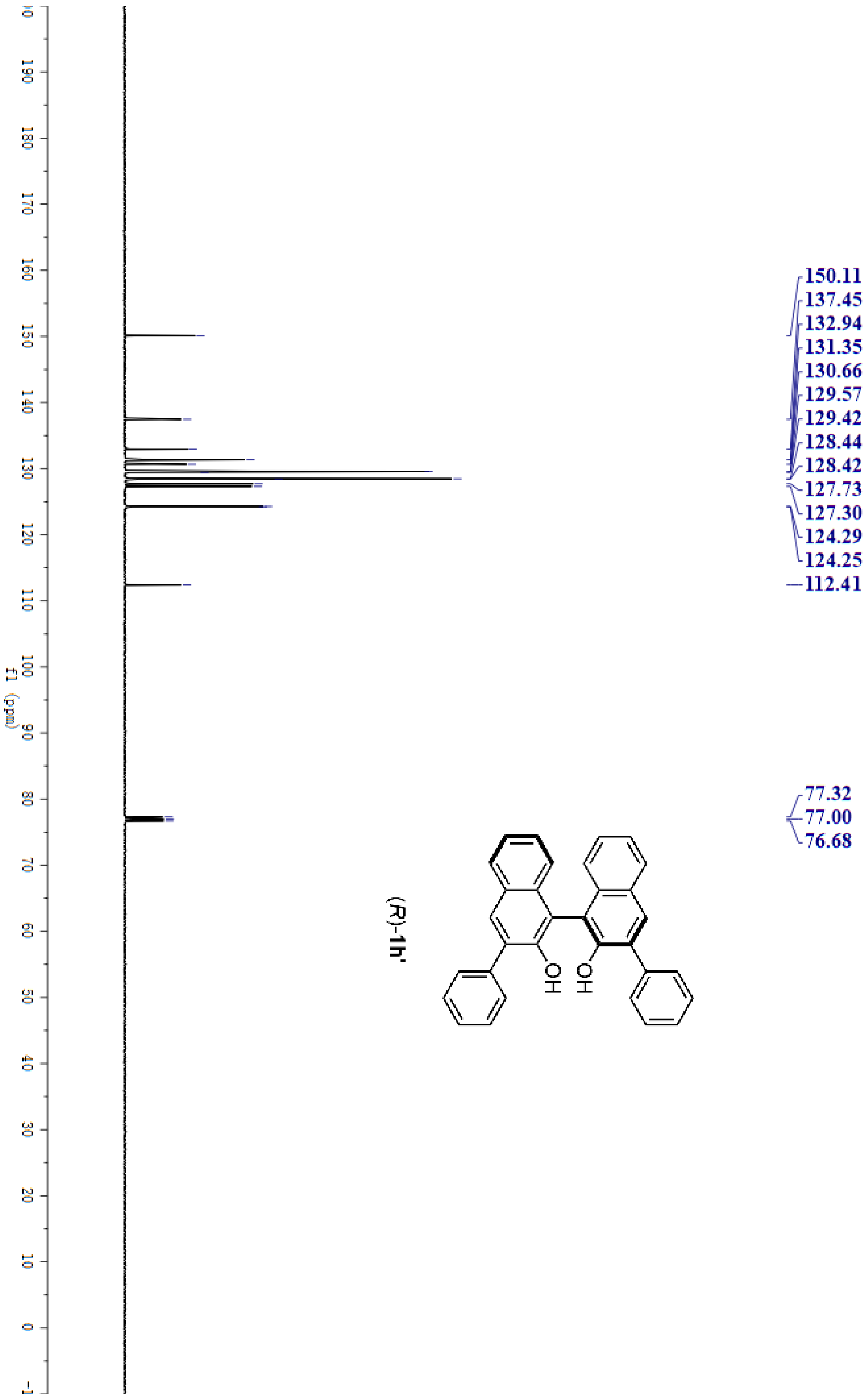
150.11
137.45
132.94
131.35
130.66
129.57
129.42
128.44
128.42
127.73
127.30
124.29
124.25
112.41
77.32
77.00
76.68
f1 (ppm)
OH
OH
(R)-1h'

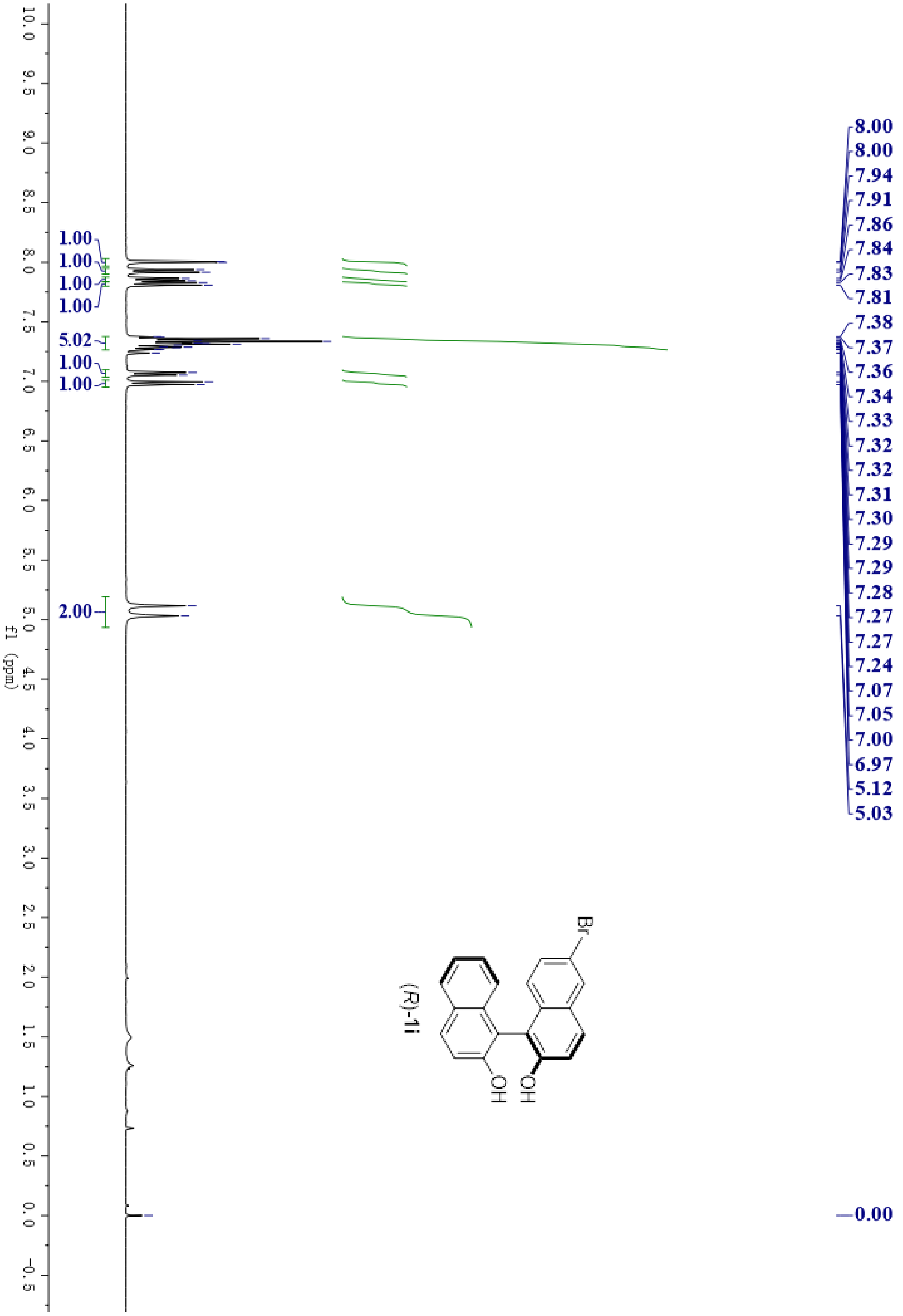
8.00
8.00
7.94
7.91
7.86
7.84
7.83
7.81
7.38
7.37
7.36
7.34
7.33
7.32
7.32
7.31
7.30
7.29
7.29
7.28
7.27
7.27
7.24
7.07
7.05
7.00
6.97
5.12
5.03
0.00
1.00
1.00
1.00
1.00
5.02
1.00
1.00
2.00
f1 (ppm)
Br
OH
OH
(R)-1i

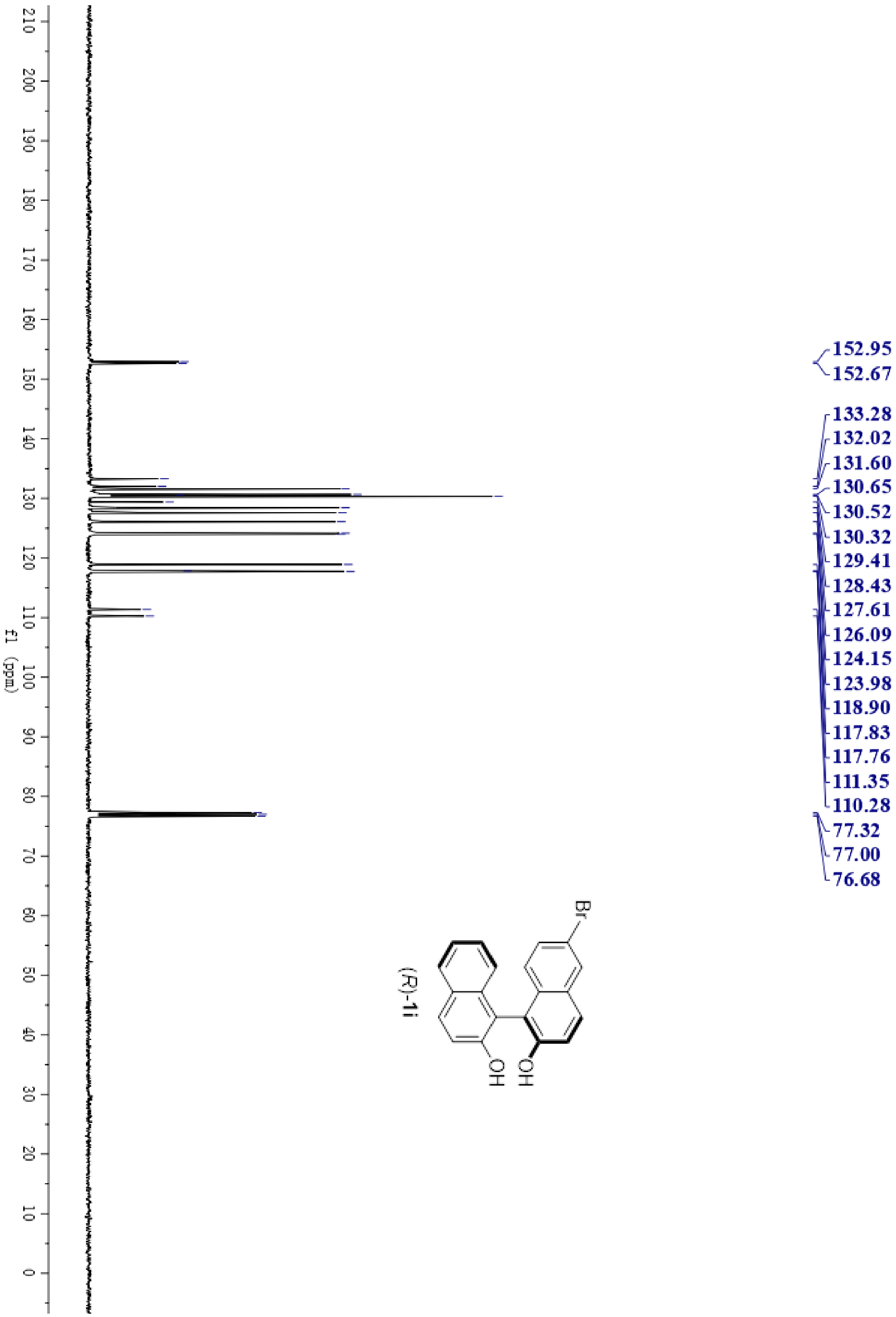
152.95
152.67
133.28
132.02
131.60
130.65
130.52
130.32
129.41
128.43
127.61
126.09
124.15
123.98
118.90
117.83
117.76
111.35
110.28
77.32
77.00
76.68
f1 (ppm)
Br
OH
OH
(R)-1i

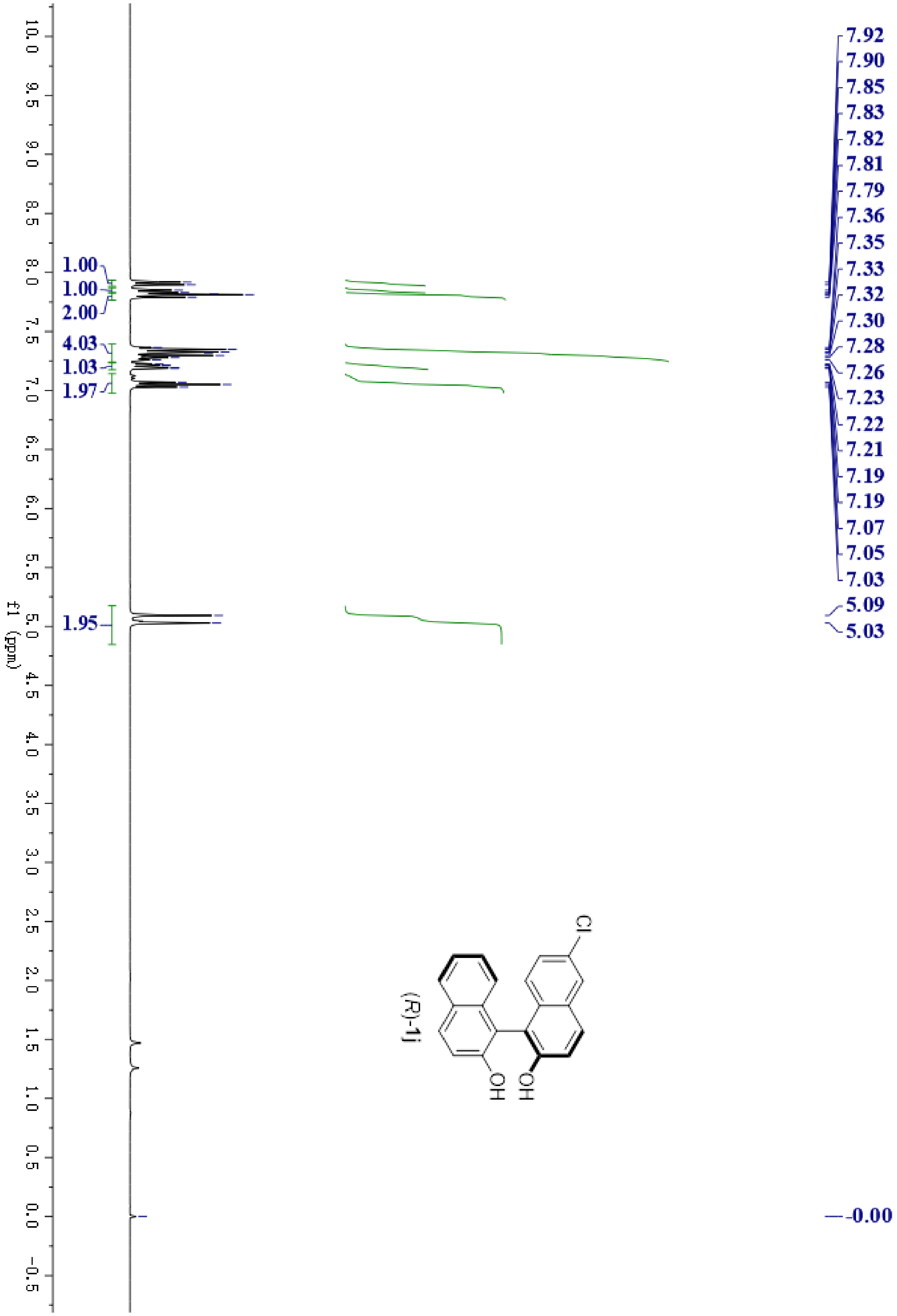
7.92
7.90
7.85
7.83
7.82
7.81
7.79
7.36
7.35
7.33
7.32
7.30
7.28
7.26
7.23
7.22
7.21
7.19
7.19
7.07
7.05
7.03
5.09
5.03
-0.00
1.00
1.00
2.00
4.03
1.03
1.97
1.95
10.0
9.5
9.0
8.5
8.0
7.5
7.0
6.5
6.0
5.5
5.0
4.5
4.0
3.5
3.0
2.5
2.0
1.5
1.0
0.5
0.0
-0.5
f1 (ppm)
Cl
OH
OH
(R)-1j

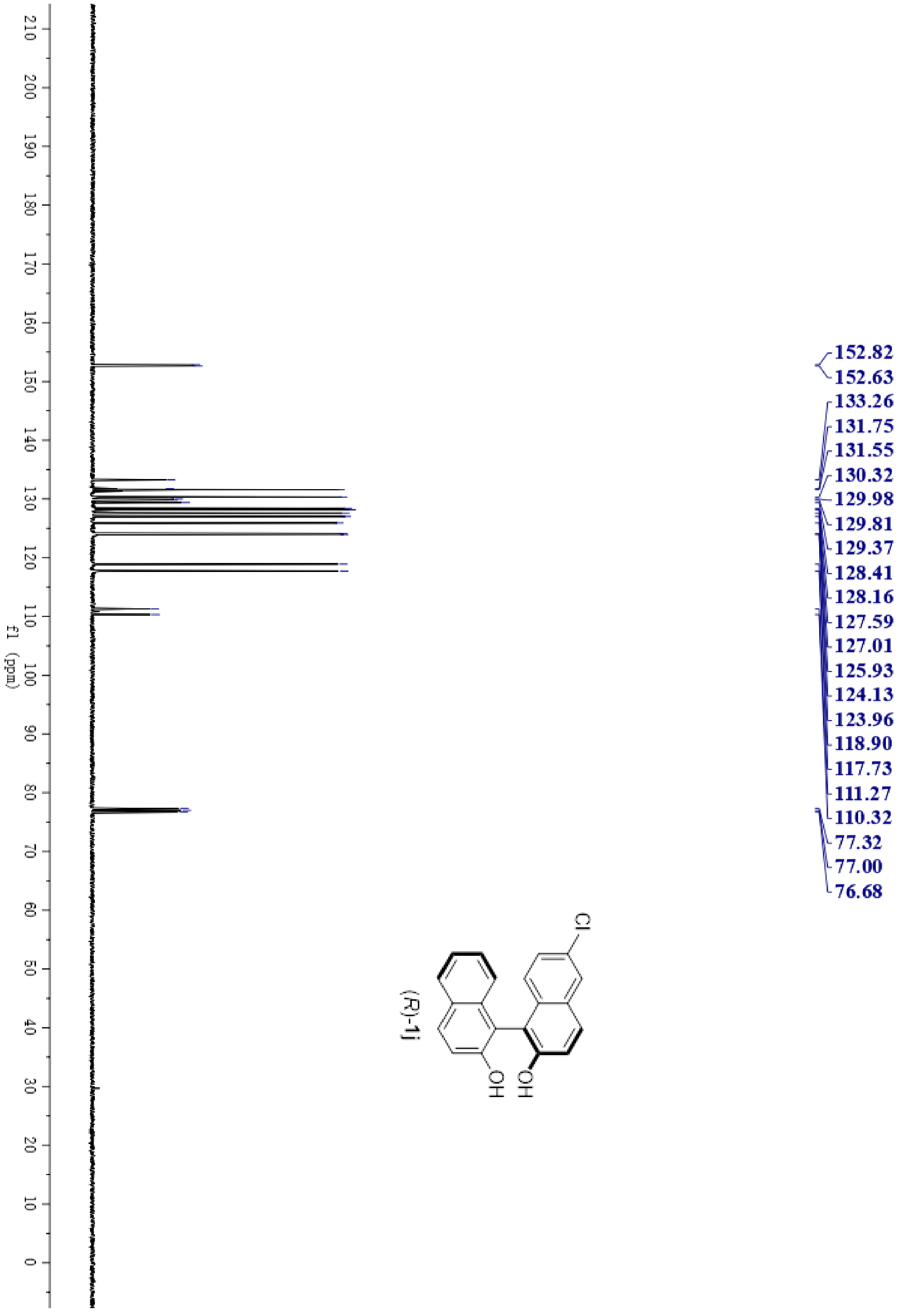
152.82
152.63
133.26
131.75
131.55
130.32
129.98
129.81
129.37
128.41
128.16
127.59
127.01
125.93
124.13
123.96
118.90
117.73
111.27
110.32
77.32
77.00
76.68
f1 (ppm)
Cl
OH
OH

(*R*)-1j

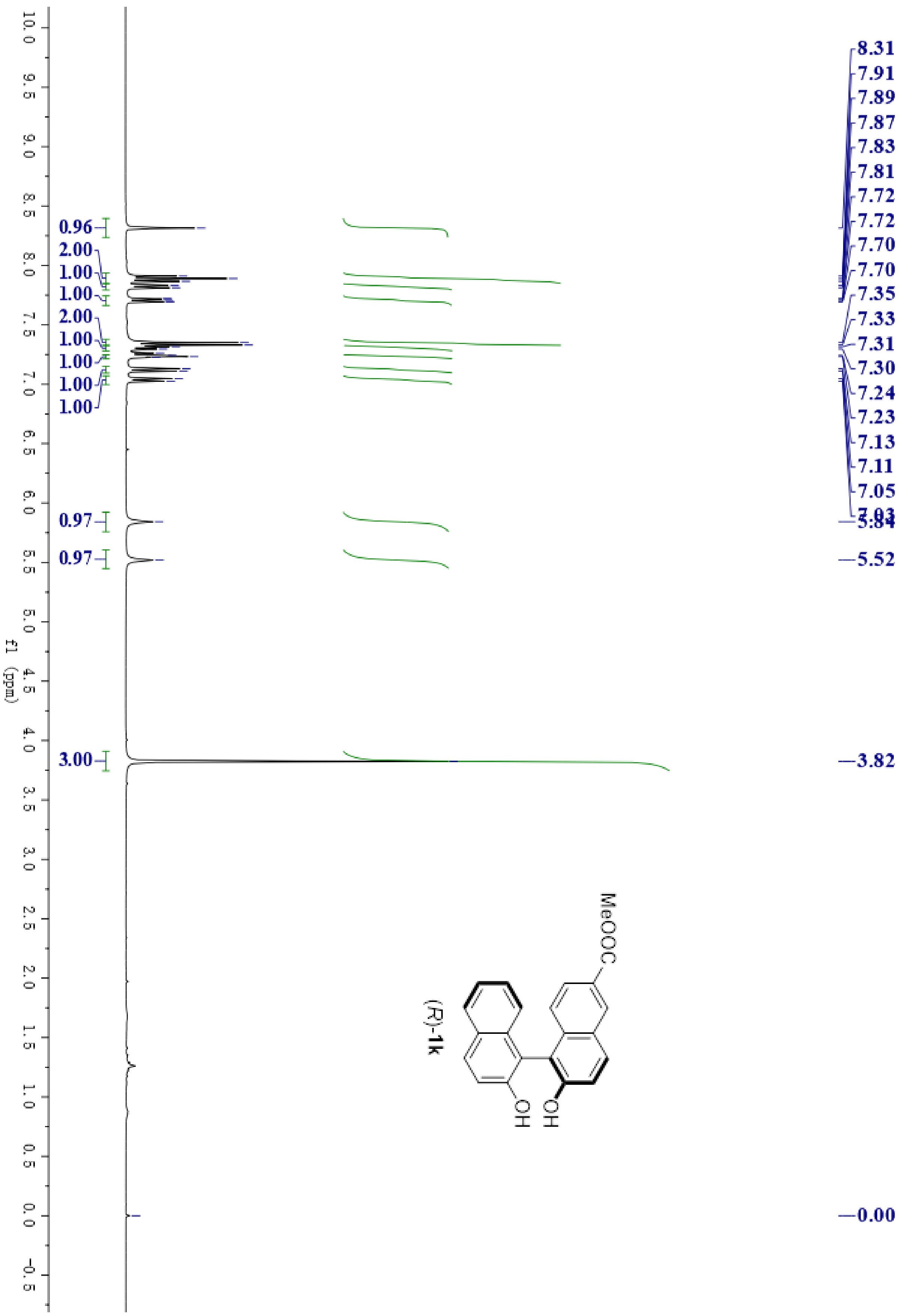
8.31
7.91
7.89
7.87
7.83
7.81
7.72
7.72
7.70
7.70
7.35
7.33
7.31
7.30
7.24
7.23
7.13
7.11
7.05
7.03
5.84
5.52
3.82
0.00
0.96
2.00
1.00
1.00
2.00
1.00
1.00
1.00
1.00
0.97
0.97
3.00
f1 (ppm)
MeOOC
OH
OH
(R)-1k

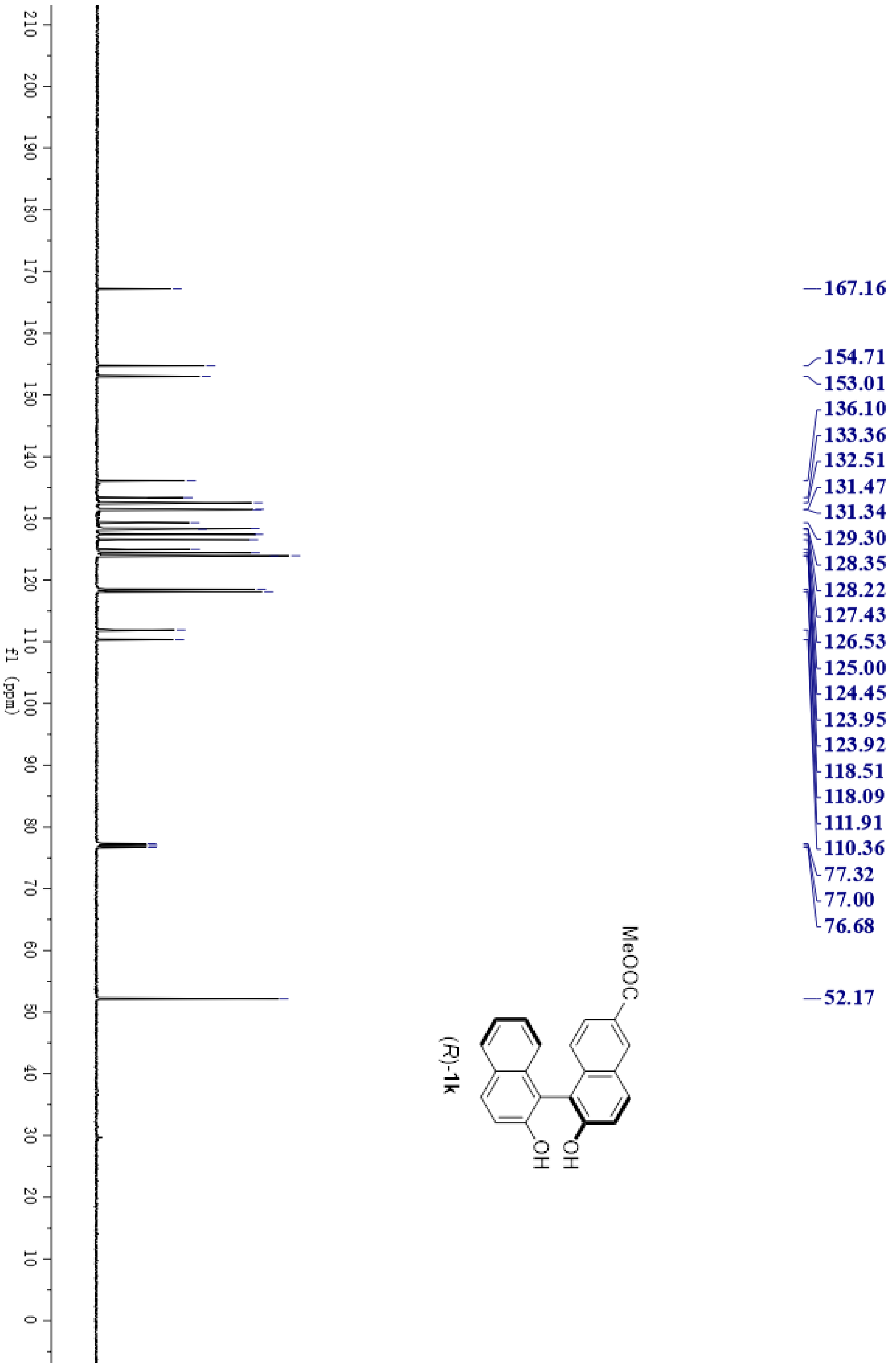
167.16
154.71
153.01
136.10
133.36
132.51
131.47
131.34
129.30
128.35
128.22
127.43
126.53
125.00
124.45
123.95
123.92
118.51
118.09
111.91
110.36
77.32
77.00
76.68
52.17
MeOOC
OH
OH
(R)-1k
f1 (ppm)
210
200
190
180
170
160
150
140
130
120
110
100
90
80
70
60
50
40
30
20
10
0

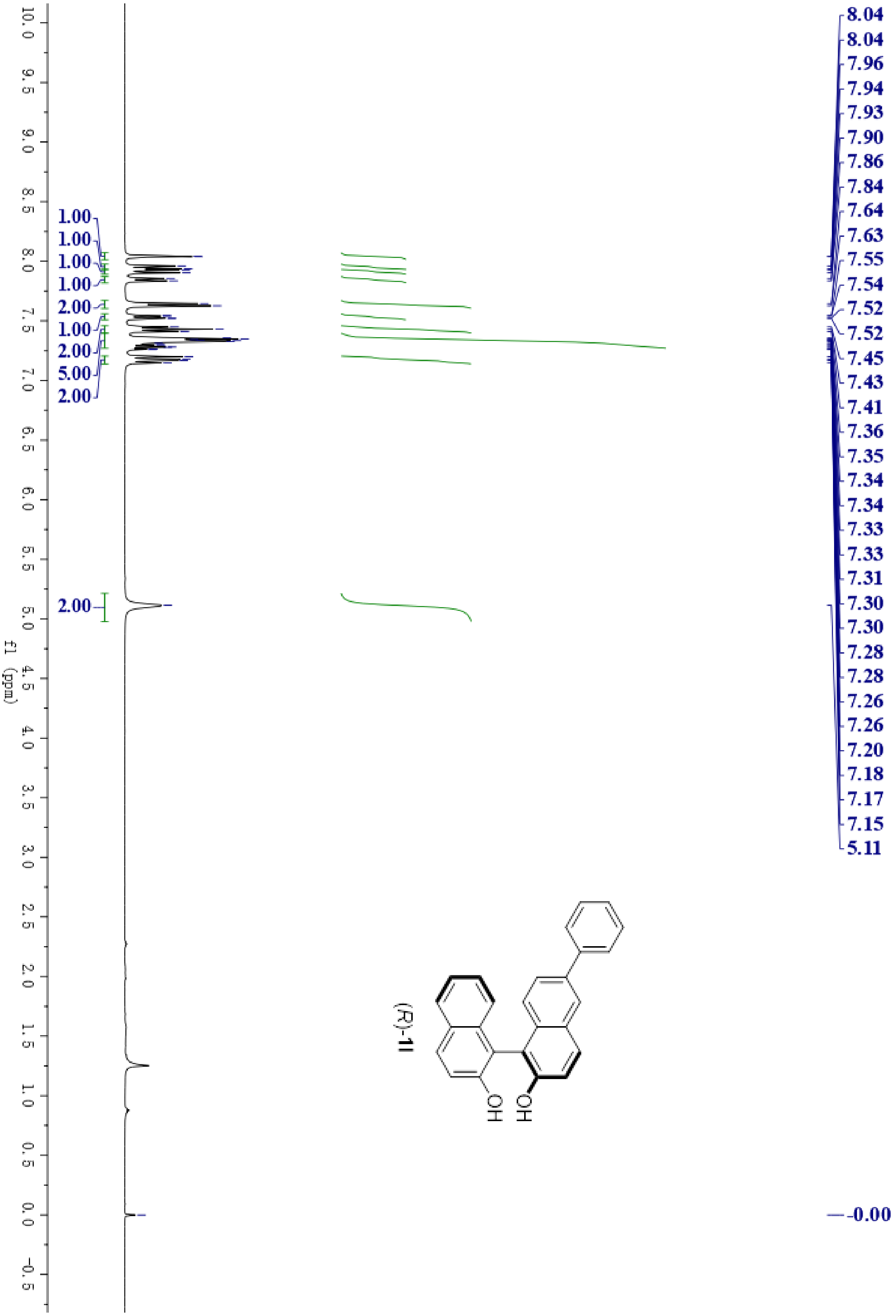
8.04
8.04
7.96
7.94
7.93
7.90
7.86
7.84
7.64
7.63
7.55
7.54
7.52
7.52
7.45
7.43
7.41
7.36
7.35
7.34
7.34
7.33
7.33
7.31
7.30
7.30
7.28
7.28
7.26
7.26
7.20
7.18
7.17
7.15
5.11
-0.00
1.00
1.00
1.00
1.00
2.00
1.00
2.00
5.00
2.00
2.00
f1 (ppm)
(R)-1l
OH
OH

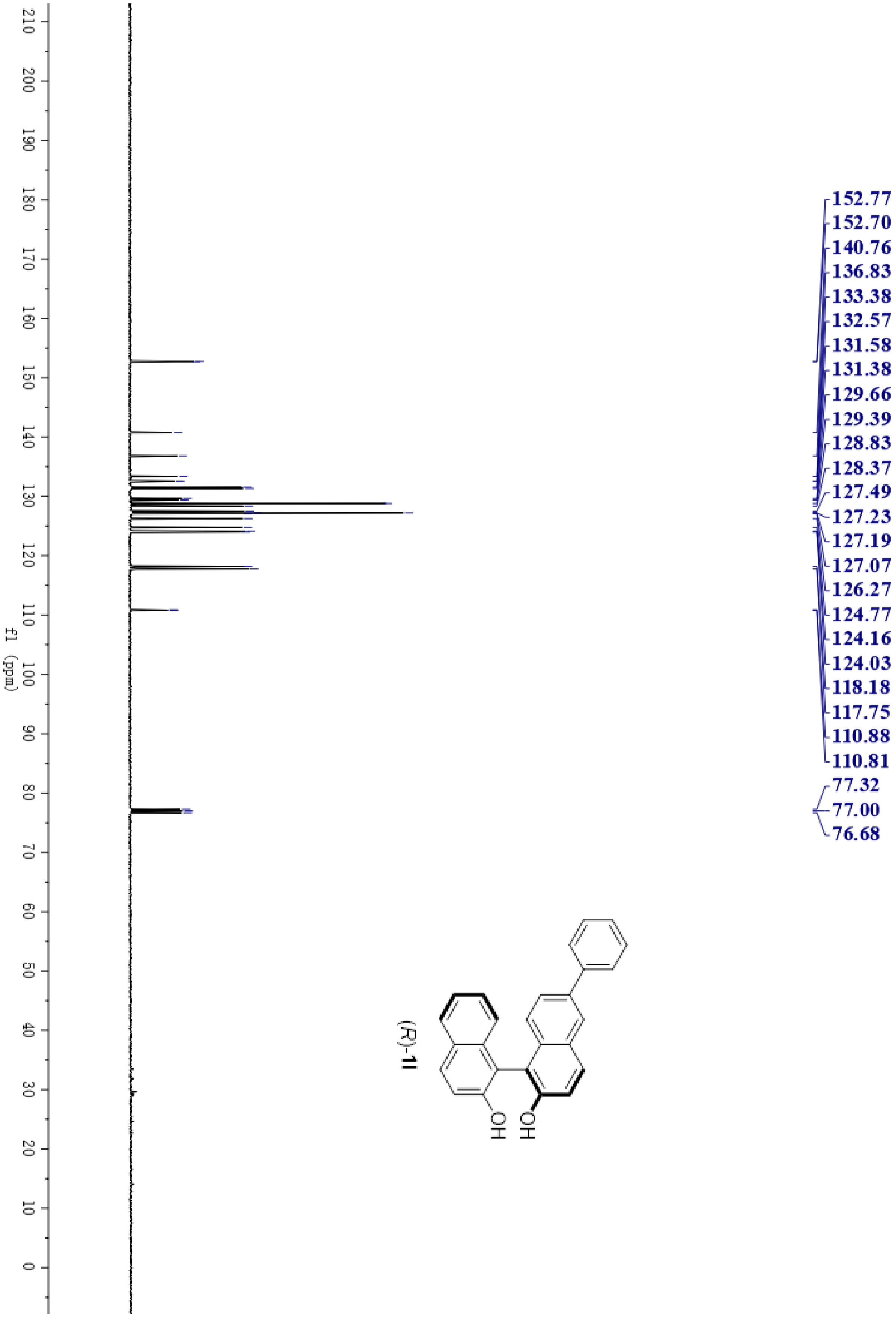
152.77
152.70
140.76
136.83
133.38
132.57
131.58
131.38
129.66
129.39
128.83
128.37
127.49
127.23
127.19
127.07
126.27
124.77
124.16
124.03
118.18
117.75
110.88
110.81
77.32
77.00
76.68
f1 (ppm)
(R)-1l
OH
OH

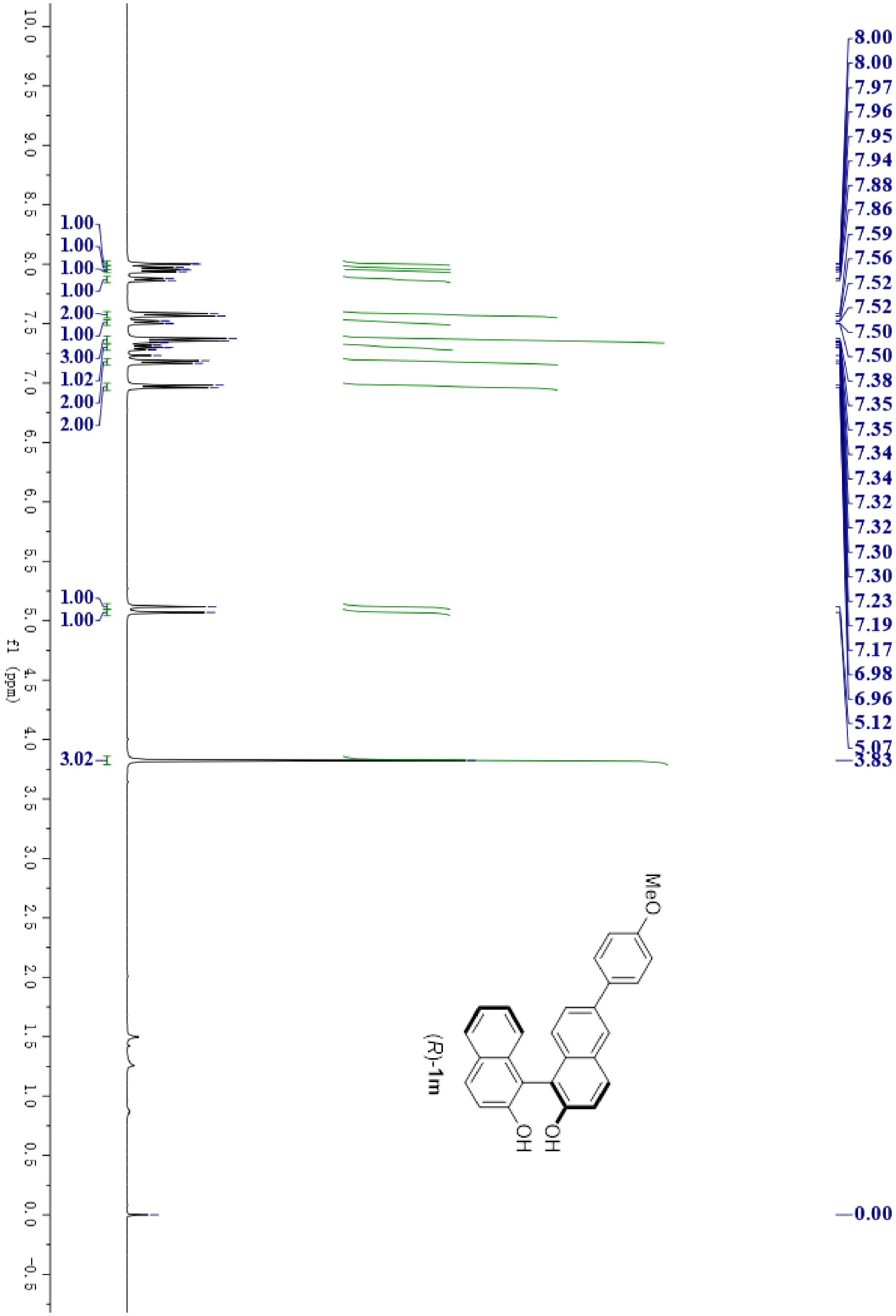
8.00
8.00
7.97
7.96
7.95
7.94
7.88
7.86
7.59
7.56
7.52
7.52
7.50
7.50
7.38
7.35
7.35
7.34
7.34
7.32
7.32
7.30
7.30
7.23
7.19
7.17
6.98
6.96
5.12
5.07
3.83
0.00
1.00
1.00
1.00
1.00
2.00
1.00
3.00
1.02
2.00
2.00
1.00
1.00
3.02
f1 (ppm)
10.0 9.5 9.0 8.5 8.0 7.5 7.0 6.5 6.0 5.5 5.0 4.5 4.0 3.5 3.0 2.5 2.0 1.5 1.0 0.5 0.0 -0.5
MeO
OH
OH
(R)-1m

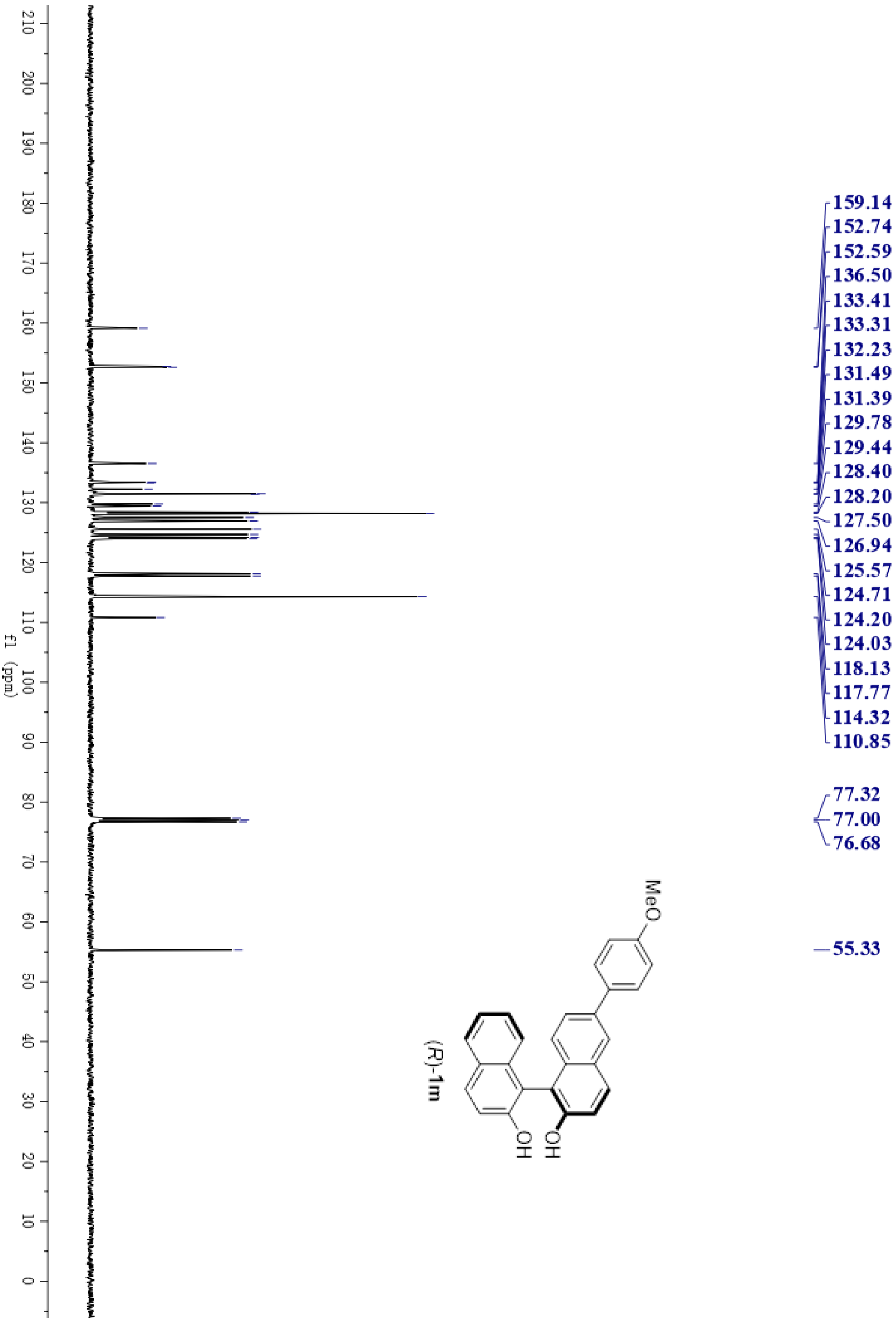
159.14
152.74
152.59
136.50
133.41
133.31
132.23
131.49
131.39
129.78
129.44
128.40
128.20
127.50
126.94
125.57
124.71
124.20
124.03
118.13
117.77
114.32
110.85
77.32
77.00
76.68
55.33
MeO
OH
OH
(R)-1m
f1 (ppm)

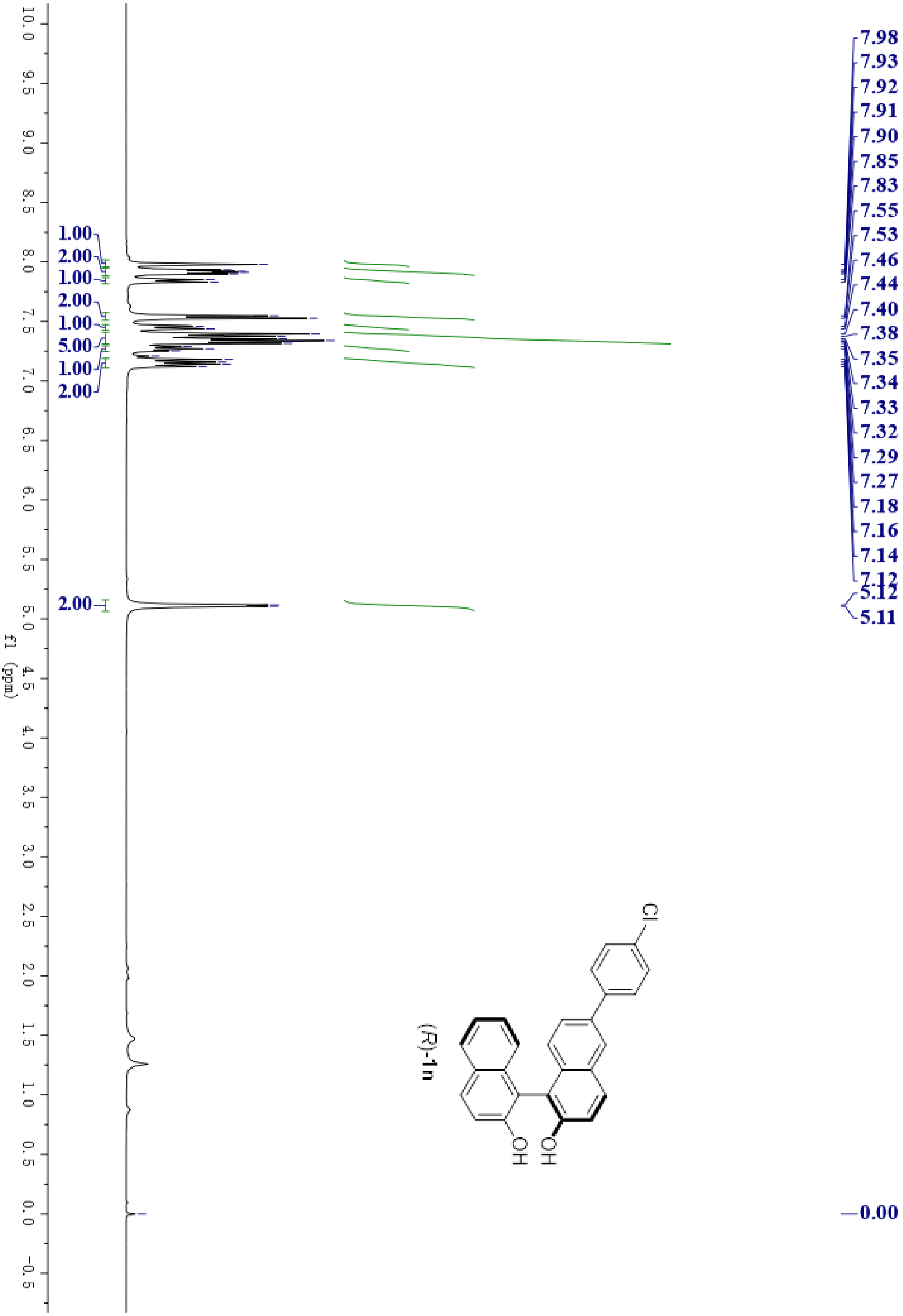
7.98
7.93
7.92
7.91
7.90
7.85
7.83
7.55
7.53
7.46
7.44
7.40
7.38
7.35
7.34
7.33
7.32
7.29
7.27
7.18
7.16
7.14
7.12
5.12
5.11
0.00
1.00
2.00
1.00
2.00
1.00
5.00
1.00
2.00
2.00
f1 (ppm)
Cl
OH
OH
(R)-1n

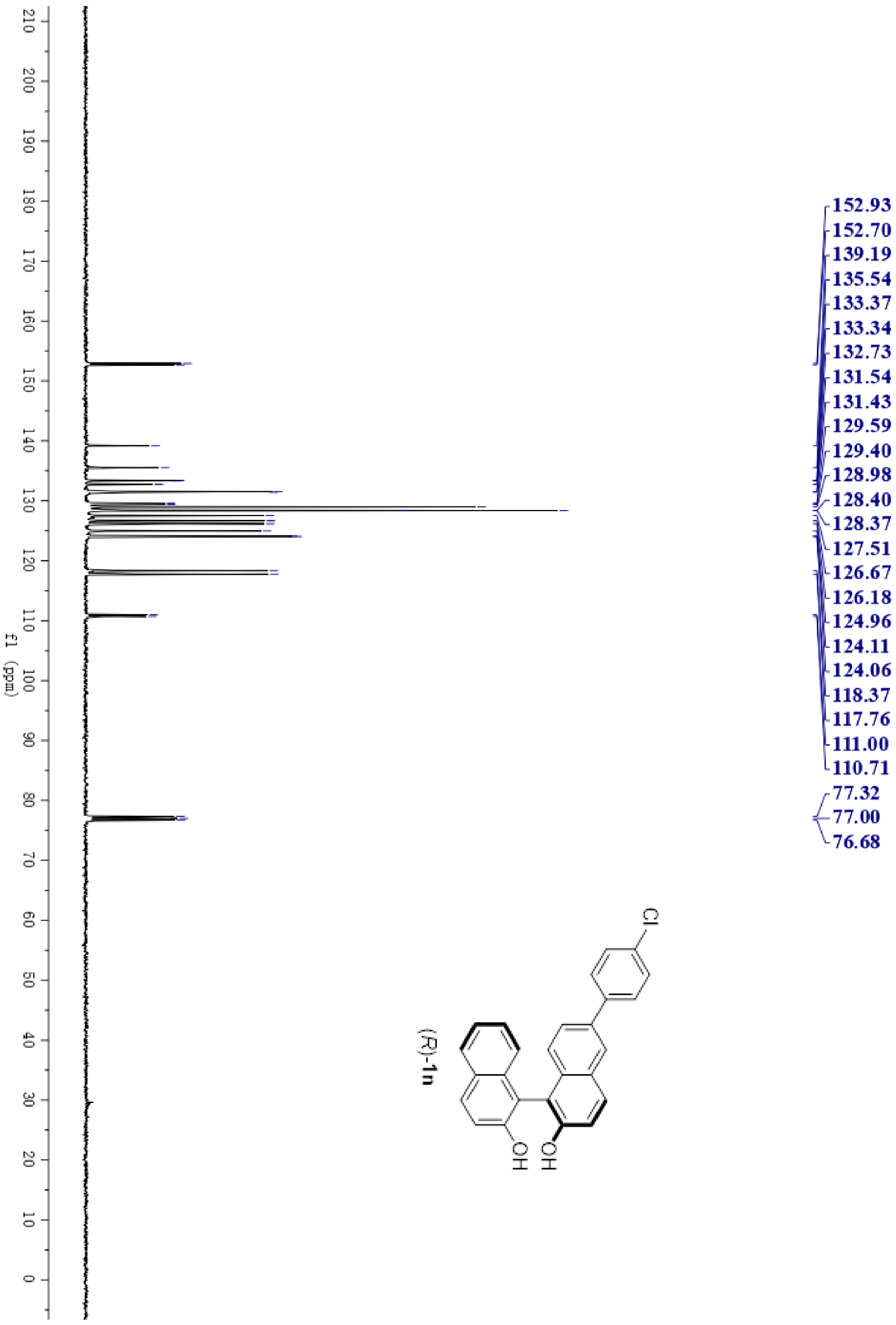
152.93
152.70
139.19
135.54
133.37
133.34
132.73
131.54
131.43
129.59
129.40
128.98
128.40
128.37
127.51
126.67
126.18
124.96
124.11
124.06
118.37
117.76
111.00
110.71
77.32
77.00
76.68
f1 (ppm)
Cl
OH
OH

(R)-1n

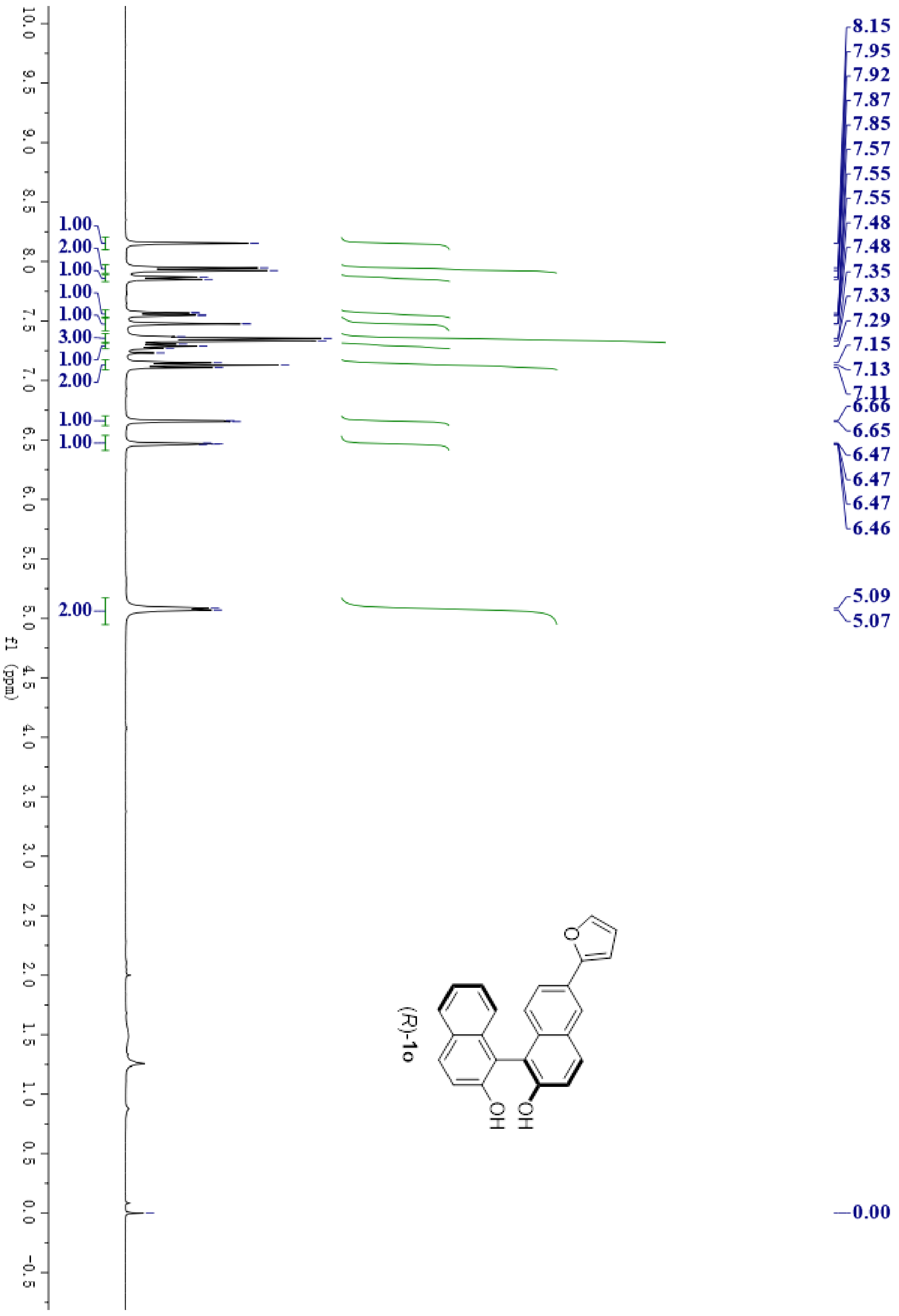
8.15
7.95
7.92
7.87
7.85
7.57
7.55
7.55
7.48
7.48
7.35
7.33
7.29
7.15
7.13
7.11
6.66
6.65
6.47
6.47
6.47
6.46
5.09
5.07
0.00
1.00
2.00
1.00
1.00
1.00
3.00
1.00
2.00
1.00
1.00
2.00
f1 (ppm)
(R)-1o

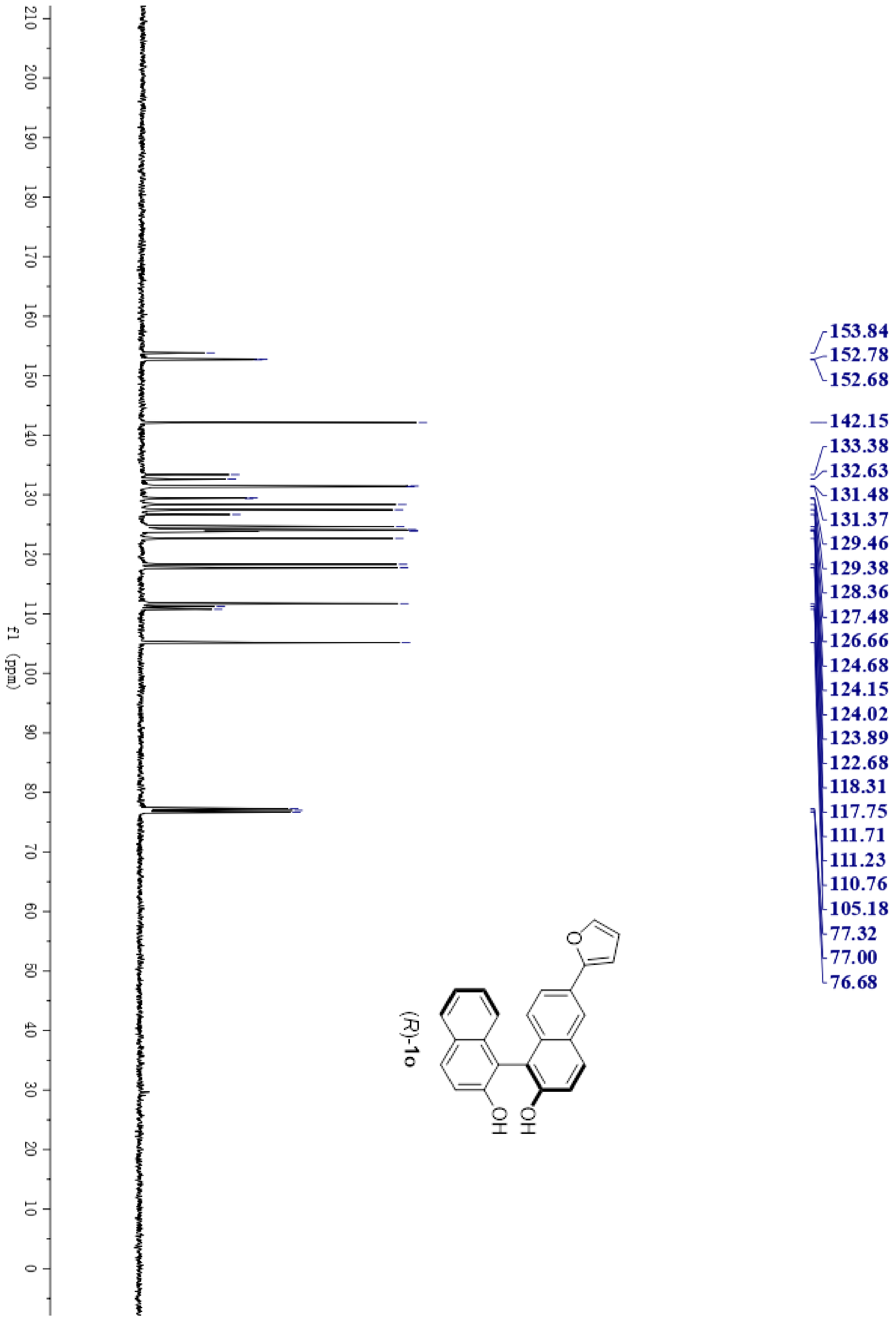
153.84
152.78
152.68
142.15
133.38
132.63
131.48
131.37
129.46
129.38
128.36
127.48
126.66
124.68
124.15
124.02
123.89
122.68
118.31
117.75
111.71
111.23
110.76
105.18
77.32
77.00
76.68
OH
OH
O
(R)-1o
f1 (ppm)

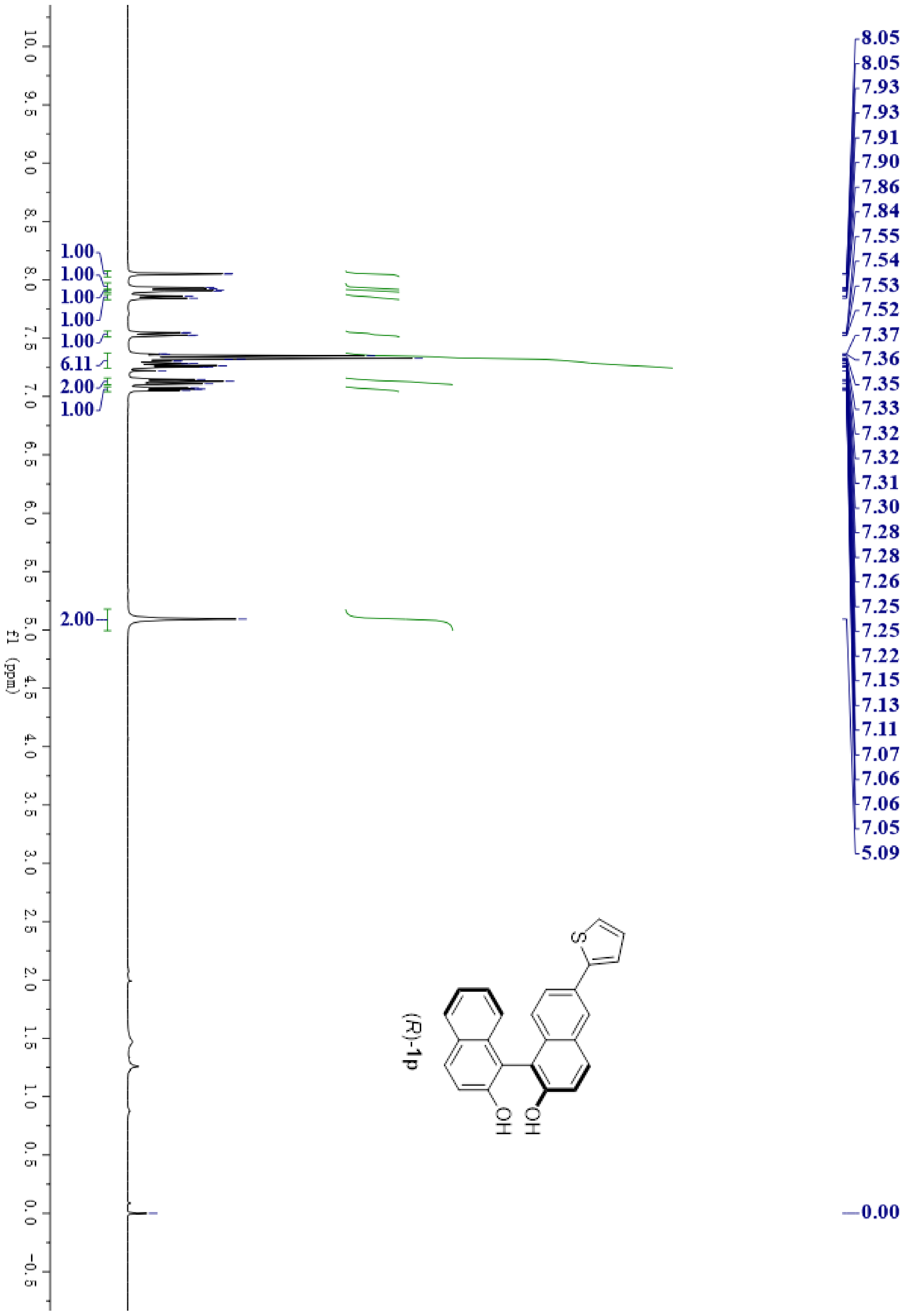
8.05
8.05
7.93
7.93
7.91
7.90
7.86
7.84
7.55
7.54
7.53
7.52
7.37
7.36
7.35
7.33
7.32
7.32
7.31
7.30
7.28
7.28
7.26
7.25
7.25
7.22
7.15
7.13
7.11
7.07
7.06
7.06
7.05
5.09
0.00
1.00
1.00
1.00
1.00
1.00
6.11
2.00
1.00
2.00
f1 (ppm)
(R)-1p
OH
OH
S

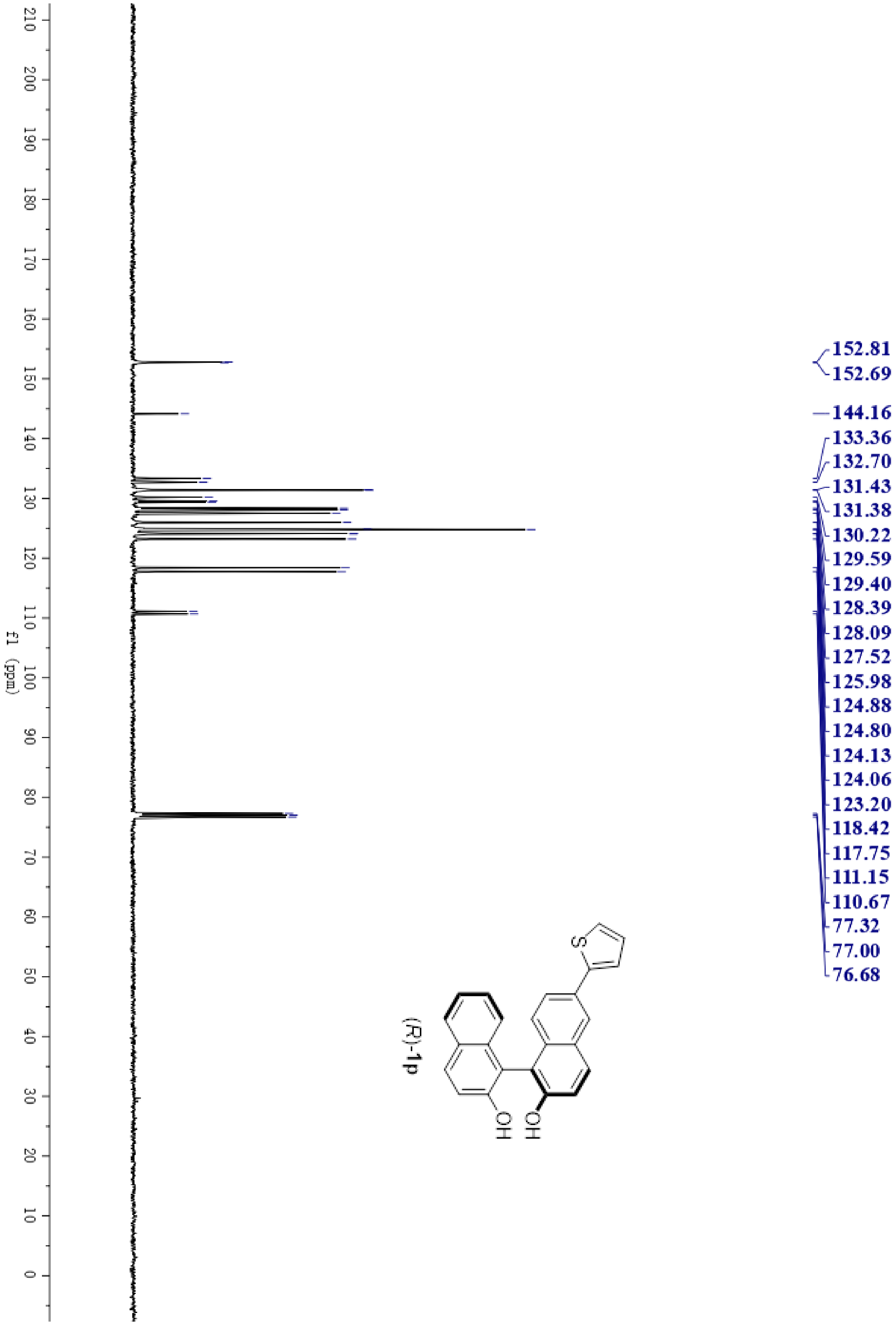
152.81
152.69
144.16
133.36
132.70
131.43
131.38
130.22
129.59
129.40
128.39
128.09
127.52
125.98
124.88
124.80
124.13
124.06
123.20
118.42
117.75
111.15
110.67
77.32
77.00
76.68
(R)-1p
OH
OH
S
f1 (ppm)

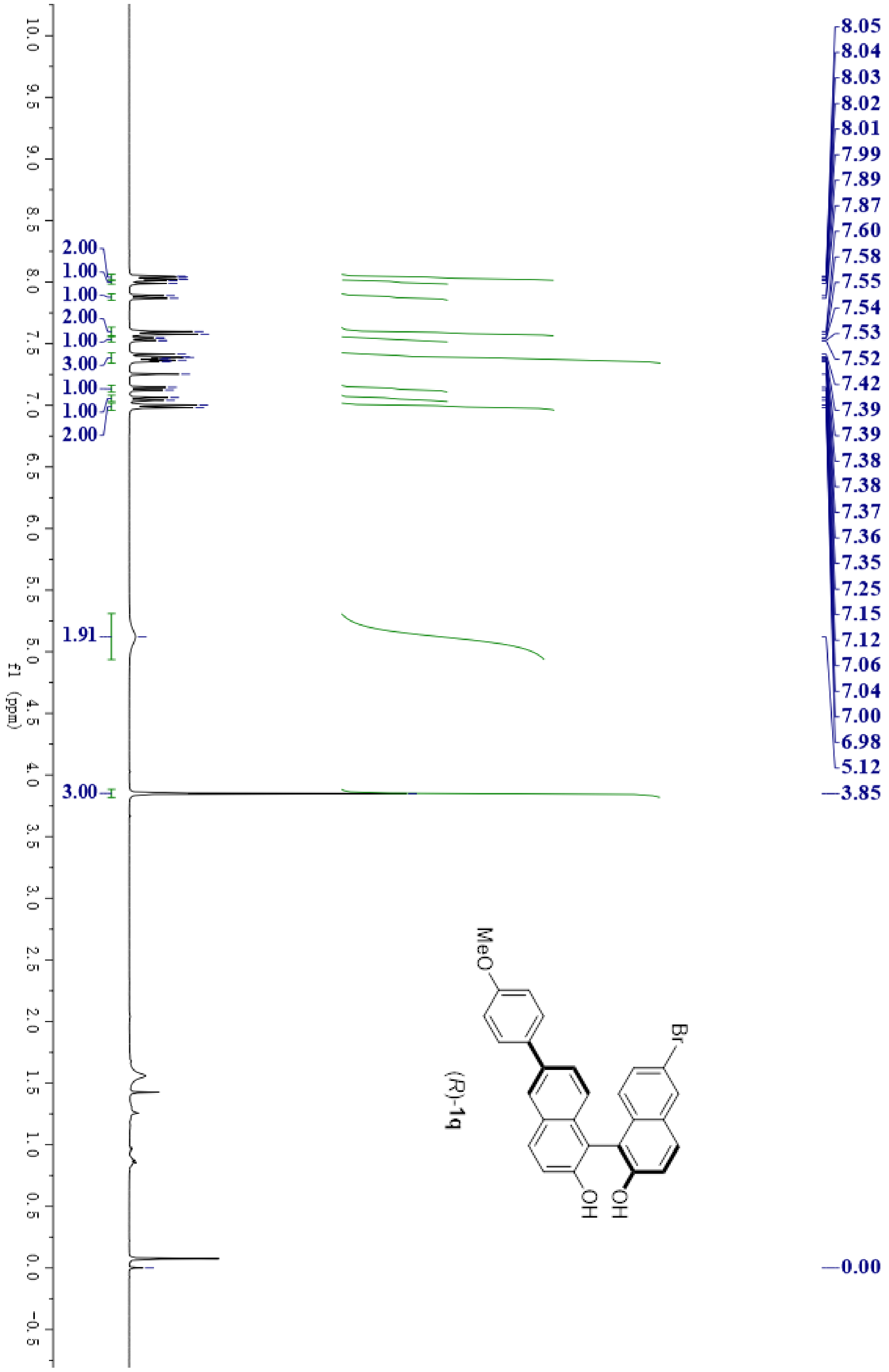
8.05
8.04
8.03
8.02
8.01
7.99
7.89
7.87
7.60
7.58
7.55
7.54
7.53
7.52
7.42
7.39
7.39
7.38
7.38
7.37
7.36
7.35
7.25
7.15
7.12
7.06
7.04
7.00
6.98
5.12
3.85
0.00
2.00
1.00
1.00
2.00
1.00
3.00
1.00
1.00
2.00
1.91
3.00
f1 (ppm)
MeO
Br
OH
OH
(R)-1q

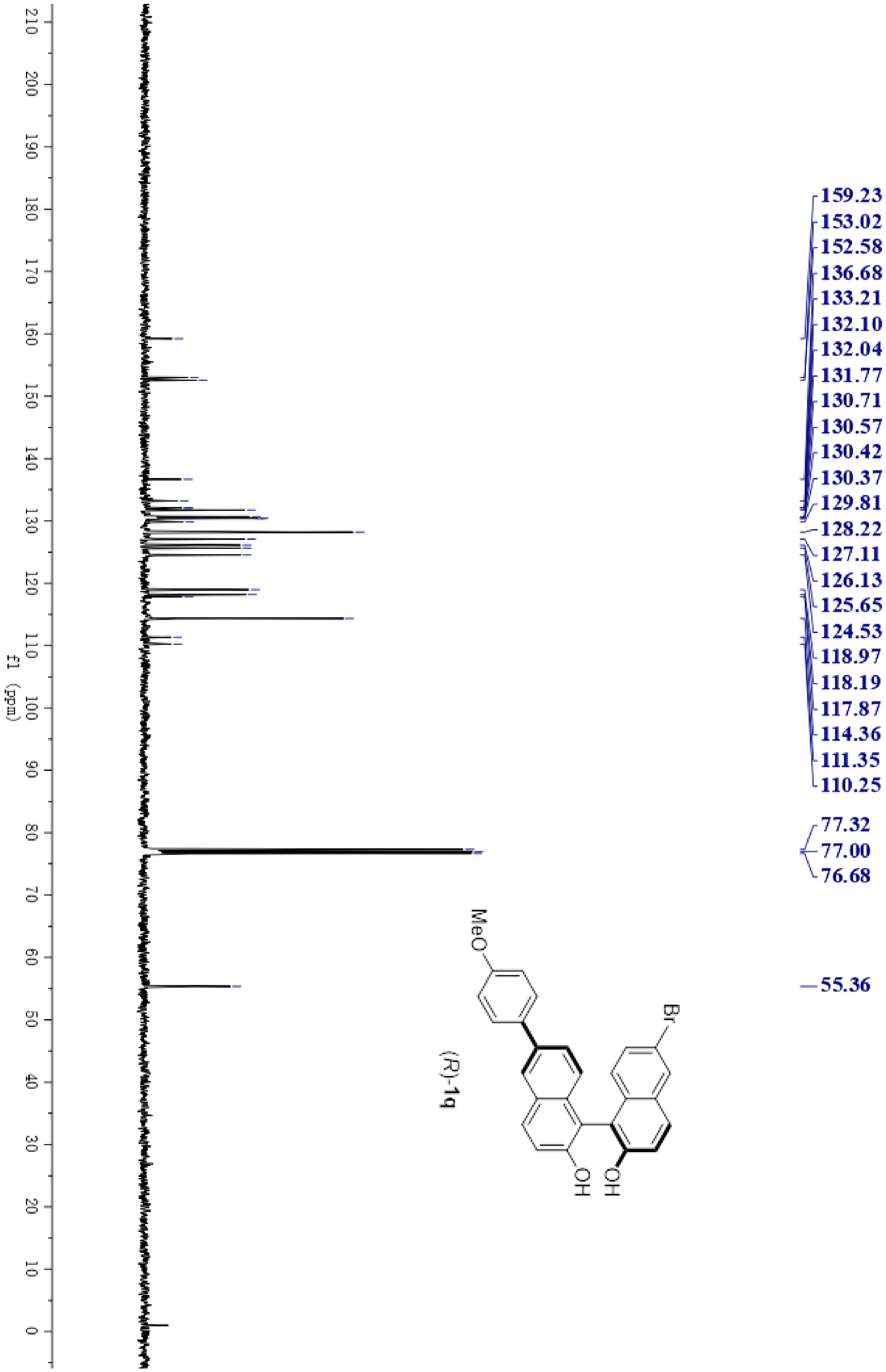
159.23
153.02
152.58
136.68
133.21
132.10
132.04
131.77
130.71
130.57
130.42
130.37
129.81
128.22
127.11
126.13
125.65
124.53
118.97
118.19
117.87
114.36
111.35
110.25
77.32
77.00
76.68
55.36
f1 (ppm)
MeO
Br
OH
OH
(R)-1q

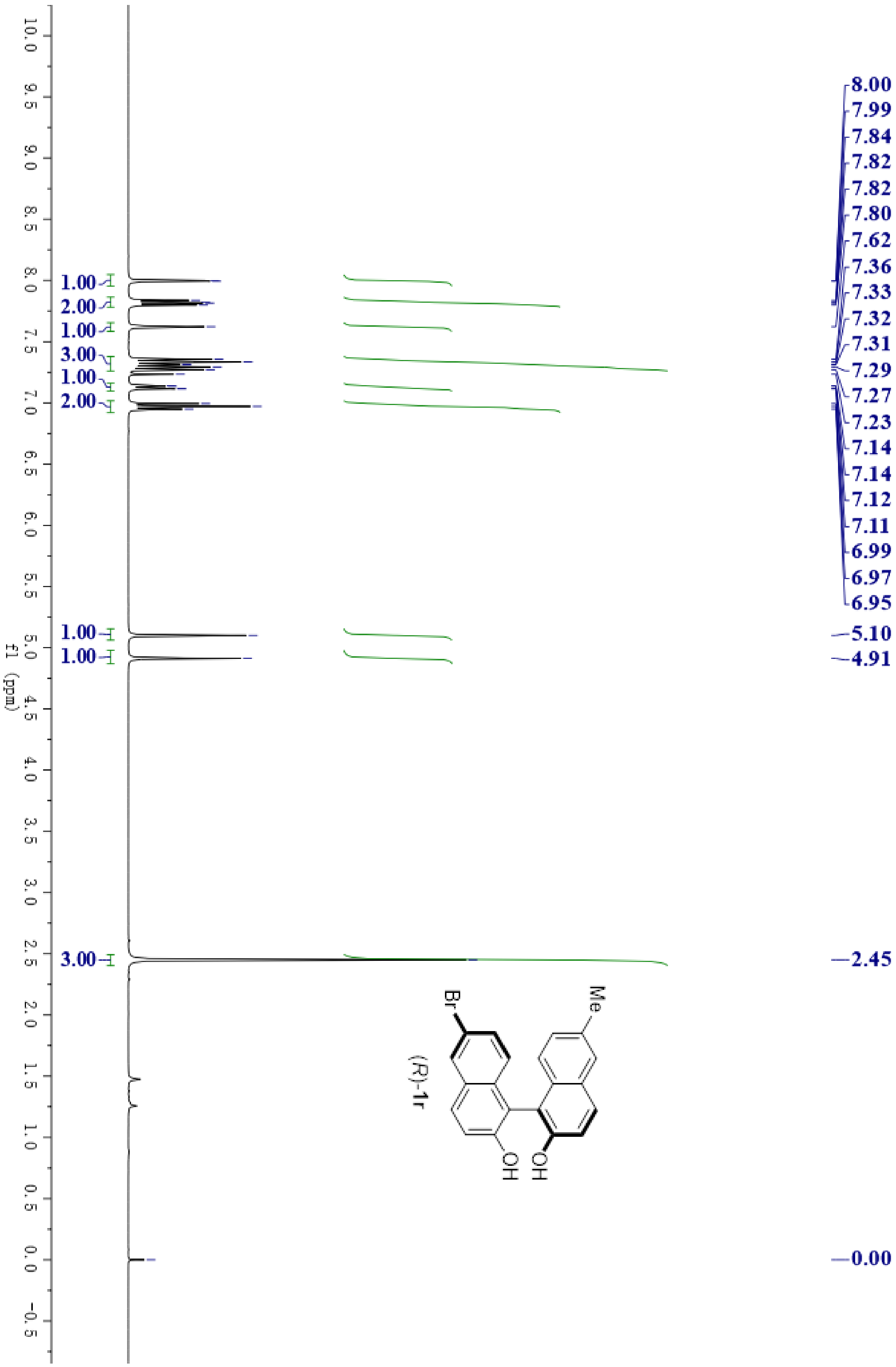
8.00
7.99
7.84
7.82
7.82
7.80
7.62
7.36
7.33
7.32
7.31
7.29
7.27
7.23
7.14
7.14
7.12
7.11
6.99
6.97
6.95
5.10
4.91
2.45
0.00
1.00
2.00
1.00
3.00
1.00
2.00
1.00
1.00
3.00
f1 (ppm)
Br
Me
OH
OH
(R)-1r

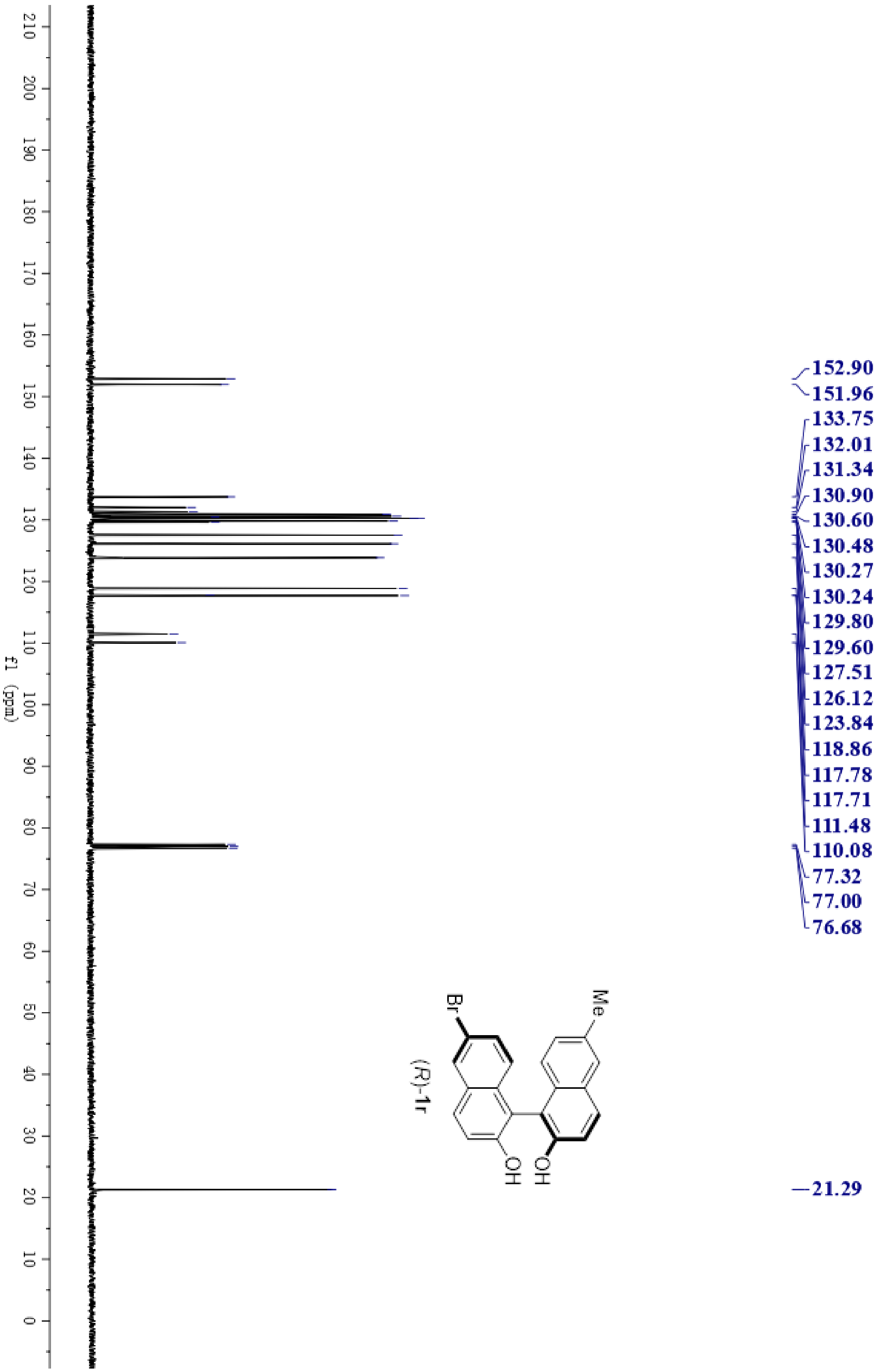
152.90
151.96
133.75
132.01
131.34
130.90
130.60
130.48
130.27
130.24
129.80
129.60
127.51
126.12
123.84
118.86
117.78
117.71
111.48
110.08
77.32
77.00
76.68
21.29
f1 (ppm)
Br
Me
OH
OH
(R)-1r

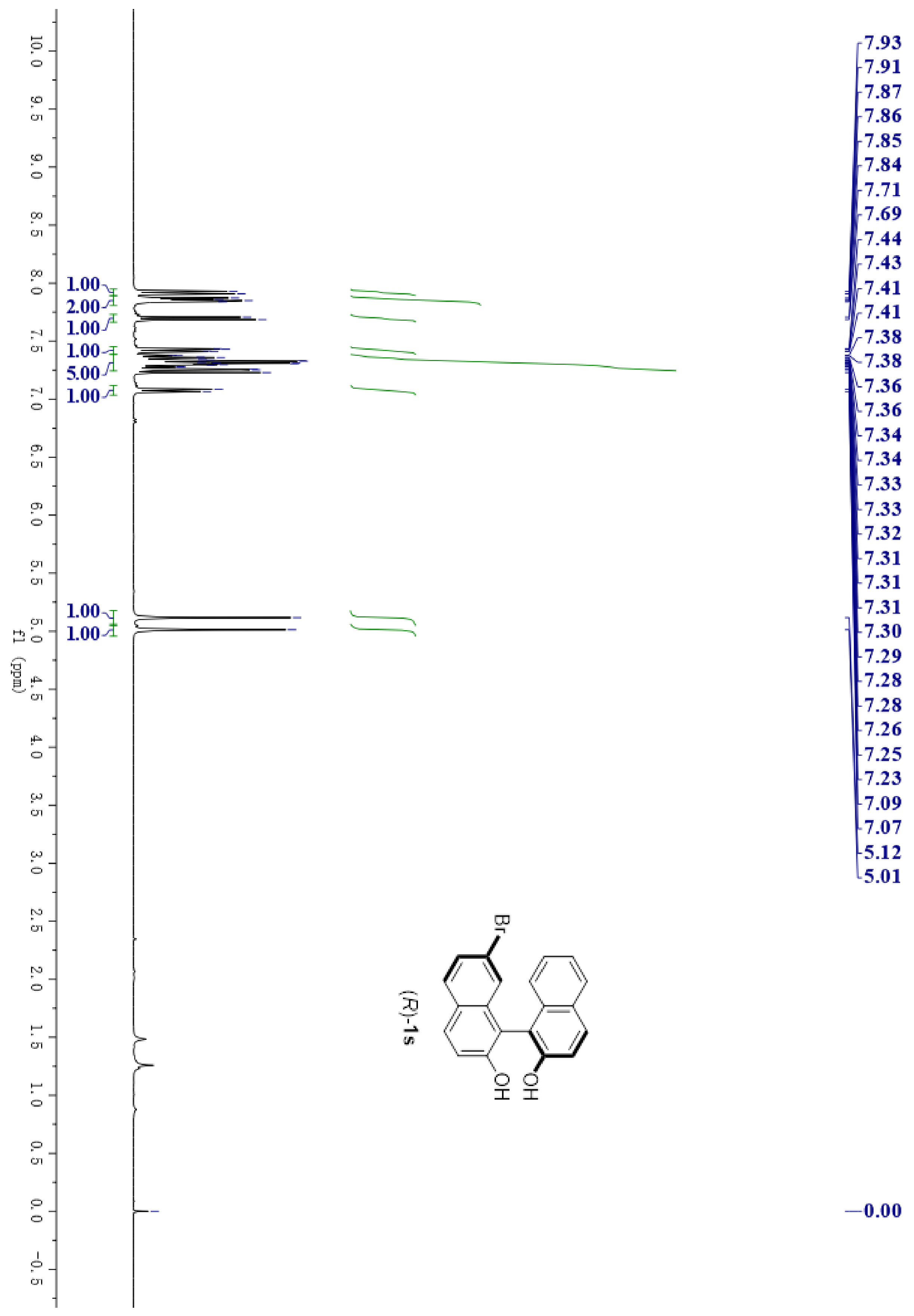
7.93
7.91
7.87
7.86
7.85
7.84
7.71
7.69
7.44
7.43
7.41
7.41
7.38
7.38
7.36
7.36
7.34
7.34
7.33
7.33
7.32
7.31
7.31
7.31
7.30
7.29
7.28
7.28
7.26
7.25
7.23
7.09
7.07
5.12
5.01
0.00
1.00
2.00
1.00
1.00
5.00
1.00
1.00
1.00
f1 (ppm)
Br
OH
OH
(R)-1s

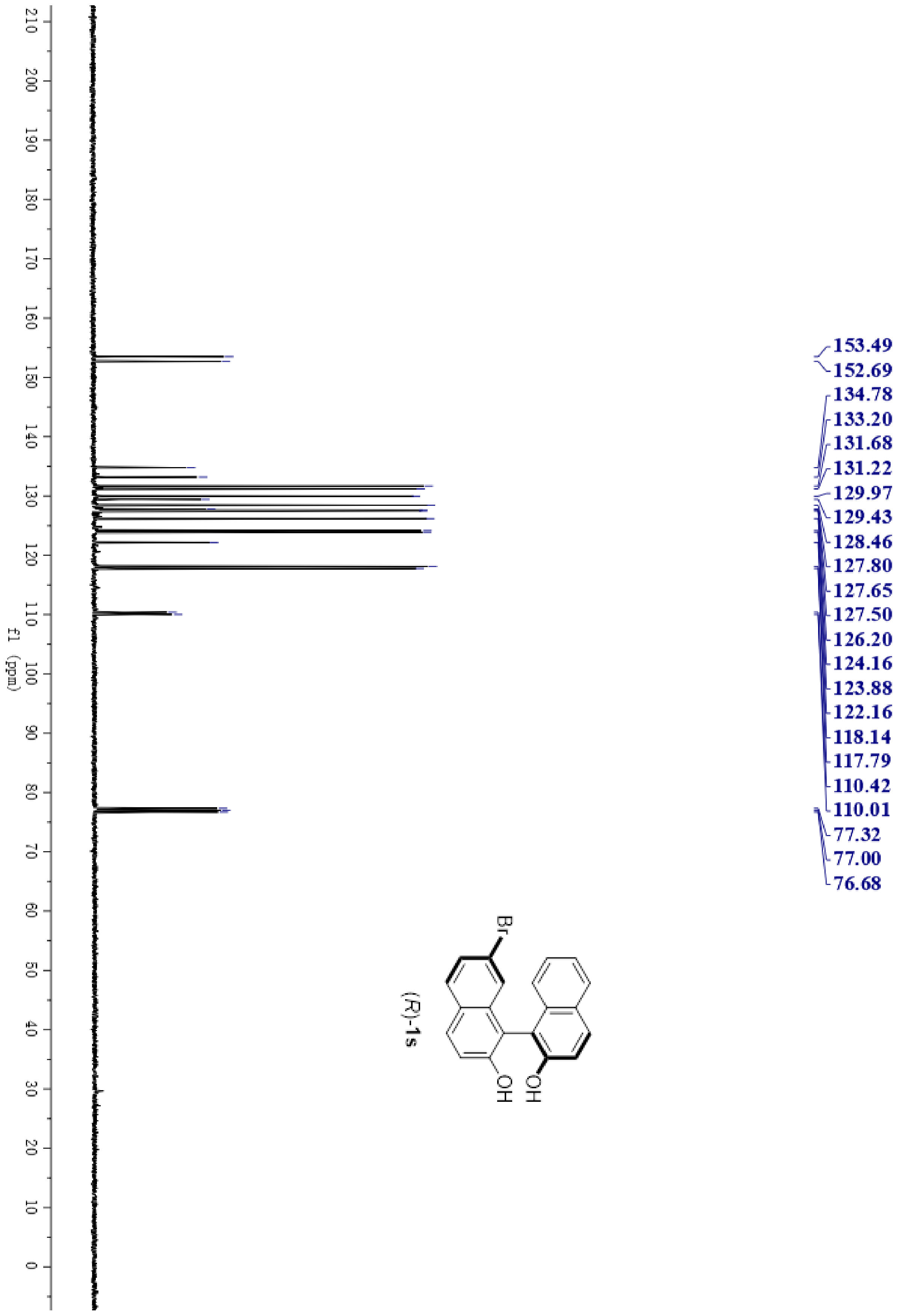

153.49
152.69
134.78
133.20
131.68
131.22
129.97
129.43
128.46
127.80
127.65
127.50
126.20
124.16
123.88
122.16
118.14
117.79
110.42
110.01
77.32
77.00
76.68
f1 (ppm)
Br
OH
OH
(R)-1s

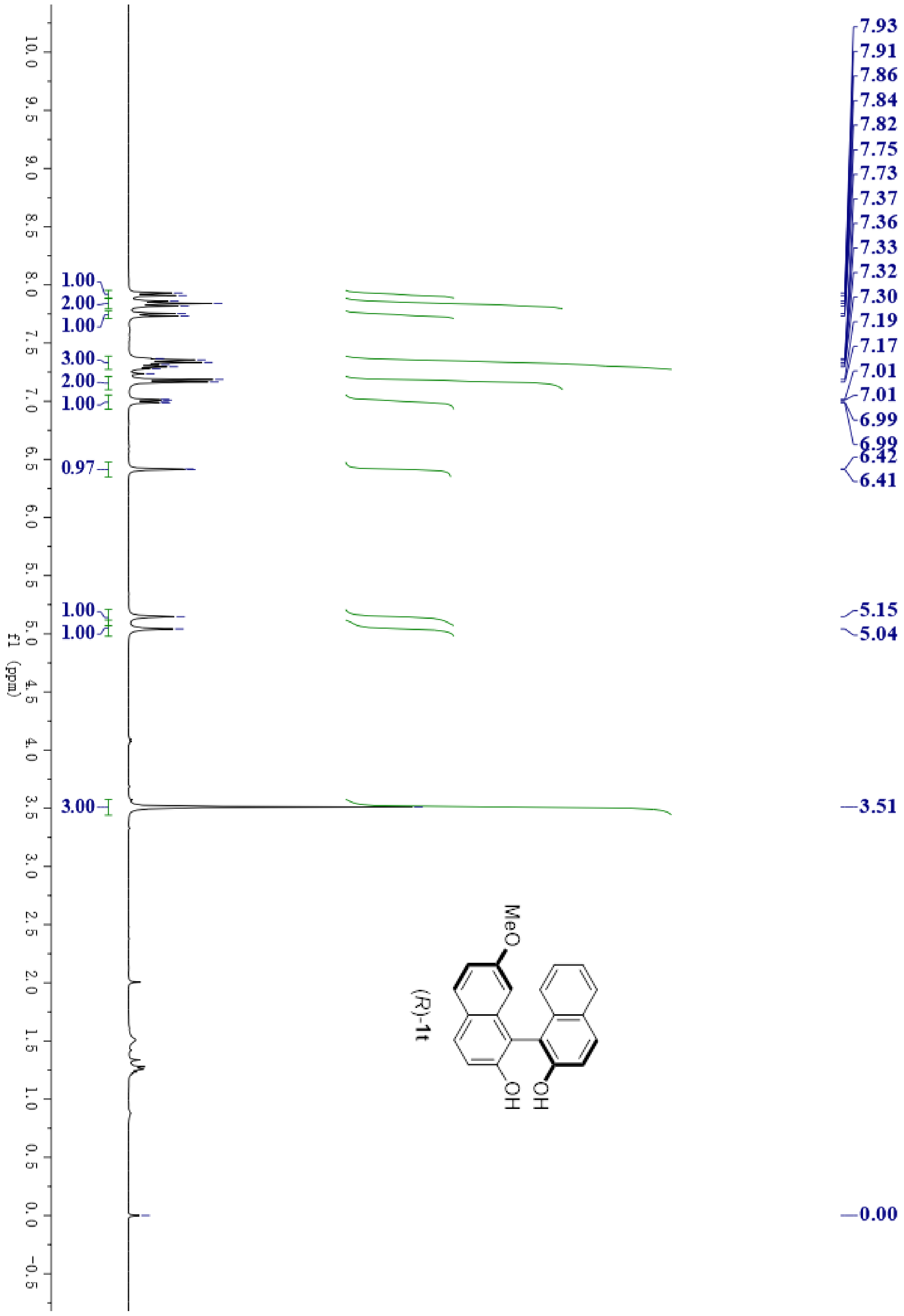
7.93
7.91
7.86
7.84
7.82
7.75
7.73
7.37
7.36
7.33
7.32
7.30
7.19
7.17
7.01
7.01
6.99
6.99
6.42
6.41
5.15
5.04
3.51
0.00
1.00
2.00
1.00
3.00
2.00
1.00
0.97
1.00
1.00
3.00
f1 (ppm)
MeO
OH
OH
(R)-1t

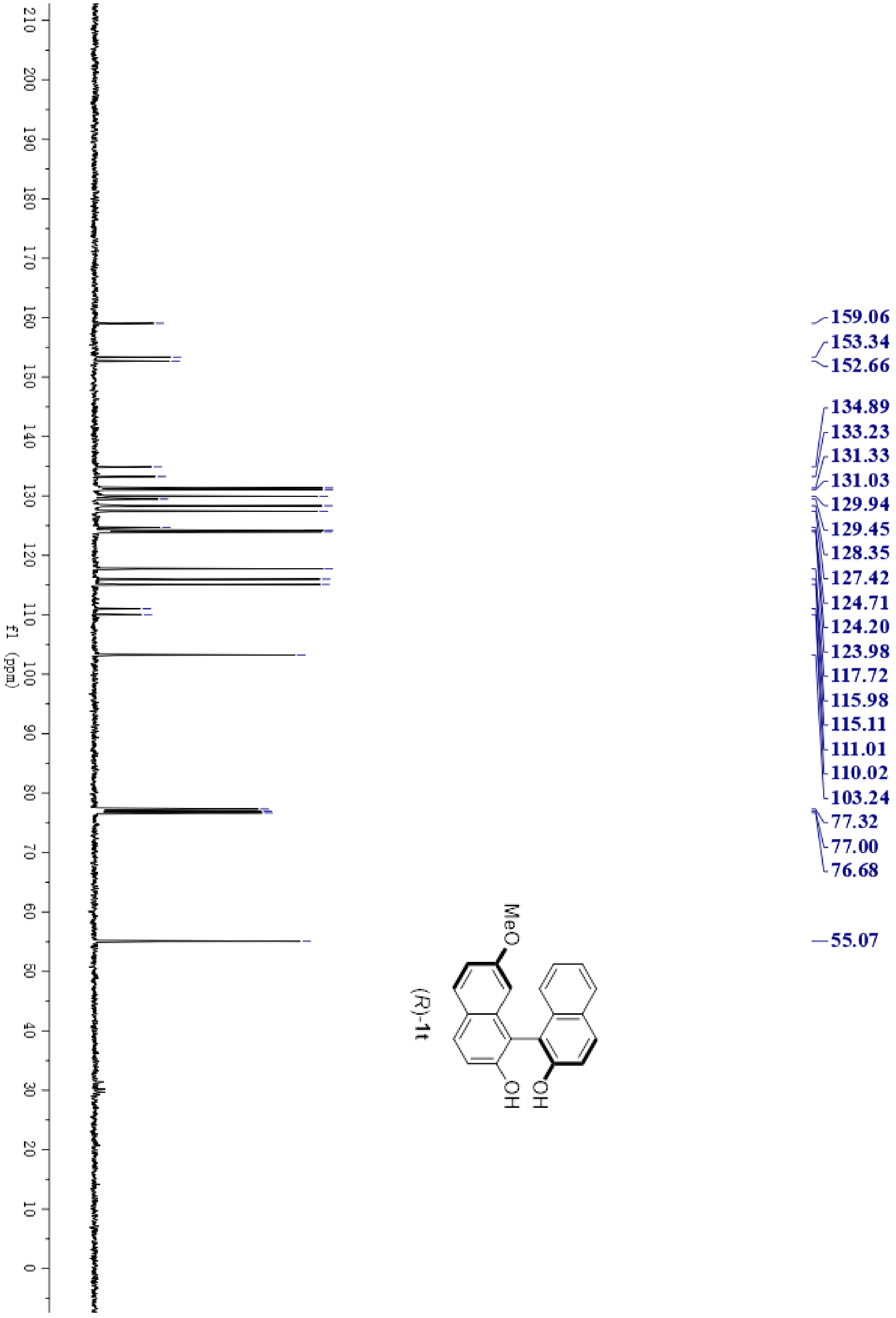
159.06
153.34
152.66
134.89
133.23
131.33
131.03
129.94
129.45
128.35
127.42
124.71
124.20
123.98
117.72
115.98
115.11
111.01
110.02
103.24
77.32
77.00
76.68
55.07
MeO
OH
OH
(R)-1t
f1 (ppm)
210
200
190
180
170
160
150
140
130
120
110
100
90
80
70
60
50
40
30
20
10
0

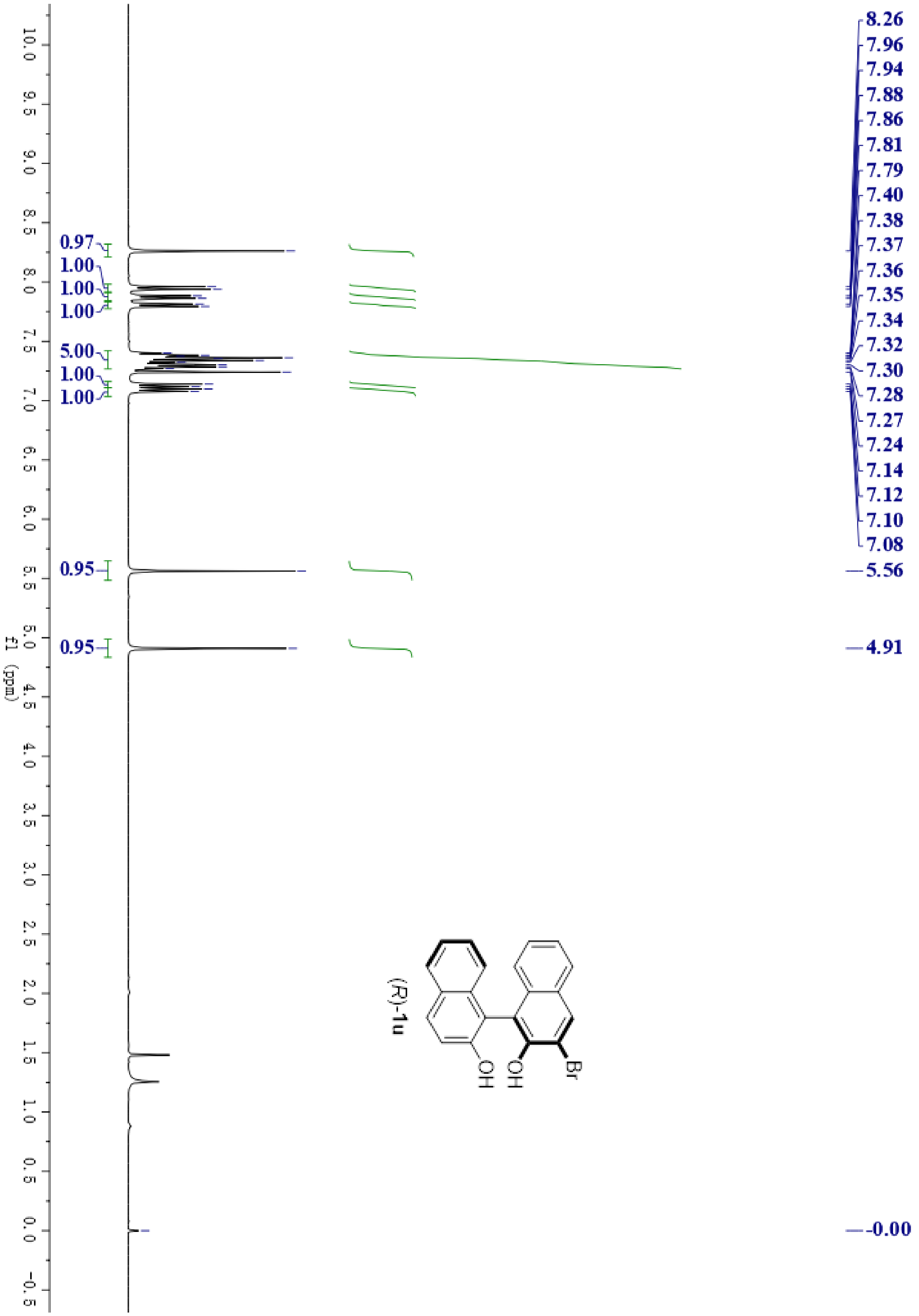
8.26
7.96
7.94
7.88
7.86
7.81
7.79
7.40
7.38
7.37
7.36
7.35
7.34
7.32
7.30
7.28
7.27
7.24
7.14
7.12
7.10
7.08
5.56
4.91
-0.00
0.97
1.00
1.00
1.00
5.00
1.00
1.00
0.95
0.95
f1 (ppm)
(R)-1u
OH
OH
Br

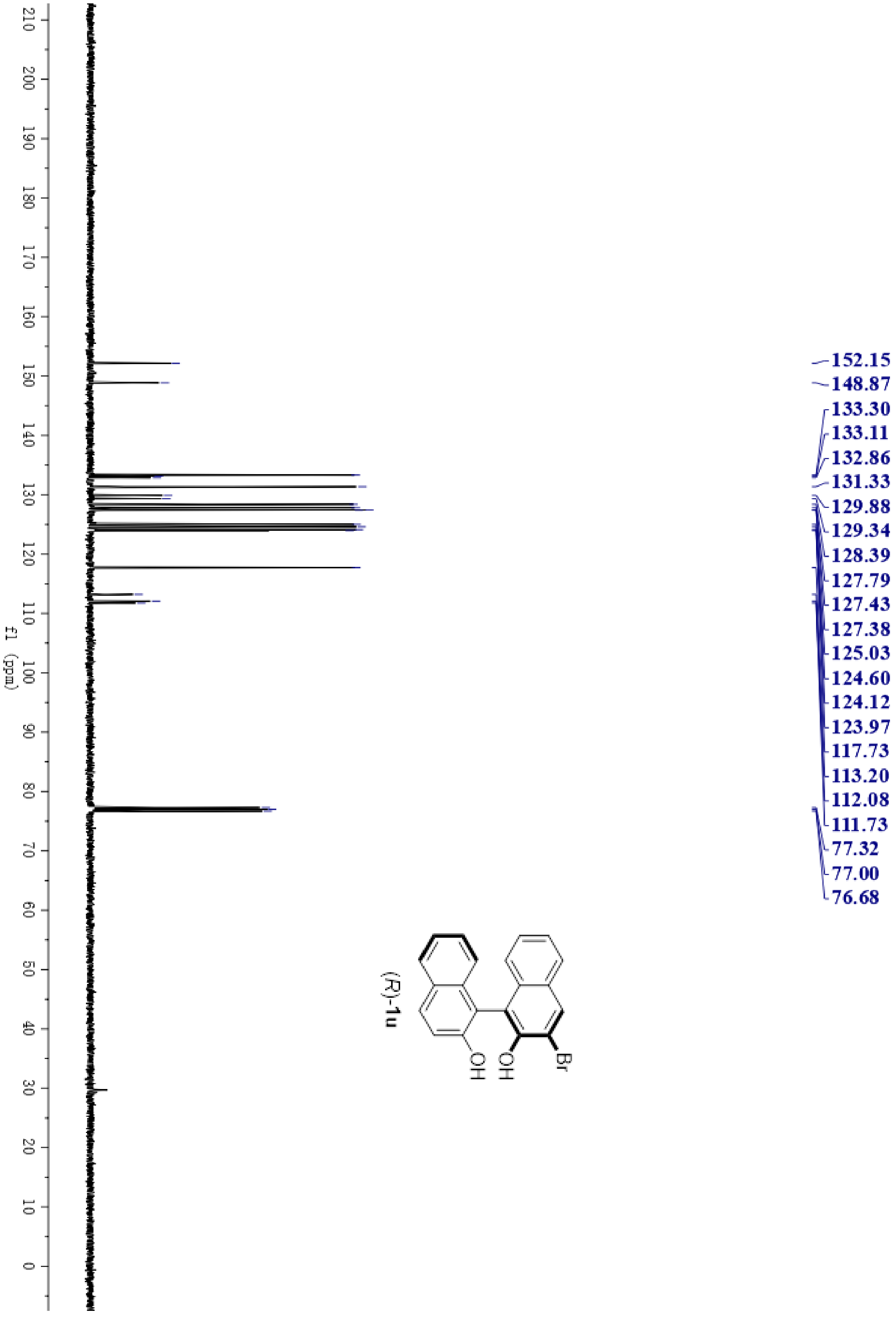
152.15
148.87
133.30
133.11
132.86
131.33
129.88
129.34
128.39
127.79
127.43
127.38
125.03
124.60
124.12
123.97
117.73
113.20
112.08
111.73
77.32
77.00
76.68
210
200
190
180
170
160
150
140
130
120
110
100
90
80
70
60
50
40
30
20
10
0
f1 (ppm)
(R)-1u
OH
OH
Br

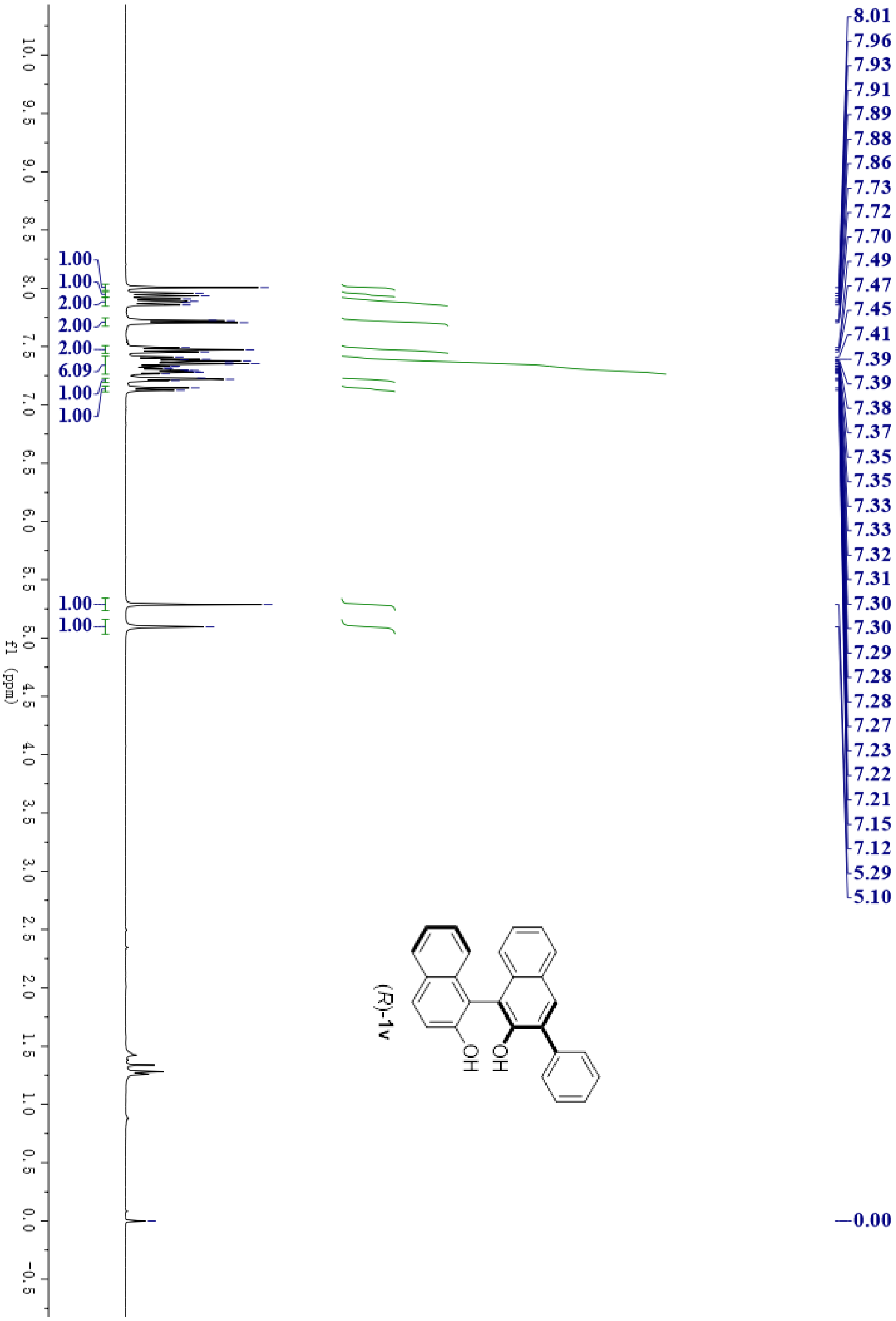
8.01
7.96
7.93
7.91
7.89
7.88
7.86
7.73
7.72
7.70
7.49
7.47
7.45
7.41
7.39
7.39
7.38
7.37
7.35
7.35
7.33
7.33
7.32
7.31
7.30
7.30
7.29
7.28
7.28
7.27
7.23
7.22
7.21
7.15
7.12
5.29
5.10
0.00
1.00
1.00
2.00
2.00
2.00
6.09
1.00
1.00
1.00
1.00
f1 (ppm)
OH
OH
(R)-1v

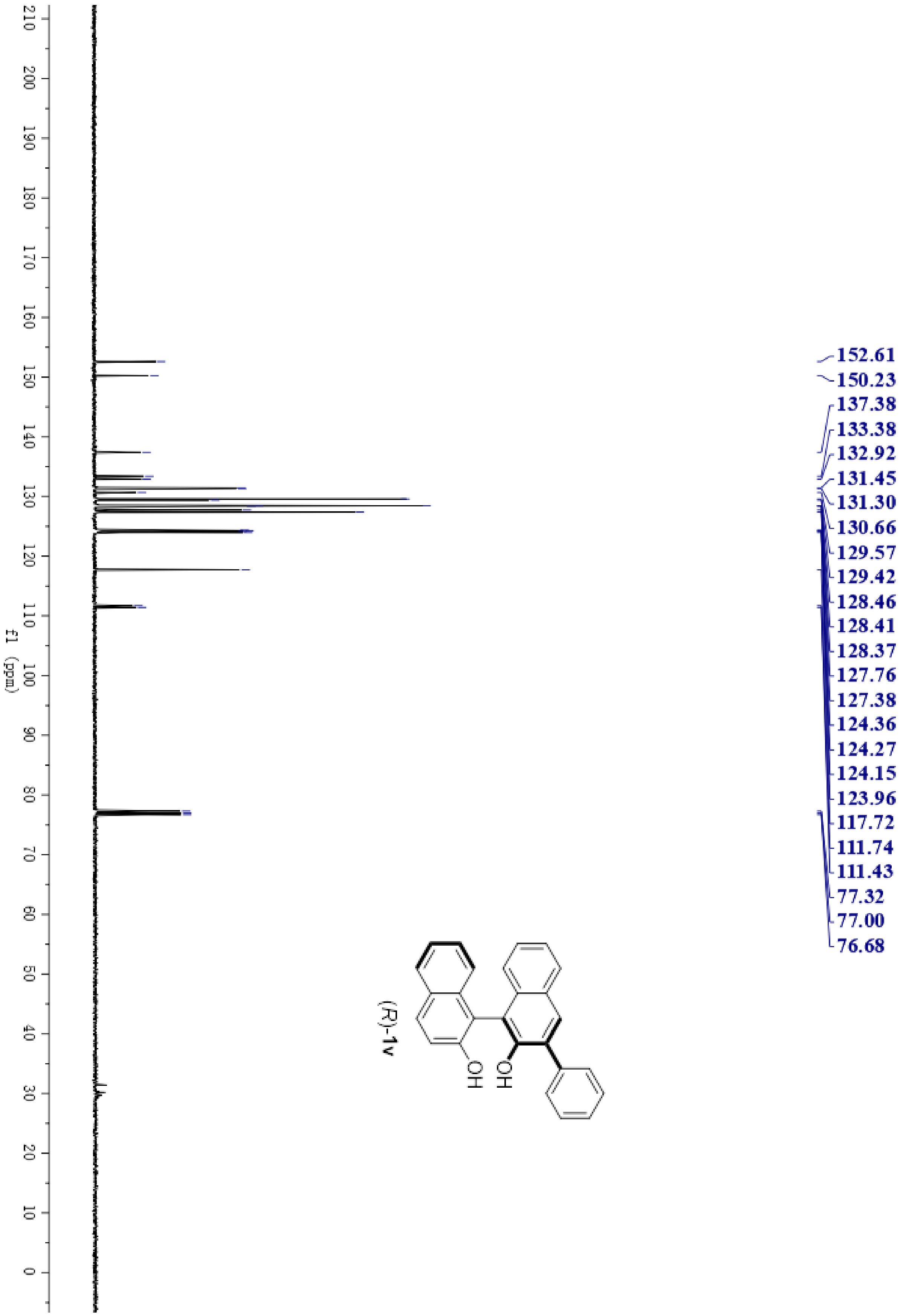
152.61
150.23
137.38
133.38
132.92
131.45
131.30
130.66
129.57
129.42
128.46
128.41
128.37
127.76
127.38
124.36
124.27
124.15
123.96
117.72
111.74
111.43
77.32
77.00
76.68
OH
OH
(R)-1v
f1 (ppm)

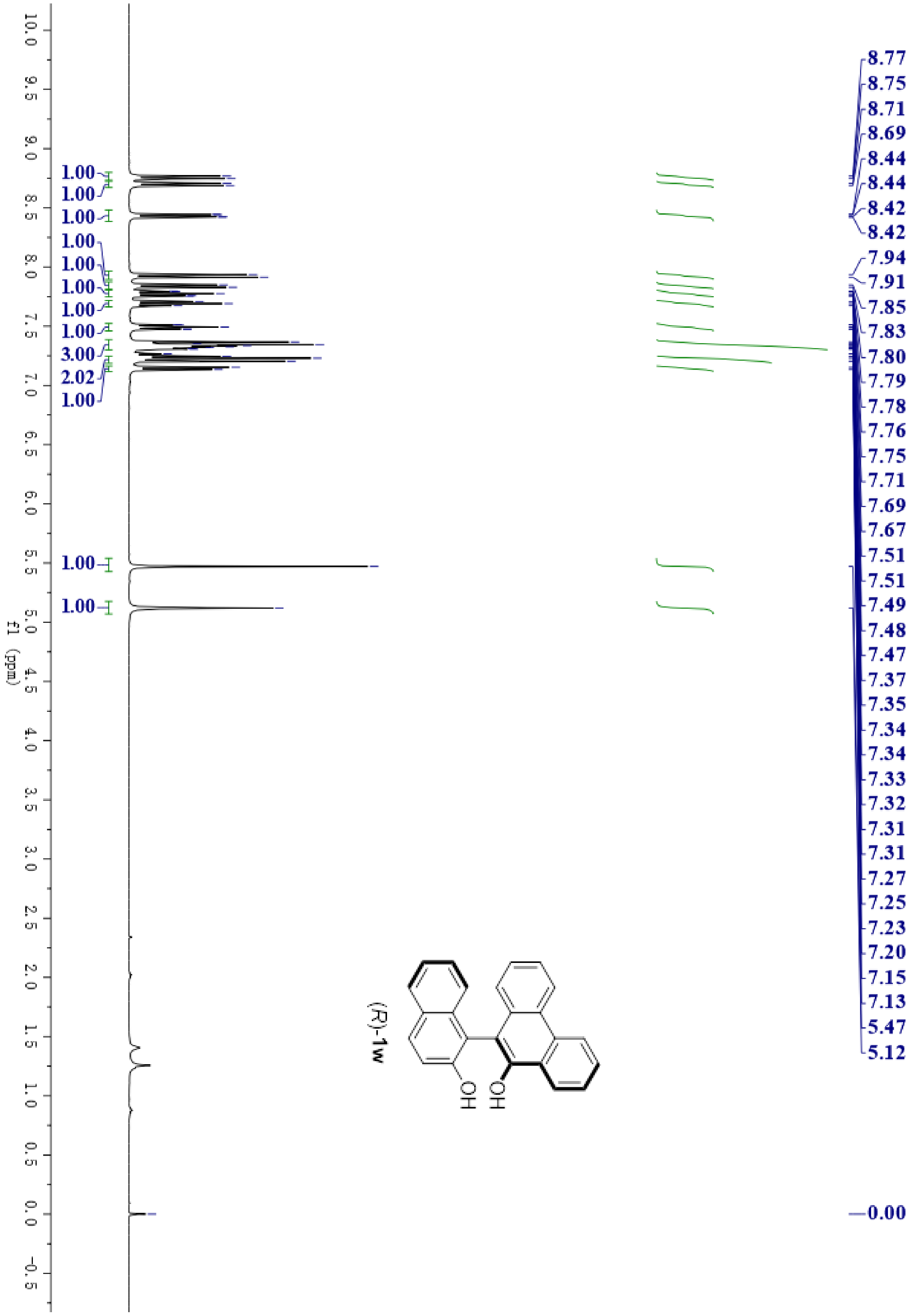
8.77
8.75
8.71
8.69
8.44
8.44
8.42
8.42
7.94
7.91
7.85
7.83
7.80
7.79
7.78
7.76
7.75
7.71
7.69
7.67
7.51
7.51
7.49
7.48
7.47
7.37
7.35
7.34
7.34
7.33
7.32
7.31
7.31
7.27
7.25
7.23
7.20
7.15
7.13
5.47
5.12
0.00
1.00
1.00
1.00
1.00
1.00
1.00
1.00
1.00
3.00
2.02
1.00
1.00
1.00
f1 (ppm)
(R)-1w
OH
OH

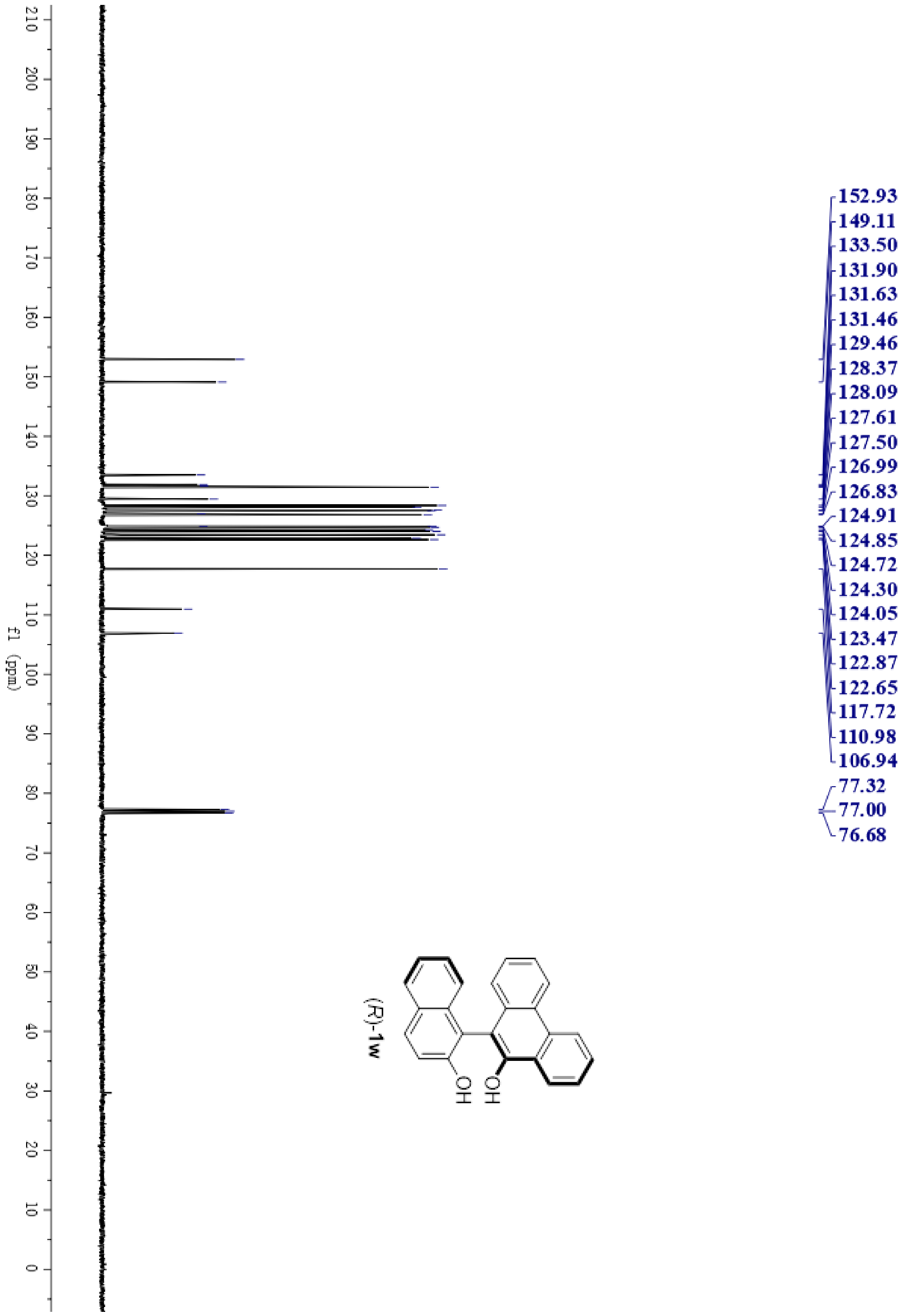
152.93
149.11
133.50
131.90
131.63
131.46
129.46
128.37
128.09
127.61
127.50
126.99
126.83
124.91
124.85
124.72
124.30
124.05
123.47
122.87
122.65
117.72
110.98
106.94
77.32
77.00
76.68
f1 (ppm)
OH
OH
(R)-1w

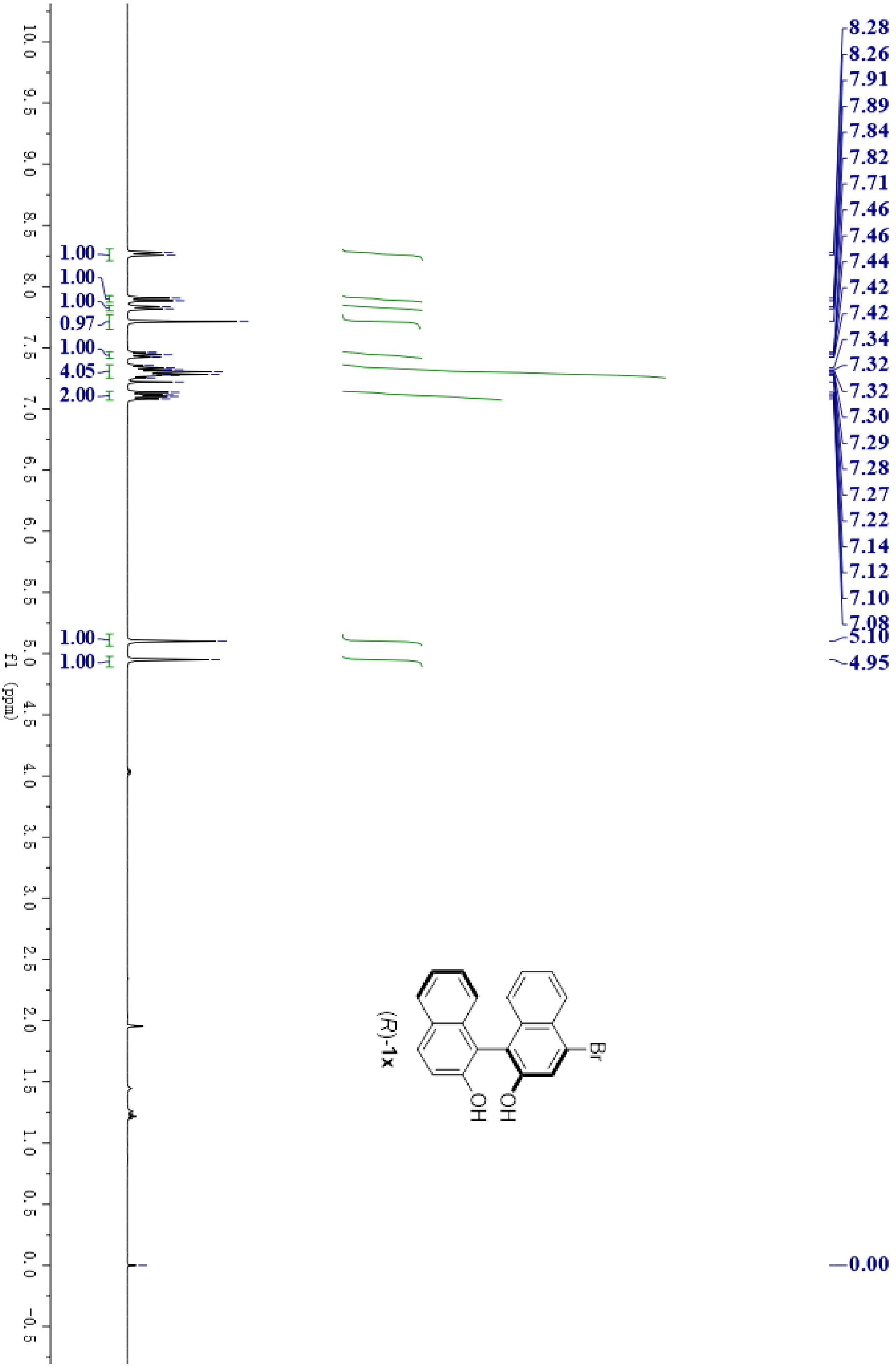
8.28
8.26
7.91
7.89
7.84
7.82
7.71
7.46
7.46
7.44
7.42
7.42
7.34
7.32
7.32
7.30
7.29
7.28
7.27
7.22
7.14
7.12
7.10
7.08
5.10
4.95
0.00
1.00
1.00
1.00
0.97
1.00
4.05
2.00
1.00
1.00
f1 (ppm)
(R)-1x
OH
OH
Br

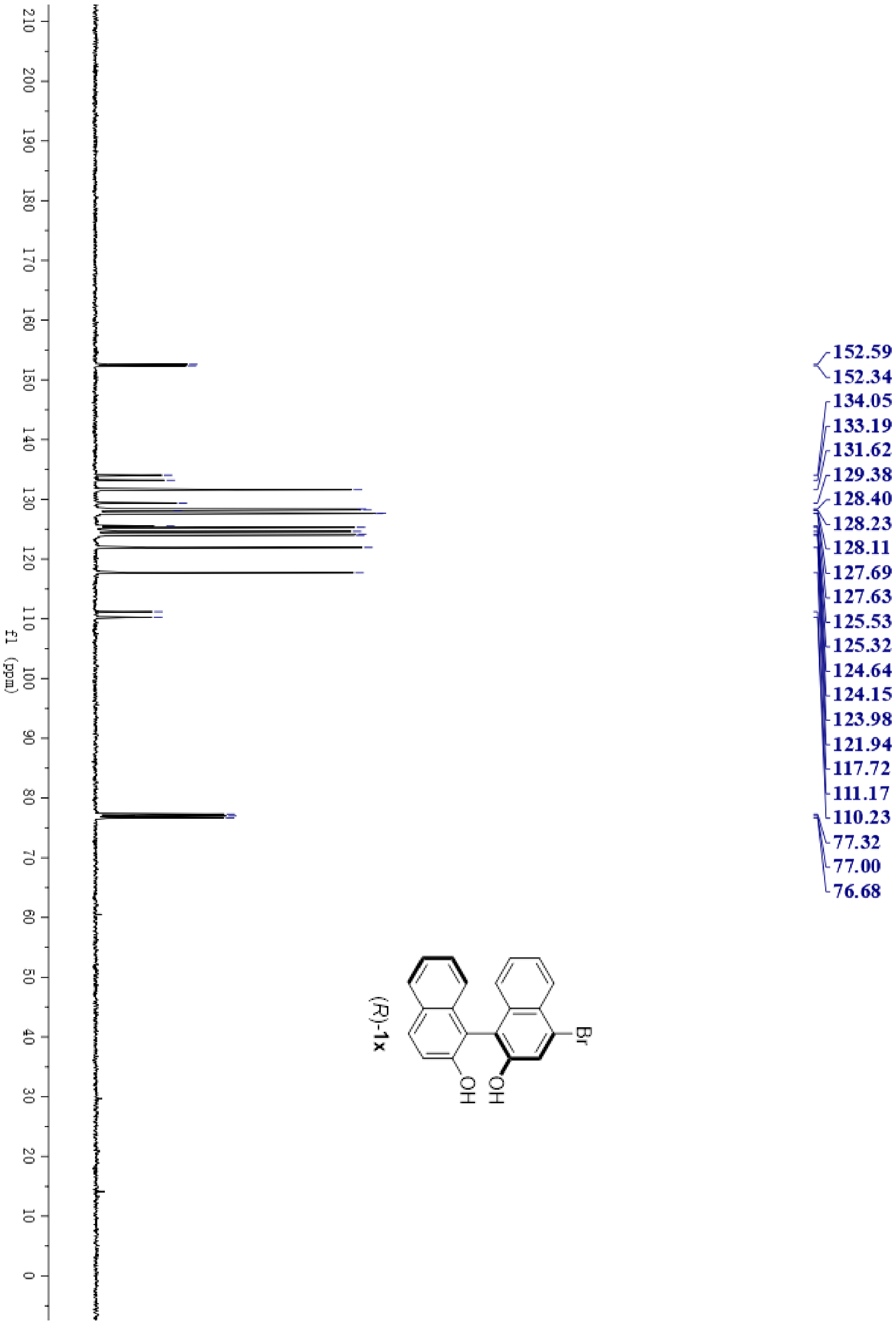

152.59
152.34
134.05
133.19
131.62
129.38
128.40
128.23
128.11
127.69
127.63
125.53
125.32
124.64
124.15
123.98
121.94
117.72
111.17
110.23
77.32
77.00
76.68
210
200
190
180
170
160
150
140
130
120
110
100
90
80
70
60
50
40
30
20
10
0
f1 (ppm)
Br
OH
OH
(R)-1x

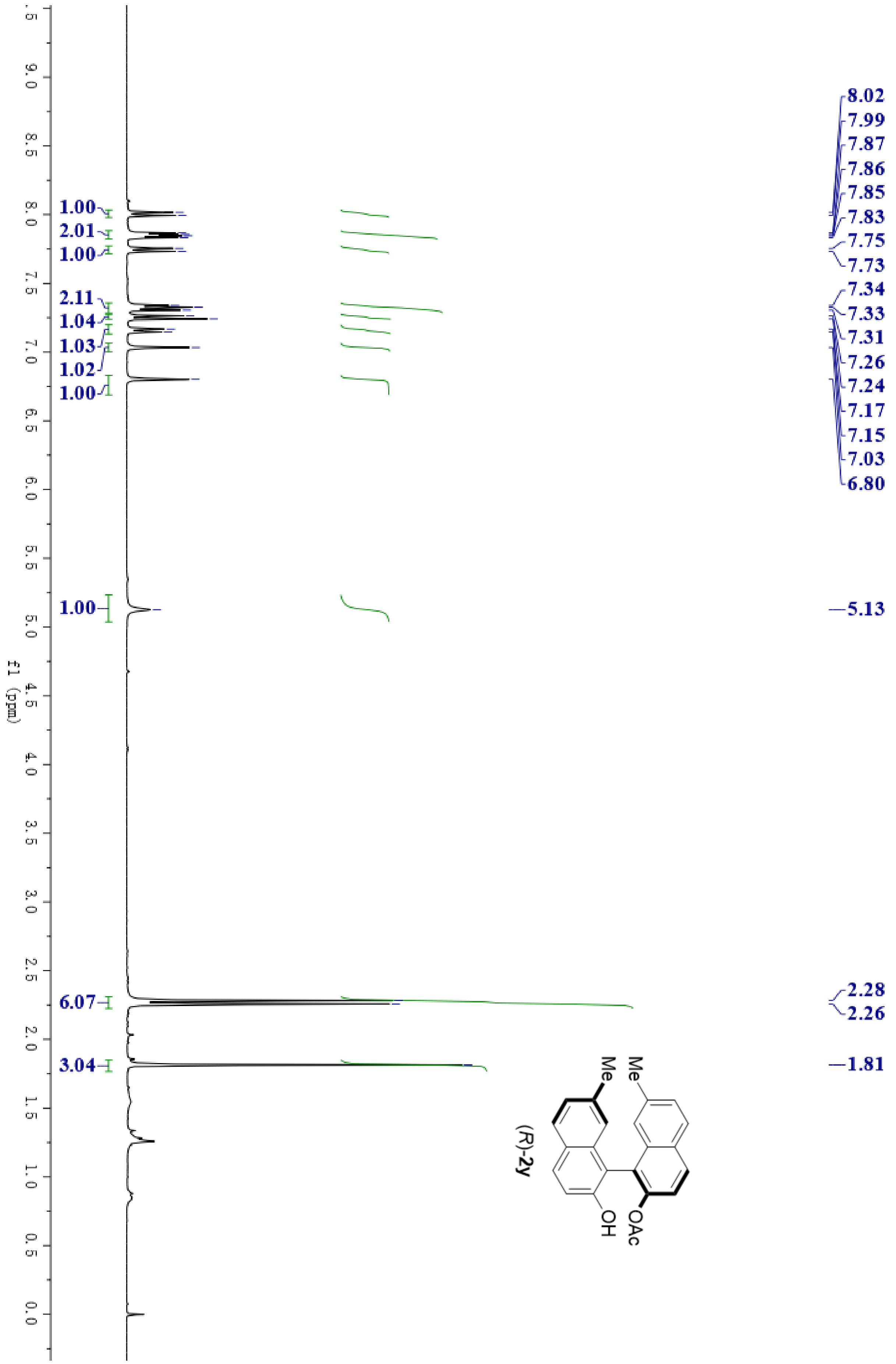
8.02
7.99
7.87
7.86
7.85
7.83
7.75
7.73
7.34
7.33
7.31
7.26
7.24
7.17
7.15
7.03
6.80
5.13
2.28
2.26
1.81
1.00
2.01
1.00
2.11
1.04
1.03
1.02
1.00
1.00
6.07
3.04
f1 (ppm)
Me
Me
OAc
OH
(R)-2y

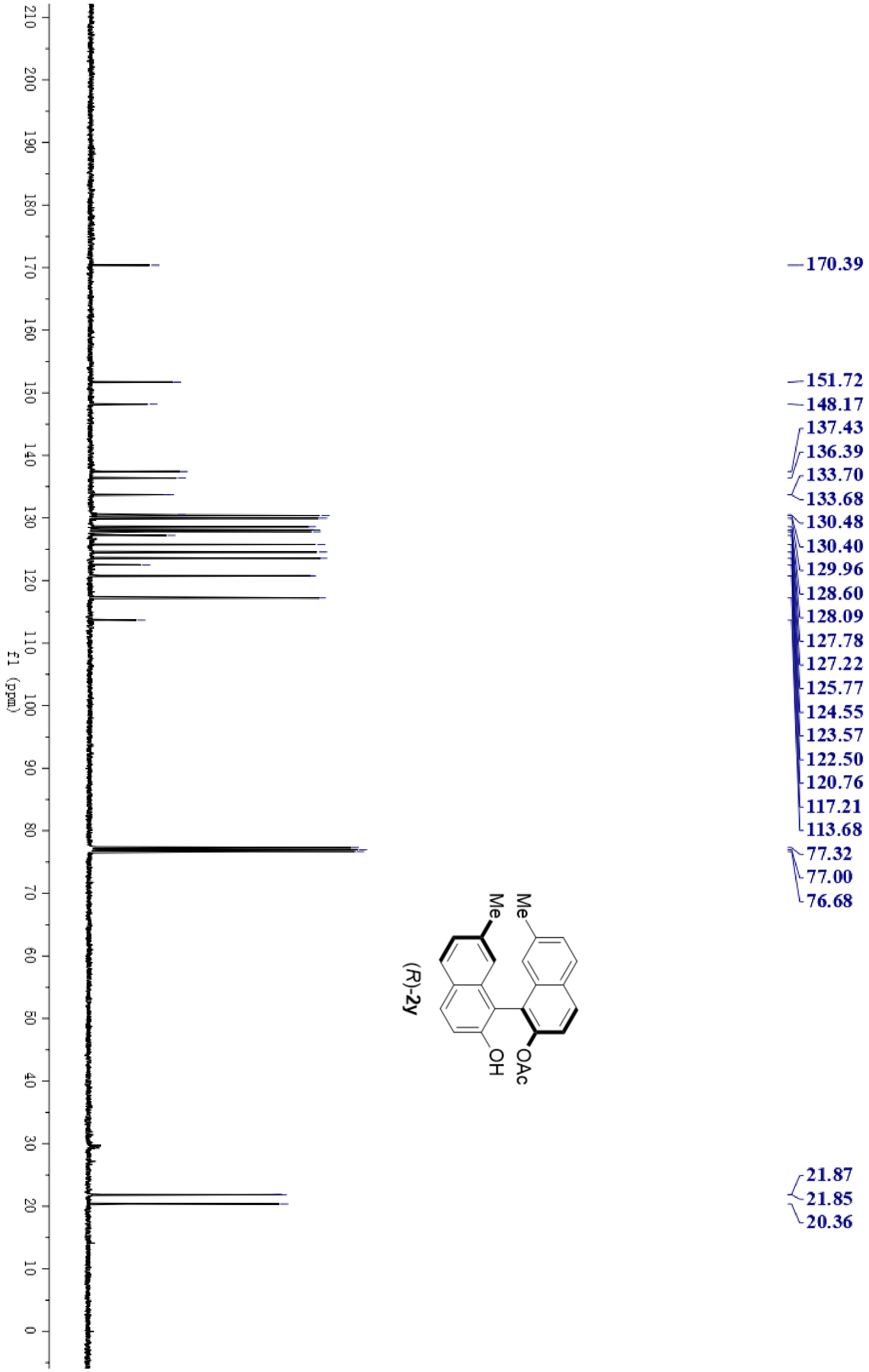
170.39
151.72
148.17
137.43
136.39
133.70
133.68
130.48
130.40
129.96
128.60
128.09
127.78
127.22
125.77
124.55
123.57
122.50
120.76
117.21
113.68
77.32
77.00
76.68
21.87
21.85
20.36
f1 (ppm)
Me
Me
OAc
OH
(R)-2y
