# Supplementary material for: Overcoming the Limitations of Transition-Metal Catalysis in the Chemoenzymatic Dynamic Kinetic Resolution (DKR) of Atropisomeric Bisnaphthols
Source: ACS Cent Sci. 2024 Nov 5;10(11):2099–110. doi: 10.1021/acscentsci.4c01370 (PMC11613327; doi:10.1021/acscentsci.4c01370)

checkCIF/PLATON report

You have not supplied any structure factors. As a result the full set of tests cannot be run.

THIS REPORT IS FOR GUIDANCE ONLY. IF USED AS PART OF A REVIEW PROCEDURE FOR PUBLICATION, IT SHOULD NOT REPLACE THE EXPERTISE OF AN EXPERIENCED CRYSTALLOGRAPHIC REFEREE.

No syntax errors found. CIF dictionary Interpreting this report

Datablock: mo_wk_7_11_0m_a

```
Bond precision:   C-C = 0.0060 A           Wavelength=0.71073

Cell:           a=11.9915(5)       b=14.8367(7)      c=15.2677(7)
                alpha=108.313(2)   beta=99.180(2)    gamma=103.501(2)
Temperature:    150 K
```

	Calculated	Reported
Volume	2426.4(2)	2426.39(19)
Space group	P -1	P -1
Hall group	-P 1	-P 1
Moiety formula	2(C26 H20 Cl2 Cu N2), C H2 Cl2 [+ solvent]	2(C26 H20 Cl2 Cu N2), C H2 Cl2
Sum formula	C53 H42 Cl6 Cu2 N4 [+ solvent]	C53 H42 Cl6 Cu2 N4
Mr	1074.71	1074.68
Dx,g cm-3	1.471	1.471
Z	2	2
Mu (mm-1)	1.248	1.248
F000	1096.0	1096.0
F000’	1099.30	
h,k,lmax	15,19,20	15,19,20
Nref	12041	11982
Tmin,Tmax	0.779,0.883	0.523,0.747
Tmin’	0.779	

```
Correction method= # Reported T Limits: Tmin=0.523 Tmax=0.747
AbsCorr = MULTI-SCAN

Data completeness= 0.995            Theta(max)= 28.282
```

R(reflections)= 0.0593(7828)

wR2(reflections)= 0.1680(11982)

S = 1.071

Npar= 590

The following ALERTS were generated. Each ALERT has the format
test-name_ALERT_alert-type_alert-level.
Click on the hyperlinks for more details of the test.

Alert level C

```
PLAT244_ALERT_4_C Low    'Solvent' Ueq as Compared to Neighbors of       C01R Check
PLAT341_ALERT_3_C Low Bond Precision on  C-C Bonds ...............     0.00604 Ang.
```

Alert level G

```
PLAT012_ALERT_1_G No       _shelx_res_checksum Found in CIF ......     Please Check
PLAT154_ALERT_1_G The s.u.'s on the Cell Angles are Equal ..(Note)      0.002 Degree
PLAT380_ALERT_4_G Incorrectly? Oriented X(sp2)-Methyl Moiety .....       C01D Check
PLAT605_ALERT_4_G Largest Solvent Accessible VOID in the Structure        130 A**3
PLAT720_ALERT_4_G Number of Unusual/Non-Standard Labels ..........        106 Note
PLAT868_ALERT_4_G ALERTS Due to the Use of _smtbx_masks Suppressed          ! Info
PLAT883_ALERT_1_G No Info/Value for _atom_sites_solution_primary .     Please Do !
```

0 **ALERT level A** = Most likely a serious problem - resolve or explain
0 **ALERT level B** = A potentially serious problem, consider carefully
2 **ALERT level C** = Check. Ensure it is not caused by an omission or oversight
7 **ALERT level G** = General information/check it is not something unexpected

3 ALERT type 1 CIF construction/syntax error, inconsistent or missing data
0 ALERT type 2 Indicator that the structure model may be wrong or deficient
1 ALERT type 3 Indicator that the structure quality may be low
5 ALERT type 4 Improvement, methodology, query or suggestion
0 ALERT type 5 Informative message, check

It is advisable to attempt to resolve as many as possible of the alerts in all categories. Often the minor alerts point to easily fixed oversights, errors and omissions in your CIF or refinement strategy, so attention to these fine details can be worthwhile. In order to resolve some of the more serious problems it may be necessary to carry out additional measurements or structure refinements. However, the purpose of your study may justify the reported deviations and the more serious of these should normally be commented upon in the discussion or experimental section of a paper or in the "special_details" fields of the CIF. checkCIF was carefully designed to identify outliers and unusual parameters, but every test has its limitations and alerts that are not important in a particular case may appear. Conversely, the absence of alerts does not guarantee there are no aspects of the results needing attention. It is up to the individual to critically assess their own results and, if necessary, seek expert advice.

Publication of your CIF in IUCr journals

A basic structural check has been run on your CIF. These basic checks will be run on all CIFs submitted for publication in IUCr journals (*Acta Crystallographica*, *Journal of Applied Crystallography*, *Journal of Synchrotron Radiation*); however, if you intend to submit to *Acta Crystallographica Section C* or *E* or *IUCrData*, you should make sure that full publication checks are run on the final version of your CIF prior to submission.

Publication of your CIF in other journals

Please refer to the *Notes for Authors* of the relevant journal for any special instructions relating to CIF submission.

PLATON version of 06/07/2023; check.def file version of 30/06/2023

Datablock mo_wk_7_11_0m_a - ellipsoid plot

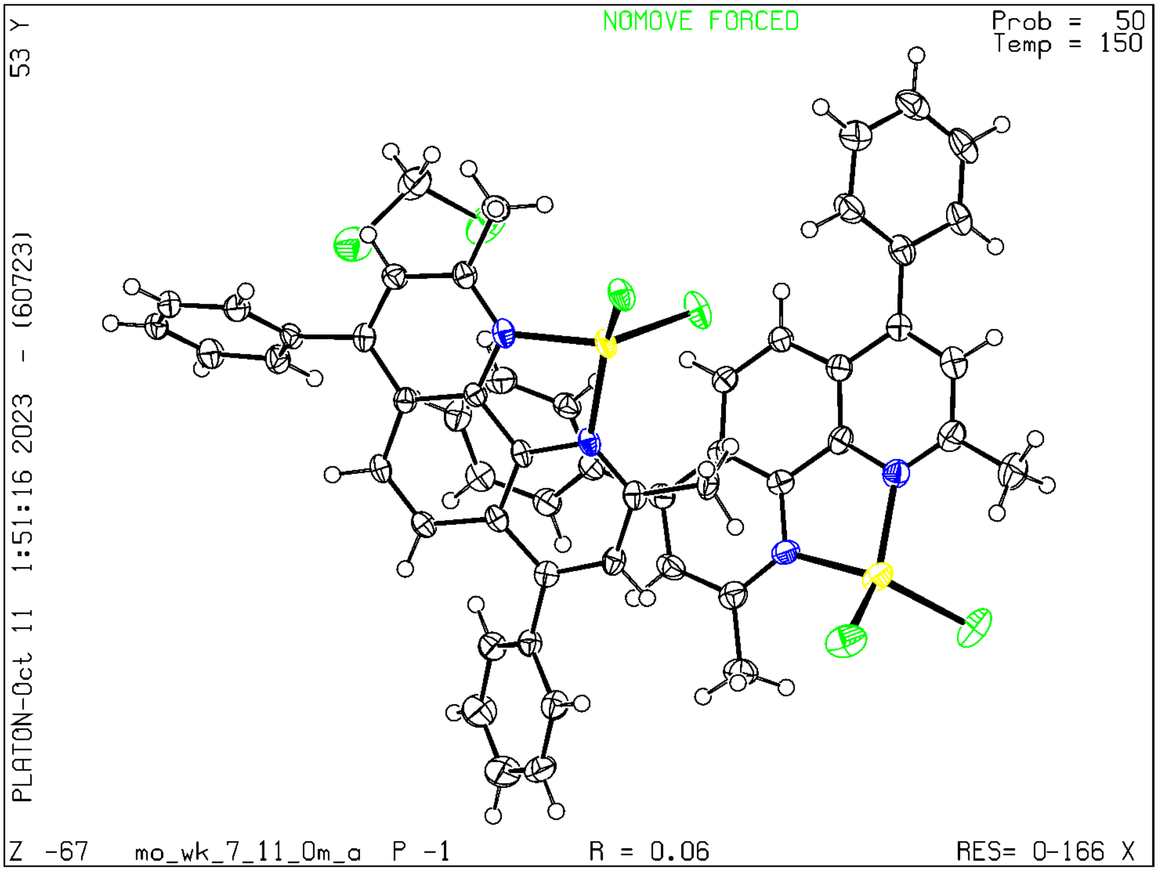

Supplement: Supplementary file 2 — oc4c01370_si_002.pdf [file oc4c01370_si_002.pdf]
