# Supplementary material for: Overcoming the Limitations of Transition-Metal Catalysis in the Chemoenzymatic Dynamic Kinetic Resolution (DKR) of Atropisomeric Bisnaphthols
Source: ACS Cent Sci. 2024 Nov 5;10(11):2099–110. doi: 10.1021/acscentsci.4c01370 (PMC11613327; doi:10.1021/acscentsci.4c01370)

checkCIF/PLATON report

You have not supplied any structure factors. As a result the full set of tests cannot be run.

THIS REPORT IS FOR GUIDANCE ONLY. IF USED AS PART OF A REVIEW PROCEDURE FOR PUBLICATION, IT SHOULD NOT REPLACE THE EXPERTISE OF AN EXPERIENCED CRYSTALLOGRAPHIC REFEREE.

No syntax errors found. CIF dictionary Interpreting this report

Datablock: mo_wk_1107_cucl2tmeda_0m_a

```
Bond precision:   C-C = 0.0040 A          Wavelength=0.71073

Cell:            a=7.6650(7)        b=8.2843(7)        c=9.1285(8)
                 alpha=76.513(3)    beta=85.305(4)     gamma=64.891(3)
Temperature:     150 K

                  Calculated                    Reported
Volume            510.31(8)                     510.31(8)
Space group       P -1                          P -1
Hall group        -P 1                          -P 1
Moiety formula    C6 H16 Cl2 Cu N2              C6 H16 Cl2 Cu N2
Sum formula       C6 H16 Cl2 Cu N2              C6 H16 Cl2 Cu N2
Mr                250.66                        250.65
Dx,g cm-3         1.631                         1.631
Z                 2                             2
Mu (mm-1)         2.608                         2.608
F000              258.0                         258.0
F000'             259.26
h,k,lmax          9,10,11                       9,10,11
Nref              1939                          1899
Tmin,Tmax         0.600,0.594                   0.470,0.746
Tmin'             0.588

Correction method= # Reported T Limits: Tmin=0.470 Tmax=0.746
AbsCorr = NONE

Data completeness= 0.979           Theta(max)= 25.674

                                                  wR2(reflections)=
R(reflections)= 0.0350( 1727)                     0.1048( 1899)

S = 1.078                   Npar= 104
```

```
The following ALERTS were generated. Each ALERT has the format
       test-name_ALERT_alert-type_alert-level.
Click on the hyperlinks for more details of the test.
```

Alert level G

```
PLAT012_ALERT_1_G No       _shelx_res_checksum Found in CIF ......     Please Check
PLAT720_ALERT_4_G Number of Unusual/Non-Standard Labels .........        27 Note
PLAT794_ALERT_5_G Tentative Bond Valency for Cu01      (I)       .      1.15 Info
PLAT883_ALERT_1_G No Info/Value for _atom_sites_solution_primary .     Please Do !
```

```
   0 ALERT level A = Most likely a serious problem - resolve or explain
   0 ALERT level B = A potentially serious problem, consider carefully
   0 ALERT level C = Check. Ensure it is not caused by an omission or oversight
   4 ALERT level G = General information/check it is not something unexpected

   2 ALERT type 1 CIF construction/syntax error, inconsistent or missing data
   0 ALERT type 2 Indicator that the structure model may be wrong or deficient
   0 ALERT type 3 Indicator that the structure quality may be low
   1 ALERT type 4 Improvement, methodology, query or suggestion
   1 ALERT type 5 Informative message, check
```

It is advisable to attempt to resolve as many as possible of the alerts in all categories. Often the minor alerts point to easily fixed oversights, errors and omissions in your CIF or refinement strategy, so attention to these fine details can be worthwhile. In order to resolve some of the more serious problems it may be necessary to carry out additional measurements or structure refinements. However, the purpose of your study may justify the reported deviations and the more serious of these should normally be commented upon in the discussion or experimental section of a paper or in the "special_details" fields of the CIF. checkCIF was carefully designed to identify outliers and unusual parameters, but every test has its limitations and alerts that are not important in a particular case may appear. Conversely, the absence of alerts does not guarantee there are no aspects of the results needing attention. It is up to the individual to critically assess their own results and, if necessary, seek expert advice.

Publication of your CIF in IUCr journals

A basic structural check has been run on your CIF. These basic checks will be run on all CIFs submitted for publication in IUCr journals (*Acta Crystallographica*, *Journal of Applied Crystallography*, *Journal of Synchrotron Radiation*); however, if you intend to submit to *Acta Crystallographica Section C* or *E* or *IUCrData*, you should make sure that full publication checks are run on the final version of your CIF prior to submission.

Publication of your CIF in other journals

Please refer to the *Notes for Authors* of the relevant journal for any special instructions relating to CIF submission.

PLATON version of 06/07/2023; check.def file version of 30/06/2023

Datablock mo_wk_1107_cucl2tmeda_0m_a - ellipsoid plot

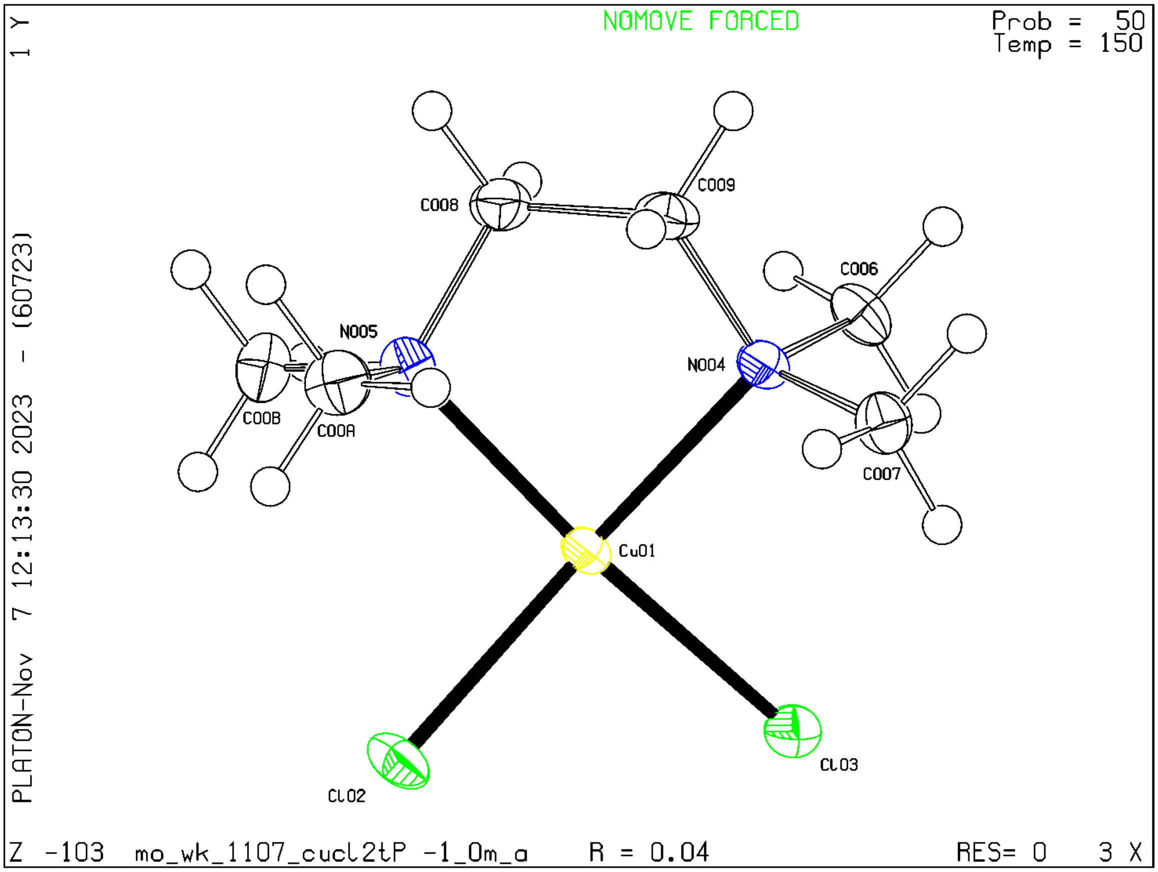

Supplement: Supplementary file 4 — oc4c01370_si_004.pdf [file oc4c01370_si_004.pdf]
